# Rh-catalyzed regiodivergent hydrosilylation of acyl aminocyclopropanes controlled by monophosphine ligands†
†Electronic supplementary information (ESI) available: Detailed experimental procedures, and spectral data for all compounds, including scanned images of ^1^H and ^13^C NMR spectra. See DOI: 10.1039/c7sc00071e



**DOI:** 10.1039/c7sc00071e

**Published:** 2017-03-15

**Authors:** Hiroki Kondo, Kenichiro Itami, Junichiro Yamaguchi

**Affiliations:** a Department of Chemistry , Graduate School of Science and Institute of Transformative Bio-Molecules (WPI-ITbM) , Nagoya University , Chikusa , Nagoya 464-8602 , Japan; b JST , ERATO , Itami Molecular Nanocarbon Project , Nagoya University , Chikusa , Nagoya 464-8602 , Japan; c Department of Applied Chemistry , Waseda University , 3-4-1 Ohkubo, Shinjuku , Tokyo 169-8555 , Japan . Email: junyamaguchi@waseda.jp

## Abstract

A Rh-catalyzed regiodivergent hydrosilylation of acyl aminocyclopropanes has been developed. Acyl aminocyclopropanes were reacted with hydrosilanes in the presence of Rh catalysts to afford ring-opened hydrosilylated adducts through carbon–carbon (C–C) bond cleavage of the cyclopropane ring. The regioselectivity of the addition of silanes (linear or branched) can be switched by changing the monophosphine ligand. This C–C bond cleavage/hydrosilylation methodology is applicable to the synthesis of silanediol precursors.

## Abstract 2

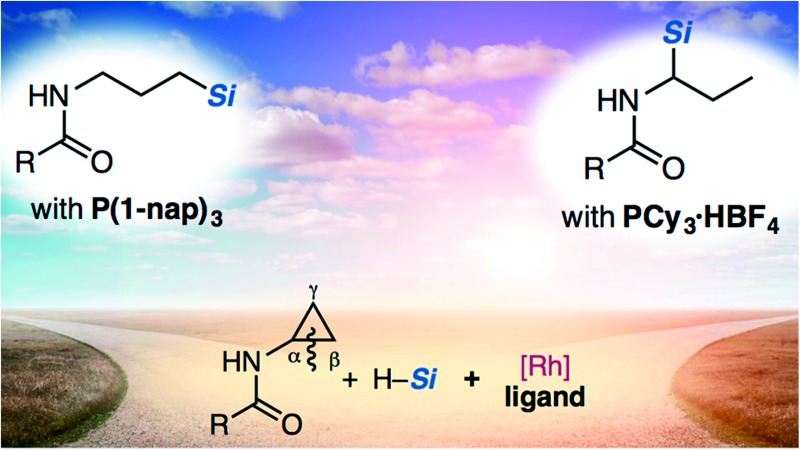
A Rh-catalyzed regiodivergent hydrosilylation of acyl aminocyclopropanes has been developed.

## Introduction

Catalytic carbon–carbon (C–C) bond activation can be an efficient way to create valuable bonds from readily available starting materials.^1^ The development of methodologies for both the breaking of C–C bonds and the forming of new C–C and C–heteroatom bonds enables the synthesis of complex organic molecules in a novel and more efficient manner. In previous studies of transition-metal catalyzed C–C bond cleavage reactions, strained molecules have usually been employed.^2,3^ Particularly, cyclopropanes have been well investigated due to their high reactivity derived from a high strain energy. Various types of ring-opening reactions of “activated” cyclopropanes such as alkylidenecyclopropanes and donor–acceptor cyclopropanes have been reported, enabling the construction of diverse frameworks. However, mono-substituted cyclopropanes, which have inert C–C bonds compared to the “activated” cyclopropanes described above, are still difficult to functionalize and have limited application in organic synthesis.^4^ Since mono-substituted cyclopropanes such as cyclopropylamine are readily available ($5 per gram from Sigma-Aldrich), its synthetic utility might be enhanced if it can be coaxed into a new C–C bond activation mode. Recently, the Bower group reported excellent examples for a C–C activation/alkene formation sequence using acyl aminocyclopropanes.^4j,k^

**Scheme 1:**
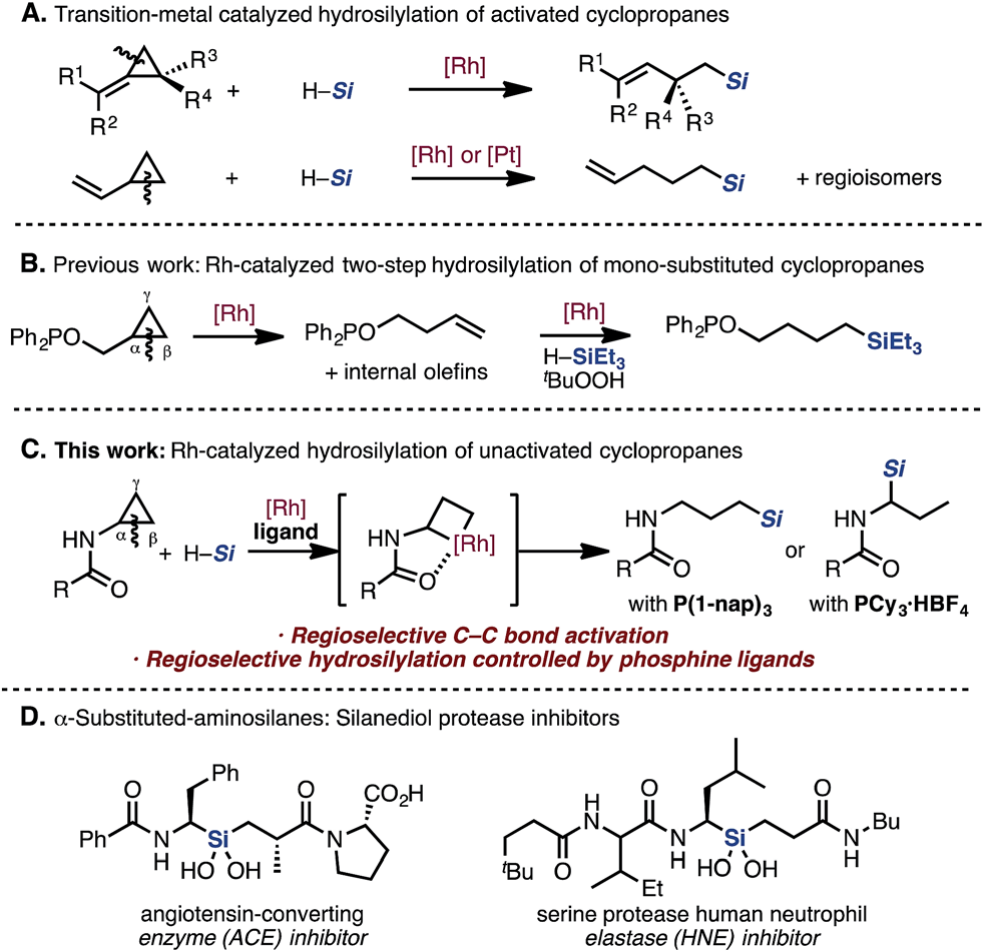
Transition-metal catalyzed hydrosilylation of cyclopropanes *via* C–C bond activation.

Hydrosilylation is a classical and well-studied reaction, particularly for double bonds such as alkenes, alkynes, ketones, and imines under transition-metal catalysis.^5^ However, regarding the hydrosilylation of C–C single bonds, only a narrow scope of selected cyclopropanes has been studied. Catalytic hydrosilylation of “activated” cyclopropanes is known, and the site-selectivity of the C–C bond cleavage depends on the substituents on both the substrate and the catalyst (Scheme 1A).^6,7^ Specifically, the catalytic hydrosilylation of mono-substituted cyclopropanes has only been demonstrated in one report.^7*b*^ In a related literature precedent, the Chirik group reported a two-step catalytic hydrosilylation of cyclopropyl alcohol in the presence of Wilkinson's catalyst (Scheme 1B).^4*c*^

They discovered that the proximal bond (C(α)–C(β) bond) of the cyclopropane can be cleaved selectively under the influence of the Rh catalyst by using PPh_2_ as a directing group, followed by hydrosilylation of the alkene to give the resulting alkylsilane.

During our efforts to develop C–H functionalization of aminocyclopropanes,^8^ we serendipitously discovered a Rh-catalyzed hydrosilylation of acyl aminocyclopropanes *via* C–C bond cleavage (Scheme 1C). We have found that the regioselectivity of the addition of silanes can be controlled by changing the monophosphine ligand to give linear or branched alkylsilanes. To the best of our knowledge, switching the regioselectivity by ligand control in a catalytic hydrosilylation of mono-substituted cyclopropanes has not been reported thus far. Additionally, since the functional motif of the resulting α-aminosilane is known to exhibit biological activity and is a prominent feature in silanediol protease inhibitors (Scheme 1D),^9^ many strategies for its preparation have been reported.^10^ This hydrosilylation methodology would introduce various silyl groups in an atom-economical fashion without the use of organometallic reagents.

## Results and discussion

At the outset of our studies, we chose cyclopropylamine **1A** and silane **2a** as model substrates (Table 1). To our delight, hydrosilylated product **3Aa**, which was formed through C(α)–C(β) bond cleavage and addition of the silyl group to the terminal carbon C(γ), was obtained in 40% yield with 2.5 mol% [Rh(cod)Cl]_2_ in THF at 120 °C for 6 h (Table 1, entry 1).^11^

**Table 1:** Investigation of the ligand effect in a Rh-catalyzed hydrosilylation of cyclopropylamine **1A** ^*a*^

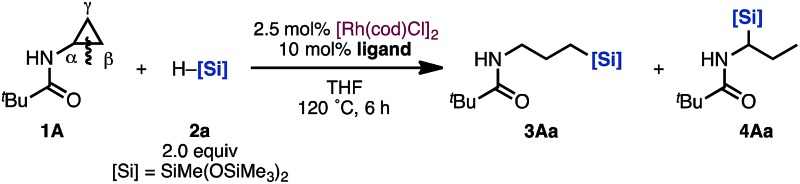			
Entry	Ligand	**3Aa** ^*b*^/%	**4Aa** ^*b*^/%
1	None	40^*c*^	<1
2	Phen	<1	<1
3	dppb	10	25
4	PCy_2_Ph	42	6
5^*d*^	PCy_3_	61 (56)^*c*^	<1
6	PPh_3_	17	83
7	P(1-nap)_3_	9	82 (81)^*c*^

^*a*^Conditions: **1A** (0.35 mmol), **2a** (2.0 equiv.), [Rh(cod)Cl]_2_ (2.5 mol%), ligand (bidentate: 5 mol%, monodentate: 10 mol%), THF (2.0 mL), 120 °C, 6 h.

^*b*^Yields were determined by ^1^H NMR analysis of the crude product using CH_2_Br_2_ as an internal standard.

^*c*^Isolated yield.

^*d*^PCy_3_·HBF_4_ was used as the precursor.

When examining ligand effects, we observed that 1,10-phenanthroline (phen), which is a bidentate nitrogen ligand, is ineffective (entry 2). When 1,4-bis(diphenylphosphino)butane (dppb) was employed, a mixture of linear product **3Aa** and branched product **4Aa** was obtained (entry 3). The monodentate phosphine ligand PCy_2_Ph provided **3Aa** as the major product (entry 4). Moreover, when a more electron-rich and bulky ligand, PCy_3_, was used, regioselective hydrosilylation proceeded to yield the linear **3Aa** in higher yield (56% isolated yield, entry 5).^12^ On the other hand, when PPh_3_ and tri(naphthalen-1-yl)phosphine (P(1-nap)_3_) were used as ligands, the regioselectivity dramatically changed: branched product **4Aa**, which has a silyl group at the α position to the nitrogen, was obtained in high yields (entries 6 and 7). In this manner, we accomplished a switching of the regioselectivity when reacting cyclopropylamine **1A** with hydrosilane **2a** *via* C–C bond cleavage solely by changing the ligand on the Rh catalyst.

**Scheme 2:**
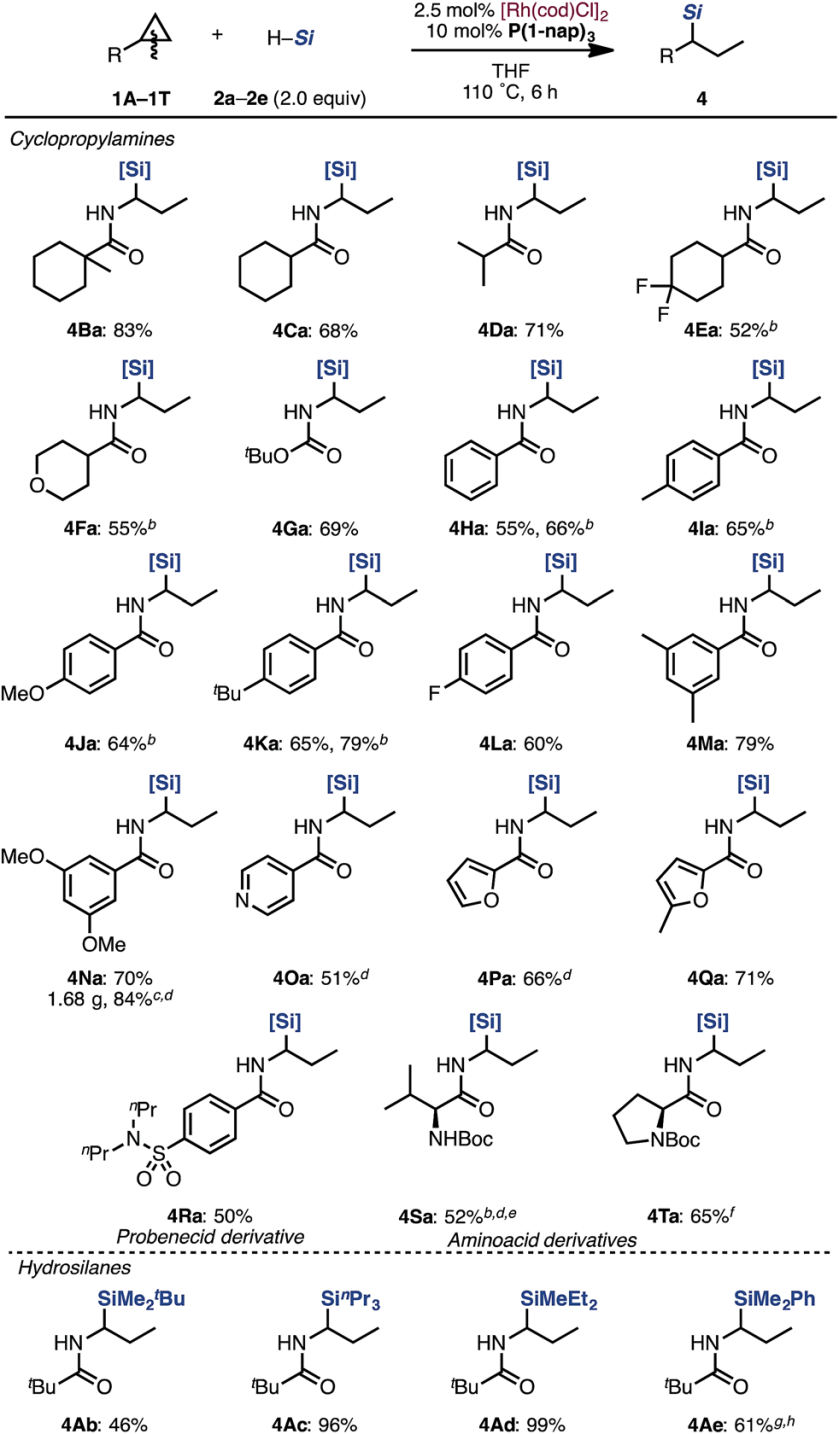
Scope of cyclopropylamines and hydrosilanes in the hydrosilylation by a Rh/P(1-nap)_3_ catalyst^a^. ^a^Isolated yields. ^b^[Rh(cod)OMe]_2_ (1.25 mol%), and P(1-nap)_3_ (5 mol%) were used. ^c^[Rh(cod)Cl]_2_ (1.25 mol%), and P(1-nap)_3_ (5 mol%) were used. ^d^18 h. ^e^Diastereomer mixtures. ^f^Mixture of diastereomers (60 : 40). ^g^[Rh(cod)Cl]_2_ (5 mol%), and P(1-nap)_3_ (20 mol%) were used. ^h^12 h. [Si] = SiMe(OSiMe_3_)_2_.

With optimized reaction conditions in hand, we evaluated the scope of cyclopropylamines and silanes under the [Rh(cod)Cl]_2_/P(1-nap)_3_ catalytic system (Scheme 2).

Cyclopropanes bearing cyclohexyl, isopropyl and tetrahydropyranyl groups were applicable in this reaction (**4Ba–4Fa**). Even carbamate **1G** performed well to give the corresponding product **4Ga** in 69% yield. Aminocyclopropanes protected by various benzoyl groups were tolerated to produce the desired products in good yields (**4Ha–4Na**). Cyclopropylamines with heteroaromatic substituents such as pyridine and furan rings were tolerated under the reaction conditions to afford hydrosilylated adducts in moderate yields (**4Oa–4Qa**). Probenecid derivative **1R** could be transformed to alkylsilane **4Ra**. Valine derivative **1S** and proline derivative **1T** can also be applied to this reaction, furnishing **4Sa** and **4Ta** in moderate yields. Various trialkylsilanes **1b–1e** were also applicable and gave the corresponding hydrosilylated products in moderate to excellent yields (**4Ab–4Ae**). Typically, the regioselectivity of branched: linear products was in the range of 10 : 1 to 3 : 1.^13^

**Scheme 3:**
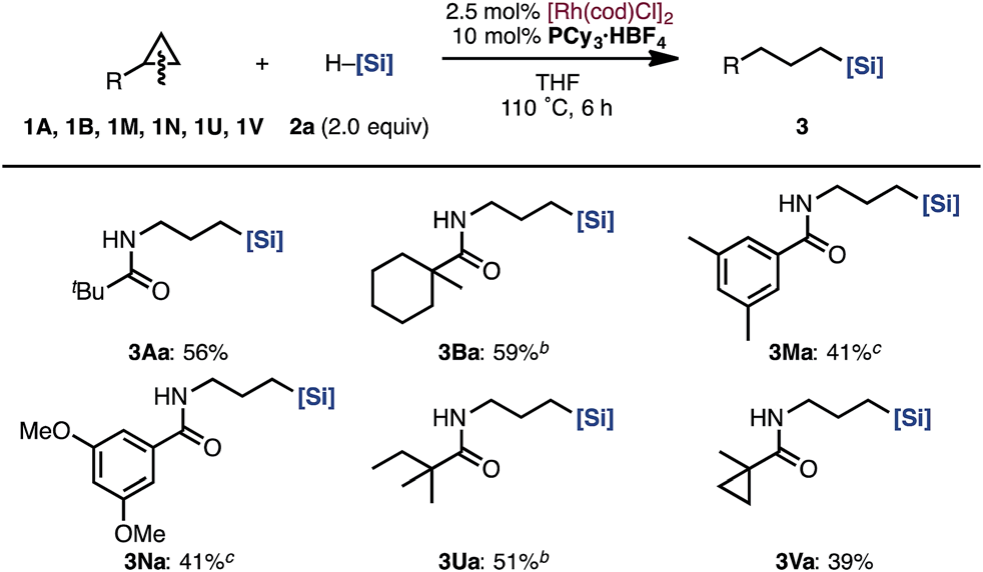
Scope of cyclopropylamines in the hydrosilylation by a Rh/PCy_3_·HBF_4_ catalyst^a^. ^a^Isolated yields. ^b^12 h. ^c^25 h. [Si] = SiMe(OSiMe_3_)_2_.

Next, the substrate scope of aminocyclopropanes in the [Rh(cod)Cl]_2_/PCy_3_·HBF_4_ catalytic system was examined (Scheme 3). The reaction of cyclopropylamines bearing various acyl protecting groups proceeded smoothly with virtually complete regioselectivities to provide the corresponding linear products (**3Aa**, **3Ba**, **3Ma**, **3Na**, **3Ua**, and **3Va**) in moderate yields.^14^

**Scheme 4:**
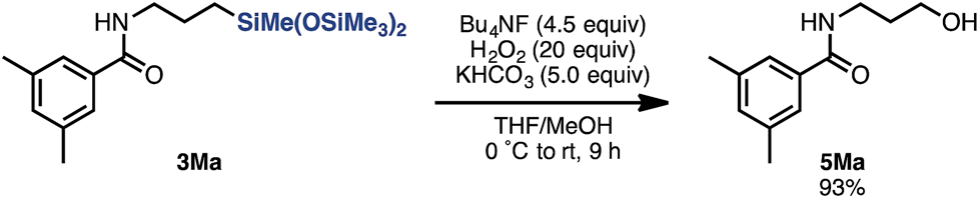
Tamao oxidation of aminosilane **3Ma**.

To demonstrate the synthetic applicability of the linear product **3**, alkylsilane **3Ma** was converted to the corresponding alcohol **5Ma** by treatment with Bu_4_NF/H_2_O_2_/KHCO_3_ in THF/MeOH (Scheme 4).^15^ In this manner, this hydrosilylation methodology can give access to a versatile building block for further transformation.

**Scheme 5:**
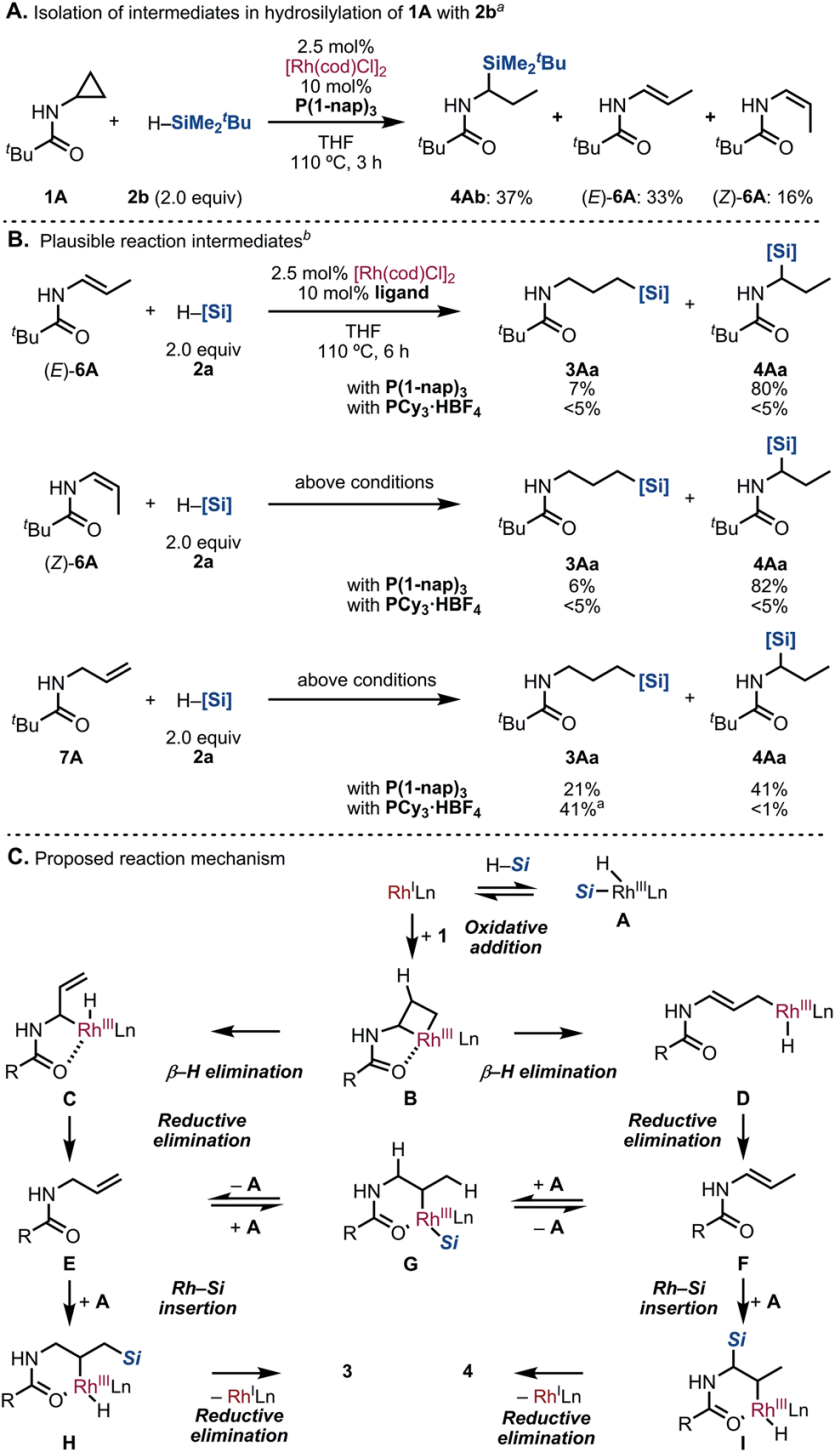
Mechanistic exploration. ^a^Isolated yields. ^b^Yields were determined by ^1^H NMR analysis of the crude product using CH_2_Br_2_ as an internal standard.

To gain insight into the reaction mechanism, several experiments were conducted (Scheme 5). Firstly, we tried to isolate the reaction intermediates. Gratifyingly, hydrosilylated product **4Ab** as well as (*E*)- and (*Z*)-enamide **6A** were isolated when the reaction of **1A** with *tert*-butyldimethylsilane (**2b**) in the presence of [Rh(cod)Cl]_2_/P(1-nap)_3_ was terminated at only 3 h of reaction time (Scheme 5A). This result indicated that the C–C bond cleavage generates the corresponding olefins *in situ*. Next, the reaction intermediates were subjected to our standard conditions (Scheme 5B). When (*E*)-**6A** and (*Z*)-**6A** were reacted with silane **2a** by using P(1-nap)_3_ as the ligand, the branched **4Aa** was obtained in 80% yield. This result is consistent with that of the hydrosilylation of **1A** (Table 1, entry 7).^16^ On the other hand, when PCy_3_·HBF_4_ was employed, the hydrosilylated products did not form.^12^ Additionally, when we subjected allylamine **7A**, which is another possible intermediate, under the same reaction conditions,^17^ **3Aa** was obtained in 21% yield along with 41% yield of **4Aa**. This suggested that the isomerization of the olefin took place *in situ*. On the other hand, the hydrosilylation of **7A** using PCy_3_·HBF_4_ afforded the linear product **3Aa** with high regioselectivity.^12^

According to our observations, a possible reaction mechanism is depicted (Scheme 5C). First, oxidative addition of the C(α)–C(β) bond to the Rh(i) catalyst occurs to generate rhodacycle **B**. Allylamine **E** and enamide **F** could be formed *via* β-H elimination, followed by reductive elimination from **C** and **D**. Both **E** and **F** would exist in equilibrium through intermediate **G**. Subsequently, Rh-catalyzed hydrosilylation of **E** or **F** would proceed by a modified Chalk–Harrod mechanism, giving **H** and **I**.^18^ Finally, a reductive elimination step yields **3** and **4** as the products.

**Fig. 1:**
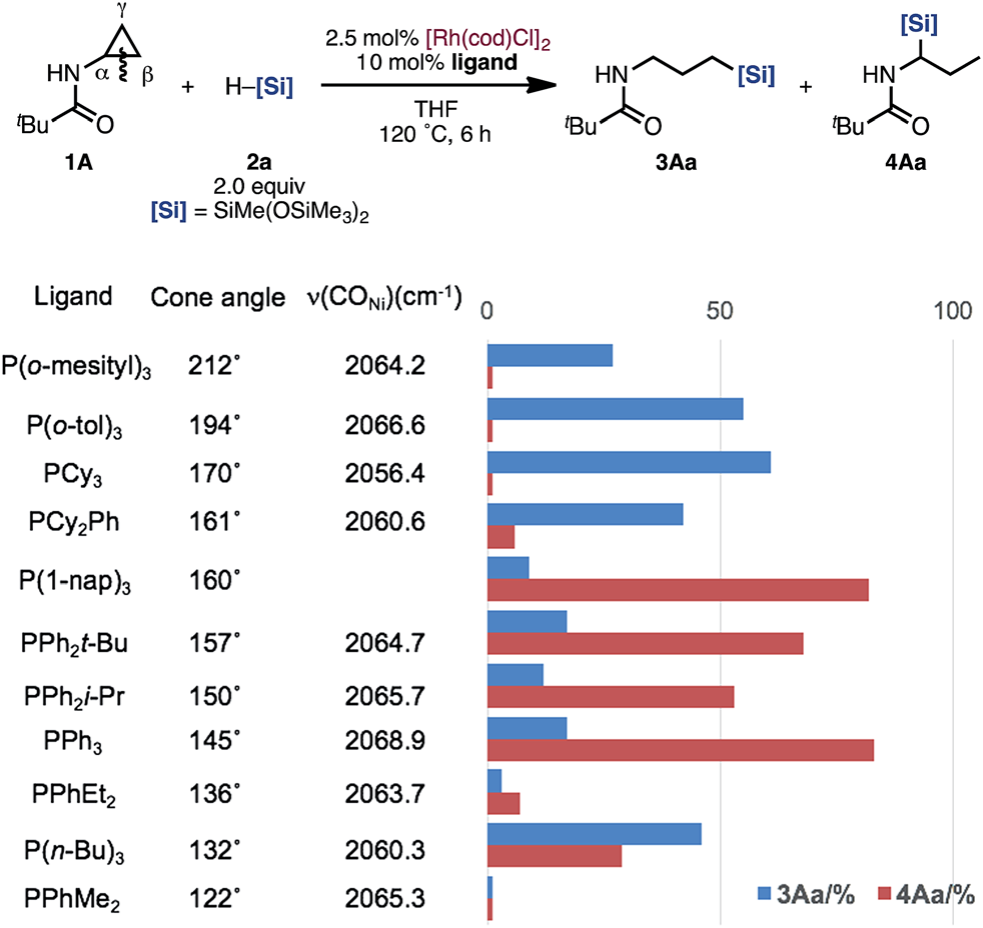
Relationship between the cone angle of ligands, the IR stretching frequency of the Ni(CO)_3_(L) catalysts, and the regioselectivity of hydrosilylation of acyl aminocyclopropanes.

Although the ligand effect remains unclear, we propose the following explanation: when PCy_3_ is used as the ligand, an electron-rich Rh complex with a high reduction ability is formed. Thus, enamides and imines, generated by isomerization, are hydrogenated immediately. This undesired pathway competes with the formation of the branched **4**, leading to the high regioselectivity of the linear **3**. On the other hand, enamide **6A** was selectively formed in the reaction of allylamine **7A** under [Rh(cod)Cl]_2_/P(1-nap)_3_ catalytic conditions.^19^ This result suggests that P(1-nap)_3_ is promoting the isomerization of allylamines to enamides. Furthermore, the regioselectivity might be correlated to the cone angle of the ligands (Fig. 1).^20^ Ligands with larger cone angles favor the linear product **3Aa**, whereas ones with smaller cone angles tend to produce branched product **4Aa**. Also, the IR stretching frequency of the Ni(CO)_3_(L) (L = ligand) catalysts did not show any relationship between the regioselectivity and the electronic *cis* effect of the phosphines.^20^ These factors suggest that the bulkiness of the ligand suppresses the hydrosilylation of the more sterically hindered enamide **6A** compared to allylamine **7A**.

## Conclusions

We have successfully developed a ligand-controlled, Rh-catalyzed regiodivergent hydrosilylation of acyl aminocyclopropanes with a wide range of silanes. Initial mechanistic investigations suggest that selective C–C bond cleavage takes place to generate allylamine and enamide, which are hydrosilylated by the Rh catalyst. Precise elucidation of ligand effects and application to asymmetric hydrosilylation are ongoing in our laboratory.

## References

[cit1] (s) C–C Bond Activation, ed. G. Dong, Springer, 2014.

[cit2] Nakamura I., Yamamoto Y. (2002). Adv. Synth. Catal..

[cit3] Seiser T., Saget T., Tran D. N., Cramer N. (2011). Angew. Chem., Int. Ed..

[cit4] Ichiyanagi T., Kuniyama S., Shimizu M., Fujisawa T. (1997). Chem. Lett..

[cit5] Riant O., Mostefaï N., Courmarcel J. (2004). Synthesis.

[cit6] Bessmertnykh A. G., Blinov K. A., Grishin Y. K., Donskaya N. A., Beletskaya I. P. (1995). Tetrahedron Lett..

[cit7] Hwu J. R. (1985). J. Chem. Soc., Chem. Commun..

[cit8] Miyamura S., Araki M., Suzuki T., Yamaguchi J., Itami K. (2015). Angew. Chem., Int. Ed..

[cit9] Mutahi M. W., Nittoli T., Guo L., Sieburth S. M. (2002). J. Am. Chem. Soc..

[cit10] (b) Kato K. Hirano K. Miura M., Angew. Chem., Int. Ed., 2016, 55 , 14400 , and references therein . 10.1002/anie.201608139 27754577

[cit11] Another ring-opened product was obtained, see the ESI.

[cit12] *N*-Propylpivalamide, which was generated *via* C–C bond cleavage and hydrogenation, was formed, see the ESI.

[cit13] For details regarding the regioselectivities of each product, see the ESI.

[cit14] The main byproducts were desilylated products in 10–30% yield. When *tert*-butyl acrylate was added as a hydrogen scavenger, these byproducts were suppressed to less than 10% yield. However, in such cases, the product yields did not increase, as the amount of recovered starting material 1 increased

[cit15] Tamao K., Ishida N., Tanaka T., Kumada M. (1983). Organometallics.

[cit16] Murai T., Oda T., Kimura F., Onishi H., Kanda T., Kato S. (1994). J. Chem. Soc., Chem. Commun..

[cit17] An example of Rh-catalyzed hydrosilylation of allylamine: Huckaba A. J. Hollis T. K. Reilly S. W., Organometallics, 2013, 32 , 6248 .

[cit18] Sakaki S., Sumimoto M., Fukuhara M., Sugimoto M., Fujimoto H., Matsuzaki S. (2002). Organometallics.

[cit19] See the ESI.

[cit20] Tolman C. A. (1977). Chem.
Rev..

