# Supplementary material for: Rh-catalyzed regiodivergent hydrosilylation of acyl aminocyclopropanes controlled by monophosphine ligands
Source: Chem Sci. 2017 Mar 15;8(5):3799–803. doi: 10.1039/c7sc00071e (PMC5436550; doi:10.1039/c7sc00071e)

Electronic Supplementary Material (ESI) for Chemical Science.
This journal is © The Royal Society of Chemistry 2017

Supplementary Information

Rh-Catalyzed Regiodivergent Hydrosilylation of Acyl aminocyclopropanes Controlled by Monophosphine Ligands

Hiroki Kondo,[1] Kenichiro Itami,[1,2] and Junichiro Yamaguchi*[3]

[1] *Department of Chemistry, Graduate School of Science and Institute of Transformative Bio-Molecules (WPI-ITbM), Nagoya University, Chikusa, Nagoya 464-8602, Japan*
[2] *JST, ERATO, Itami Molecular Nanocarbon Project, Nagoya University, Chikusa, Nagoya 464-8602, Japan*
[3] *Department of Applied Chemistry, Waseda University, 3-4-1 Ohkubo, Shinjuku, Tokyo 169-8555, Japan.*

E-mail: junyamaguchi@waseda.jp

Table of Contents

1. General S2
2. Preparation of Substituted Aminocyclopropanes S3-S9
3. General Procedure for Hydrosilylation with $[Rh(cod)Cl]_2/P(1\text{-nap})_3$ S10-S19
4. General Procedure for Hydrosilylation with $[Rh(cod)Cl]_2/PCy_3\cdot HBF_4$ S20-S22
5. Synthetic Applications S23-S24
6. Mechanistic Considerations S25-S29
7. Proposed Reaction Mechanism S30
8. Effect of Reaction Parameters S31
9. Discovery of Rh-catalyzed Hydrosilylation S32
10. ^{1}H and ^{13}C NMR Spectra S33-S134

1. General

Unless otherwise noted, all reactants or reagents including dry solvents were obtained from commercial suppliers and used as received. 1,4-Bis(diphenylphosphino)butane (dppb), 1,1,1,3,5,5,5-heptamethyltrisiloxane (**2a**) and dimethyl(phenyl)silane (**2e**) were obtained from TCI Chemical. Triphenylphosphine, tricyclohexylphosphonium tetrafluoroborate ($PCy_3 \cdot HBF_4$) and *tert*-butyldimethylsilane (**2b**) were obtained from Wako Chemicals. 1,10-Phenanthroline (phen), dicyclohexylphenylphosphine (PCy_2Ph), tri(naphthalen-1-yl)phosphine ($P(1\text{-nap})_3$), tripropylsilane (**2c**), diethylmethylsilane (**2d**) and $[Rh(cod)OMe]_2$ were obtained from Sigma-Aldrich. $[Rh(cod)Cl]_2$ was prepared by according to a procedure reported in the literature.[1] Unless otherwise noted, all reactions were performed with dry solvents under an atmosphere of nitrogen in dried glassware using standard vacuum-line techniques. All hydrosilylation reactions were performed in 20-mL glass vessel tubes equipped with a J. Young® O-ring tap and heated in an 8-well reaction block (heater + magnetic stirrer). All work-up and purification procedures were carried out with reagent-grade solvents in air.

Analytical thin-layer chromatography (TLC) was performed using E. Merck silica gel 60 F_{254} precoated plates (0.25 mm). The developed chromatogram was analyzed by UV lamp (254 nm) or a phosphomolybdic acid/sulfuric acid solution. Flash column chromatography was performed with E. Merck silica gel 60 (230–400 mesh) or Biotage Isolera® equipped with Biotage SNAP Cartridge KP-Sil columns using hexane/ethyl acetate as eluent. Medium-pressure liquid chromatography (MPLC) was performed using Yamazen W-prep 2XY. Preparative thin-layer chromatography (PTLC) was performed using Wakogel B5-F silica coated plates (0.75 mm) prepared in our laboratory. Preparative gel permeation chromatography (GPC) was performed with a JAI LC-9204 instrument equipped with JAIGEL-1H/JAIGEL-2H columns using chloroform as eluent. LCMS analysis was conducted on an *Agilent 6100* instrument equipped with Poroshell 120 EC-C18 column (2.1x100 nm, 2.7 um) using acetonitrile/5 mM $HCOONH_4$ in water as eluent. High-resolution mass spectra (HRMS) were obtained from Thermo Fisher Scientific Exactive (ESI and DART). Nuclear magnetic resonance (NMR) spectra were recorded on a JEOL JNM-ECA-600 spectrometer (^{1}H 600 MHz, ^{13}C 151 MHz), a JEOL JNM-ECA-500 spectrometer (^{1}H 500 MHz, ^{13}C 126 MHz) and a JEOL JNM-ECA-400 spectrometer (^{1}H 400 MHz, ^{13}C 101 MHz). Chemical shifts for ^{1}H NMR are expressed in parts per million (ppm) relative to tetramethylsilane (δ 0.00 ppm) or residual peak of DMSO (δ 2.50 ppm) or CH_2Cl_2 (δ 5.32 ppm). Chemical shifts for ^{13}C NMR are expressed in ppm relative to $CDCl_3$ (δ 77.0 ppm) or DMSO (δ 39.5 ppm) or CD_2Cl_2 (δ 53.84 ppm). Data are reported as follows: chemical shift, multiplicity (s = singlet, d = doublet, dd = doublet of doublets, t = triplet, dt = doublet of triplets, td = triplet of doublets, q = quartet, quin = quintet, sext = sextet, m = multiplet, brs = broad singlet), coupling constant (Hz), and integration.

[1] Uson, R.; Oro, L. A.; Cabeza, J. A. ; Bryndza, H. E. ; Stepro, M. P. *Inorg. Synth.* **1985**, *23*, 126.

2. Preparation of Substituted Aminocyclopropanes

Note: *N*-Cyclopropylpivalamide (**1A**),[2] *N*-cyclopropylcyclohexanecarboxamide (**1C**),[2] *N*-cyclopropylisobutyramide (**1D**),[2] *tert*-butyl cyclopropylcarbamate (**1G**),[2] *N*-cyclopropyl-4-methylbenzamide (**1I**),[3] and *N*-cyclopropyl-4-methoxybenzamide (**1J**)[4] were synthesized according to procedures reported in the literature.

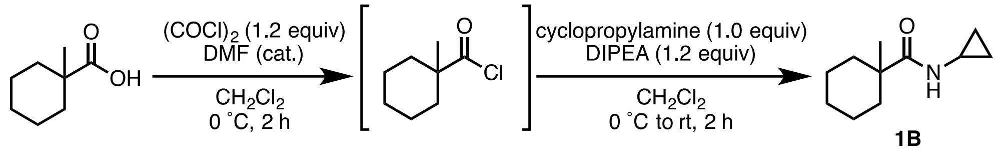

***N*-Cyclopropyl-1-methylcyclohexane-1-carboxamide (1B)**: To a solution of 1-methylcyclohexane-1-carboxylic acid (1.1 g, 9.5 mmol) and *N*,*N*-dimethylformamide (DMF: 0.1 mL) in dichloromethane (50 mL) was slowly added $(COCl)_2$ (0.97 mL, 11.4 mmol) at 0 ˚C. The reaction mixture was stirred at 0 ˚C for 2 h. The reaction mixture was evaporated. The crude acid chloride and *N*,*N*-diisopropylethylamine (DIPEA: 2.0 mL, 11.4 mmol) were dissolved in dichloromethane (50 mL). To this solution was slowly added cyclopropylamine (1.5 mL, 9.5 mmol) at 0 ˚C. The reaction mixture was stirred at room temperature for 2 h. The mixture was extracted with dichloromethane and the combined organic layers were washed with brine, dried over Na_2SO_4, filtered and concentrated *in vacuo*. The residue was purified by Isolera® (hexane/ethyl acetate = 2:1 to 0:1). The obtained solid was washed with hexane to afford **1B** (1.5 g, 87%) as a orange solid. ^{1}H NMR (600 MHz, $CDCl_3$) δ 5.70 (brs, 1H), 2.74–2.68 (m, 1H), 1.89–1.81 (m, 2H), 1.57–1.50 (m, 2H), 1.49–1.37 (m, 3H), 1.36–1.27 (m, 3H), 1.11 (s, 3H), 0.80–0.74 (m, 2H), 0.47–0.42 (m, 2H); ^{13}C NMR (151 MHz, $CDCl_3$) δ 179.1, 42.4, 35.6, 26.2, 25.8, 22.8, 22.7, 6.7; HRMS (ESI) *m*/*z* calcd for $C_{11}H_{19}NNaO$ $[M+Na]^+$: 204.1359, found 204.1364.

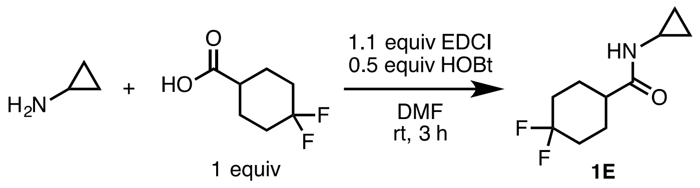

***N*-Cyclopropyl-4,4-difluorocyclohexane-1-carboxamide (1E):** To a round-bottom flask was added 4,4-difluorocyclohexane-1-carboxylic acid (443 mg, 2.7 mmol), 1-ethyl-3-(3-dimethylaminopropyl)carbodiimide (EDCI: 569 mg, 3.0 mmol, 1.1 equiv), 1-hydroxybenzotriazole

[2] Miyamura, S.; Araki, M.; Suzuki, T.; Yamaguchi, J.; Itami, K. *Angew. Chem., Int. Ed.* **2015**, *54*, 846.

[3] Zheng, Y.; Liu, B.; Gou, Z.; Li, Y.; Zhang, X.; Wang, Y.; Yu, S.; Li, Y.; Sun, D. *Bioorg. Med. Chem. Lett.* **2015**, *25*, 791.

[4] Baburajan, P.; Elango, K. P. *Tetrahedron Lett.* **2014**, *55*, 1006.

(HOBt: 182 mg, 1.4 mmol, 0.5 equiv) and DMF (10 mL). Cyclopropylamine (0.19 mL, 2.7 mmol) was added to the mixture. After stirring for 3 h at room temperature, saturated aqueous $NaHCO_3$ was added to the mixture and the mixture was extracted with ethyl acetate. The organic layers were washed with brine, dried over Na_2SO_4 and concentrated *in vacuo*. The residue was purified by Isolera® (hexane/ethyl acetate = 1:1 to 0:1). The obtained solid was crystallized from hexane to afford **1E** (372 mg, 68%) as a white solid. ^{1}H NMR (600 MHz, $CDCl_3$) δ 5.54 (brs, 1H), 2.74–2.69 (m, 1H), 2.21–2.07 (m, 3H), 1.94–1.87 (m, 2H), 1.85–1.66 (m, 4H), 0.81–0.76 (m, 2H), 0.49–0.45 (m, 2H); ^{13}C NMR (126 MHz, $CDCl_3$) δ 175.3, 122.6 (t, J = 239.6 Hz), 42.6, 32.8 (t, J = 23.9 Hz), 25.8 (t, J = 9.5 Hz), 22.6, 6.7; HRMS (ESI) *m*/*z* calcd for $C_{10}H_{15}F_2NNaO$ $[M+Na]^+$: 226.1014, found 226.1014.

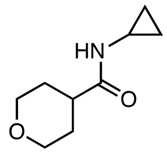

1F

***N*-Cyclopropyltetrahydro-2*H*-pyran-4-carboxamide (1F):** The synthetic procedure of **1F** is the same as that of **1E**. 6.0 mmol scale. Purification by Isolera® (hexane/ethyl acetate = 1:1 to 0:1); the obtained solid was crystallized from hexane and ethyl acetate to afford **1F** (437 mg, 43%) as a white solid. ^{1}H NMR (600 MHz, $CDCl_3$) δ 5.55 (brs, 1H), 4.03–3.98 (m, 2H), 3.39 (td, J = 11.4, 2.4 Hz, 2H), 2.74–2.69 (m, 1H), 2.30–2.23 (m, 1H), 1.82–1.70 (m, 4H), 0.81–0.75 (m, 2H), 0.50–0.45 (m, 2H); ^{13}C NMR (126 MHz, $CDCl_3$) δ 175.6, 67.2, 42.0, 29.2, 22.6, 6.7; HRMS (ESI) *m*/*z* calcd for $C_9H_{15}NNaO_2$ $[M+Na]^+$: 192.0995, found 192.0996.

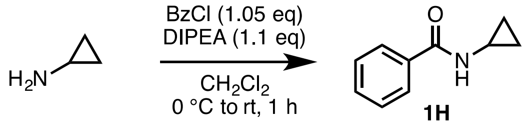

***N*-Cyclopropylbenzamide (1H)[5]:** To a solution of cyclopropylamine (0.49 mL, 7.0 mmol) and *N,N*-diisopropylethylamine (DIPEA: 1.3 mL, 7.7 mmol, 1.1 equiv) in dichloromethane (10 mL) was slowly added a solution of benzoyl chloride (BzCl: 0.85 mL, 7.4 mmol, 1.05 equiv) in dichloromethane (5 mL) at 0 °C. The reaction mixture was stirred at room temperature for 1 h. To the reaction mixture was added saturated aqueous $NaHCO_3$, which was then extracted with dichloromethane. The combined organic layers were washed with brine, dried over Na_2SO_4, filtered and concentrated *in vacuo*. The residue was purified by MPLC (hexane/ethyl acetate = 5:1 to 2:1). The obtained solid was crystallized from hexane and ethyl acetate to give **1H** (1.1 g, 97%) as a white solid. ^{1}H NMR (600 MHz, $CDCl_3$) δ 7.73 (dd, J = 7.8,

[5] Lin, J.-P.; Long, Y.-Q. *Chem. Commun.* **2013**, *49*, 5313.

1.8 Hz, 2H), 7.51–7.47 (m, 1H), 7.42 (t, *J* = 1.8 Hz, 2H), 6.23 (brs, 1H), 2.94–2.89 (m, 1H), 0.91–0.84 (m, 2H), 0.65–0.60 (m, 2H); ^{13}C NMR (126 MHz, $CDCl_3$) δ 168.9, 134.4, 131.4, 128.5, 126.8, 23.1, 6.8; HRMS (ESI) *m*/*z* calcd for $C_{10}H_{11}NNaO$ $[M+Na]^+$: 184.0733, found 184.0734.

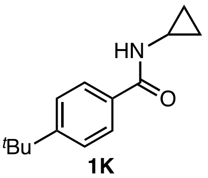

1K

4-(*tert*-Butyl)-*N*-cyclopropylbenzamide (1K): The synthetic procedure of **1K** is the same as that of **1H**. 4.0 mmol scale. Purification by MPLC (hexane/ethyl acetate = 2:1); the obtained solid was crystallized from hexane and ethyl acetate to afford **1K** (720 mg, 83%) as a white solid. ^{1}H NMR (600 MHz, $CDCl_3$) δ 7.67 (d, *J* = 8.4 Hz, 2H), 7.42 (d, *J* = 8.4 Hz, 2H), 6.22 (brs, 1H), 2.92–2.87 (m, 1H), 1.32 (s, 9H), 0.90–0.83 (m, 2H), 0.64–0.58 (m, 2H); ^{13}C NMR (151 MHz, $CDCl_3$) δ 168.7, 154.9, 131.6, 126.6, 125.4, 34.9, 31.1, 23.0, 6.8; HRMS (ESI) *m*/*z* calcd for $C_{14}H_{19}NNaO$ $[M+Na]^+$: 240.1359, found 240.1357.

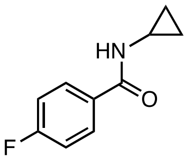

1L

***N*-Cyclopropyl-4-fluorobenzamide (1L):** The synthetic procedure of **1L** is the same as that of **1H**. 21.3 mmol scale. Purification by MPLC (hexane/ethyl acetate = 3:1 to 1:1); the obtained solid was crystallized from hexane and ethyl acetate to afford **1L** (2.1 g, 55%) as a white solid. ^{1}H NMR (600 MHz, $CDCl_3$) δ 7.78–7.72 (m, 2H), 7.12–7.06 (m, 2H), 6.20 (brs, 1H), 2.92–2.86 (m, 1H), 0.92–0.83 (m, 2H), 0.66–0.58 (m, 2H); ^{13}C NMR (151 MHz, $CDCl_3$) δ 167.8, 164.7 (d, J_{FC} = 250.2 Hz), 130.6 (d, J_{FC} = 2.9 Hz), 129.1 (d, J_{FC} = 8.6 Hz), 115.5 (d, J_{FC} = 21.6 Hz), 23.2, 6.7; HRMS (ESI) *m*/*z* calcd for $C_{10}H_{10}FNNaO$ $[M+Na]^+$: 202.0639, found 202.0639.

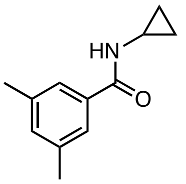

1M

***N*-Cyclopropyl-3,5-dimethylbenzamide (1M):** The synthetic procedure of **1M** is the same as that of **1H**. 3.5 mmol scale. Purification by MPLC (hexane/ethyl acetate = 3:1 to 1:1); the obtained solid was

crystallized from hexane and ethyl acetate to afford **1M** (250 mg, 38%) as a white solid. ^{1}H NMR (600 MHz, $CDCl_3$) δ 7.33 (s, 2H), 7.11 (s, 1H), 6.15 (brs, 1H), 2.92–2.87 (m, 1H), 2.34 (s, 6H), 0.90–0.84 (m, 2H), 0.63–0.58 (m, 2H); ^{13}C NMR (126 MHz, $CDCl_3$) δ 169.2, 138.2, 134.4, 133.0, 124.6, 23.0, 21.2, 6.8; HRMS (ESI) *m/z* calcd for $C_{12}H_{15}NNaO$ $[M+Na]^+$: 212.1046, found 212.1045.

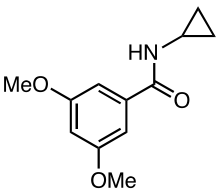

1N

***N*-Cyclopropyl-3,5-dimethoxybenzamide (1N):** The synthetic procedure of **1N** is the same as that of **1B**. 21.3 mmol scale. Purification by MPLC (hexane/ethyl acetate = 3:1 to 1:1); the obtained solid was crystallized from hexane and ethyl acetate to afford **1N** (932 mg, 21%) as a white solid. ^{1}H NMR (600 MHz, $CDCl_3$) δ 6.85 (d, J = 1.8 Hz, 2H), 6.56 (dd, J = 1.8 Hz, 1H), 6.17 (brs, 1H), 3.82 (s, 6H), 2.92–2.86 (m, 1H), 0.90–0.83 (m, 2H), 0.64–0.57 (m, 2H); ^{13}C NMR (151 MHz, $CDCl_3$) δ 168.7, 160.8, 136.6, 104.8, 103.5, 55.5, 23.1, 6.7; HRMS (ESI) *m/z* calcd for $C_{12}H_{15}NNaO_3$ $[M+Na]^+$: 244.0944, found 244.0942.

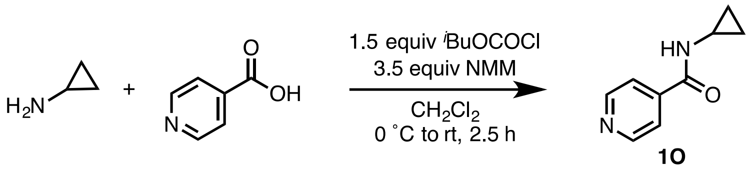

1O

***N*-Cyclopropylisonicotinamide (1O):** To a mixture of isonicotinic acid (739 mg, 6.0 mmol) and 4-methylmorpholine (NMM: 1.3 mL, 12.0 mmol) in dichloromethane (15 mL) was slowly added isobutyl chloroformate (1.2 mL, 9.0 mmol) at 0 °C, and this mixture was stirred at the same temperature for 30 min. Cyclopropylamine (0.63 mL, 9.0 mmol) in dichloromethane (10 mL) and 4-methylmorpholine (NMM: 1.0 mL, 9.0 mmol) was slowly added at 0 ˚C. The reaction mixture was stirred at room temperature for 2 h. The reaction was quenched by adding saturated aqueous $NaHCO_3$, which was then extracted with dichloromethane. The combined organic layers were washed with brine, dried over Na_2SO_4, filtered and concentrated *in vacuo*. The residue was purified by Isolera® (hexane/ethyl acetate = 1:1 to 0:1). The obtained solid was washed by hexane to give **1O** (772 mg, 79%) as a white solid. ^{1}H NMR (600 MHz, $CDCl_3$) δ 8.73 (dd, J = 4.2, 1.8 Hz, 2H), 7.57 (dd, J = 4.2, 1.8 Hz, 2H), 6.30 (brs, 1H), 2.95–2.90 (m, 1H), 0.95–0.87 (m, 2H), 0.68–0.62 (m, 2H); ^{13}C NMR (126 MHz, $CDCl_3$) δ 167.0, 150.4, 141.4, 120.8, 23.2, 6.6; HRMS (ESI) *m/z* calcd for $C_9H_{10}N_2NaO$ $[M+Na]^+$: 185.0685, found 185.0687.

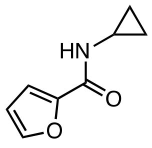

1P

***N*-Cyclopropylfuran-2-carboxamide (1P):** The synthetic procedure of **1P** is the same as that of **1H**. 20.0 mmol scale. Purification by MPLC (hexane/ethyl acetate = 3:1 to 1:1); the obtained solid was crystallized from hexane and ethyl acetate to afford **1P** (1.7 g, 57%) as a white solid. ^{1}H NMR (600 MHz, $CDCl_3$) δ 7.40 (s, 1H), 7.11 (d, *J* = 3.6 Hz, 1H), 6.49 (dd, *J* = 3.6, 1.2 Hz, 1H), 6.41 (brs, 1H), 2.89–2.84 (m, 1H), 0.89–0.82 (m, 2H), 0.66–0.59 (m, 2H); ^{13}C NMR (151 MHz, $CDCl_3$) δ 159.6, 148.0, 143.7, 114.0, 112.1, 22.2, 6.7; HRMS (ESI) *m*/*z* calcd for $C_8H_9NNaO_2$ $[M+Na]^+$: 174.0525, found 174.0521.

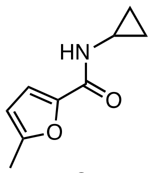

1Q

***N*-Cyclopropyl-5-methylfuran-2-carboxamide (1Q):** The synthetic procedure of **1Q** is the same as that of **1E**. 5.0 mmol scale. Purification by Isolera® (hexane/ethyl acetate = 1:1 to 0:1); the obtained solid was crystallized from hexane and ethyl acetate to afford **1Q** (538 mg, 65%) as a white solid. ^{1}H NMR (600 MHz, $CDCl_3$) δ 7.00 (d, *J* = 3.6 Hz, 1H), 6.33 (brs, 1H), 6.08 (d, *J* = 3.6 Hz, 1H), 2.88–2.83 (m, 1H), 2.32 (s, 3H), 0.87–0.81 (m, 2H), 0.64–0.59 (m, 2H); ^{13}C NMR (126 MHz, $CDCl_3$) δ 159.7, 154.2, 146.4, 115.3, 108.5, 22.2, 13.8, 6.7; HRMS (ESI) *m*/*z* calcd for $C_9H_{11}NNaO_2$ $[M+Na]^+$: 188.0682, found 188.0682.

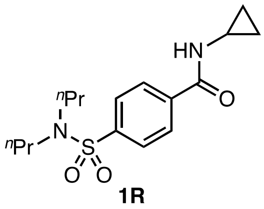

1R

***N*-Cyclopropyl-4-(*N*,*N*-dipropylsulfamoyl)benzamide (1R):** The synthetic procedure of **1R** is the same as that of **1E**. 2.5 mmol scale. Purification by Isolera® (hexane/ethyl acetate = 1:1 to 0:1); the obtained solid was crystallized from hexane to afford **1R** (561 mg, 69%) as a white solid. ^{1}H NMR (600 MHz, $CDCl_3$) δ 7.83 (s, 4H), 6.34 (brs, 1H), 3.08 (t, *J* = 7.8 Hz, 4H), 2.95–2.90 (m, 1H), 1.54 (sext, *J* = 7.8 Hz, 4H), 0.93–0.88 (m, 2H), 0.87 (t, *J* = 7.8 Hz, 6H), 0.68–0.63 (m, 2H); ^{13}C NMR (151 MHz, $CDCl_3$) δ 167.7, 142.5, 138.1, 127.7, 127.0, 49.9, 23.3, 21.8, 11.1, 6.5; HRMS (ESI) *m*/*z* calcd for $C_{16}H_{24}N_2NaO_3S$ $[M+Na]^+$: 347.1400, found 347.1396.

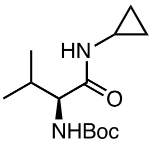

1S

***tert*-Butyl (*S*)-(1-(cyclopropylamino)-3-methyl-1-oxobutan-2-yl)carbamate (1S):** The synthetic procedure of **1S** is the same as that of **1E**. 4.0 mmol scale. Purification by Isolera® (hexane/ethyl acetate = 2:1 to 0:1); the obtained solid was crystallized from hexane and ethyl acetate to afford **1S** (715 mg, 72%) as a white solid. ^{1}H NMR (600 MHz, 90 ˚C, DMSO-d_6) δ 7.62 (brs, 1H), 6.08 (brs, 1H), 3.72–3.68 (m, 1H), 2.67–2.61 (m, 1H), 1.93–1.86 (m, 1H), 1.40 (s, 9H), 0.85 (d, J = 6.8 Hz, 3H), 0.83 (d, J = 6.9 Hz, 3H), 0.65–0.59 (m, 2H), 0.46–0.37 (m, 2H); ^{13}C NMR (151 MHz, 90 ˚C, DMSO-d_6) δ 171.8, 154.8, 77.7, 59.4, 30.2, 27.7, 21.7, 18.6, 17.6, 5.1, 5.0; HRMS (ESI) *m*/*z* calcd for $C_{13}H_{24}N_2NaO_3$ [M+Na]$^+$: 279.1679, found 279.1675.

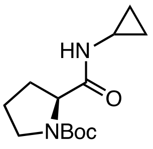

1T

***tert*-Butyl (*S*)-2-(cyclopropylcarbamoyl)pyrrolidine-1-carboxylate (1T):** The synthetic procedure of **1T** is the same as that of **1E**. 5.0 mmol scale. Purification by Isolera® (hexane/ethyl acetate = 2:1 to 0:1); the obtained solid was crystallized from hexane and ethyl acetate to afford **1T** (715 mg, 72%) as a white solid. ^{1}H NMR (500 MHz, 90 ˚C, DMSO-d_6) δ 7.53 (brs, 1H), 4.01–3.95 (m, 1H), 3.40–3.34 (m, 1H), 3.33–3.27 (m, 1H), 2.66–2.60 (m, 1H), 2.09–2.01 (m, 1H), 1.87–1.70 (m, 3H), 1.38 (s, 9H), 0.65–0.58 (m, 2H), 0.47–0.38 (m, 2H); ^{13}C NMR (126 MHz, 90 ˚C, DMSO-d_6) δ 172.8, 153.1, 78.0, 59.3, 46.1, 30.0, 27.7, 22.9, 21.8, 5.0; HRMS (ESI) *m*/*z* calcd for $C_{13}H_{22}N_2NaO_3$ [M+Na]$^+$: 277.1523, found 277.1518.

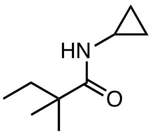

1U

***N*-Cyclopropyl-2,2-dimethylbutanamide (1U):** The synthetic procedure of **1U** is the same as that of **1B**. 9.5 mmol scale. Purification by MPLC (hexane/ethyl acetate = 3:2 to 0:1) to afford **1T** (1.0 g, 86%) as a yellow oil. ^{1}H NMR (500 MHz, $CDCl_3$) δ 5.68 (brs, 1H), 2.73–2.67 (m, 1H), 1.52 (q, J = 7.5 Hz, 2H), 1.12 (s, 6H), 0.82 (t, J = 7.5 Hz, 3H), 0.79–0.74 (m, 2H), 0.47–0.42 (m, 2H); ^{13}C NMR (126 MHz,

$CDCl_3$) δ 179.1, 42.1, 33.8, 24.8, 22.6, 9.1, 6.6; HRMS (ESI) *m*/*z* calcd for $C_9H_{17}NNaO$ $[M+Na]^+$: 178.1202, found 178.1204.

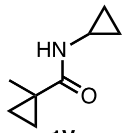

1V

***N*-Cyclopropyl-1-methylcyclopropane-1-carboxamide (1V):** The synthetic procedure of **1V** is the same as that of **1B**. 6.2 mmol scale. Purification by Isolera® (hexane/ethyl acetate = 2:1 to 0:1); the obtained solid was crystallized from hexane to afford **1V** (289 mg, 34%) as a white solid. ^1H NMR (600 MHz, $CDCl_3$) δ 5.83 (brs, 1H), 2.74–2.68 (m, 1H), 1.27 (s, 3H), 1.21–1.17 (m, 2H), 0.79–0.74 (m, 2H), 0.56–0.53 (m, 2H), 0.51–0.47 (m, 2H); ^{13}C NMR (151 MHz, $CDCl_3$) δ 176.2, 23.0, 19.7, 18.9, 16.0, 6.6; HRMS (ESI) *m*/*z* calcd for $C_8H_{13}NNaO$ $[M+Na]^+$: 162.0889, found 162.0886.

3. General Procedure for the Hydrosilylation of Aminocyclopropanes with $[Rh(cod)Cl]_2$/P(1-nap)$_3$ Catalyst

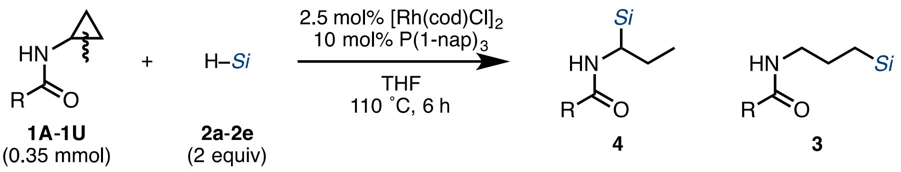

A 20 mL glass vessel tube equipped with a J. Young® O-ring tap containing a magnetic stirring bar was dried with a heat gun under reduced pressure and filled with nitrogen after cooling to room temperature. To this vessel was added aminocyclopropane (0.35 mmol), $[Rh(cod)Cl]_2$ (4.3 mg, 8.8 μmol, 2.5 mol%), and (P(1-nap)$_3$) (14.4 mg, 0.035 mmol, 10 mol%), after which it was introduced inside an argon atmosphere glovebox. In the glovebox, silane (0.70 mmol, 2 equiv) and THF (2.0 mL) were added to the vessel. After the vessel was sealed with the O-ring tap, the vessel was taken out of the glovebox. The mixture was heated at 110 ˚C for 6 h in an 8-well reaction block with stirring. After cooling the reaction mixture to room temperature, the mixture was concentrated *in vacuo*. The residue was purified by Isolera® to afford aminosilane **4**. The branch/linear (**4**/**3**) ratio was determined by ^{1}H NMR analysis of the crude product.

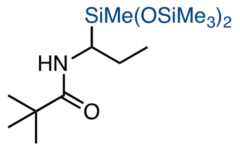

4Aa

***N*-(1-(1,1,1,3,5,5,5-Heptamethyltrisiloxan-3-yl)propyl)pivalamide (4Aa):** Purification by Isolera® (hexane/ethyl acetate = 1:0 to 5:1) afforded **4Aa** as a yellow oil (103.4 mg, 81%, branch/linear = 90:10). ^{1}H NMR (600 MHz, $CDCl_3$) δ 5.45 (d, *J* = 8.4 Hz, 1H), 3.32 (td, *J* = 9.6, 4.8 Hz, 1H), 1.68–1.60 (m, 1H), 1.39–1.30 (m, 1H), 1.20 (s, 9H), 0.90 (t, *J* = 7.8 Hz, 3H), 0.12 (s, 9H), 0.11 (s, 9H), 0.052 (s, 3H); ^{13}C NMR (151 MHz, $CDCl_3$) δ 177.9, 40.9, 38.7, 27.7, 23.6, 11.4, 1.81, 1.79, -1.6; HRMS (DART) *m*/*z* calcd for $C_{15}H_{38}NO_3Si_3$ $[M+H]^+$: 364.2154, found 364.2153.

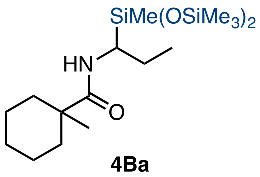

4Ba

***N*-(1-(1,1,1,3,5,5,5-Heptamethyltrisiloxan-3-yl)propyl)-1-methylcyclohexane-1-carboxamide (4Ba):** Purification by Isolera® (hexane/ethyl acetate = 1:0 to 5:1) afforded **4Ba** as a colorless oil (117.3 mg, 83%, branch/linear = 90:10). ^{1}H NMR (400 MHz, $CDCl_3$) δ 5.48 (d, *J* = 10.0 Hz, 1H), 3.36 (td, *J* = 9.6, 4.8 Hz,

1H), 1.97–1.87 (m, 2H), 1.71–1.24 (m, 10H), 1.14 (s, 3H), 0.91 (t, *J* = 7.6 Hz, 3H), 0.12 (s, 9H), 0.11 (s, 9H), 0.055 (s, 3H); ^{13}C NMR (151 MHz, $CDCl_3$) δ 177.0, 42.8, 40.7, 35.9, 35.8, 27.0, 25.9, 23.7, 23.0, 11.6, 1.82, 1.78, -1.5; HRMS (DART) *m*/*z* calcd for $C_{18}H_{42}NO_3Si_3$ $[M+H]^+$: 404.2467, found 404.2470.

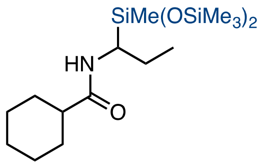

4Ca

***N*-(1-(λ^1-Silyl)propyl)cyclohexanecarboxamide (4Ca):** Purification by Isolera® (hexane/ethyl acetate = 1:0 to 5:1) afforded **4Ca** as a white solid (92.6 mg, 68%, branch/linear = 78:22). ^{1}H NMR (600 MHz, $CDCl_3$) δ 5.18 (d, *J* = 9.6 Hz, 1H), 3.32 (td, *J* = 9.6, 4.8 Hz, 1H), 2.10–2.03 (m, 1H), 1.91–1.83 (m, 2H), 1.82–1.75 (m, 2H), 1.71–1.58 (m, 2H), 1.48–1.17 (m, 6H), 0.90 (t, *J* = 7.2 Hz, 3H), 0.114 (s, 9H), 0.111 (s, 9H), 0.049 (s, 3H); ^{13}C NMR (151 MHz, $CDCl_3$) δ 175.6, 46.0, 40.9, 30.0, 29.9, 25.8, 23.6, 11.5, 1.80, 1.77, -1.7; HRMS (DART) *m*/*z* calcd for $C_{17}H_{40}NO_3Si_3$ $[M+H]^+$: 390.2311, found 390.2311.

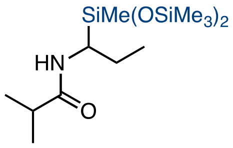

4Da

***N*-(1-(1,1,1,3,5,5,5-Heptamethyltrisiloxan-3-yl)propyl)isobutyramide (4Da):** Purification by Isolera® (hexane/ethyl acetate = 1:0 to 5:1) afforded **4Da** as a yellow solid (86.5 mg, 71%). The branch/linear ratio couldn't be determined because of the peak overlap. ^{1}H NMR (600 MHz, $CDCl_3$) δ 5.18 (d, *J* = 7.8 Hz, 1H), 3.32 (td, *J* = 9.6, 4.8 Hz, 1H), 2.38–2.31 (m, 1H), 1.68–1.59 (m, 1H), 1.39–1.31 (m, 1H), 1.17 (d, *J* = 7.2 Hz, 3H), 1.16 (d, *J* = 7.2 Hz, 3H), 0.91 (t, *J* = 7.2 Hz, 3H), 0.12 (s, 9H), 0.11 (s, 9H), 0.055 (s, 3H); ^{13}C NMR (151 MHz, $CDCl_3$) δ 176.4, 40.9, 36.0, 23.6, 19.83, 19.76, 11.5, 1.77, 1.75, -1.7; HRMS (DART) *m*/*z* calcd for $C_{14}H_{36}NO_3Si_3$ $[M+H]^+$: 350.1997, found 350.2003.

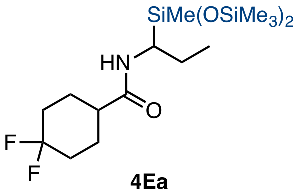

4Ea

4,4-Difluoro-*N*-(1-(1,1,1,3,5,5,5-heptamethyltrisiloxan-3-yl)propyl)cyclohexane-1-carboxamide (4Ea): The reaction was performed using $[Rh(cod)OMe]_2$ (2.1 mg, 4.4 μmol, 1.25 mol%) and P(1-nap)$_3$ (7.2 mg, 17.5 μmol, 5 mol%). Purification by Isolera® (hexane/ethyl acetate = 1:0 to 5:1) afforded **4Ea** as

a white solid (77.3 mg, 52%, branch/linear = 56:44). ^{1}H NMR (400 MHz, $CDCl_3$) δ 5.20 (d, J = 9.6 Hz, 1H), 3.33 (td, J = 9.6, 5.2 Hz, 1H), 2.24–2.11 (m, 3H), 1.99–1.55 (m, 7H), 1.42–1.25 (m, 1H), 0.90 (t, J = 7.2 Hz, 3H), 0.12 (s, 9H), 0.11 (s, 9H), 0.055 (s, 3H); ^{13}C NMR (101 MHz, $CDCl_3$) δ 173.5, 122.7 (t, J = 244.2 Hz), 43.3, 41.2, 32.9 (t, J = 24.8 Hz), 26.1 (t, J = 8.6 Hz), 23.6, 11.5, 1.82, 1.79, -1.6; HRMS (DART) *m*/*z* calcd for $C_{17}H_{38}F_2NO_3Si_3$ $[M+H]^+$: 426.2122, found 426.2124.

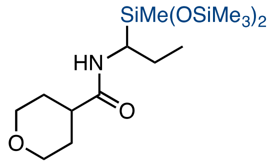

4Fa

***N*-(1-(1,1,1,3,5,5,5-Heptamethyltrisiloxan-3-yl)propyl)tetrahydro-2*H*-pyran-4-carboxamide (4Fa):** The reaction was performed using $[Rh(cod)OMe]_2$ (2.1 mg, 4.4 µmol, 1.25 mol%) and $P(1\text{-nap})_3$ (7.2 mg, 17.5 µmol, 5 mol%). Purification by Isolera® (hexane/ethyl acetate = 1:0 to 5:1) afforded **4Fa** as a white solid (76.0 mg, 55%). The branch/linear ratio couldn't be determined because of the peak overlap. ^{1}H NMR (600 MHz, $CDCl_3$) δ 5.21 (d, J = 9.6 Hz, 1H), 4.05–4.00 (m, 2H), 3.43 (td, J = 11.4, 3.0 Hz, 2H), 3.34 (td, J = 9.6, 4.8 Hz, 1H), 2.37–2.30 (m, 1H), 1.85–1.72 (m, 4H), 1.68–1.60 (m, 1H), 1.40–1.30 (m, 1H), 0.90 (t, J = 7.8 Hz, 3H), 0.12 (s, 9H), 0.11 (s, 9H), 0.056 (s, 3H); ^{13}C NMR (151 MHz, $CDCl_3$) δ 173.8, 67.4, 42.6, 41.1, 29.6, 23.6, 11.5, 1.84, 1.81, -1.6; HRMS (DART) *m*/*z* calcd for $C_{16}H_{38}NO_4Si_3$ $[M+H]^+$: 392.2103, found 392.2101.

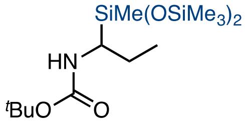

4Ga

***tert*-butyl (1-(1,1,1,3,5,5,5-Heptamethyltrisiloxan-3-yl)propyl)carbamate (4Ga):** Purification by Isolera® (hexane/ethyl acetate = 1:0 to 5:1) afforded **4Ga** as a colorless oil (92.1 mg, 69%). The branch/linear ratio couldn't be determined because of the peak overlap. ^{1}H NMR (600 MHz, 120 °C, DMSO-d_6) δ 5.53 (brs, 1H), 2.72 (td, J = 9.6, 4.8 Hz, 1H), 1.58–1.50 (m, 1H), 1.44–1.35 (m, 10H), 0.90 (t, J = 7.2 Hz, 3H), 0.13–0.10 (m, 18H), 0.072 (s, 3H); ^{13}C NMR (151 MHz, 120 °C, DMSO-d_6) δ 155.3, 76.8, 42.6, 27.7, 22.7, 10.8, 1.0, -2.5; HRMS (DART) *m*/*z* calcd for $C_{15}H_{38}NO_4Si_3$ $[M+H]^+$: 380.2103, found 380.2101.

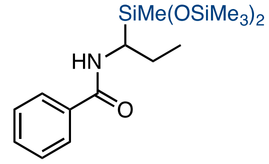

4Ha

***N*-(1-(1,1,1,3,5,5,5-Heptamethyltrisiloxan-3-yl)propyl)benzamide (4Ha):** Purification by Isolera® (hexane/ethyl acetate = 1:0 to 5:1) afforded **4Ha** as a white solid (73.8 mg, 55%). When the reaction was performed using $[Rh(cod)OMe]_2$ (2.1 mg, 4.4 μmol, 1.25 mol%) and $P(1\text{-nap})_3$ (7.2 mg, 17.5 μmol, 5 mol%), **4Ha** was obtained in 66% yield (88.9 mg, branch/linear = 78:22). ^{1}H NMR (600 MHz, $CDCl_3$) δ 7.75–7.72 (m, 2H), 7.50–7.46 (m, 1H), 7.45–7.41 (m, 2H), 5.90 (d, *J* = 9.0 Hz, 1H), 3.55 (td, *J* = 9.6, 4.8 Hz, 1H), 1.79–1.71 (m, 1H), 1.52–1.44 (m, 1H), 0.98 (t, *J* = 7.3 Hz, 3H), 0.13 (s, 9H), 0.12 (s, 3H), 0.11 (s, 9H); ^{13}C NMR (151 MHz, $CDCl_3$) δ 167.3, 135.5, 131.0, 128.5, 126.6, 42.0, 23.8, 11.7, 1.8, -1.5; HRMS (DART) *m*/*z* calcd for $C_{17}H_{34}NO_3Si_3$ $[M+H]^+$: 384.1841, found 384.1846.

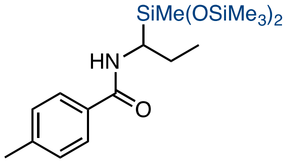

4Ia

***N*-(1-(1,1,1,3,5,5,5-Heptamethyltrisiloxan-3-yl)propyl)-4-methylbenzamide (4Ia):** The reaction was performed using $[Rh(cod)OMe]_2$ (2.1 mg, 4.4 μmol, 1.25 mol%) and $P(1\text{-nap})_3$ (7.2 mg, 17.5 μmol, 5 mol%). Purification by Isolera® (hexane/ethyl acetate = 1:0 to 5:1) afforded **4Ia** as a colorless oil (90.8 mg, 65%, branch/linear = 82:18). ^{1}H NMR (600 MHz, $CDCl_3$) δ 7.64 (d, *J* = 8.4 Hz, 2H), 7.23 (d, *J* = 8.4 Hz, 2H), 5.88 (d, *J* = 9.6 Hz, 1H), 3.54 (td, *J* = 9.6, 4.8 Hz, 1H), 2.39 (s, 3H), 1.78–1.69 (m, 1H), 1.52–1.42 (m, 1H), 0.97 (t, *J* = 7.2 Hz, 3H), 0.13 (s, 9H), 0.11 (s, 3H), 0.10 (s, 9H); ^{13}C NMR (151 MHz, $CDCl_3$) δ 167.3, 141.3, 132.6, 129.2, 126.6, 41.9, 23.8, 21.4, 11.7, 1.8, -1.5; HRMS (DART) *m*/*z* calcd for $C_{18}H_{36}NO_3Si_3$ $[M+H]^+$: 398.1997, found 398.1997.

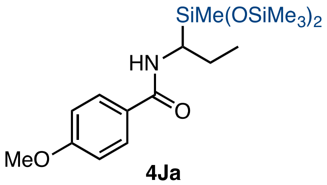

4Ja

***N*-(1-(1,1,1,3,5,5,5-Heptamethyltrisiloxan-3-yl)propyl)-4-methoxybenzamide (4Ja):** The reaction was performed using $[Rh(cod)OMe]_2$ (2.1 mg, 4.4 μmol, 1.25 mol%) and $P(1\text{-nap})_3$ (7.2 mg, 17.5 μmol, 5 mol%). Purification by Isolera® (hexane/ethyl acetate = 1:0 to 5:1) afforded **4Ja** as a colorless oil (92.8 mg, 64%, branch/linear = 82:18). ^{1}H NMR (600 MHz, $CDCl_3$) δ 7.71 (d, *J* = 9.0 Hz, 2H), 6.93 (d, *J* = 9.0

Hz, 2H), 5.84 (d, *J* = 10.2 Hz, 1H), 3.85 (s, 3H), 3.53 (td, *J* = 9.6, 4.8 Hz, 1H), 1.78–1.68 (m, 1H), 1.52–1.43 (m, 1H), 0.97 (t, *J* = 7.2 Hz, 3H), 0.13 (s, 9H), 0.110 (s, 3H), 0.105 (s, 9H); ^{13}C NMR (151 MHz, $CDCl_3$) δ 166.8, 161.9, 128.3, 127.7, 113.7, 55.4, 41.8, 23.9, 11.7, 1.8, -1.5; HRMS (DART) *m*/*z* calcd for $C_{18}H_{36}NO_4Si_3$ [M+H]$^+$: 414.1947, found 414.1950.

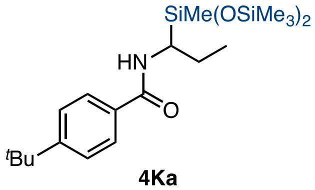

4Ka

4-(*tert*-Butyl)-*N*-(1-(1,1,1,3,5,5,5-heptamethyltrisiloxan-3-yl)propyl)benzamide (4Ka): Purification by Isolera® (hexane/ethyl acetate = 1:0 to 5:1) afforded **4Ka** as a colorless oil (99.0 mg, 65%, branch/linear = 82:18). When the reaction was performed using $[Rh(cod)OMe]_2$ (2.1 mg, 4.4 µmol, 1.25 mol%) and $P(1\text{-}nap)_3$ (7.2 mg, 17.5 µmol, 5 mol%), **4Ha** was obtained in 79% (122.0 mg, branch/linear = 79:21). ^{1}H NMR (600 MHz, $CDCl_3$) δ 7.68 (d, *J* = 8.6 Hz, 2H), 7.45 (d, *J* = 8.6 Hz, 2H), 5.89 (d, *J* = 9.0 Hz, 1H), 3.55 (td, *J* = 9.6, 4.8 Hz, 1H), 1.78–1.70 (m, 1H), 1.51–1.43 (m, 1H), 1.34 (s, 9H), 0.97 (t, *J* = 7.4 Hz, 3H), 0.14 (s, 9H), 0.12 (s, 9H), 0.11 (s, 3H); ^{13}C NMR (151 MHz, $CDCl_3$) δ 167.2, 154.4, 132.5, 126.4, 125.4, 41.8, 34.8, 31.1, 23.8, 11.7, 1.8, -1.6; HRMS (DART) *m*/*z* calcd for $C_{21}H_{42}NO_3Si_3$ [M+H]$^+$: 440.2467, found 440.2466.

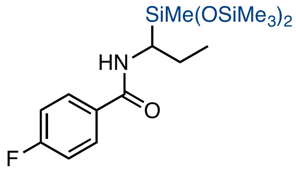

4La

4-Fluoro-*N*-(1-(1,1,1,3,5,5,5-heptamethyltrisiloxan-3-yl)propyl)benzamide (4La): Purification by Isolera® (hexane/ethyl acetate = 1:0 to 5:1) afforded **4La** as a yellow oil (84.5 mg, 60%, branch/linear = 72:28). ^{1}H NMR (600 MHz, $CDCl_3$) δ 7.76–7.72 (m, 2H), 7.13–7.08 (m, 2H), 5.83 (d, *J* = 9.6 Hz, 1H), 3.53 (td, *J* = 9.0, 4.8 Hz, 1H), 1.78–1.70 (m, 1H), 1.52–1.43 (m, 1H), 0.98 (t, *J* = 7.8 Hz, 3H), 0.15–0.08 (m, 21H); ^{13}C NMR (151 MHz, $CDCl_3$) δ 166.3, 164.5 (d, J_{FC} = 248.6 Hz), 131.6 (d, J_{FC} = 2.9 Hz), 128.8 (d, J_{FC} = 8.7 Hz), 115.5 (d, J_{FC} = 21.5 Hz), 42.1, 23.8, 11.7, 1.8, -1.5; HRMS (DART) *m*/*z* calcd for $C_{17}H_{33}FNO_3Si_3$ [M+H]$^+$: 402.1747, found 402.1745.

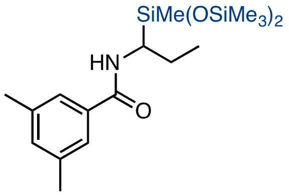

4Ma

***N*-(1-(1,1,1,3,5,5,5-Heptamethyltrisiloxan-3-yl)propyl)-3,5-dimethylbenzamide (4Ma):** Purification by Isolera® (hexane/ethyl acetate = 1:0 to 5:1) afforded **4Ma** as a white solid (113.6 mg, 79%). The branch/linear ratio couldn't be determined because of the peak overlap. ^{1}H NMR (600 MHz, $CDCl_3$) δ 7.34 (s, 2H), 7.11 (s, 1H), 5.89 (d, *J* = 10.2 Hz, 1H), 3.53 (td, *J* = 9.6, 4.8 Hz, 1H), 2.35 (s, 6H), 1.78–1.70 (m, 1H), 1.51–1.43 (m, 1H), 0.97 (t, *J* = 7.8 Hz, 3H), 0.14 (s, 9H), 0.12 (s, 9H), 0.11 (s, 3H); ^{13}C NMR (151 MHz, $CDCl_3$) δ 167.7, 138.2, 135.4, 132.6, 124.4, 41.8, 23.8, 21.2, 11.7, 1.8, -1.5; HRMS (DART) *m*/*z* calcd for $C_{19}H_{38}NO_3Si_3$ $[M+H]^+$: 412.2154, found 412.2152.

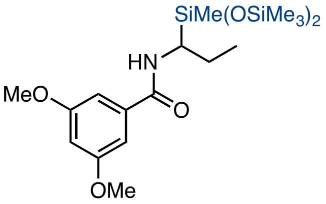

4Na

***N*-(1-(1,1,1,3,5,5,5-Heptamethyltrisiloxan-3-yl)propyl)-3,5-dimethoxybenzamide (4Na):** Purification by Isolera® (hexane/ethyl acetate = 1:0 to 5:1) afforded **4Na** as a white solid (108.0 mg, 70%, branch/linear = 83:17). ^{1}H NMR (600 MHz, $CDCl_3$) δ 6.87 (d, *J* = 2.4 Hz, 2H), 6.57–6.56 (m, 1H), 5.86 (d, *J* = 9.6 Hz, 1H), 3.83 (s, 6H), 3.52 (td, *J* = 9.6, 4.8 Hz, 1H), 1.78–1.70 (m, 1H), 1.51–1.42 (m, 1H), 0.98 (t, *J* = 7.2 Hz, 3H), 0.14–0.10 (m, 21H); ^{13}C NMR (151 MHz, $CDCl_3$) δ 167.2, 160.9, 137.7, 104.6, 103.2, 55.5, 42.1, 23.8, 11.7, 1.8, -1.6; HRMS (DART) *m*/*z* calcd for $C_{19}H_{38}NO_5Si_3$ $[M+H]^+$: 444.2052, found 444.2052.

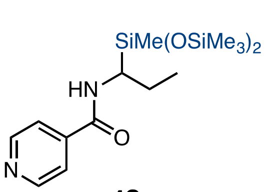

4Oa

***N*-(1-(1,1,1,3,5,5,5-Heptamethyltrisiloxan-3-yl)propyl)isonicotinamide (4Oa):** The reaction was stirred for 18 h. Purification by Isolera® (hexane/ethyl acetate = 1:0 to 5:1) afforded **4Oa** as a yellow oil (72.9 mg, 51%, branch/linear = 80:20). ^{1}H NMR (600 MHz, $CDCl_3$) δ 8.74 (dd, *J* = 4.2, 1.8 Hz, 2H), 7.57 (dd, *J* = 4.2, 1.8 Hz, 2H), 5.95 (d, *J* = 9.6 Hz, 1H), 3.54 (td, *J* = 9.6, 4.8 Hz, 1H), 1.80–1.71 (m, 1H),

1.53–1.44 (m, 1H), 0.98 (t, *J* = 7.2 Hz, 3H), 0.134 (s, 9H), 0.126 (s, 3H), 0.10 (s, 9H); ^{13}C NMR (151 MHz, $CDCl_3$) δ 165.3, 150.6, 142.4, 120.6, 42.5, 23.7, 11.7, 1.8, -1.5; HRMS (DART) *m*/*z* calcd for $C_{16}H_{33}N_2O_3Si_3$ $[M+H]^+$: 385.1793, found 385.1795.

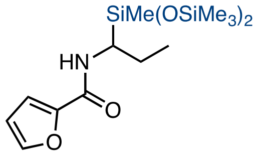

4Pa

***N*-(1-(1,1,1,3,5,5,5-Heptamethyltrisiloxan-3-yl)propyl)furan-2-carboxamide (4Pa):** The reaction was stirred for 18 h. Purification by Isolera® (hexane/ethyl acetate = 1:0 to 5:1) afforded **4Pa** as a colorless oil (86.2 mg, 66%). The branch/linear ratio couldn't be determined because of the peak overlap. ^{1}H NMR (600 MHz, $CDCl_3$) δ 7.42 (s, 1H), 7.07 (d, *J* = 3.6 Hz, 1H), 6.49 (dd, *J* = 3.6, 1.8 Hz, 1H), 6.17 (d, *J* = 10.2 Hz, 1H), 3.48 (td, *J* = 9.6, 4.8 Hz, 1H), 1.76–1.68 (m, 1H), 1.51–1.42 (m, 1H), 0.97 (t, *J* = 7.2 Hz, 3H), 0.13 (s, 9H), 0.11 (s, 9H), 0.10 (s, 3H); ^{13}C NMR (151 MHz, $CDCl_3$) δ 158.4, 148.5, 143.4, 113.5, 112.0, 41.1, 23.8, 11.7, 1.7, -1.6; HRMS (DART) *m*/*z* calcd for $C_{15}H_{32}NO_4Si_3$ $[M+H]^+$: 374.1634, found 374.1635.

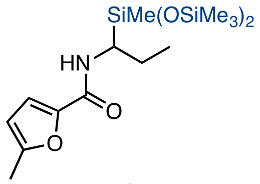

4Qa

***N*-(1-(1,1,1,3,5,5,5-Heptamethyltrisiloxan-3-yl)propyl)-5-methylfuran-2-carboxamide (4Qa):** Purification by Isolera® (hexane/ethyl acetate = 1:0 to 5:1) afforded **4Qa** as a colorless oil (96.5 mg, 71%, branch/linear = 81:19). ^{1}H NMR (500 MHz, $CDCl_3$) δ 6.96 (d, *J* = 3.0 Hz, 1H), 6.12 (d, *J* = 10.0 Hz, 1H), 6.08 (d, *J* = 3.0 Hz, 1H), 3.47 (td, *J* = 9.5, 5.0 Hz, 1H), 2.33 (s, 3H), 1.77–1.67 (m, 1H), 1.51–1.41 (m, 1H), 0.97 (t, *J* = 7.5 Hz, 3H), 0.13 (s, 9H), 0.12 (s, 9H), 0.10 (s, 3H); ^{13}C NMR (151 MHz, $CDCl_3$) δ 158.6, 153.9, 146.9, 114.6, 108.3, 40.9, 23.8, 13.7, 11.7, 1.7, -1.6; HRMS (DART) *m*/*z* calcd for $C_{16}H_{34}NO_4Si_3$ $[M+H]^+$: 388.1790, found 388.1790.

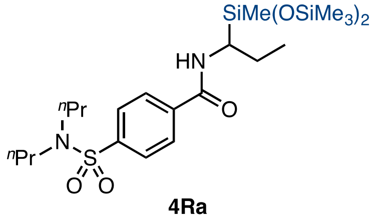

4Ra

4-(*N*,*N*-Dipropylsulfamoyl)-*N*-(1-(1,1,1,3,5,5,5-heptamethyltrisiloxan-3-yl)propyl)benzamide (4Ra): Purification by Isolera® (hexane/ethyl acetate = 1:0 to 5:1) afforded **4Ra** as a colorless oil (94.8 mg, 50%, branch/linear = 65:35). ^{1}H NMR (500 MHz, $CDCl_3$) δ 7.89–7.81 (m, 4H), 5.91 (d, *J* = 10.0 Hz, 1H), 3.54 (td, *J* = 9.5, 5.0 Hz, 1H), 3.10 (t, *J* = 7.0 Hz, 4H), 1.80–1.71 (m, 1H), 1.60–1.45 (m, 5H), 0.99 (t, *J* = 7.5 Hz, 3H), 0.87 (t, *J* = 7.0 Hz, 6H), 0.14–0.080 (m, 21H); ^{13}C NMR (151 MHz, $CDCl_3$) δ 166.0, 142.6, 138.9, 127.3, 49.9, 42.5, 23.7, 21.9, 11.8, 11.1, 1.8, -1.5; HRMS (DART) *m*/*z* calcd for $C_{23}H_{47}N_2O_5SSi_3$ $[M+H]^+$: 547.2508, found 547.2507.

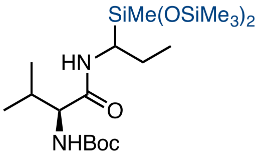

4Sa

***tert*-Butyl ((2*S*)-1-((1-(1,1,1,3,5,5,5-heptamethyltrisiloxan-3-yl)propyl)amino)-3-methyl-1-oxobutan-2-yl)carbamate (4Sa):** The reaction was performed using $[Rh(cod)OMe]_2$ (2.1 mg, 4.4 μmol, 1.25 mol%) and P(1-nap)$_3$ (7.2 mg, 17.5 μmol, 5 mol%) for 18 h. Purification by Isolera® (hexane/ethyl acetate = 1:0 to 5:1) afforded **4Sa** as a white solid (87.0 mg, 52%, mixture of diastereomers). The branch/linear ratio couldn't be determined because of the peak overlap. ^{1}H NMR (600 MHz, 60 ˚C, $CDCl_3$) δ 5.64–5.53 (m, 1H), 4.95 (brs, 1H), 3.86–3.80 (m, 1H), 3.35–3.28 (m, 1H), 2.20–2.11 (m, 1H), 1.69–1.60 (m, 1H), 1.44–1.43 (m, 9H), 1.41–1.32 (m, 1H), 0.96 (t, *J* = 7.2 Hz, 3H), 0.94–0.88 (m, 6H), 0.13–0.11 (m, 18H), 0.080–0.040 (m, 3H); ^{13}C NMR (101 MHz, 60 ˚C, $CDCl_3$) δ 171.3, 171.1, 155.8, 155.6, 79.6, 60.5, 41.5, 30.7, 30.6, 28.2, 23.51, 23.47, 19.34, 19.28, 17.9, 17.7, 11.7, 11.5, 1.7, -1.6, -1.8; HRMS (DART) *m*/*z* calcd for $C_{20}H_{47}N_2O_5Si_3$ $[M+H]^+$: 479.2787, found 479.2786.

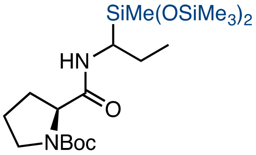

4Ta

***tert*-Butyl (2*S*)-2-((1-(1,1,1,3,5,5,5-heptamethyltrisiloxan-3-yl)propyl)carbamoyl)pyrrolidine-1-carboxylate (4Ta):** Purification by Isolera® (hexane/ethyl acetate = 1:0 to 5:1) afforded **4Ta** as a colorless oil (43.0 mg, 26%) and Diastereomer-**4Ta** (65.6 mg, 39%). The branch/linear ratio couldn't be determined because of the peak overlap. ^{1}H NMR (600 MHz, 120 ˚C, DMSO-d_6) δ 6.72 (d, *J* = 8.4 Hz, 1H), 4.21 (dd, *J* = 8.4, 3.0 Hz, 1H), 3.38–3.32 (m, 2H), 3.09 (td, *J* = 9.0, 4.8 Hz, 1H), 2.07–1.99 (m, 1H), 1.97–1.90 (m, 1H), 1.88–1.76 (m, 2H), 1.63–1.55 (m, 1H), 1.44–1.36 (m, 10H), 0.89 (t, *J* = 7.2 Hz, 3H), 0.124 (s, 9H), 0.116 (s, 9H), 0.056 (s, 3H); ^{13}C NMR (151 MHz, 120 ˚C, DMSO-d_6) δ 170.8, 153.5, 78.2,

59.2, 46.0, 41.1, 29.2, 27.6, 22.8, 22.4, 10.9, 1.0, -2.4; HRMS (DART) *m*/*z* calcd for $C_{20}H_{45}N_2O_5Si_3$ $[M+H]^+$: 477.2631, found 477.2635. Diastereomer-**4Ta** ^1H NMR (600 MHz, 120 ˚C, DMSO-d_6) δ 6.48 (d, *J* = 7.8 Hz, 1H), 4.18 (dd, *J* = 8.4, 3.0 Hz, 1H), 3.41–3.32 (m, 2H), 3.08 (td, *J* = 9.0, 4.8 Hz, 1H), 2.10–2.04 (m, 1H), 1.96–1.91 (m, 1H), 1.84–1.77 (m, 2H), 1.63–1.55 (m, 1H), 1.43–1.36 (m, 10H), 0.89 (t, *J* = 7.2 Hz, 3H), 0.122 (s, 9H), 0.117 (s, 9H), 0.059 (s, 3H); ^{13}C NMR (151 MHz, 120 ˚C, DMSO-d_6) δ 170.8, 153.5, 78.4, 59.7, 46.1, 41.0, 29.4, 27.6, 22.8, 22.4, 10.9, 1.0, -2.3; HRMS (DART) *m*/*z* calcd for $C_{20}H_{45}N_2O_5Si_3$ $[M+H]^+$: 477.2631, found 477.2639.

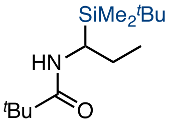

4Ab

***N*-(1-(*tert*-Butyldimethylsilyl)propyl)pivalamide (4Ab):** Purification by Isolera® (hexane/ethyl acetate = 1:0 to 5:1) afforded **4Ab** as a yellow solid (41.1 mg, 46%, branch/linear = 84:16). ^1H NMR (600 MHz, $CDCl_3$) δ 5.32 (d, *J* = 8.4 Hz, 1H), 3.65 (td, *J* = 10.2, 3.6 Hz, 1H), 1.71–1.63 (m, 1H), 1.41–1.31 (m, 1H), 1.21 (s, 9H), 0.94–0.88 (m, 12 H), 0.013 (s, 6H); ^{13}C NMR (151 MHz, $CDCl_3$) δ 177.3, 38.8, 38.4, 27.7, 26.8, 25.3, 16.9, 11.7, -7.2, -7.8; HRMS (DART) *m*/*z* calcd for $C_{14}H_{32}NOSi$ $[M+H]^+$: 258.2248, found 258.2249.

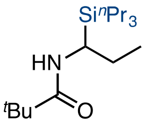

4Ac

***N*-(1-(Tripropylsilyl)propyl)pivalamide (4Ac):** Purification by Isolera® (hexane/ethyl acetate = 1:0 to 5:1) afforded **4Ac** as a white solid (100.8 mg, 96%, branch/linear = 94:6). ^1H NMR (600 MHz, $CDCl_3$) δ 5.27 (d, *J* = 10.2 Hz, 1H), 3.58 (td, *J* = 10.8, 3.6 Hz, 1H), 1.65–1.58 (m, 1H), 1.43–1.30 (m, 7H), 1.20 (s, 9H), 0.96 (t, *J* = 7.2 Hz, 9H), 0.90 (t, *J* = 7.2 Hz, 3H), 0.62–0.52 (m, 6H); ^{13}C NMR (151 MHz, $CDCl_3$) δ 177.5, 39.3, 38.8, 27.8, 24.9, 18.7, 17.5, 14.0, 12.0; HRMS (DART) *m*/*z* calcd for $C_{17}H_{38}NOSi$ $[M+H]^+$: 300.2717, found 300.2719.

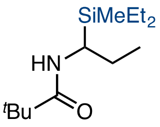

4Ad

***N*-(1-(Diethyl(methyl)silyl)propyl)pivalamide (4Ad):** Purification by Isolera® (hexane/ethyl acetate = 1:0 to 5:1) afforded **4Ad** as a white solid (86.0 mg, 99%, branch/linear = >95:5). ^{1}H NMR (600 MHz, $CDCl_3$) δ 5.27 (d, *J* = 8.4 Hz, 1H), 3.54 (td, *J* = 10.2, 3.6 Hz, 1H), 1.66–1.58 (m, 1H), 1.40–1.30 (m, 1H), 1.20 (s, 9H), 0.98–0.94 (m, 6H), 0.91 (t, *J* = 7.2 Hz, 3H), 0.63–0.50 (m, 4H), -0.023 (s, 3H); ^{13}C NMR (151 MHz, $CDCl_3$) δ 177.6, 39.6, 38.8, 27.8, 24.5, 11.9, 7.42, 7.39, 3.68, 3.66, -7.8; HRMS (DART) *m*/*z* calcd for $C_{13}H_{30}NOSi$ $[M+H]^+$: 244.2091, found 244.2091.

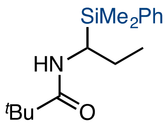

4Ae

***N*-(1-(Dimethyl(phenyl)silyl)propyl)pivalamide (4Ae):** The reaction was performed using $[Rh(cod)Cl]_2$ (8.6 mg, 17.5 µmol, 5.0 mol%), and $P(1\text{-nap})_3$ (28.8 mg, 0.070 mmol, 20 mol%) for 12 h. Purification by Isolera® (hexane/ethyl acetate = 1:0 to 5:1) afforded **4Ae** as a white solid (59.7 mg, 61%, branch/linear = 87:13). ^{1}H NMR (600 MHz, $CDCl_3$) δ 7.51 (dd, *J* = 7.2, 2.4 Hz, 2H), 7.40–7.35 (m, 3H), 5.14 (d, *J* = 9.0 Hz, 1H), 3.63 (td, *J* = 10.8, 4.2 Hz, 1H), 1.66–1.59 (m, 1H), 1.33–1.24 (m, 1H), 1.13 (s, 9H), 0.86 (t, *J* = 7.2 Hz, 3H), 0.35 (s, 3H), 0.33 (s, 3H); ^{13}C NMR (151 MHz, $CDCl_3$) δ 177.8, 136.3, 134.0, 129.4, 127.9, 40.7, 38.8, 27.7, 24.3, 11.9, -4.7, -5.0; HRMS (DART) *m*/*z* calcd for $C_{16}H_{28}NOSi$ $[M+H]^+$: 278.1935, found 278.1934.

4. General Procedure for the Hydrosilylation of Aminocyclopropanes with $[Rh(cod)Cl]_2/PCy_3{\cdot}HBF_4$ Catalyst

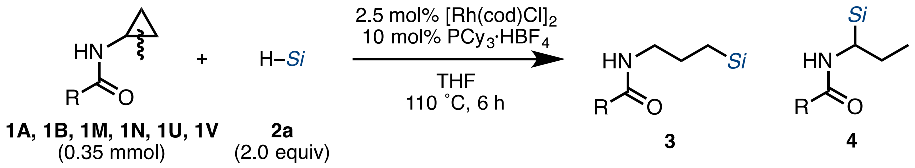

A 20 mL glass vessel tube equipped with a J. Young® O-ring tap containing a magnetic stirring bar was dried with a heatgun under reduced pressure and filled with nitrogen after cooling to room temperature. To this vessel was added aminocyclopropane (0.35 mmol), $[Rh(cod)Cl]_2$ (4.3 mg, 8.8 μmol, 2.5 mol%), and $PCy_3{\cdot}HBF_4$ (12.9 mg, 0.035 mmol, 10 mol%), after which it was introduced inside an argon atmosphere glovebox. In the glovebox, 1,1,1,3,5,5,5-heptamethyltrisiloxane (**2a**: 191 μL, 0.70 mmol, 2.0 equiv) and THF (2.0 mL) were added to the vessel. After the vessel was sealed with the O-ring tap, the vessel was taken out of the glovebox. The mixture was heated at 110 °C for 6 h in an 8-well reaction block with stirring. After cooling the reaction mixture to room temperature, the mixture was concentrated *in vacuo*. The residue was purified by Isolera® to afford aminosilane **3**. The linear/branch (**3**/**4**) ratio was determined by ^{1}H NMR analysis of the crude product.

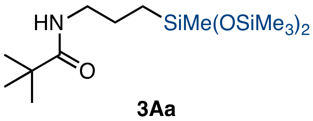

3Aa

***N*-(3-(1,1,1,3,5,5,5-Heptamethyltrisiloxan-3-yl)propyl)pivalamide (3Aa):** Purification by Isolera® (hexane/ethyl acetate = 1:0 to 5:1) afforded **3Aa** as a colorless oil (71.1 mg, 56%, linear/branch = 99:1). ^{1}H NMR (600 MHz, $CDCl_3$) δ 5.66 (brs, 1H), 3.22 (q, J = 6.6 Hz, 2H), 1.53–1.47 (m, 2H), 1.20 (s, 9H), 0.47–0.43 (m, 2H), 0.089 (s, 18H), 0.018 (s, 3H); ^{13}C NMR (151 MHz, $CDCl_3$) δ 178.2, 42.1, 38.6, 27.6, 23.3, 14.7, 1.8, -0.37; HRMS (ESI) m/z calcd for $C_{15}H_{37}NNaO_3Si_3$ $[M+Na]^+$: 386.1973, found 386.1972.

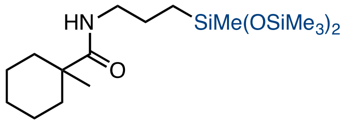

3Ba

***N*-(3-(1,1,1,3,5,5,5-Heptamethyltrisiloxan-3-yl)propyl)-1-methylcyclohexane-1-carboxamide (3Ba):** The reaction was stirred for 12 h. Purification by Isolera® (hexane/ethyl acetate = 1:0 to 5:1) afforded **3Ba** as a brown oil (83.3 mg, 59%, linear/branch = 99:1). ^{1}H NMR (600 MHz, $CDCl_3$) δ 5.66 (br, 1H), 3.24 (q, J = 6.6 Hz, 2H), 1.93–1.87 (m, 2H), 1.58–1.39 (m, 7H), 1.37–1.30 (m, 3H), 1.14 (s, 3H), 0.47–0.43 (m, 2H), 0.089 (s, 18H), 0.017 (s, 3H); ^{13}C NMR (151 MHz, $CDCl_3$) δ 177.4, 42.6, 42.0, 35.7, 26.5, 25.8,

23.5, 22.9, 14.8, 1.8, -0.36; HRMS (DART) *m*/*z* calcd for $C_{18}H_{42}NO_3Si_3$ $[M+H]^+$: 404.2467, found 404.2468.

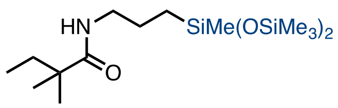

3Ua

***N*-(3-(1,1,1,3,5,5,5-Heptamethyltrisiloxan-3-yl)propyl)-2,2-dimethylbutanamide (3Ua):** The reaction was stirred for 12 h. Purification by Isolera® (hexane/ethyl acetate = 1:0 to 5:1) afforded **3Ua** as a colorless oil (67.2 mg, 51%, linear/branch = 99:1). ^{1}H NMR (600 MHz, $CDCl_3$) δ 5.62 (brs, 1H), 3.23 (q, *J* = 7.2 Hz, 2H), 1.54 (q, *J* = 7.2 Hz, 2H), 1.52–1.46 (m, 2H), 1.15 (s, 6H), 0.84 (t, *J* = 7.2 Hz, 3H), 0.47–0.42 (m, 2H), 0.088 (s, 18H), 0.016 (s, 3H); ^{13}C NMR (151 MHz, $CDCl_3$) δ 177.4, 42.3, 42.1, 33.9, 25.0, 23.5, 14.8, 9.2, 1.8, -0.37; HRMS (DART) *m*/*z* calcd for $C_{16}H_{40}NO_3Si_3$ $[M+H]^+$: 378.2311, found 378.2310.

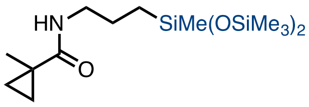

3Va

***N*-(3-(1,1,1,3,5,5,5-Heptamethyltrisiloxan-3-yl)propyl)-1-methylcyclopropane-1-carboxamide (3Va):** Purification by Isolera® (hexane/ethyl acetate = 1:0 to 5:1) afforded **3Va** as a brown oil (49.2 mg, 39%, linear/branch = 99:1). ^{1}H NMR (600 MHz, $CDCl_3$) δ 5.77 (brs, 1H), 3.24 (q, *J* = 6.6 Hz, 2H), 1.56–1.49 (m, 2H), 1.32 (s, 3H), 1.19 (dd, *J* = 6.0, 3.6 Hz, 2H), 0.55 (dd, *J* = 6.0, 3.6 Hz, 2H), 0.48–0.44 (m, 2H), 0.094 (s, 18H), 0.023 (s, 3H); ^{13}C NMR (151 MHz, $CDCl_3$) δ 174.7, 42.5, 23.4, 19.7, 18.9, 15.8, 14.8, 1.8, -0.37; HRMS (DART) *m*/*z* calcd for $C_{15}H_{36}NO_3Si_3$ $[M+H]^+$: 362.1997, found 362.1996.

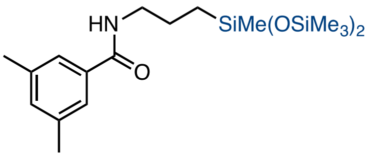

3Ma

***N*-(3-(1,1,1,3,5,5,5-Heptamethyltrisiloxan-3-yl)propyl)-3,5-dimethylbenzamide (3Ma):** The reaction was stirred for 25 h. Purification by Isolera® (hexane/ethyl acetate = 1:0 to 5:1) afforded **3Ma** as a brown oil (59.0 mg, 41%, linear/branch = 99:1). ^{1}H NMR (600 MHz, $CDCl_3$) δ 7.36 (s, 2H), 7.11 (s, 1H), 6.17 (brs, 1H), 3.42 (q, *J* = 6.6 Hz, 2H), 2.35 (s, 6H), 1.66–1.59 (m, 2H), 0.56–0.51 (m, 2H), 0.096 (s, 18H), 0.034 (s, 3H); ^{13}C NMR (151 MHz, $CDCl_3$) δ 167.8, 138.2, 135.0, 132.8, 124.6, 42.6, 23.4, 21.2, 14.9, 1.8, -0.33; HRMS (DART) *m*/*z* calcd for $C_{19}H_{38}NO_3Si_3$ $[M+H]^+$: 412.2154, found 412.2153.

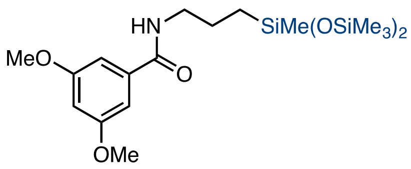

3Na

***N*-(3-(1,1,1,3,5,5,5-Heptamethyltrisiloxan-3-yl)propyl)-3,5-dimethoxybenzamide (3Na):** The reaction was stirred for 25 h. Purification by Isolera® (hexane/ethyl acetate = 1:0 to 5:1) afforded **3Na** as a colorless oil (63.5 mg, 41%, linear/branch = 99:1). ^{1}H NMR (600 MHz, $CDCl_3$) δ 6.89 (d, *J* = 2.4 Hz, 2H), 6.57 (d, *J* = 2.4 Hz, 1H), 6.15 (brs, 1H), 3.82 (s, 6H), 3.42 (q, *J* = 7.2 Hz, 2H), 1.66–1.59 (m, 2H), 0.54–0.50 (m, 2H), 0.095 (s, 18H), 0.040 (s, 3H); ^{13}C NMR (151 MHz, $CDCl_3$) δ 167.2, 160.8, 137.2, 104.8, 103.3, 55.5, 42.7, 23.4, 14.9, 1.8, -0.32; HRMS (DART) *m*/*z* calcd for $C_{19}H_{38}NO_5Si_3$ $[M+H]^+$: 444.2052, found 444.2054.

5. Synthetic Applications

5-1. Gram-scale Reaction

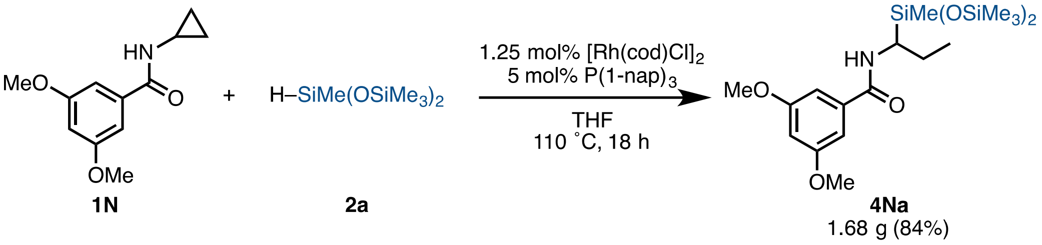

A 100 mL glass vessel tube equipped with a J. Young® O-ring tap containing a magnetic stirring bar was dried with a heatgun under reduced pressure and filled with nitrogen after cooling to room temperature. After adding *N*-cyclopropyl-3,5-dimethoxybenzamide (**1N**: 1.0 g, 4.5 mmol) and tri(naphthalen-1-yl)phosphine ($P(1\text{-nap})_3$: 93 mg, 0.23 mmol), the vessel was introduced inside an argon-atmosphere glovebox. In the glovebox, $[Rh(cod)Cl]_2$ (27.2 mg, 0.056 mmol) and THF (13 mL) were added. After 1,1,1,3,5,5,5-heptamethyltrisiloxane (**2a**: 2.5 □L, 9.0 mmol) and THF (10 mL) were added, the glass vessel was sealed with the O-ring tap and taken out of the glovebox. The mixture was stirred in an oil bath at 110 °C for 18 h, cooled to room temperature and concentrated *in vacuo*. The residue was purified by Isolera® (hexane/ethyl acetate = 9:1 to 5:1) to afford **4Na** (1.68 g, 84%, branch/linear = 88:12) as a white solid.

5-2. Tamao Oxidation

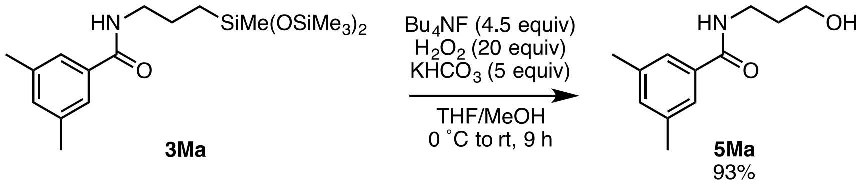

To a screw-cap 10 mL glass vessel containing a magnetic stirring bar was added **3Ma** (30.0 mg, 0.073 mmol). The contents were evacuated, then filled with nitrogen (repeat for a total of 3 times). After THF (1.4 mL) was added, the solution was cooled to 0 °C. To the solution was added tetrabutylammonium fluoride (Bu_4NF: 1 M in THF, 0.32 mL, 0.32 mmol), which was then stirred at 0 °C for 10 min. MeOH (0.5 mL), $KHCO_3$ (36.5 mg, 0.37 mmol) and 30% aqueous H_2O_2 (0.17 mL, 1.5 mmol) were added, and the mixture was stirred at room temperature for 9 h. The reaction was quenched by adding saturated aqueous $Na_2S_2O_3$. The mixture was extracted with diethyl ether and the combined organic layers were washed with brine and concentrated *in vacuo*. The residue was purified by PTLC (hexane/ethyl acetate = 1:1) to afford **5Ma** (14.0 mg, 93%) as a colorless oil. ***N*-(3-Hydroxypropyl)-3,5-dimethylbenzamide (5Ma)**[6]**:** ^{1}H NMR (500 MHz, $CDCl_3$) δ 7.37 (s, 2H), 7.13 (s, 1H), 6.63 (brs, 1H), 3.70 (q, *J* = 6.0 Hz, 2H),

[6] Tachibana, Y. ; Kawasaki, H. ; Kihara, N. ; Takata, T. *J. Org. Chem.* **2006**, *71*, 5093.

3.62 (q, *J* = 6.0 Hz, 2H), 3.36 (t, *J* = 6.0 Hz, 1H), 2.34 (s, 6H), 1.78 (quin, *J* = 6.0 Hz, 2H); ^{13}C NMR (126 MHz, $CDCl_3$) δ 169.0, 138.3, 134.1, 133.2, 124.7, 59.4, 36.8, 32.3, 21.2; HRMS (ESI) *m*/*z* calcd for $C_{12}H_{17}NNaO_2$ $[M+Na]^+$: 230.1151, found 230.1150.

6. Mechanistic Considerations

6-1. Isolation of reaction intermediates in the Rh-catalyzed hydrosilylation

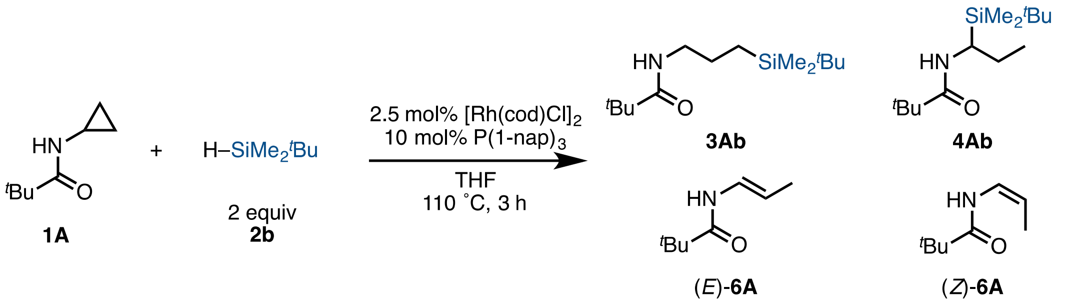

A 20 mL glass vessel tube equipped with a J. Young® O-ring tap containing a magnetic stirring bar was dried with a heatgun under reduced pressure and filled with nitrogen after cooling to room temperature. To this vessel was added *N*-cyclopropylpivalamide (49.4 mg, 0.35 mmol), $[Rh(cod)Cl]_2$ (4.3 mg, mmol, 8.8 μmol), and $(P(1\text{-nap})_3)$ (14.4 mg, mmol, 35.0 μmol), after which it was introduced inside an argon atmosphere glovebox. In the glovebox, *tert*-butyldimethylsilane (0.11 mL, 0.70 mmol) and THF (2.0 mL) were added to the vessel. After the vessel was sealed with the O-ring tap, the vessel was taken out of the glovebox. The mixture was heated at 110 ˚C for 3 h in an 8-well reaction block with stirring. After cooling the reaction mixture to room temperature, the mixture was concentrated *in vacuo*. The NMR yield was determined by ^{1}H NMR analysis of the crude product using dibromomethane as an internal standard.
NMR yields: **3Ab**: 8%, **4Ab**: 38%, (*E*)-**6A**: 35%, (*Z*)-**6A**: 21%
The residue was purified by Isolera® (hexane/ethyl acetate = 1:0 to 5:1) to afford **4Ab** (32.9 mg, 37%, a white solid), (*E*)-**6A** (16.4 mg, 33%, a white solid) and (*Z*)-**6A** (8.0 mg, 16%, a white solid).

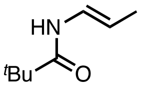

(*E*)-**6A**

(*E*)-*N*-(Prop-1-en-1-yl)pivalamide ((*E*)-6A)[7]: ^{1}H NMR (600 MHz, $CDCl_3$) δ 7.06 (brs, 1H), 6.79–6.73 (m, 1H), 5.19–5.12 (m, 1H), 1.68 (dd, *J* = 7.2, 1.2 Hz, 3H), 1.22 (s, 9H); ^{13}C NMR (151 MHz, $CDCl_3$) δ 175.2, 123.6, 107.5, 38.6, 27.4, 14.8; HRMS (ESI) *m*/*z* calcd for $C_8H_{15}NNaO$ $[M+Na]^+$: 164.1046, found 164.1047.

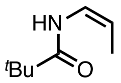

(*Z*)-**6A**

[7] Halli, J.; Kramer, P.; Bechthold, M.; Manolikakes, G. *Adv. Synth. Catal.* **2015**, *357*, 3321.

(*Z*)-*N*-(Prop-1-en-1-yl)pivalamide ((*Z*)-6A)[7]: ^{1}H NMR (600 MHz, $CDCl_3$) δ 7.13 (brs, 1H), 6.76–6.70 (m, 1H), 4.85–4.78 (m, 1H), 1.62 (d, *J* = 6.6 Hz, 3H), 1.25 (s, 9H); ^{13}C NMR (151 MHz, $CDCl_3$) δ 175.3, 122.3, 104.9, 38.9, 27.5, 10.7; HRMS (ESI) *m*/*z* calcd for $C_8H_{15}NNaO$ $[M+Na]^+$: 164.1046, found 164.1048.

6-2. Hydrosilylation of reaction intermediates (*E*)-6A and (*Z*)-6A

6-2-1. Reaction of (*E*)-6A or (*Z*)-6A with $[Rh(cod)Cl]_2$/$P(1\text{-nap})_3$

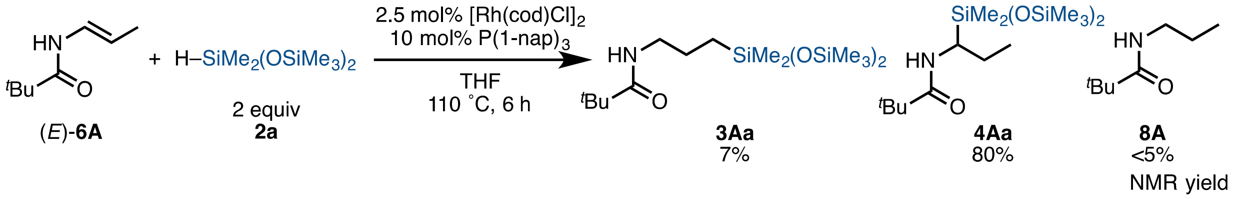

A 20 mL glass vessel tube equipped with a J. Young® O-ring tap containing a magnetic stirring bar was dried with a heatgun under reduced pressure and filled with nitrogen after cooling to room temperature. To this vessel was added (*E*)-*N*-(prop-1-en-1-yl)pivalamide ((*E*)-**6A**: 60.0 mg, 0.42 mmol), $[Rh(cod)Cl]_2$ (5.2 mg, 0.011 mmol), and P(1-nap) $_3$ (17.5 mg, 0.042 mmol), after which it was introduced inside an argon atmosphere glovebox. In the glovebox, 1,1,1,3,5,5,5-heptamethyltrisiloxane (**2a**: 0.23 mL, 0.85 mmol) and THF (2.4 mL) were added to the vessel. After the vessel was sealed with the O-ring tap, the reaction vessel was taken out of the glovebox. The mixture was heated at 110 °C for 6 h in an 8-well reaction block with stirring. After cooling the reaction mixture to room temperature, the mixture was concentrated *in vacuo*. The NMR yield was determined by ^{1}H NMR analysis of the crude product using dibromomethane as an internal standard.

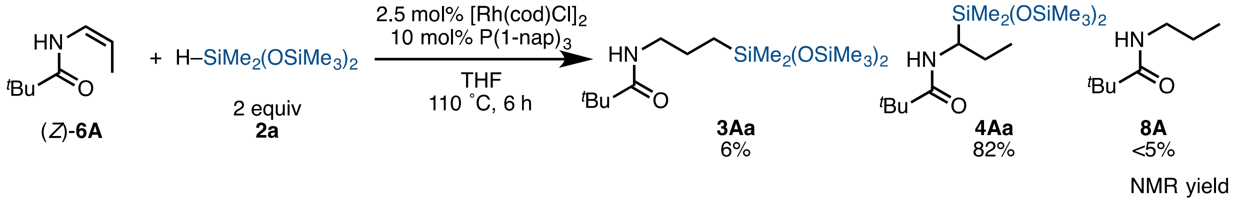

A 20 mL glass vessel tube equipped with a J. Young® O-ring tap containing a magnetic stirring bar was dried with a heatgun under reduced pressure and filled with nitrogen after cooling to room temperature. To this vessel was added (*Z*)-*N*-(prop-1-en-1-yl)pivalamide ((*Z*)-**6A**: 50.0 mg, 0.35 mmol), $[Rh(cod)Cl]_2$ (4.3 mg, 0.089 mmol), and P(1-nap) $_3$ (14.6 mg, 0.035 mmol), after which it was introduced inside an argon atmosphere glovebox. In the glovebox, 1,1,1,3,5,5,5-heptamethyltrisiloxane (**2a**: 0.19 mL, 0.71 mmol) and THF (2.0 mL) were added to the vessel. After the vessel was sealed with the O-ring tap, the reaction vessel was taken out of the glovebox. The mixture was heated at 110 °C for 6 h in an 8-well reaction block with stirring. After cooling the reaction mixture to room temperature, the mixture was

concentrated *in vacuo*. The NMR yield was determined by ^{1}H NMR analysis of the crude product using dibromomethane as an internal standard.

These results indicated that the hydrosilylated product **4Aa** would be formed through Rh-catalyzed hydrosilylation of enamide **6A**.

6-2-2. Reaction of allylamine 7A with [Rh(cod)Cl]$_2$/P(1-nap)$_3$

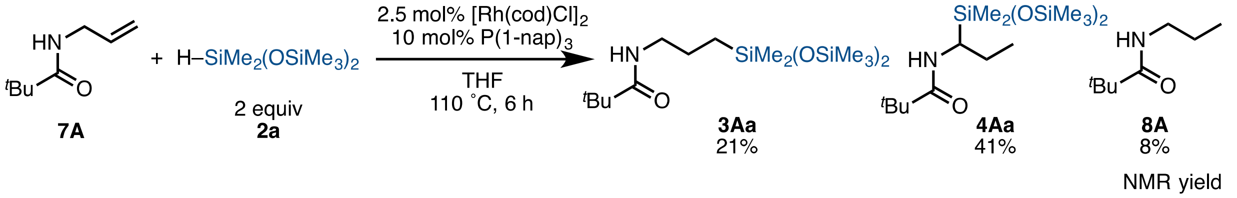

A 20 mL glass vessel tube equipped with a J. Young® O-ring tap containing a magnetic stirring bar was dried with a heatgun under reduced pressure and filled with nitrogen after cooling to room temperature. To this vessel was added *N*-allylpivalamide[8] (**7A**: 49.4 mg, 0.35 mmol), [Rh(cod)Cl]$_2$ (4.3 mg, 0.0088 mmol), and P(1-nap)$_3$ (14.4 mg, 0.035 mmol), after which it was introduced inside an argon atmosphere glovebox. In the glovebox, 1,1,1,3,5,5,5-heptamethyltrisiloxane (**2a**: 0.19 mL, 0.70 mmol) and THF (2.0 mL) were added to the vessel. After the vessel was sealed with the O-ring tap, the reaction vessel was taken out of the glovebox. The mixture was heated at 110 ˚C for 6 h in an 8-well reaction block with stirring. After cooling the reaction mixture to room temperature, the mixture was concentrated *in vacuo*. The NMR yield was determined by ^{1}H NMR analysis of the crude product using dibromomethane as an internal standard.

The result suggested that the isomerization of allylamine to enamide took place *in situ* in the presence of P(1-nap)$_3$.

6-2-3. Reaction of (*E*)-6A or (*Z*)-6A with [Rh(cod)Cl]$_2$/PCy$_3$·HBF$_4$

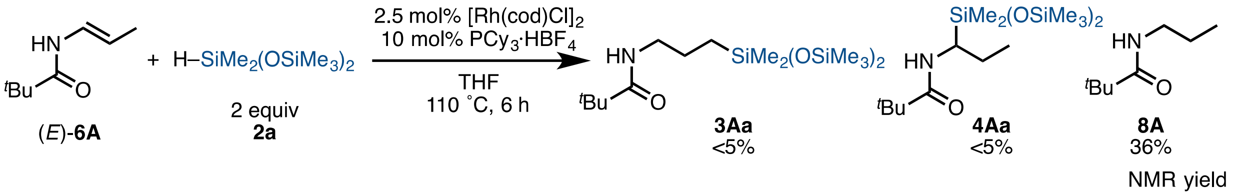

A 20 mL glass vessel tube equipped with a J. Young® O-ring tap containing a magnetic stirring bar was dried with a heatgun under reduced pressure and filled with nitrogen after cooling to room temperature. To this vessel was added (*E*)-*N*-(prop-1-en-1-yl)pivalamide ((*E*)-**6A**: 70.0 mg, 0.50 mmol), [Rh(cod)Cl]$_2$ (6.1 mg, 0.012 mmol), and PCy$_3$·HBF$_4$ (18.3 mg, 0.050 mmol), after which it was introduced inside an argon atmosphere glovebox. In the glovebox, 1,1,1,3,5,5,5-heptamethyltrisiloxane (**2a**: 0.27 mL, 0.99

[8] Moon, N. G. ; Harned, A. M. *Tetrahedron Lett.* **2013**, *54*, 2960.

mmol) and THF (2.8 mL) were added to the vessel. After the vessel was sealed with the O-ring tap, the reaction vessel was taken out of the glovebox. The mixture was heated at 110 ˚C for 6 h in an 8-well reaction block with stirring. After cooling the reaction mixture to room temperature, the mixture was concentrated *in vacuo*. The NMR yield was determined by ^{1}H NMR analysis of the crude product using dibromomethane as an internal standard.

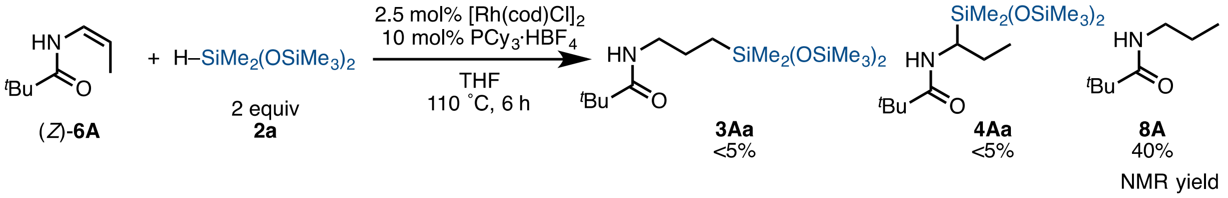

A 20 mL glass vessel tube equipped with a J. Young® O-ring tap containing a magnetic stirring bar was dried with a heatgun under reduced pressure and filled with nitrogen after cooling to room temperature. To this vessel was added (*Z*)-*N*-(prop-1-en-1-yl)pivalamide ((*Z*)-**6A**: 68.0 mg, 0.48 mmol), $[Rh(cod)Cl]_2$ (5.9 mg, 0.012 mmol), and $PCy_3·HBF_4$ (17.7 mg, 0.048 mmol), after which it was introduced inside an argon atmosphere glovebox. In the glovebox, 1,1,1,3,5,5,5-heptamethyltrisiloxane (**2a**: 0.26 mL, 0.96 mmol) and THF (2.8 mL) were added to the vessel. After the vessel was sealed with the O-ring tap, the reaction vessel was taken out of the glovebox. The mixture was heated at 110 ˚C for 6 h in an 8-well reaction block with stirring. After cooling the reaction mixture to room temperature, the mixture was concentrated *in vacuo*. The NMR yield was determined by ^{1}H NMR analysis of the crude product using dibromomethane as an internal standard.

These results showed that hydrogenated product **8A** was formed when $PCy_3·HBF_4$ was used as the ligand in the reaction of enamide **6A** and that hydrosilylation of enamide **6A** was suppressed.

6-2-4. Reaction of allylamine 7A with $[Rh(cod)Cl]_2/PCy_3·HBF_4$

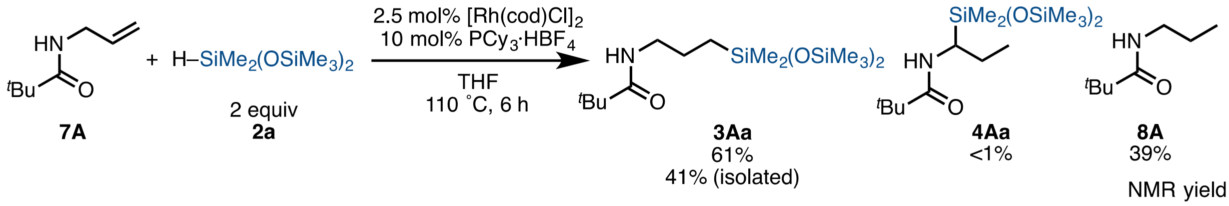

A 20 mL glass vessel tube equipped with a J. Young® O-ring tap containing a magnetic stirring bar was dried with a heatgun under reduced pressure and filled with nitrogen after cooling to room temperature. To this vessel was added *N*-allylpivalamide[8] (**7A**: 49.4 mg, 0.35 mmol), $[Rh(cod)Cl]_2$ (4.3 mg, 0.0088

[8] Moon, N. G. ; Harned, A. M. *Tetrahedron Lett.* **2013**, *54*, 2960.

mmol), and $PCy_3 \cdot HBF_4$ (12.9 mg, 0.035 mmol), after which it was introduced inside an argon atmosphere glovebox. In the glovebox, 1,1,1,3,5,5,5-heptamethyltrisiloxane (**2a**: 0.19 mL, 0.70 mmol) and THF (2.0 mL) were added to the vessel. After the vessel was sealed with the O-ring tap, the reaction vessel was taken out of the glovebox. The mixture was heated at 110 ˚C for 6 h in an 8-well reaction block with stirring. After cooling the reaction mixture to room temperature, the mixture was concentrated *in vacuo*. The NMR yield was determined by ^{1}H NMR analysis of the crude product using dibromomethane as an internal standard. The residue was purified by Isolera® (hexane/ethyl acetate = 1:0 to 5:1) to afford **3Aa** (52.7 mg, 41%) as a colorless oil.

This result indicated that the formation of linear product **3** arises from the hydrosilylation of allylamine.

6-3. Isomerization of allylamine to enamide under $[Rh(cod)Cl]_2$/$P(1\text{-}nap)_3$ catalytic condition

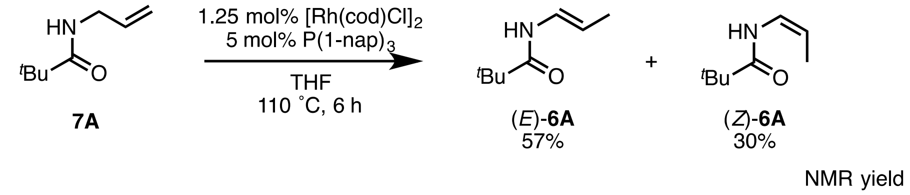

A 20 mL glass vessel tube equipped with a J. Young® O-ring tap containing a magnetic stirring bar was dried with a heatgun under reduced pressure and filled with nitrogen after cooling to room temperature. To this vessel was added $[Rh(cod)Cl]_2$ (2.2 mg, 0.0044 mmol), $P(1\text{-}nap)_3$ (7.2 mg, 0.018 mmol) and THF (2.0 mL), then *N*-allylpivalamide[8] (**7A**: 49.4 mg, 0.35 mmol) was added under nitrogen. After the vessel was sealed with the O-ring tap, the mixture was heated at 110 ˚C for 6 h in an 8-well reaction block with stirring. After cooling the reaction mixture to room temperature, the mixture was concentrated *in vacuo*. The NMR yield was determined by ^{1}H NMR analysis of the crude product using dibromomethane as an internal standard.

This result showed that allylamine is isomerized to enamide in the presence of $[Rh(cod)Cl]_2$ and $P(1\text{-}nap)_3$. The ligand effect of $P(1\text{-}nap)_3$ is supposed to control isomerization of allylamine to enamide.

[8] Moon, N. G. ; Harned, A. M. *Tetrahedron Lett.* **2013**, *54*, 2960.

7. Proposed Reaction Mechanism

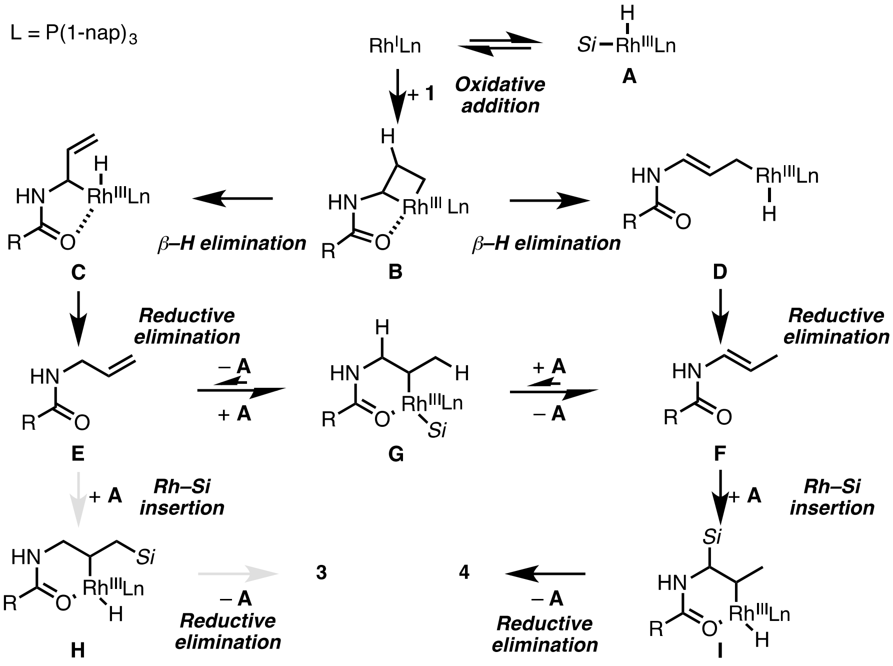

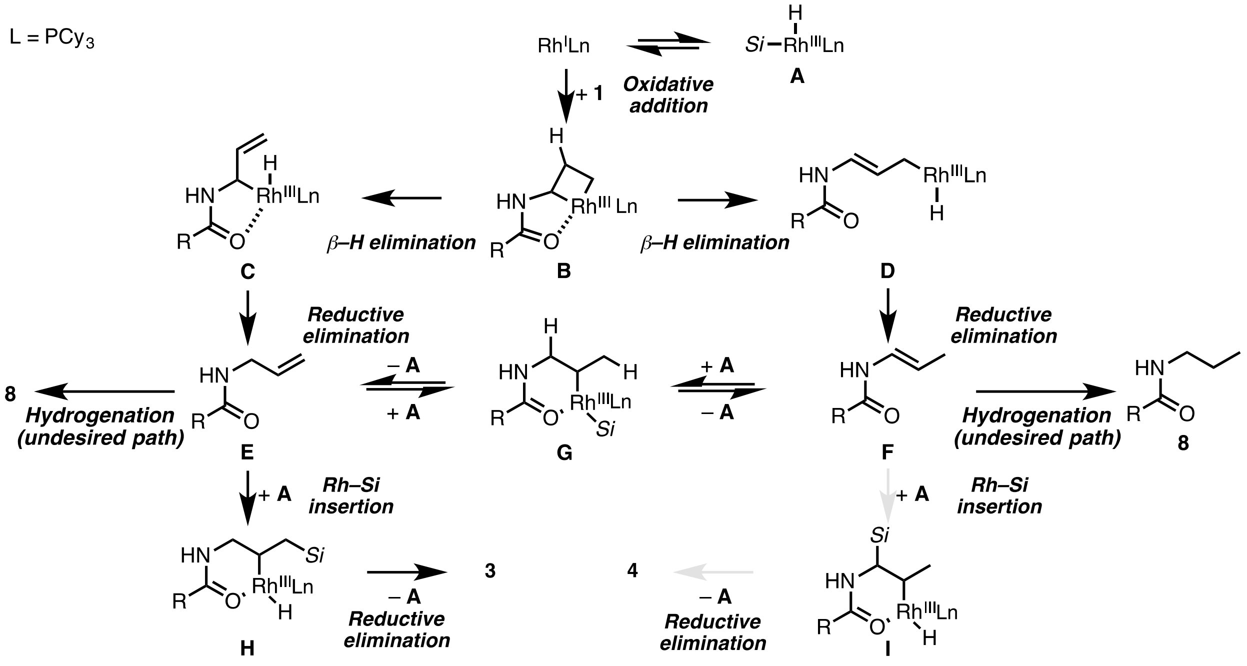

8. Effect of Reaction Parameters

[Rh(cod)Cl]$_2$/P(1-nap)$_3$ catalytic system

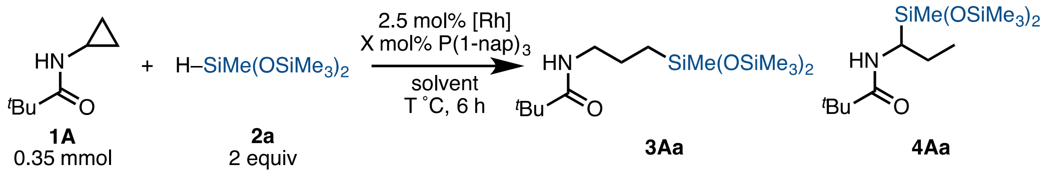

Entry	[Rh]	X mol%	Solvent	T °C	NMR yield (**3Aa**)	NMR yield (**4Aa**)
1	$[Rh(cod)Cl]_2$	5 mol%	THF	120 °C	20%	75%
2	$[Rh(cod)Cl]_2$	10 mol%	THF	120 °C	9% (4%)[a]	82% (81%)[a]
3	$[Rh(cod)Cl]_2$	15 mol%	THF	120 °C	9%	81%
4	$[Rh(cod)Cl]_2$	20 mol%	THF	120 °C	8%	76%
5	$[Rh(cod)Cl]_2$	10 mol%	THF	110 °C	10%	80%
6	$[Rh(cod)Cl]_2$	10 mol%	THF	100 °C	10%	65%
7	$[Rh(cod)OMe]_2$	10 mol%	THF	110 °C	9%	91% (88%)[a]
8	$[Rh(PPh_3)_3Cl]$	5 mol%	THF	120 °C	3%	10%
9	$[Rh(cod)Cl]_2$	10 mol%	cyclohexane	120 °C	14%	80%
10	$[Rh(cod)Cl]_2$	10 mol%	toluene	120 °C	14%	75%

NMR yield was determined by ^{1}H NMR analysis of crude products using dibromomethane as an internal standard.
[a]Isolated yield.

[Rh(cod)Cl]$_2$/PCy$_3$ catalytic system

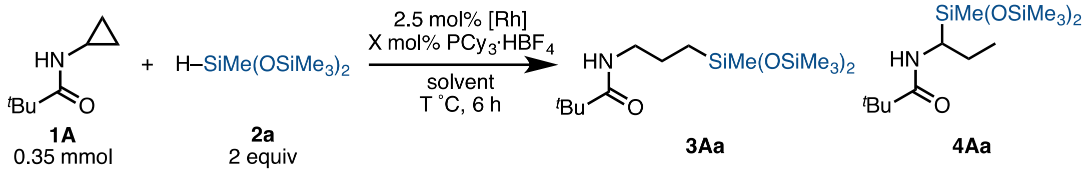

Entry	[Rh]	X mol%	Solvent	T °C	NMR yield (**3Aa**)	NMR yield (**4Aa**)
1	$[Rh(cod)Cl]_2$	5 mol%	THF	120 °C	54%	<1%
2	$[Rh(cod)Cl]_2$	10 mol%	THF	120 °C	61% (56%)[a]	<1%
3	$[Rh(cod)Cl]_2$	15 mol%	THF	120 °C	50%	<1%
4	$[Rh(cod)Cl]_2$	20 mol%	THF	120 °C	42%	<1%
5	$[Rh(cod)Cl]_2$	5 mol%	THF	110 °C	60%	<1%
6	$[Rh(cod)Cl]_2$	5 mol%	THF	100 °C	57%	<1%
7	$[Rh(cod)Cl]_2$	10 mol%	THF	110 °C	60%	3%
8	$[Rh(cod)OMe]_2$	10 mol%	THF	110 °C	47%	7%
9	$[Rh(cod)Cl]_2$	5 mol%	cyclohexane	110 °C	58%	7%
10	$[Rh(cod)Cl]_2$	5 mol%	toluene	110 °C	43%	3%

NMR yield was determined by ^{1}H NMR analysis of crude products using dibromomethane as an internal standard.
[a]Isolated yield.
In all reactions, the formation of *N*-propylpivalamide was observed.

9. Discovery of Rh-catalyzed Hydrosilylation

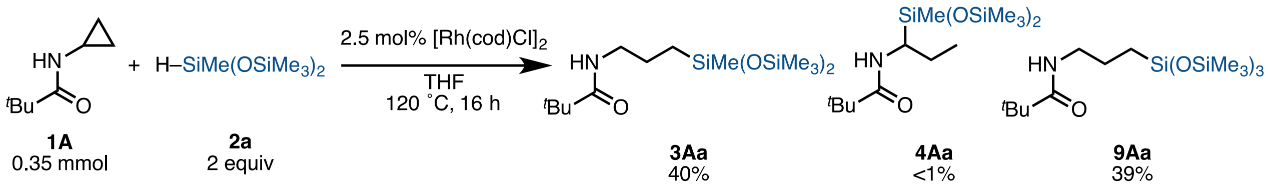

A 20 mL glass vessel tube equipped with a J. Young® O-ring tap containing a magnetic stirring bar was dried with a heatgun under reduced pressure and filled with nitrogen after cooling to room temperature. To this vessel was added *N*-cyclopropylpivalamide (**1A**: 49.5 mg, 0.35 mmol), $[Rh(cod)Cl]_2$ (4.3 mg, 8.8 μmol), after which it was introduced inside an argon atmosphere glovebox. In the glovebox, 1,1,1,3,5,5,5-heptamethyltrisiloxane (**2a**: 191 μL, 0.70 mmol) and THF (2.0 mL) were added to the vessel. After the vessel was sealed with the O-ring tap, the vessel was taken out of the glovebox. The mixture was heated at 120 ˚C for 16 h in an 8-well reaction block with stirring. After cooling the reaction mixture to room temperature, the mixture was concentrated *in vacuo*. The residue was purified by Isolera® (hexane/ethyl acetate = 5:1 to 2:1) to afford aminosilane **3Aa** (50.1 mg, 40%, colorless oil) and **9Aa** (61.8 mg, 39%, white solid).

***N*-(3-(1,1,1,5,5,5-Hexamethyl-3-((trimethylsilyl)oxy)trisiloxan-3-yl)propyl)pivalamide (9Aa):** ^{1}H NMR (600 MHz, CD_2Cl_2) δ 5.66 (brs, 1H), 3.17 (q, *J* = 6.6 Hz, 2H), 1.54–1.47 (m, 2H), 1.16 (s, 9H), 0.47–0.42 (m, 2H), 0.11 (s, 27H); ^{13}C NMR (151 MHz, CD_2Cl_2) δ 178.2, 42.3, 38.8, 27.8, 24.0, 12.0, 1.8; HRMS (ESI) *m*/*z* calcd for $C_{17}H_{43}NNaO_4Si_4$ $[M+Na]^+$: 460.2161, found 460.2160.

10. ^{1}H and ^{13}C NMR Spectra

^{1}H NMR (600MHz, $CDCl_3$) of 1B

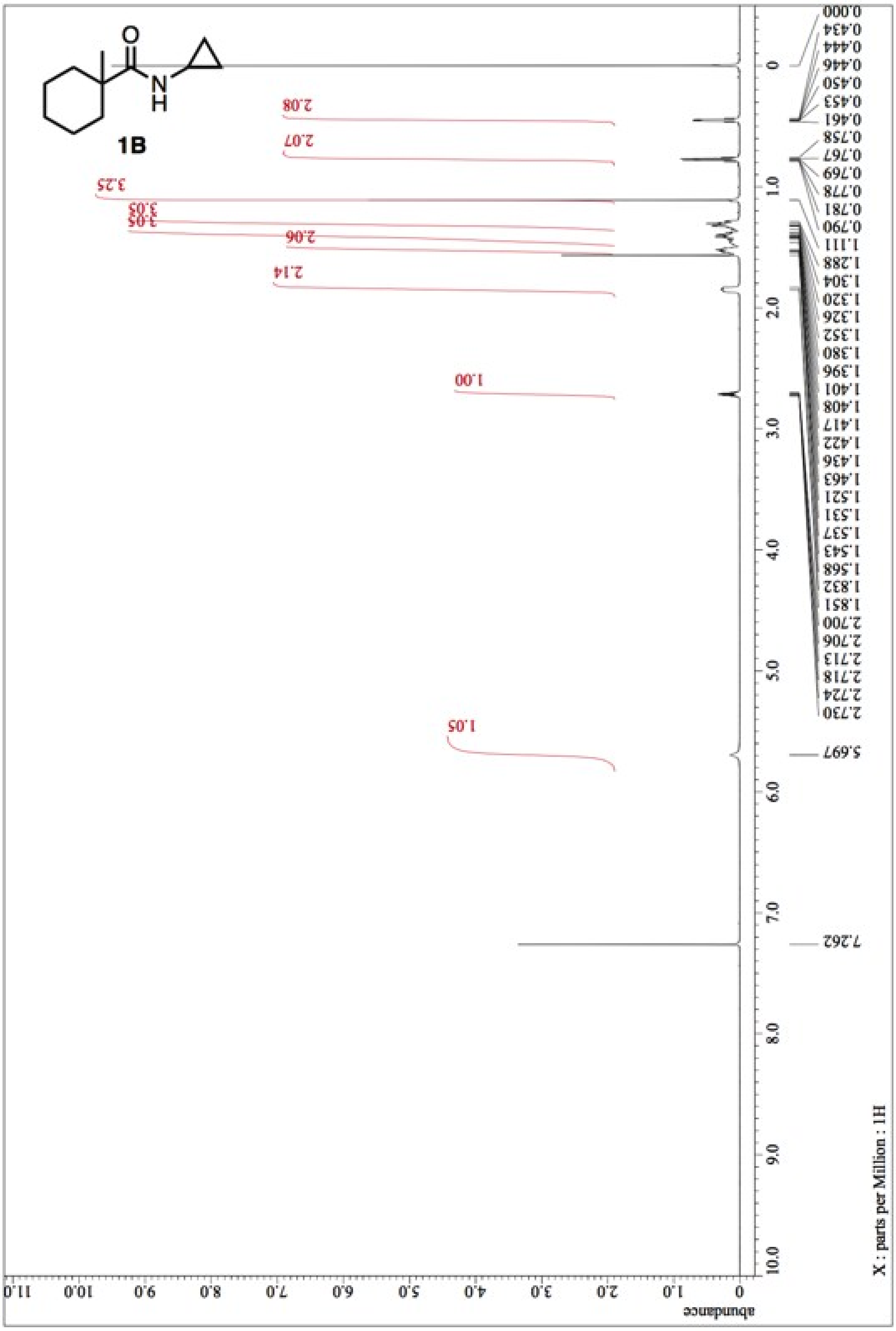

^{13}C NMR (151MHz, $CDCl_3$) of 1B

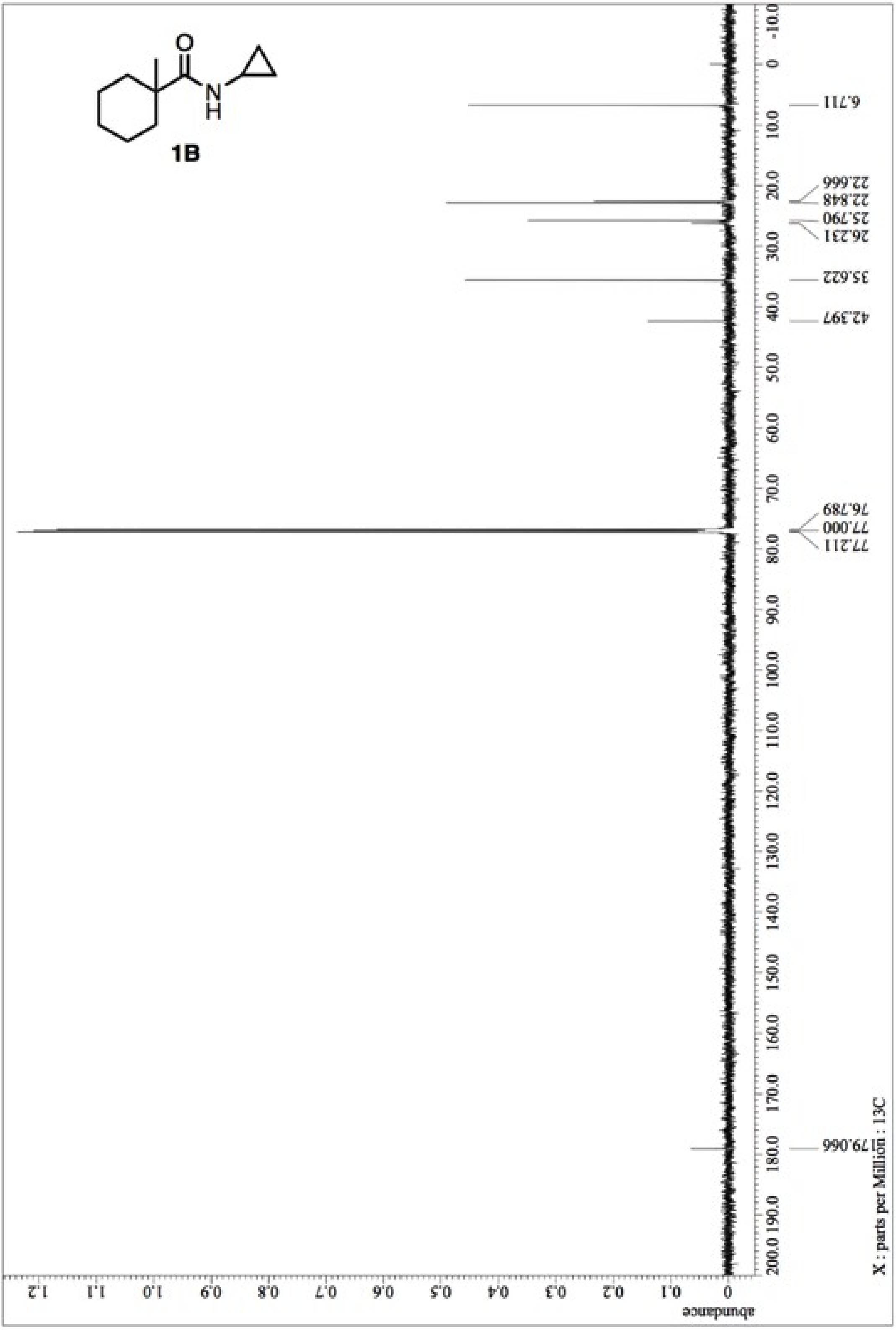

^{1}H NMR (600MHz, $CDCl_3$) of 1E

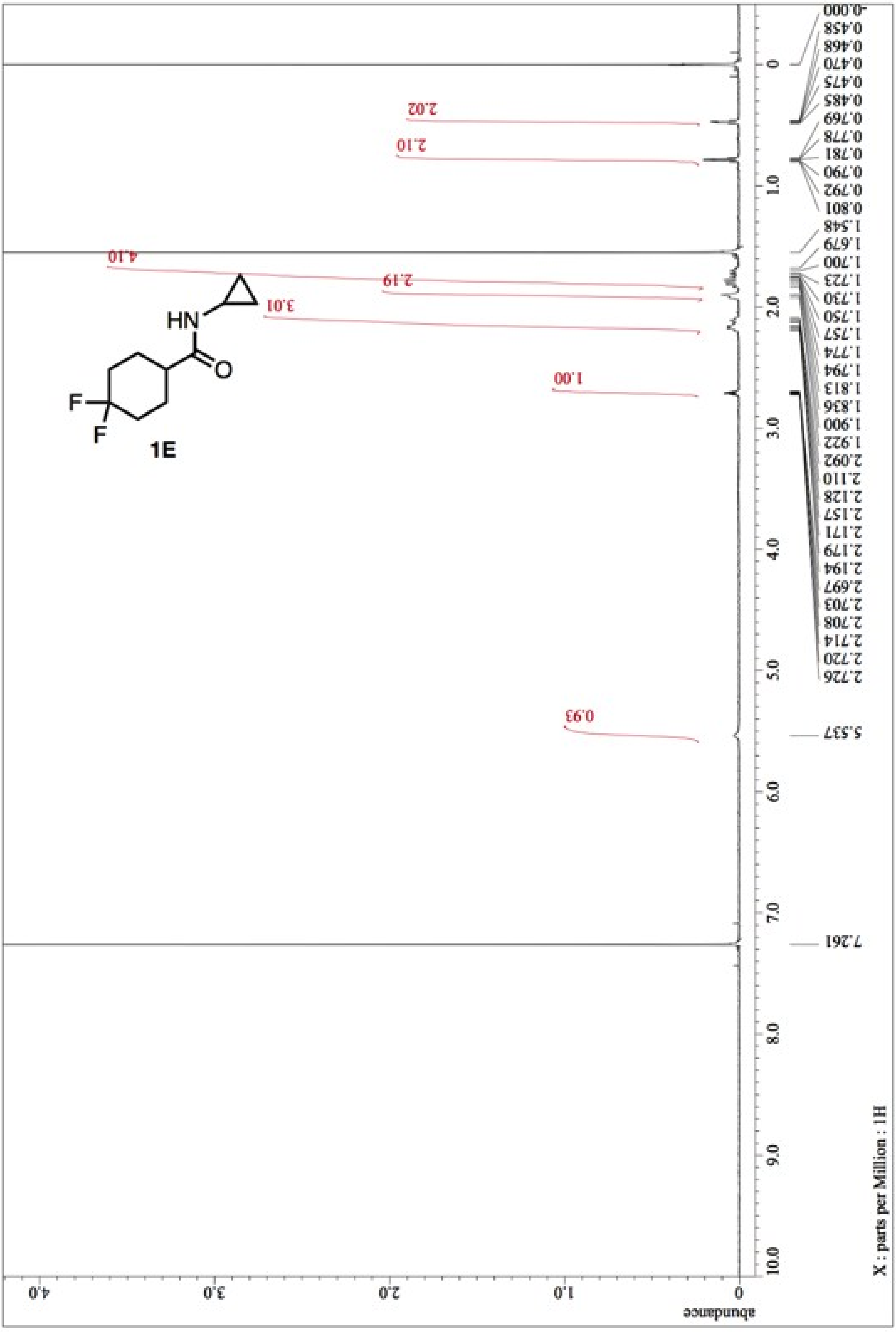

^{13}C NMR (126MHz, $CDCl_3$) of 1E

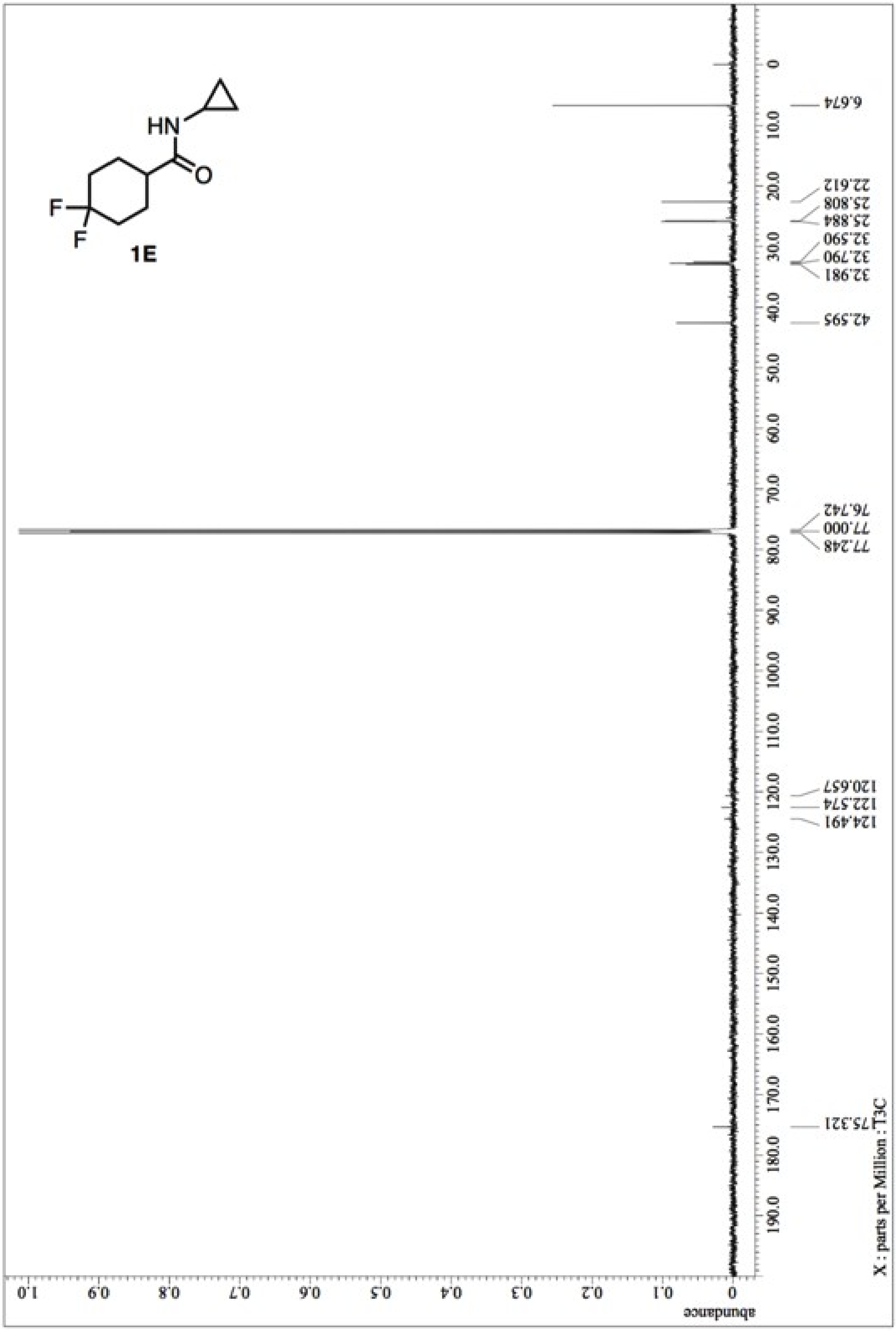

^{1}H NMR (600MHz, $CDCl_3$) of 1F

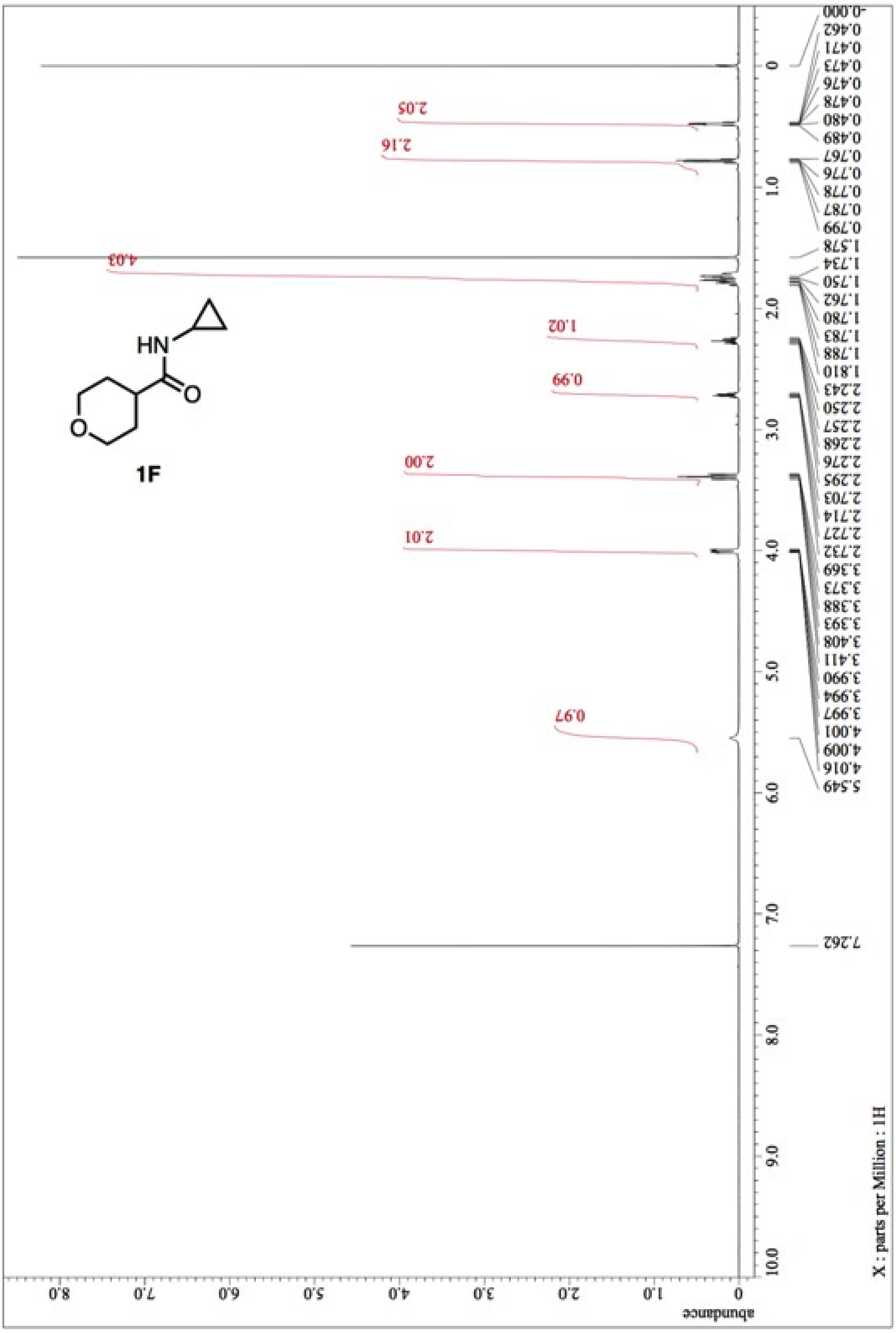

^{13}C NMR (126MHz, $CDCl_3$) of 1F

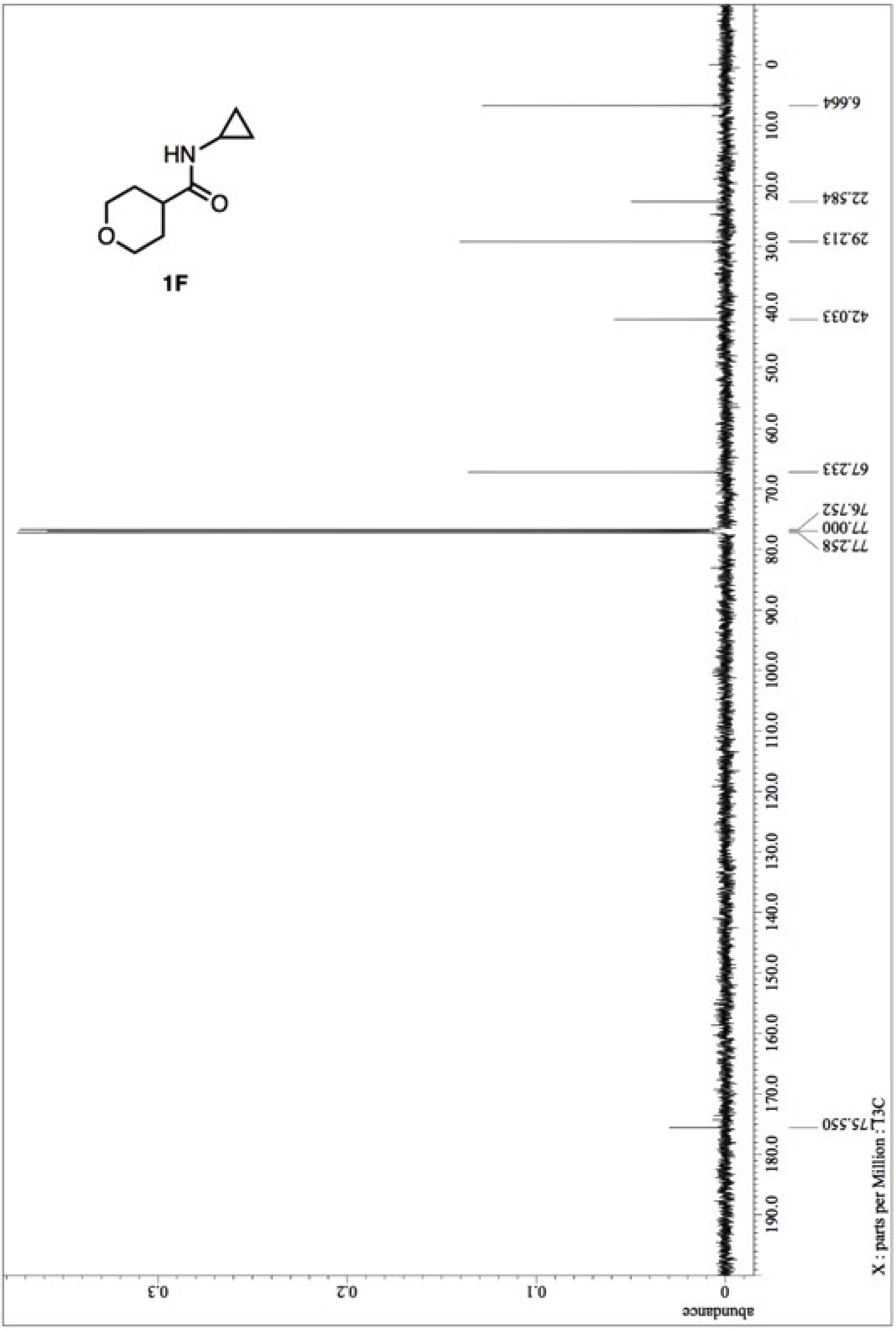

^{1}H NMR (600MHz, $CDCl_3$) of 1H

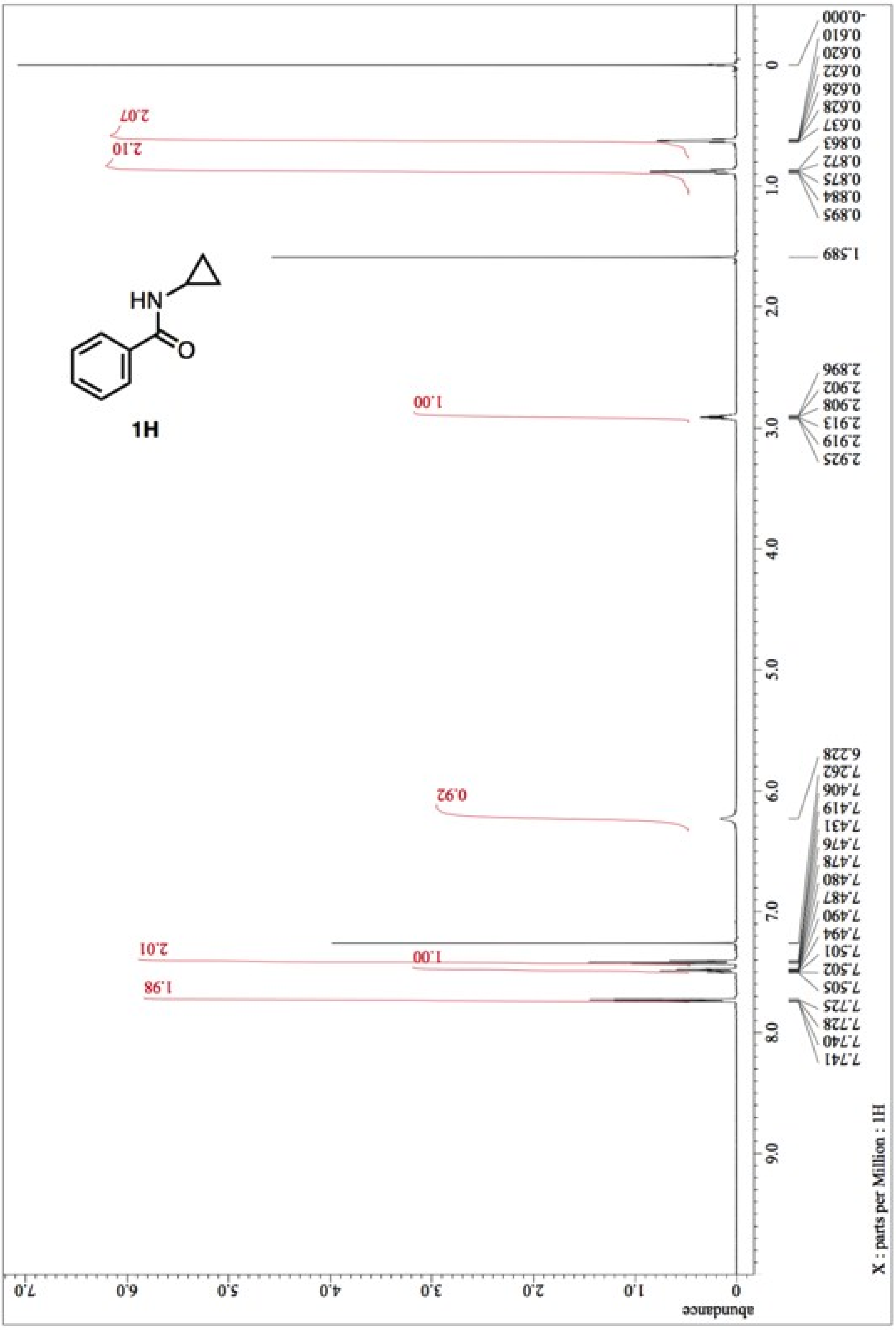

^{13}C NMR (126MHz, $CDCl_3$) of 1H

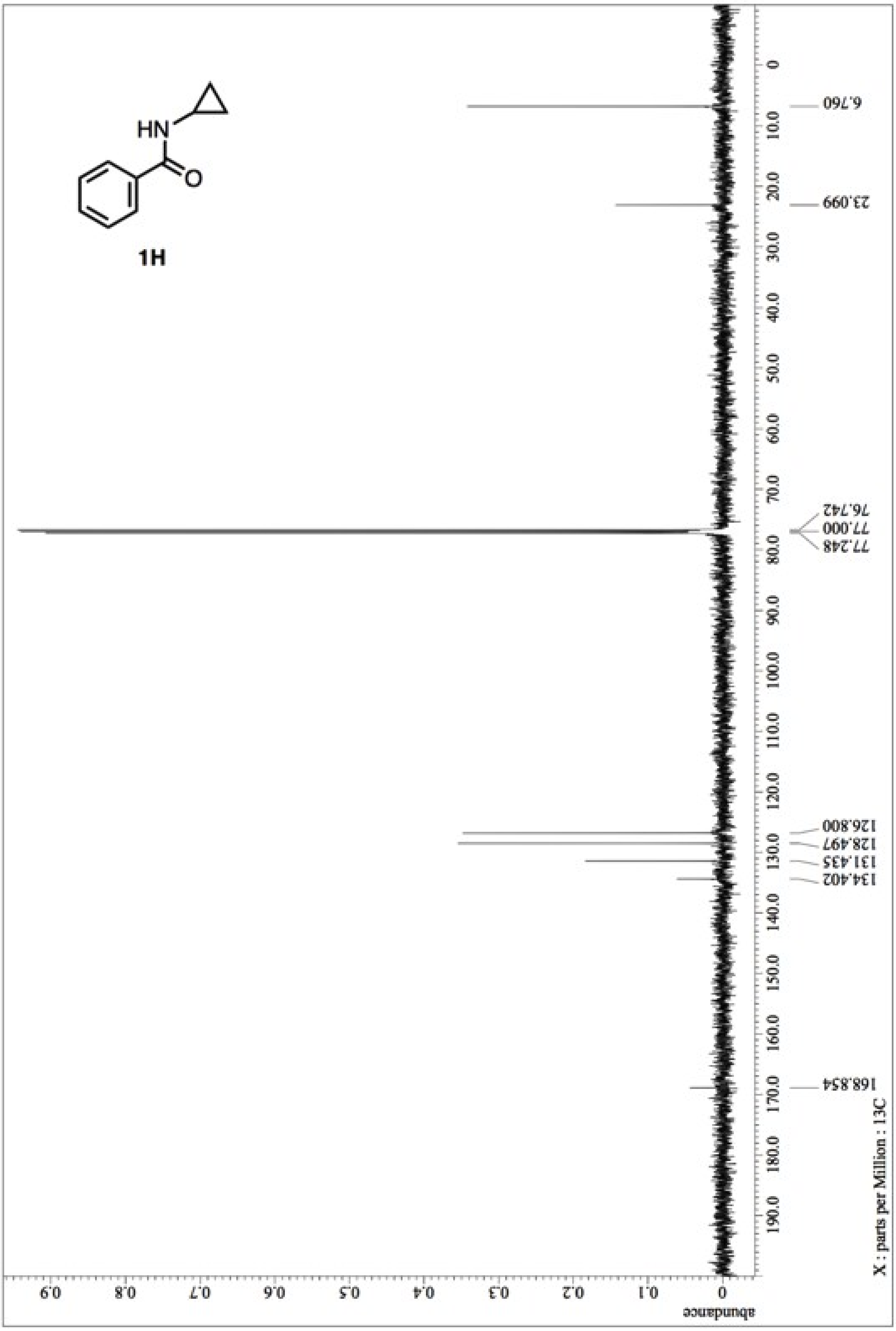

^{1}H NMR (600MHz, $CDCl_3$) of 1K

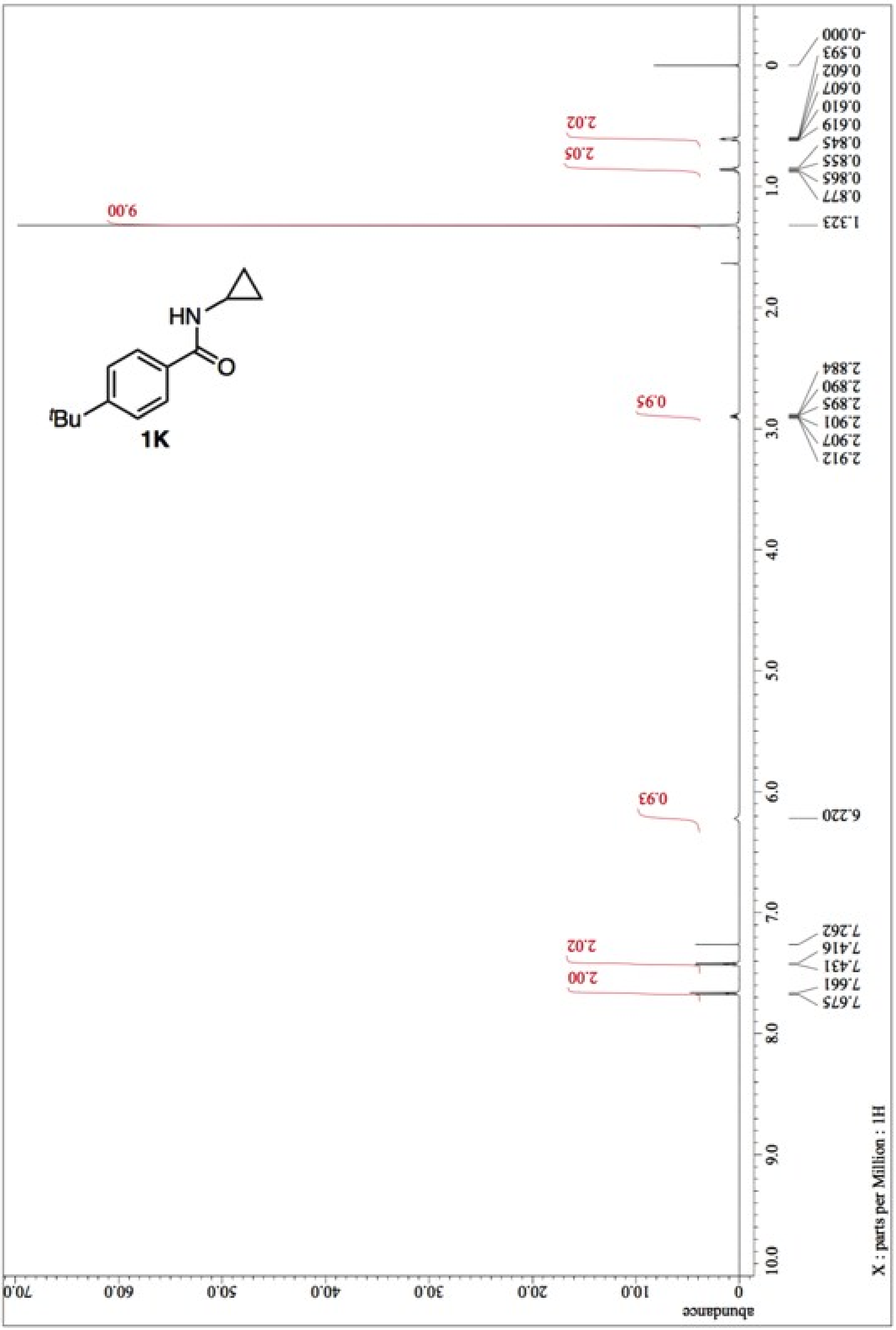

^{13}C NMR (151MHz, $CDCl_3$) of 1K

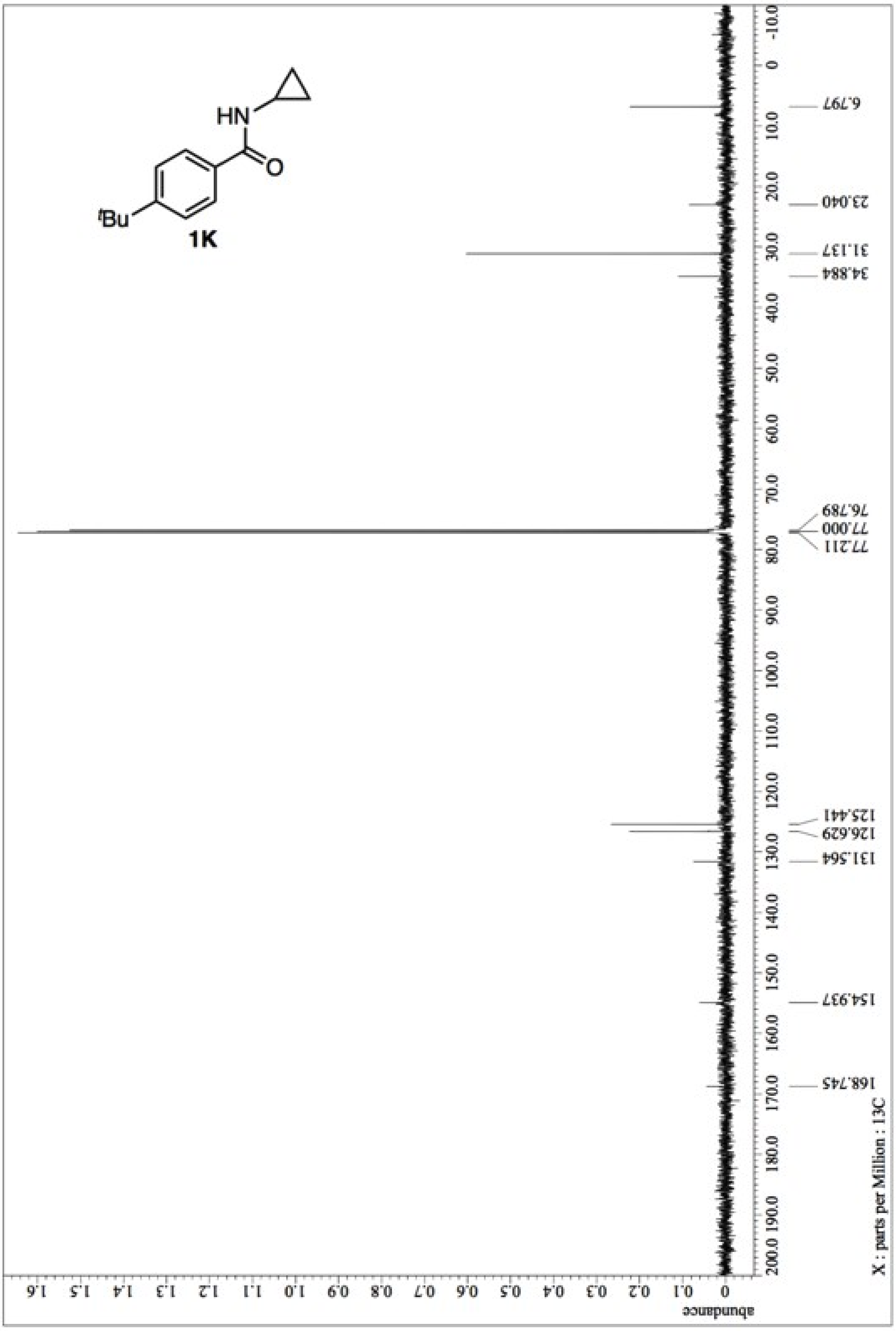

^{1}H NMR (600MHz, $CDCl_3$) of 1L

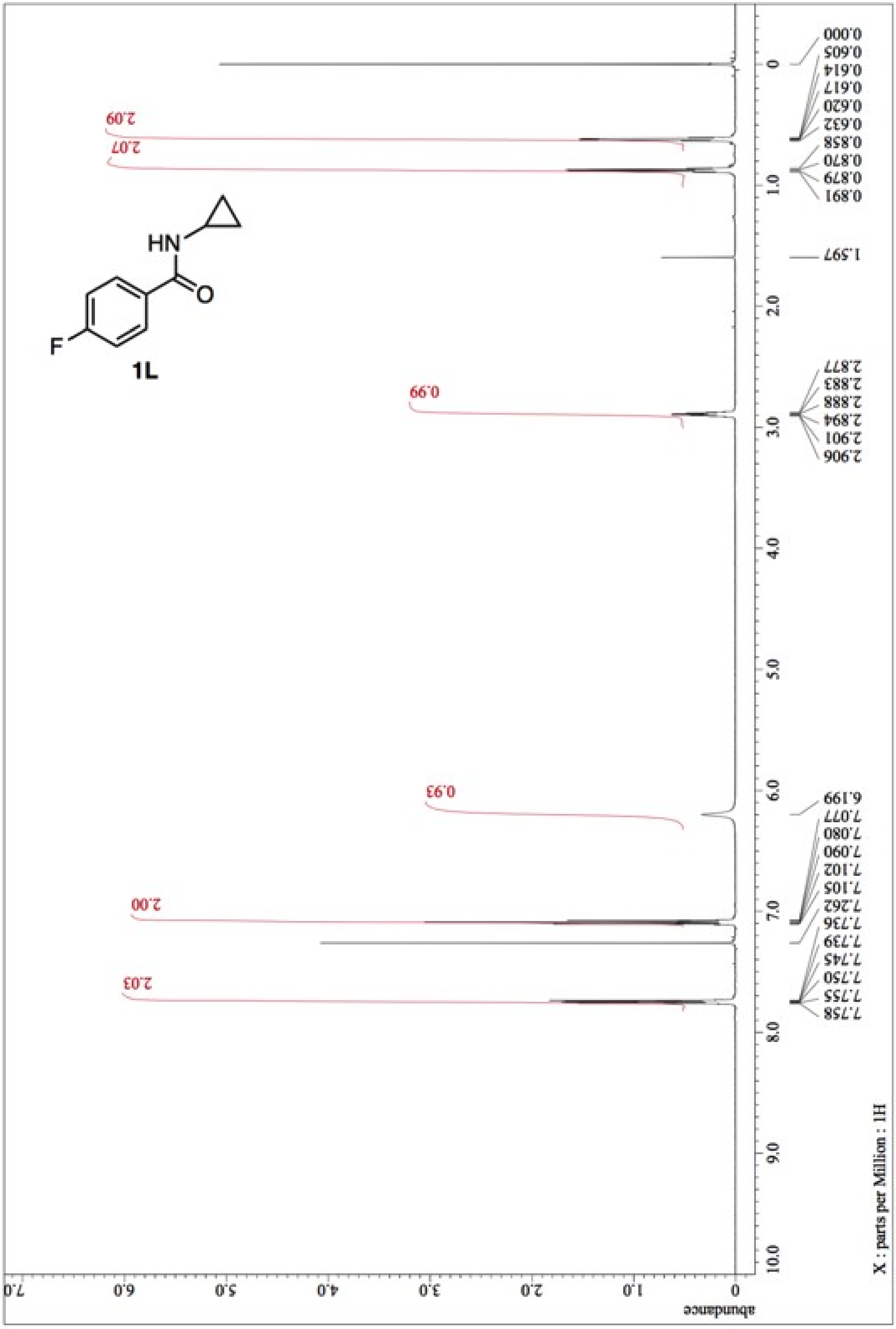

^{13}C NMR (151MHz, CDCl$_3$) of 1L

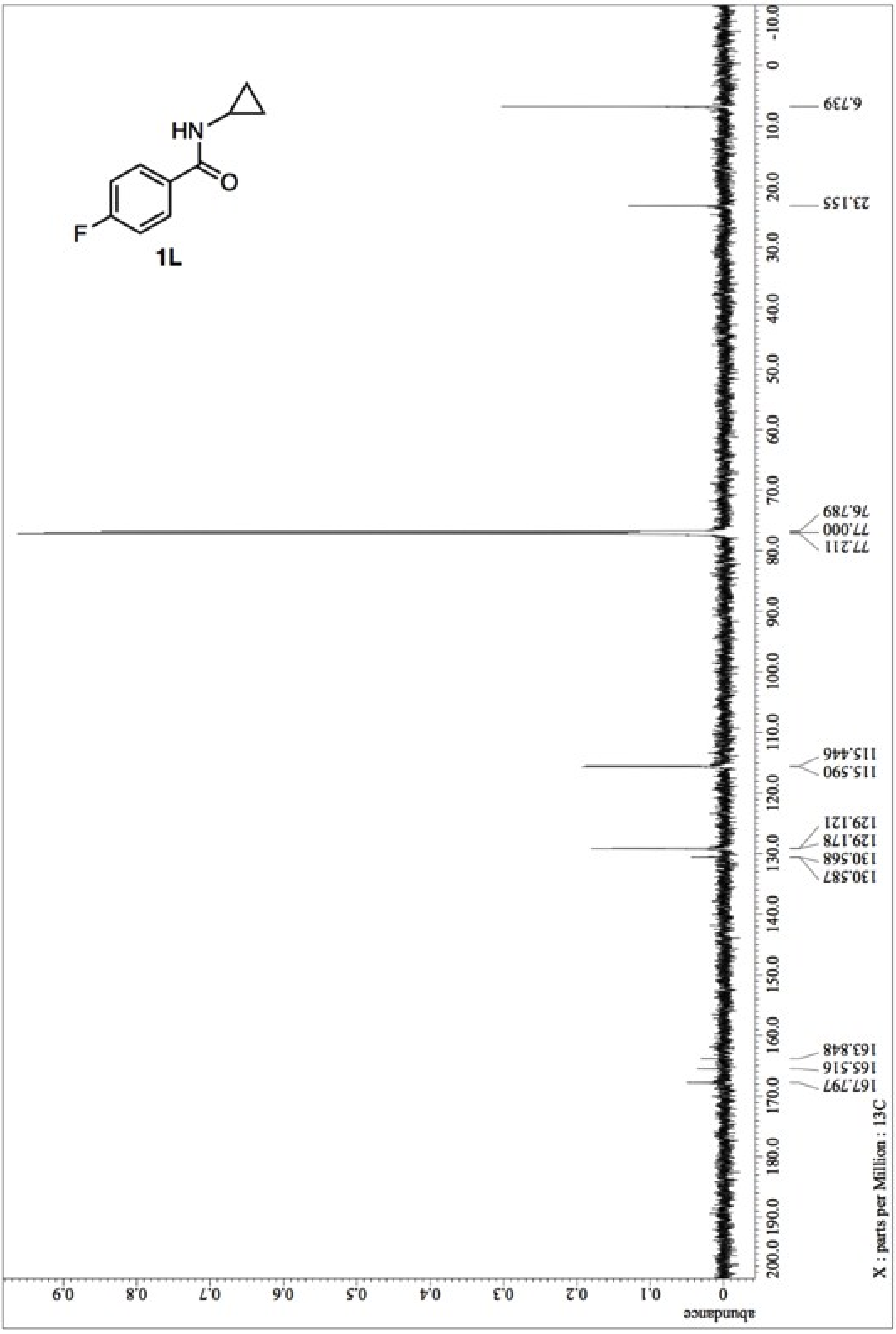

^{1}H NMR (600MHz, $CDCl_3$) of 1M

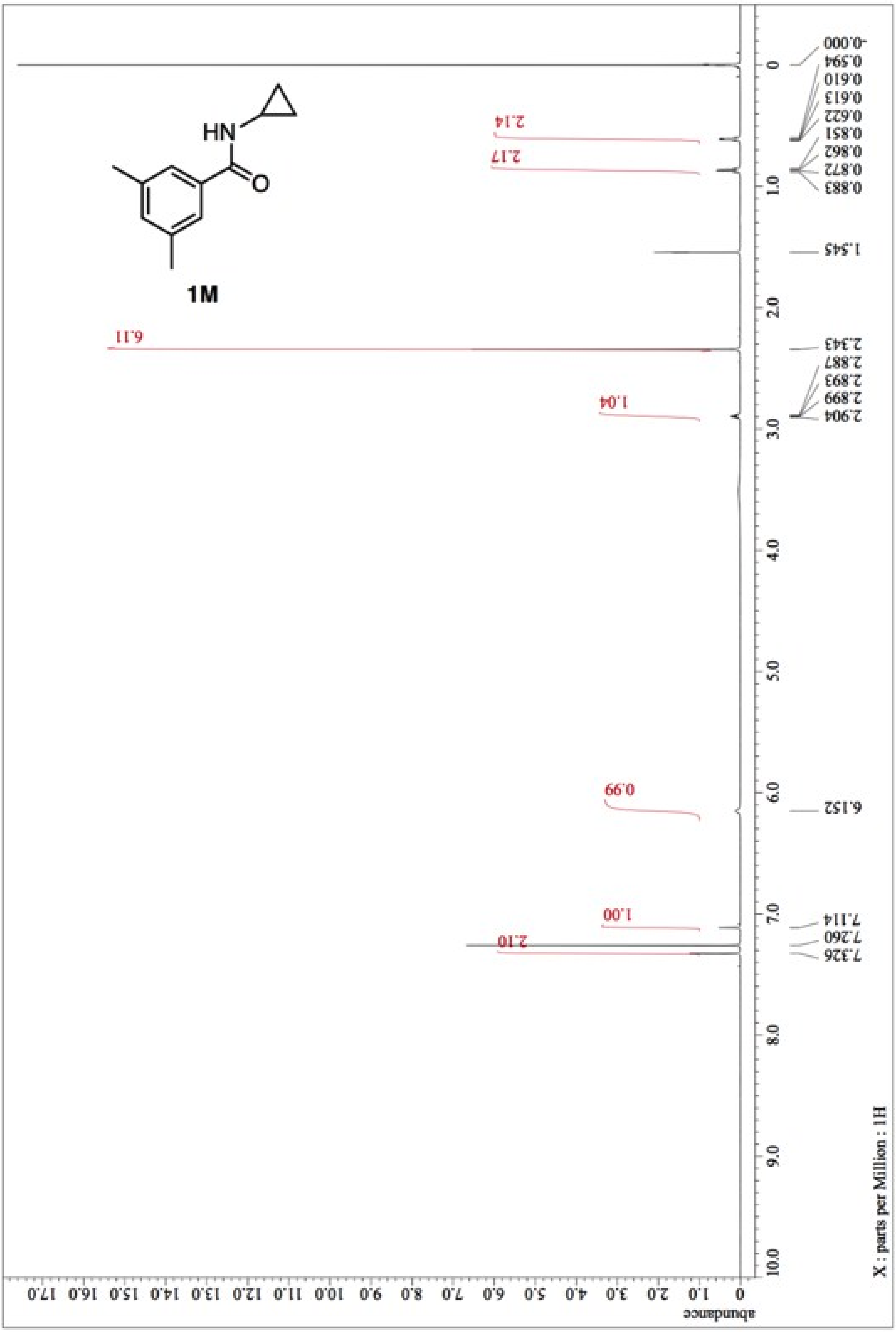

^{13}C NMR (126MHz, $CDCl_3$) of 1M

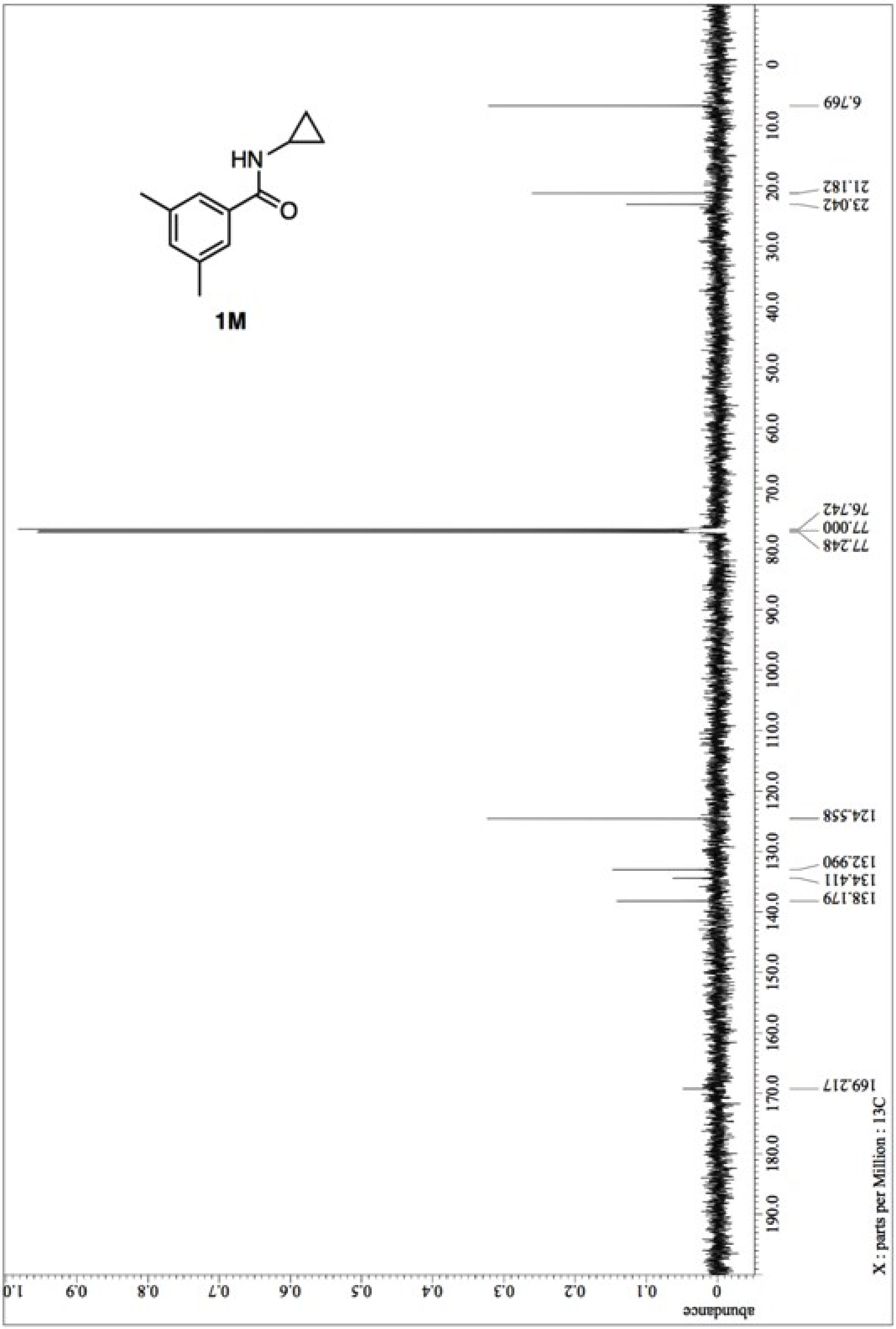

^{1}H NMR (600MHz, $CDCl_3$) of 1N

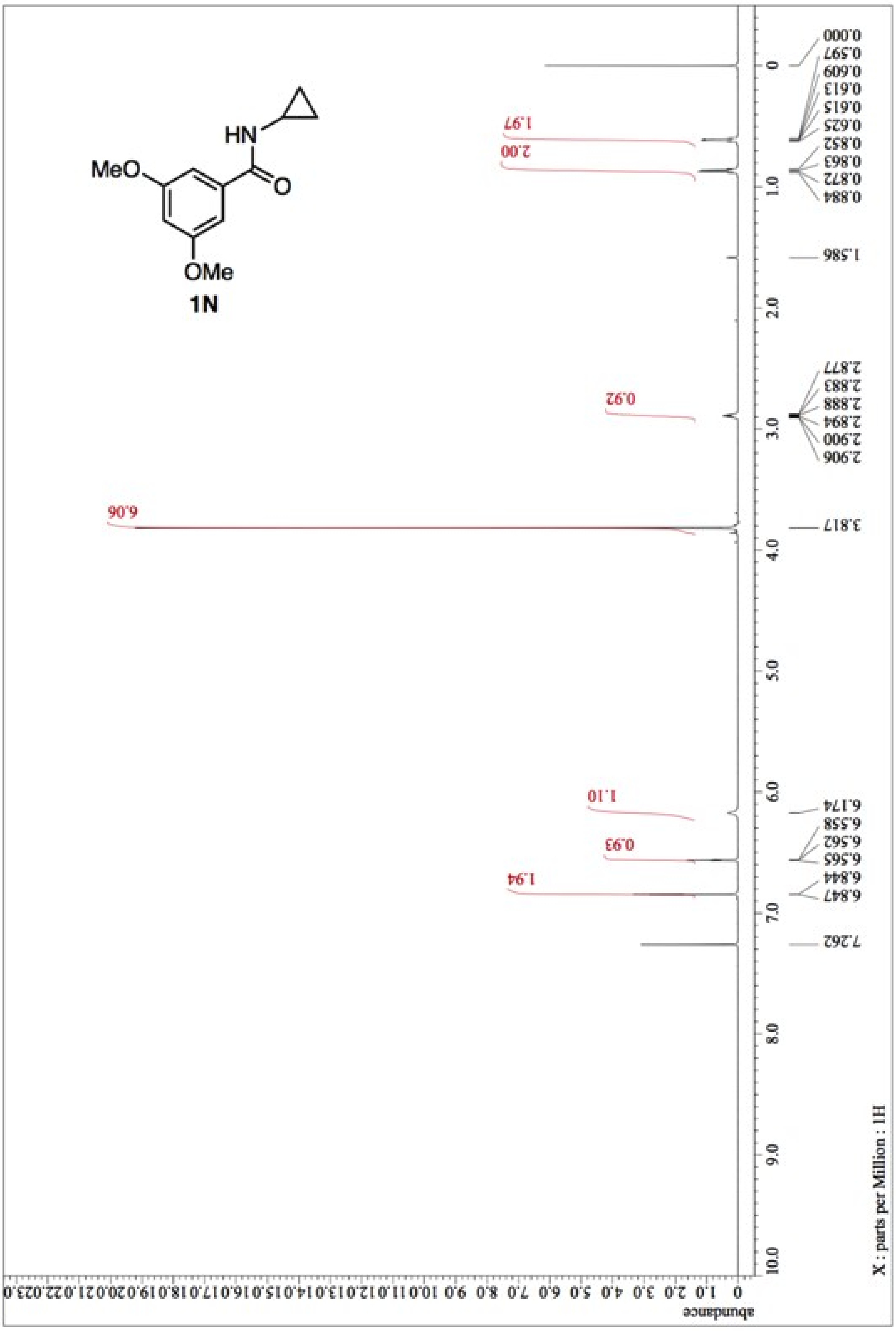

^{13}C NMR (151MHz, $CDCl_3$) of 1N

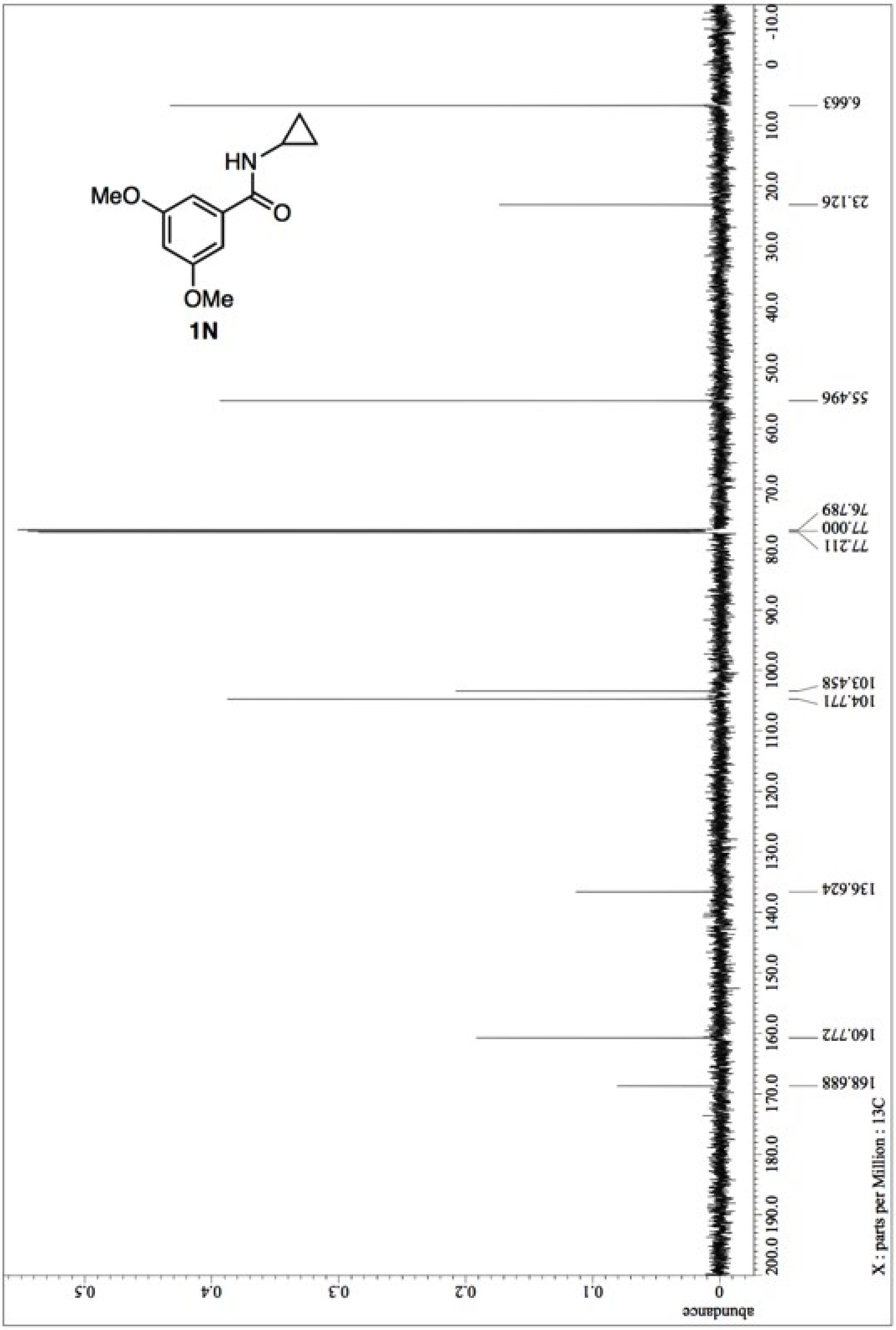

^{1}H NMR (600MHz, $CDCl_3$) of 1O

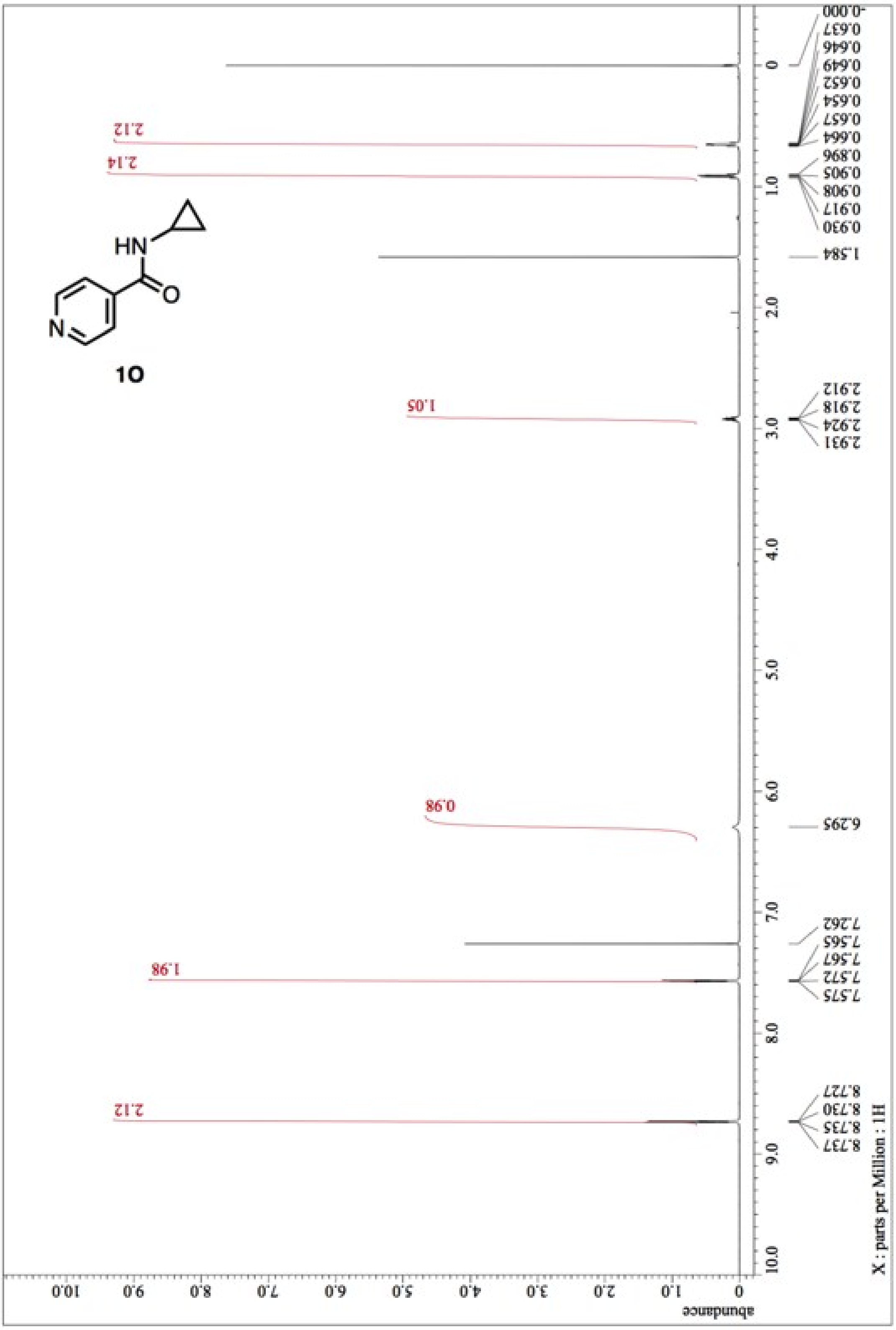

^{13}C NMR (126MHz, CDCl$_3$) of 1O

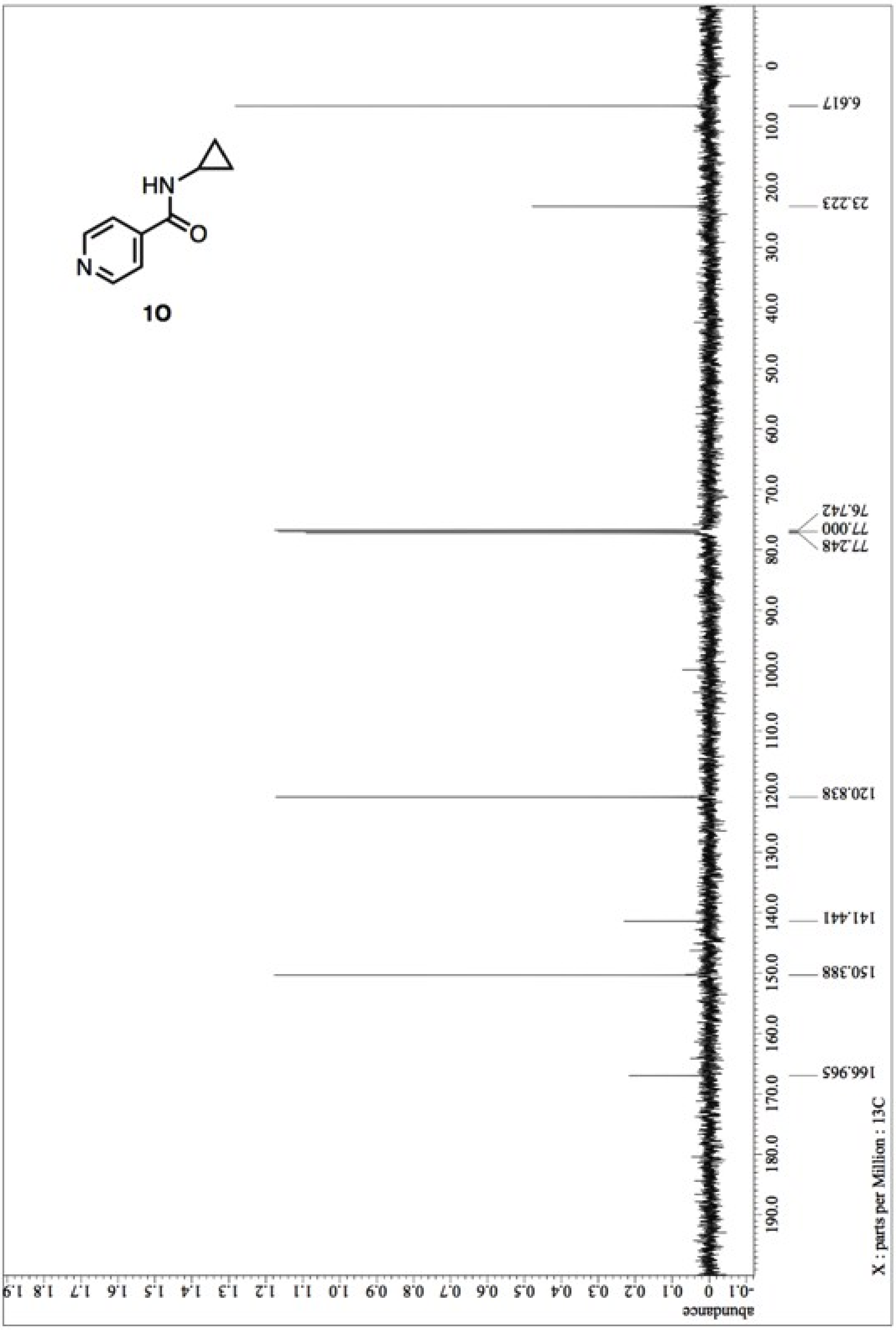

^{1}H NMR (600MHz, $CDCl_3$) of 1P

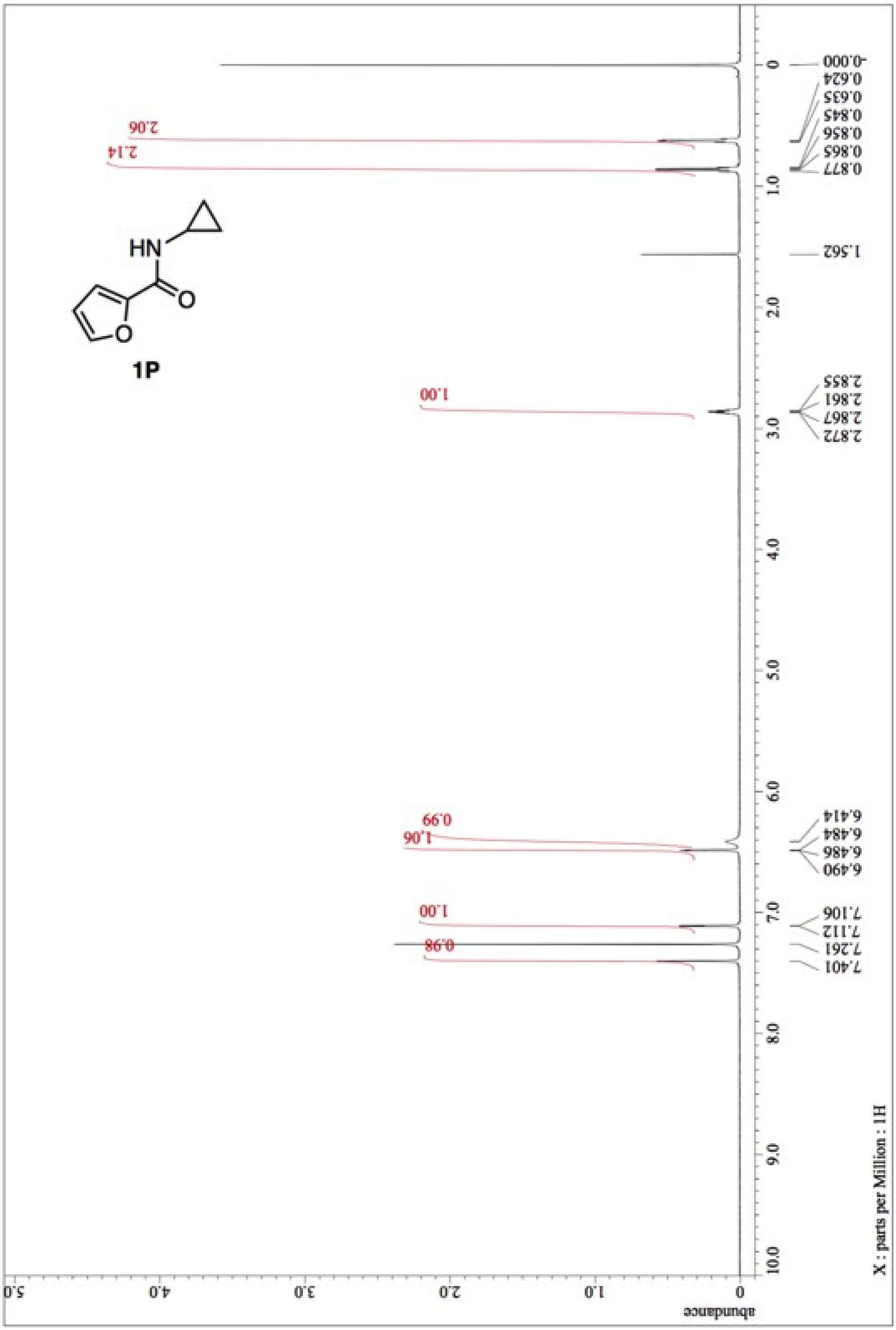

^{13}C NMR (151MHz, $CDCl_3$) of 1P

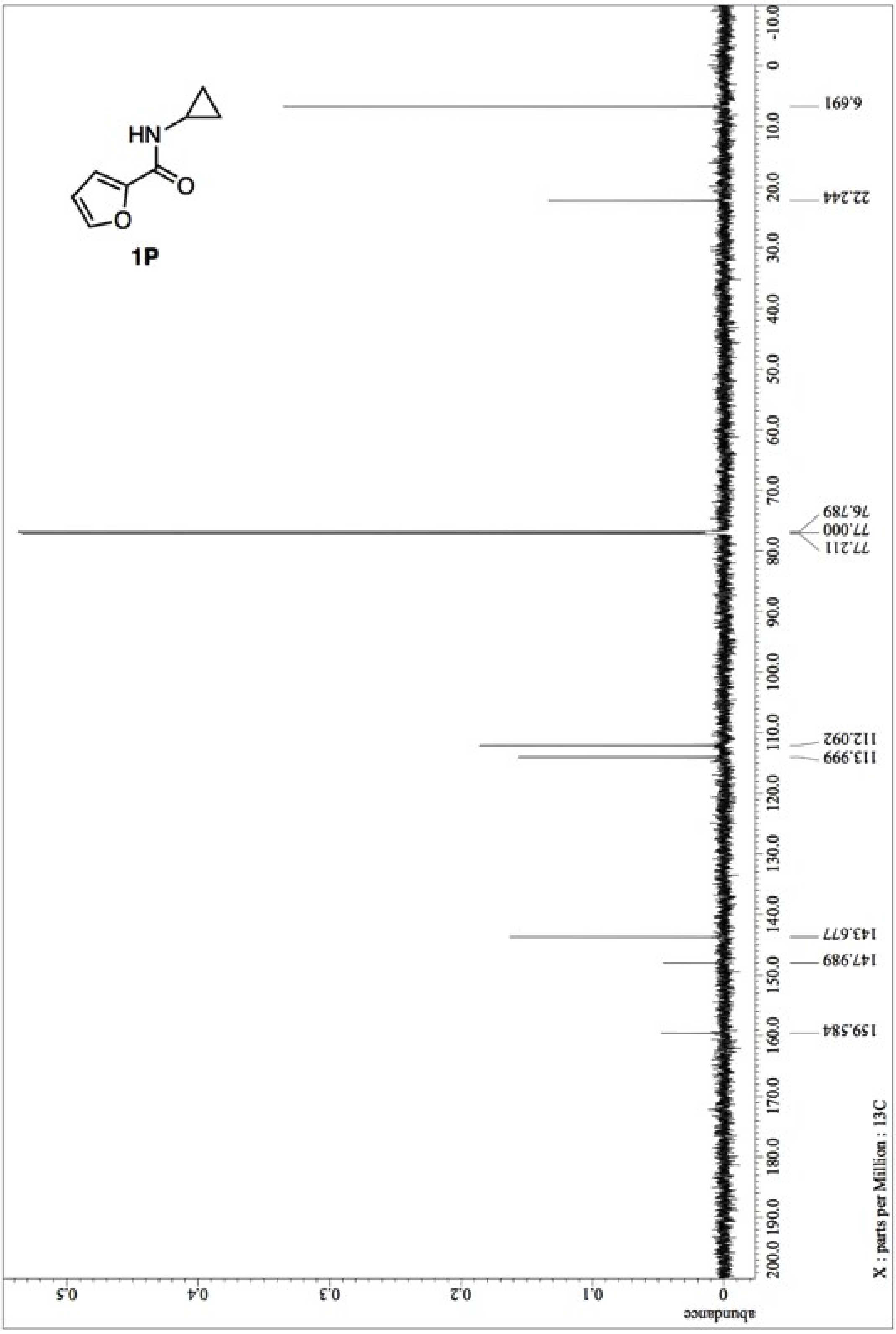

^{1}H NMR (600MHz, $CDCl_3$) of 1Q

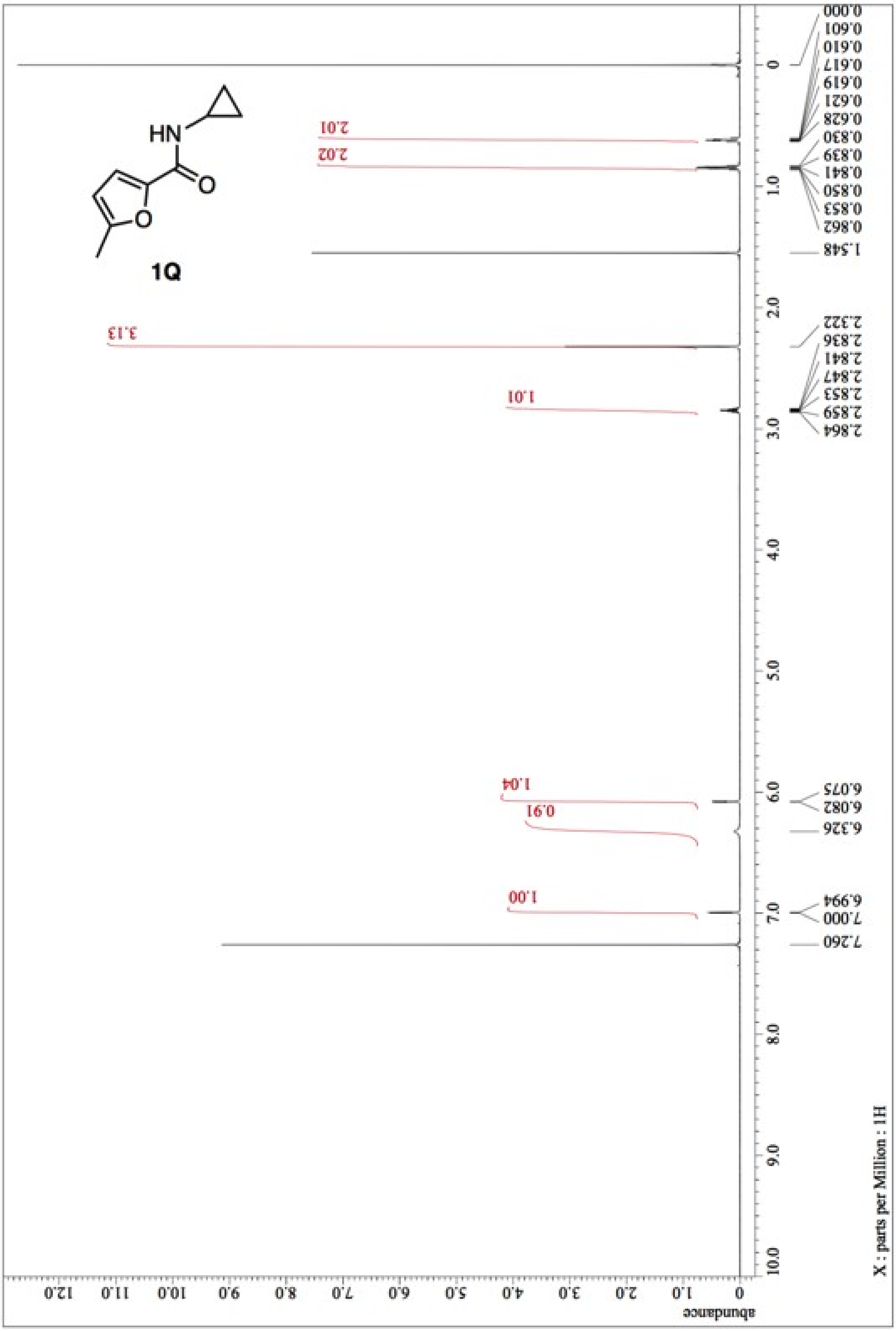

^{13}C NMR (126MHz, CDCl$_3$) of 1Q

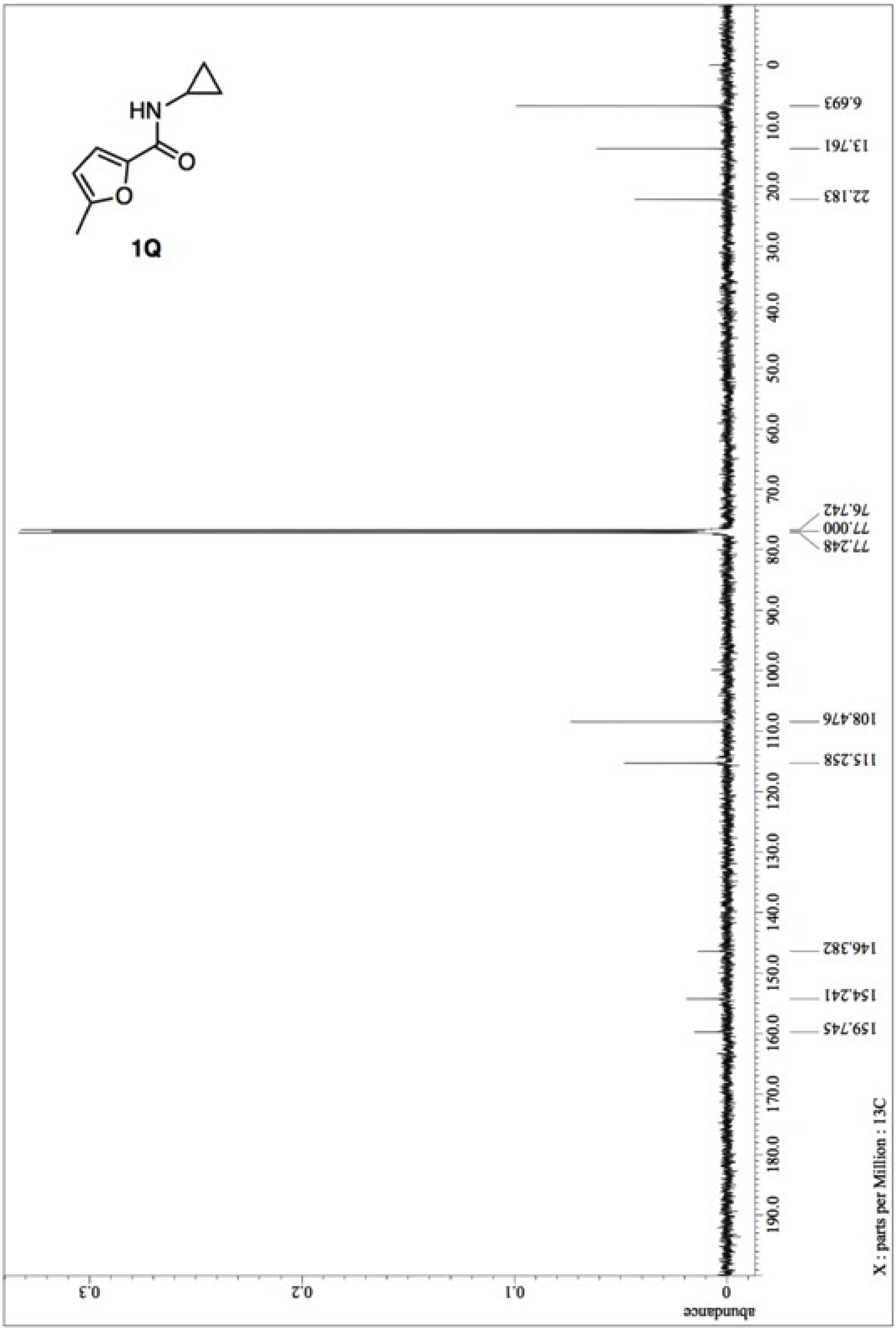

^{1}H NMR (600MHz, $CDCl_3$) of 1R

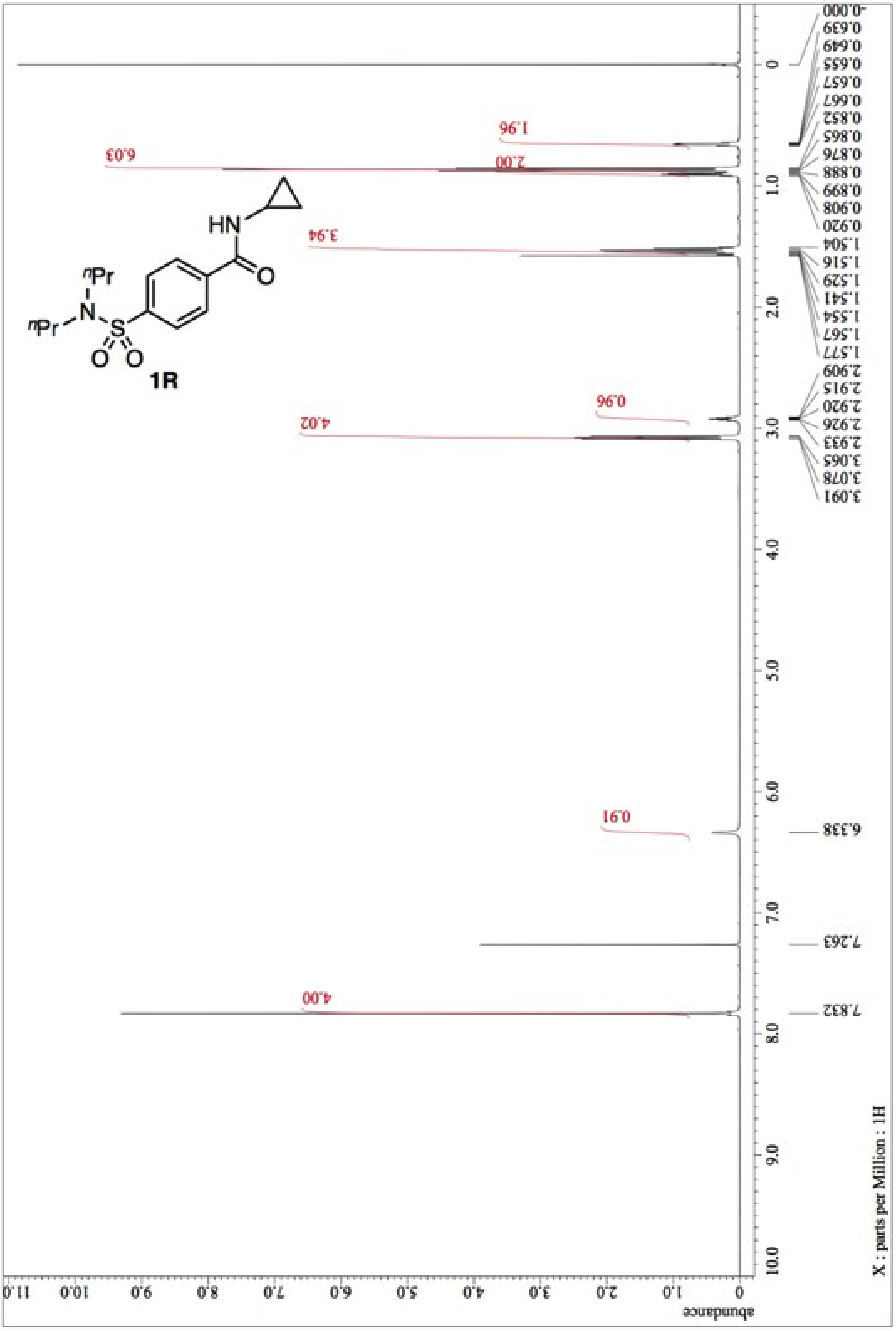

^{13}C NMR (151MHz, $CDCl_3$) of 1R

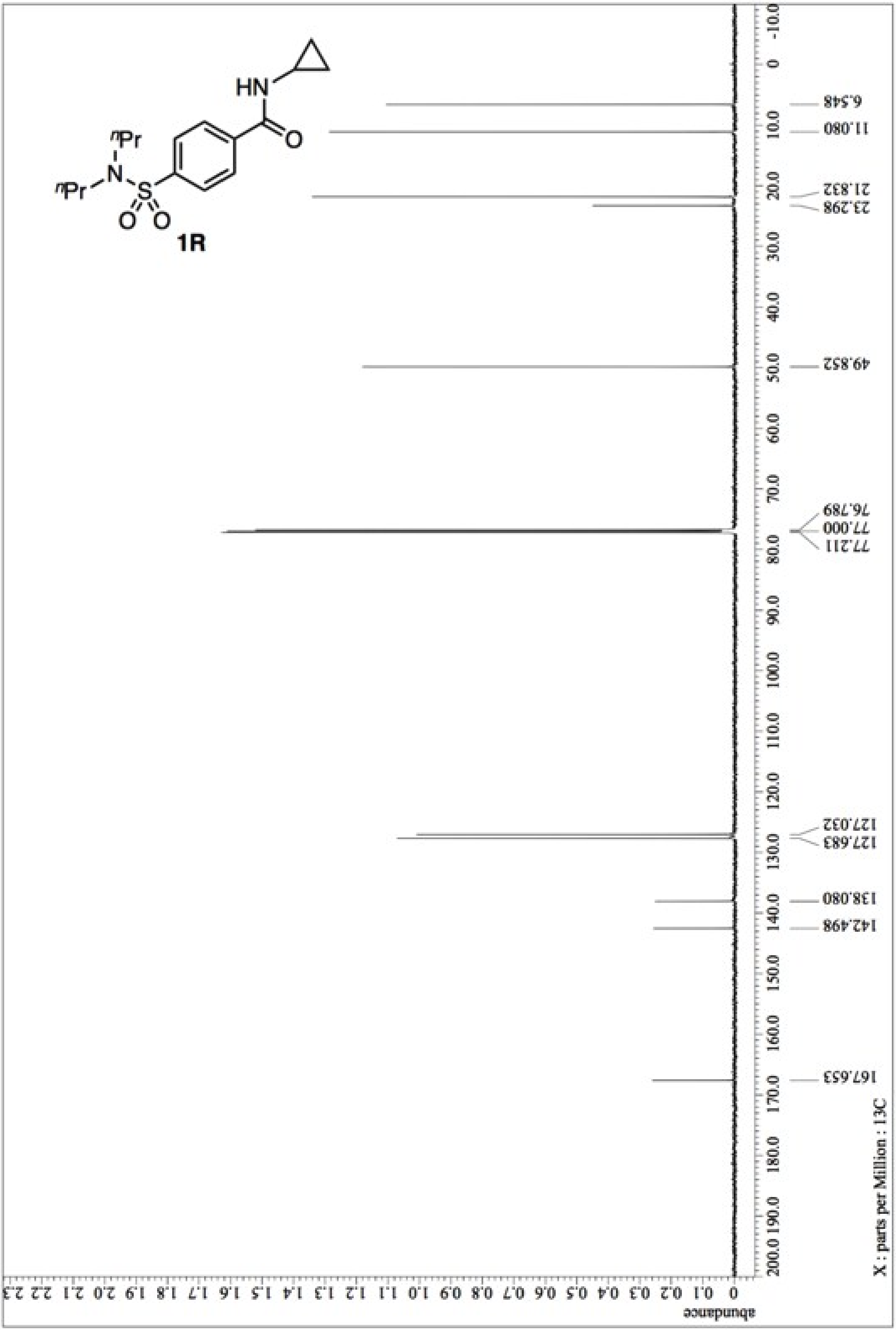

^{1}H NMR (600 MHz, 90 °C, DMSO-d_6) of 1S

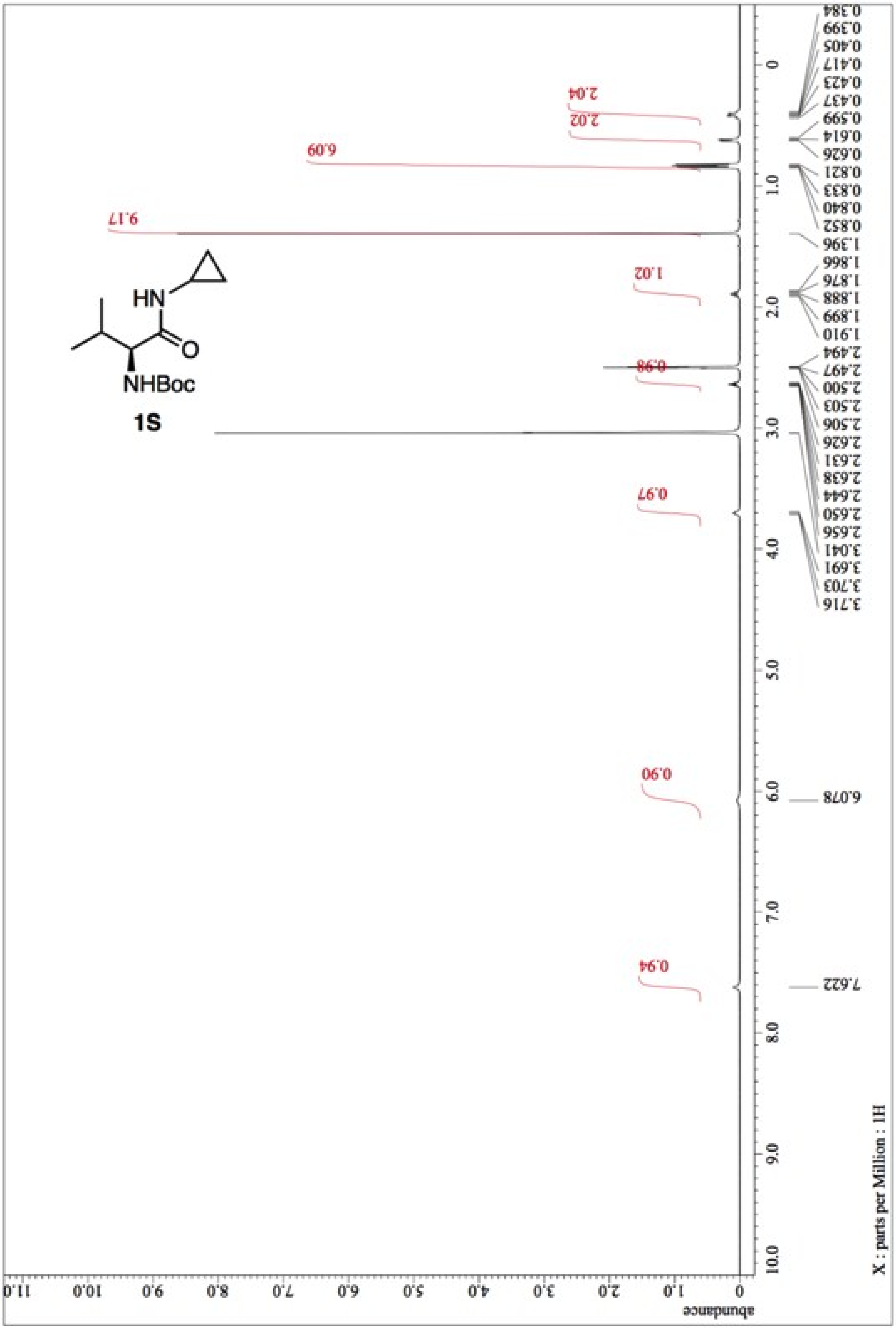

^{13}C NMR (151 MHz, 90 ˚C, DMSO-d_6) of 1S

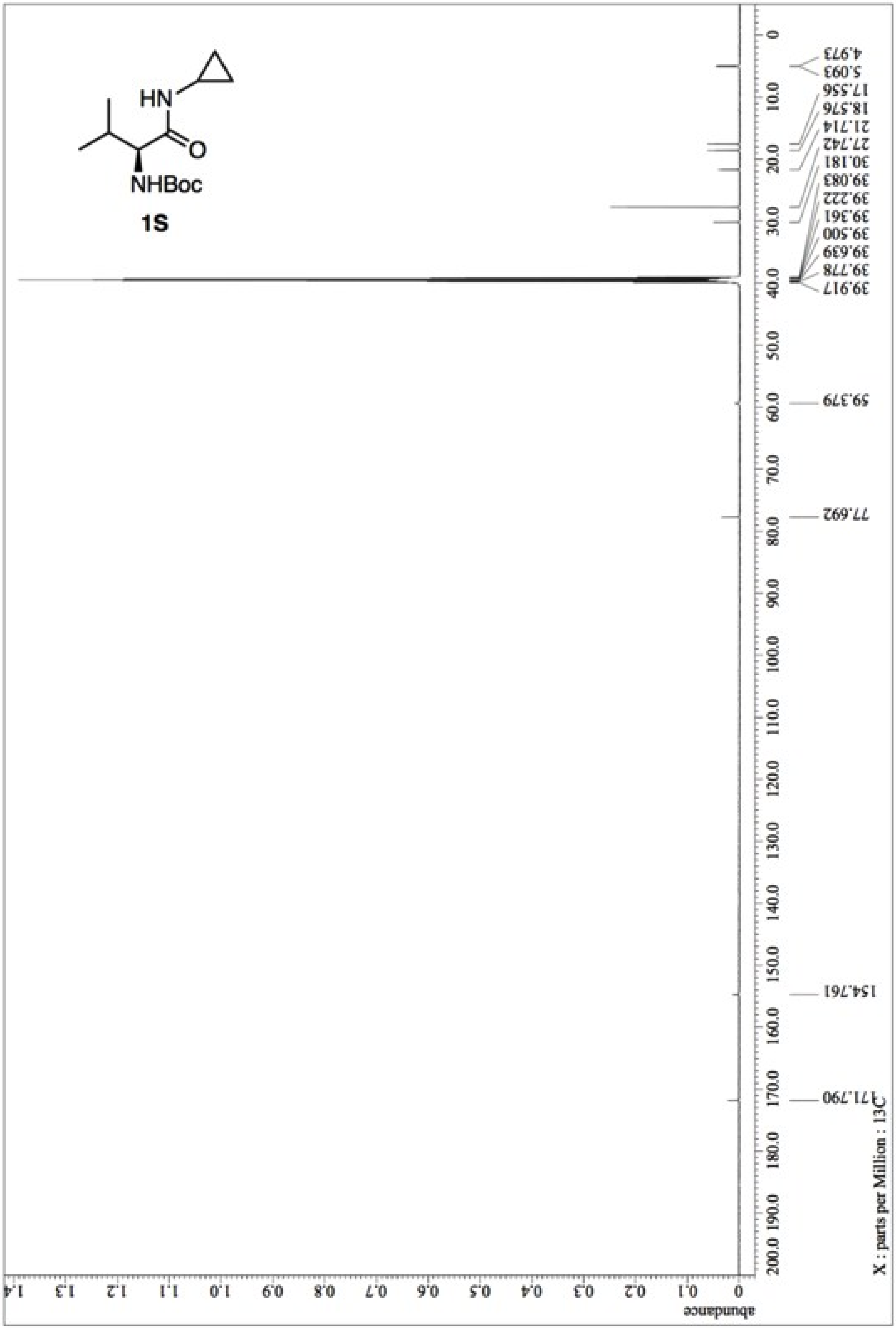

^{1}H NMR (500 MHz, 90 °C, DMSO-d_6) of 1T

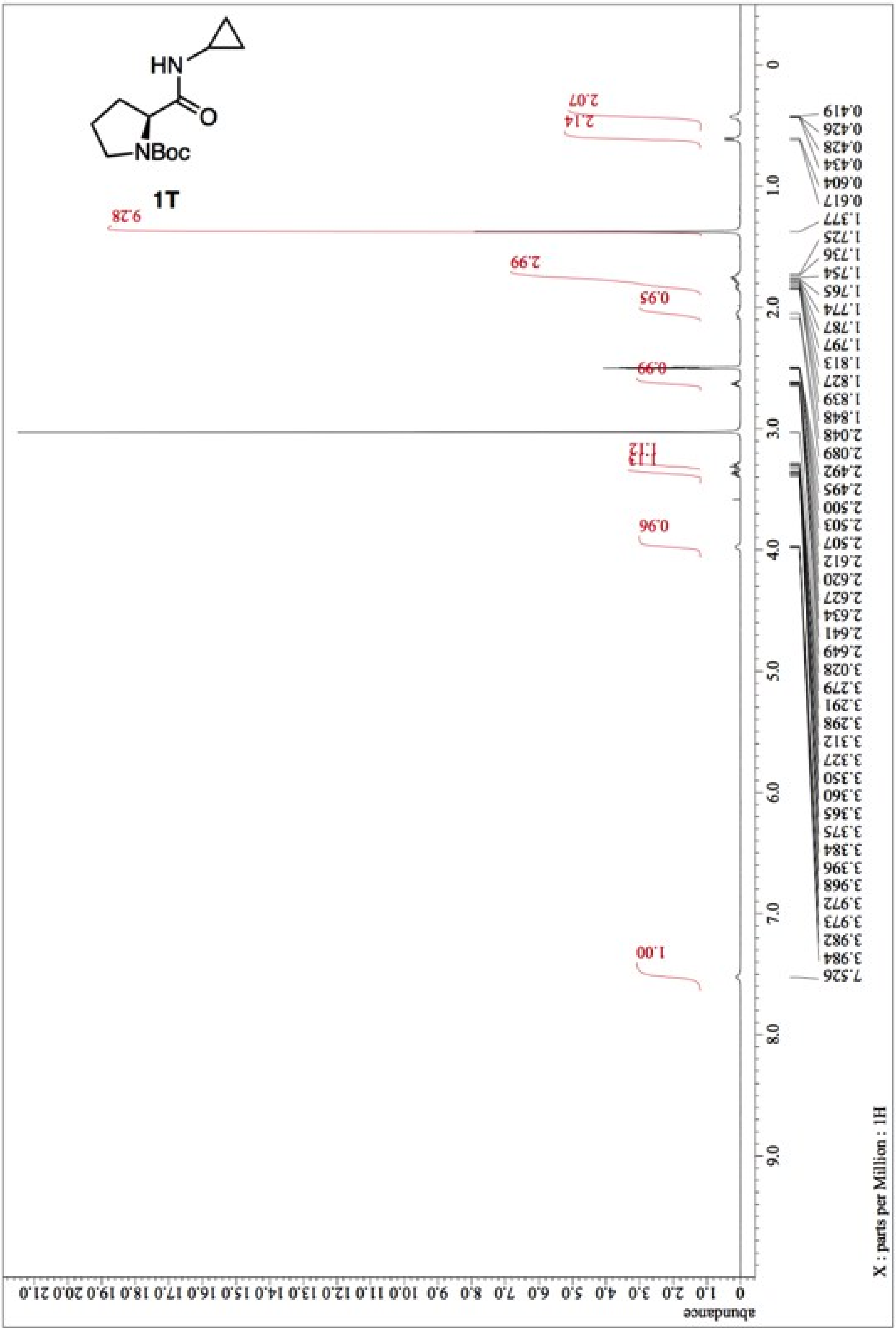

^{13}C NMR (126 MHz, 90 ˚C, DMSO-d_6) of 1T

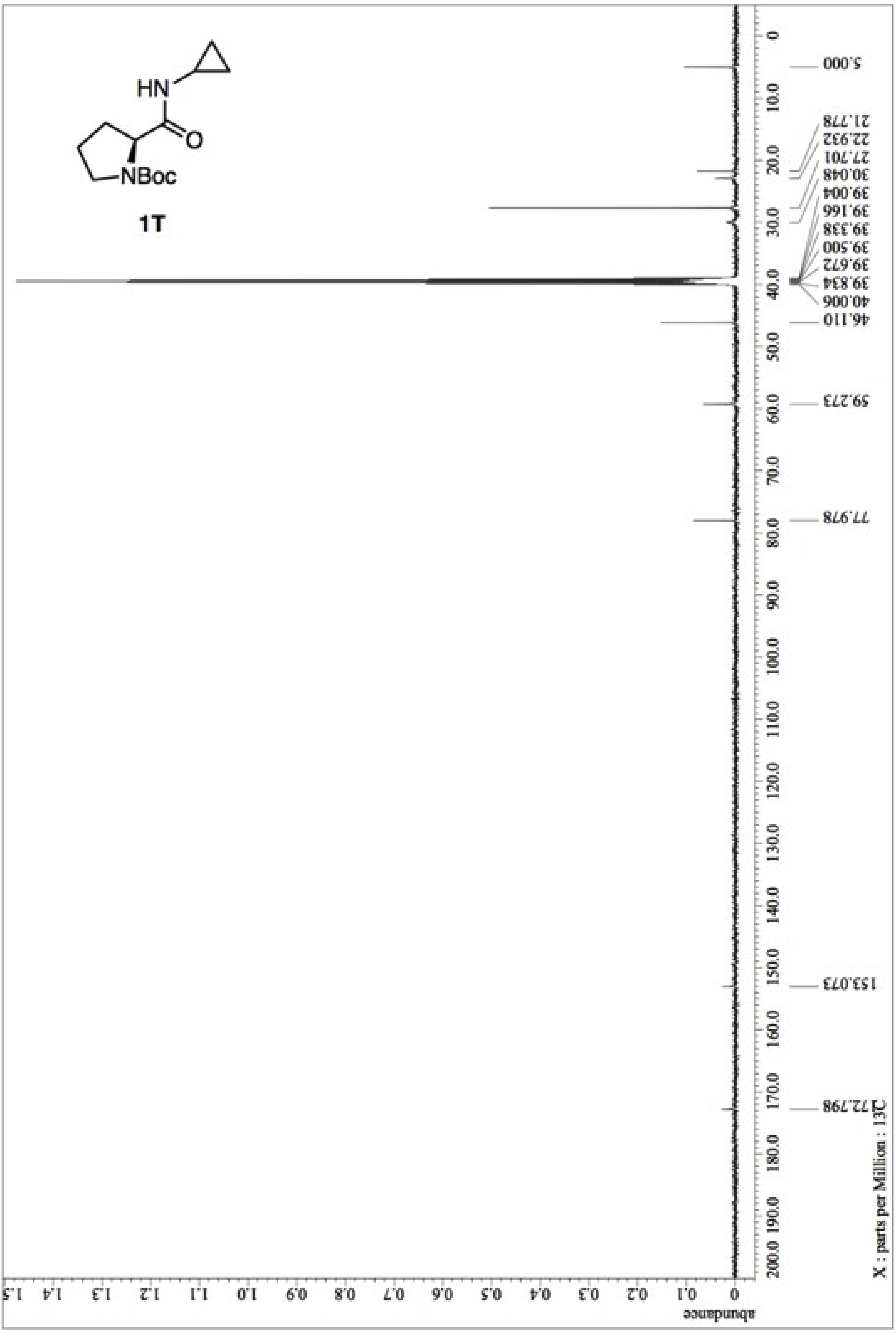

^{1}H NMR (500MHz, $CDCl_3$) of 1U

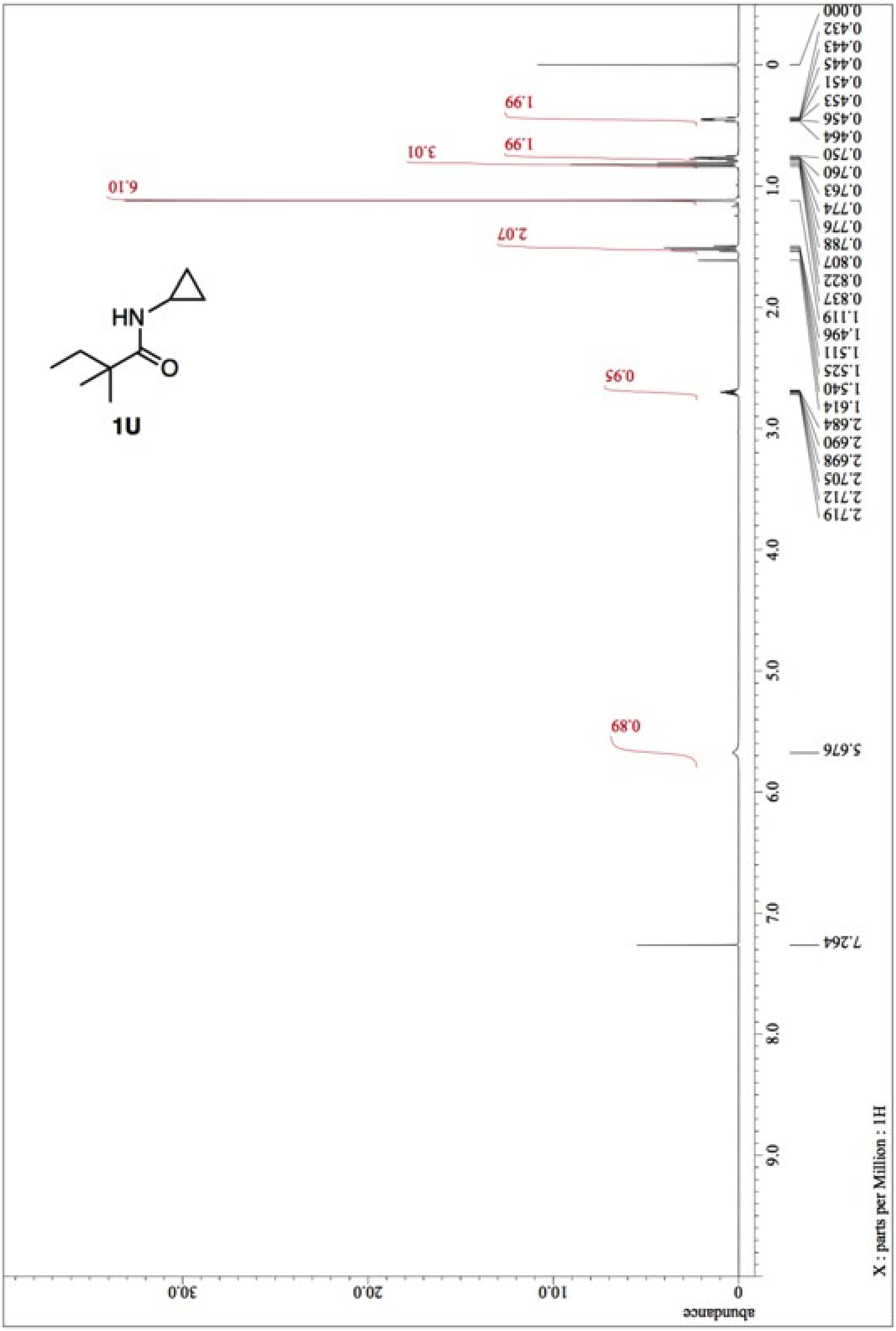

^{13}C NMR (126MHz, $CDCl_3$) of 1U

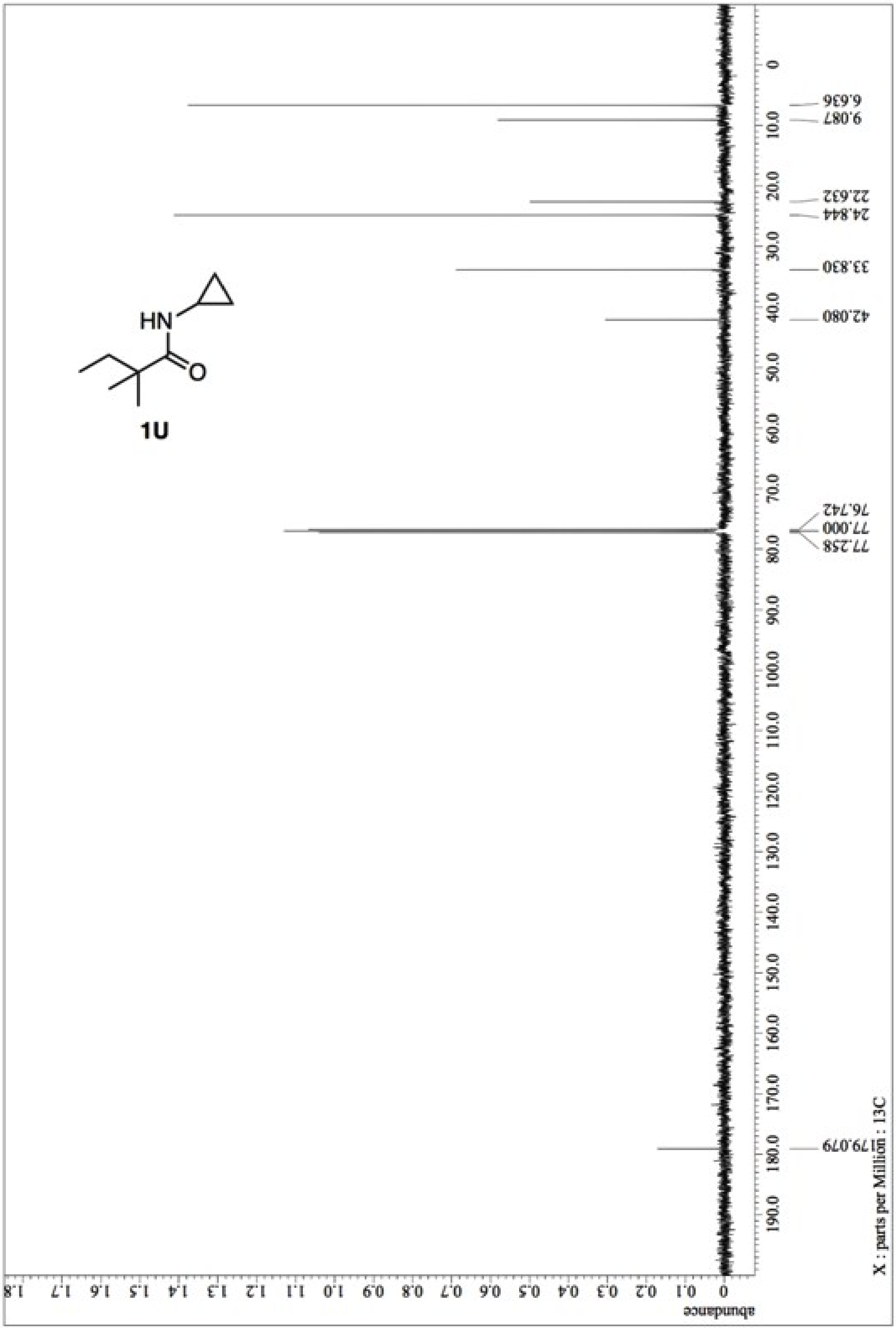

^{1}H NMR (600MHz, $CDCl_3$) of 1V

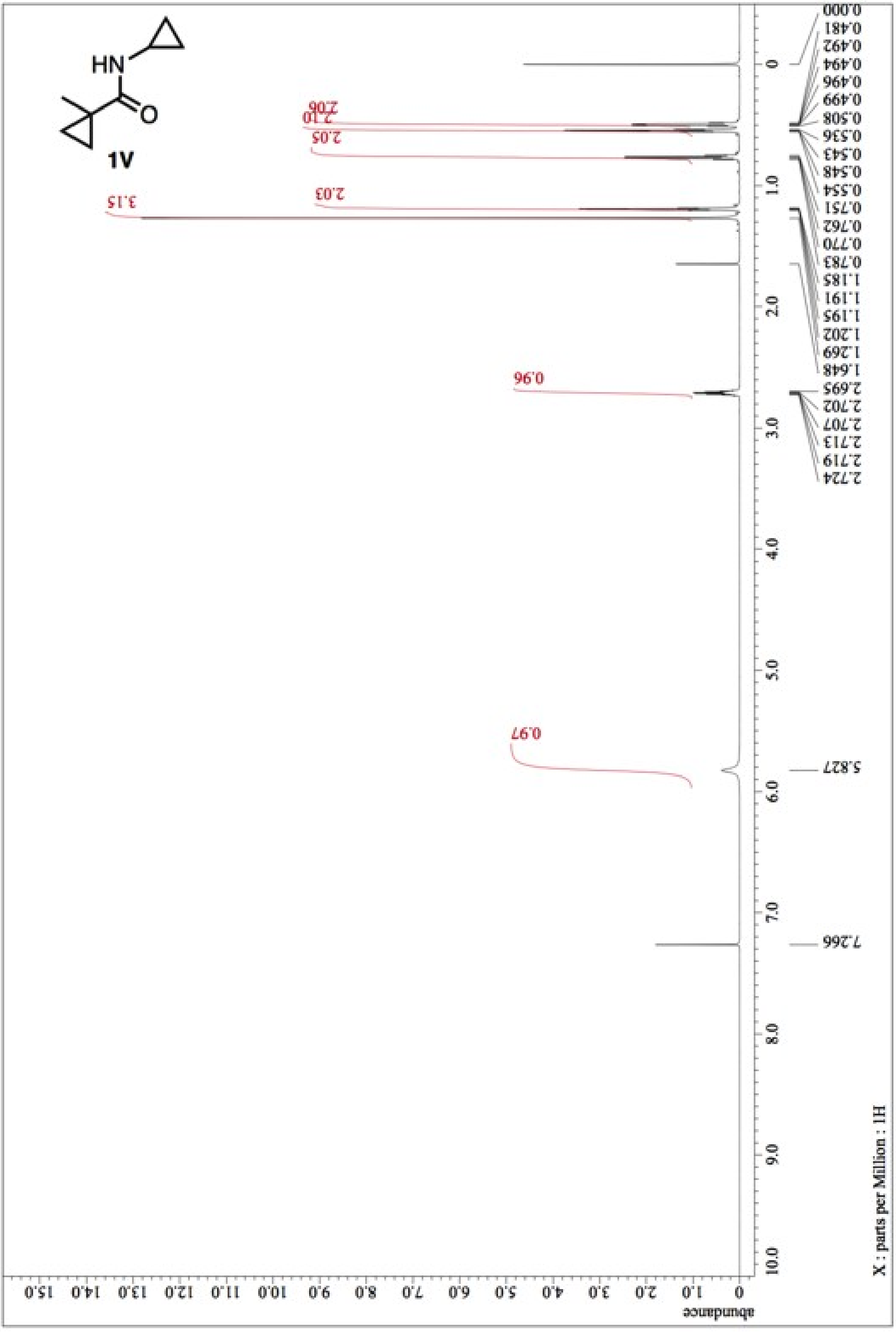

^{13}C NMR (151MHz, $CDCl_3$) of 1V

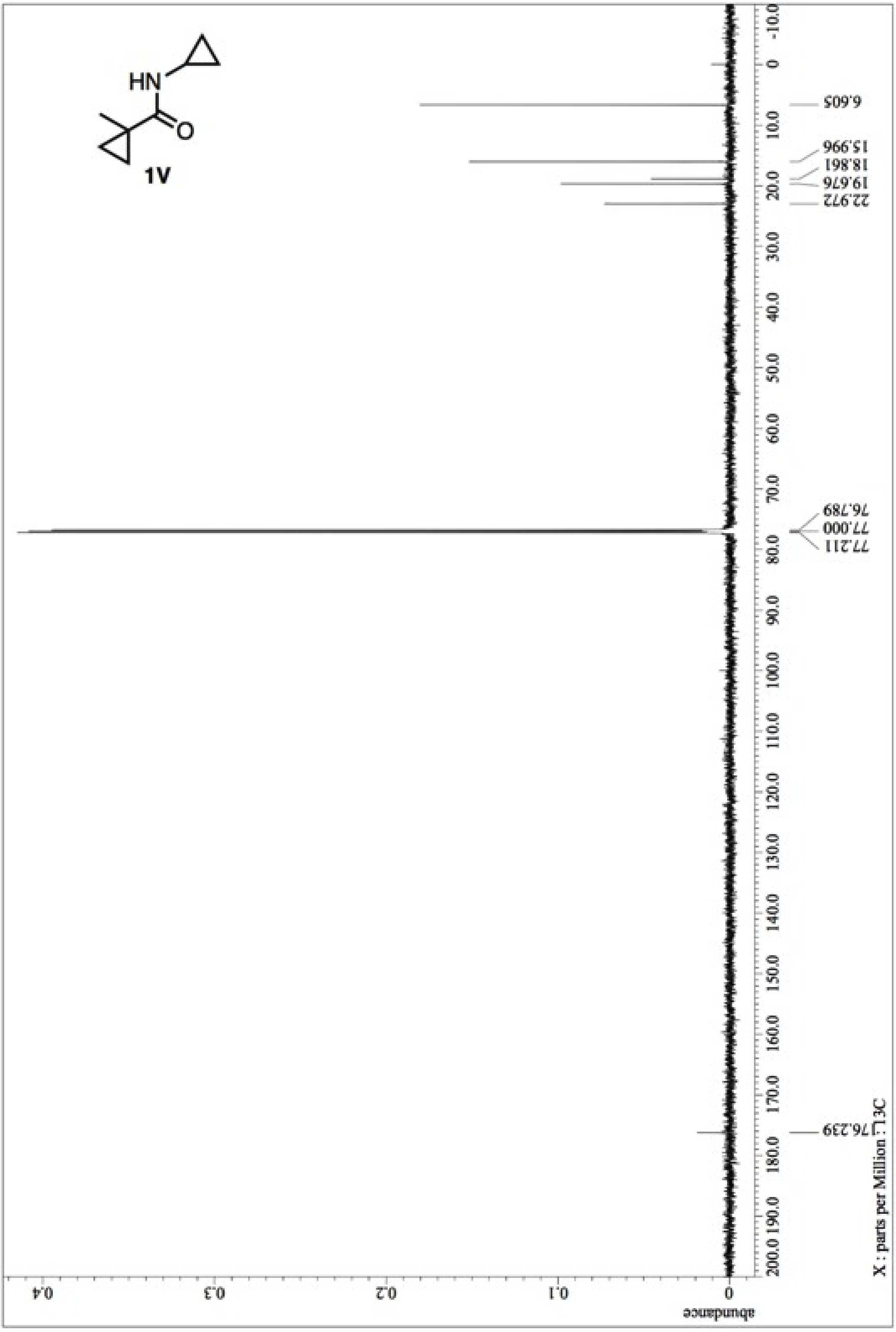

^{1}H NMR (600MHz, $CDCl_3$) of 4Aa

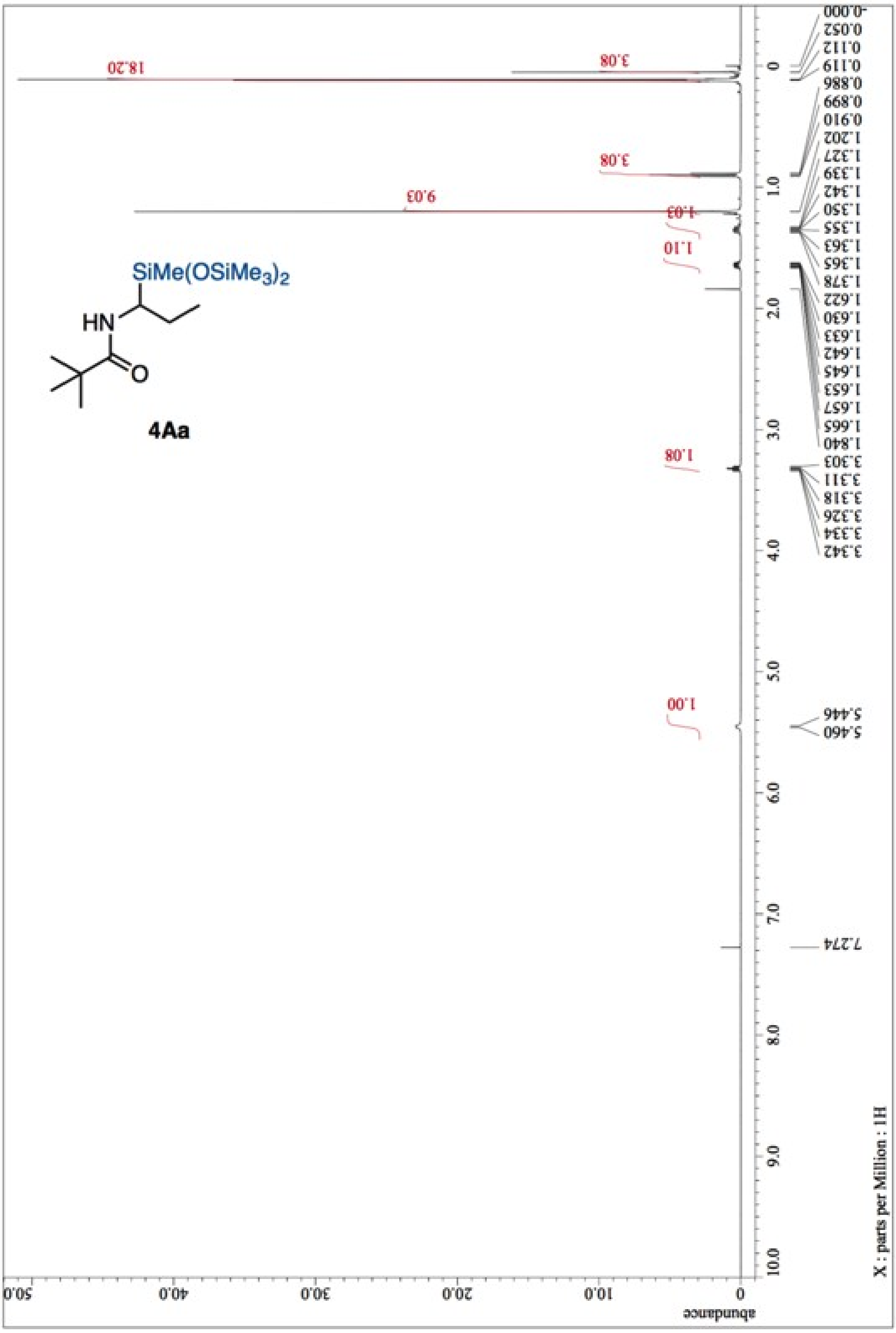

^{13}C NMR (151MHz, $CDCl_3$) of 4Aa

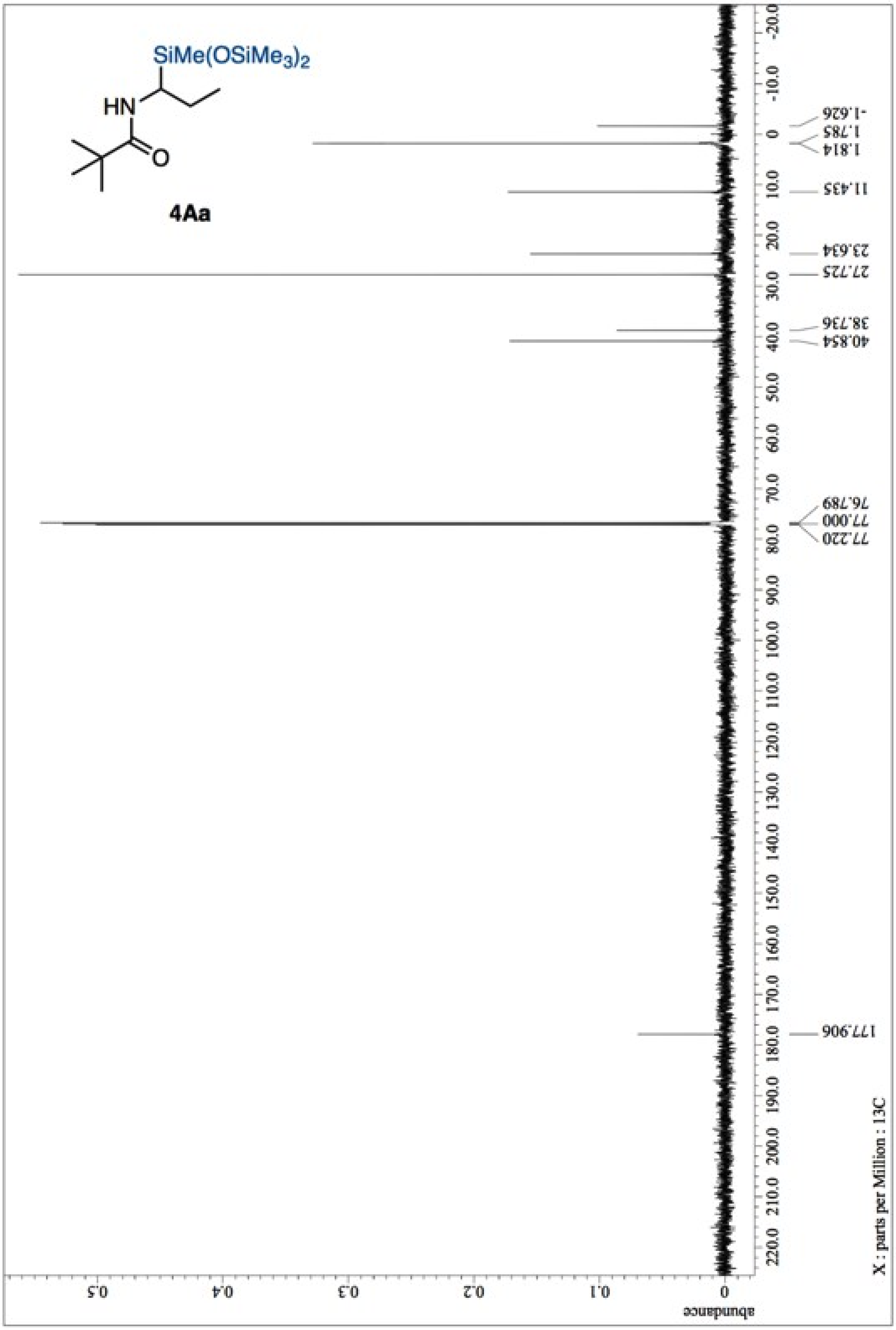

^{1}H NMR (400MHz, $CDCl_3$) of 4Ba

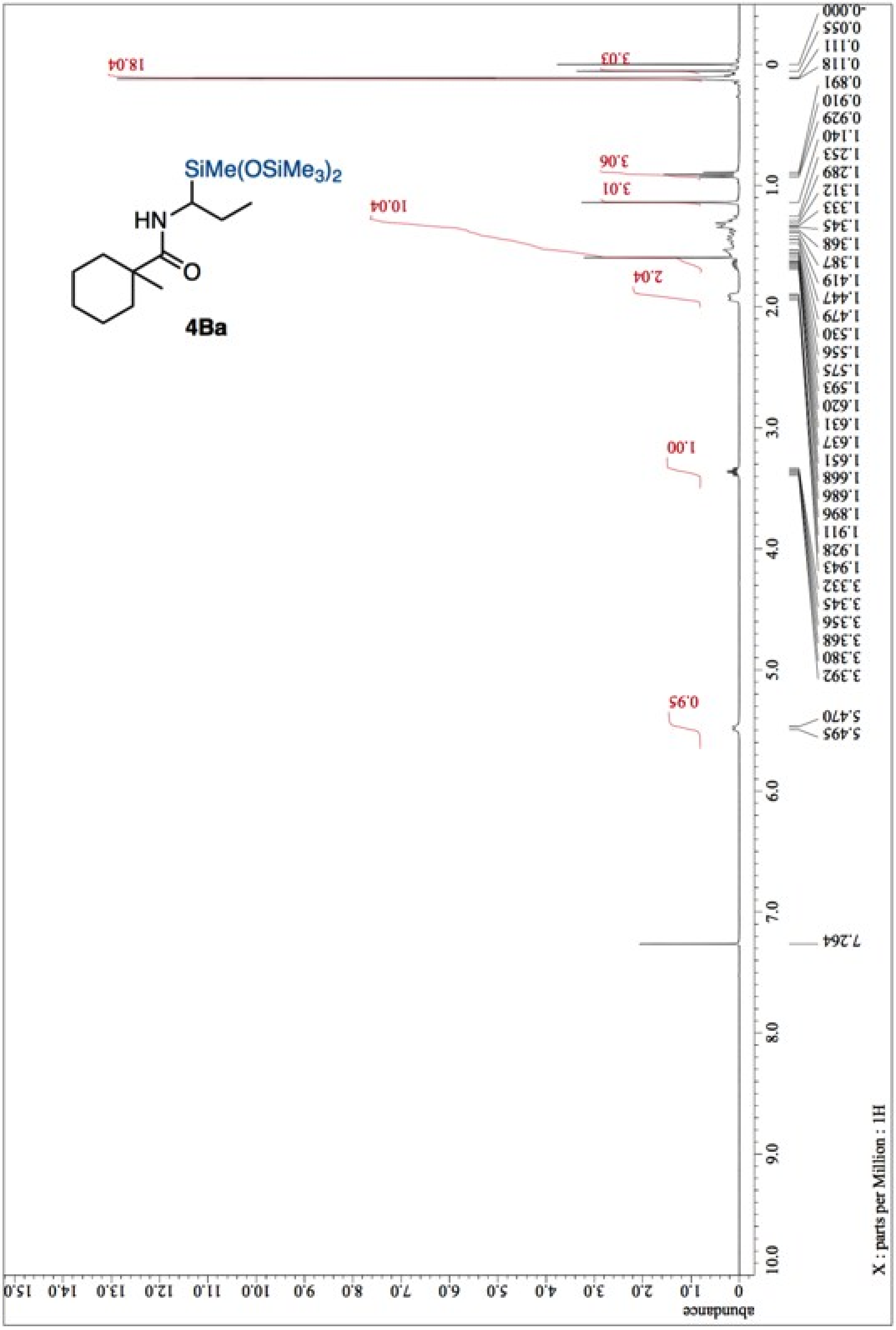

^{13}C NMR (151MHz, $CDCl_3$) of 4Ba

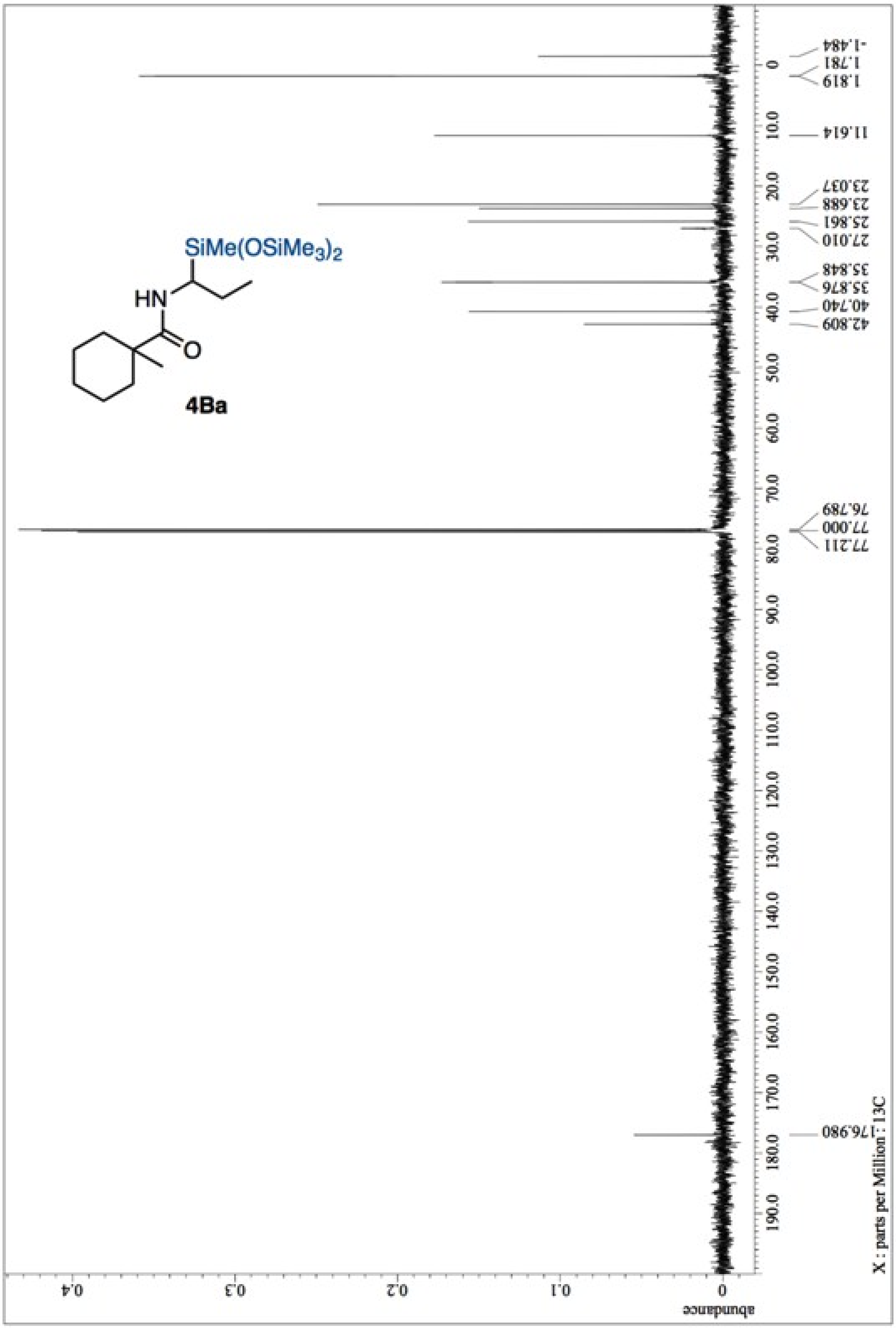

^{1}H NMR (600MHz, $CDCl_3$) of 4Ca

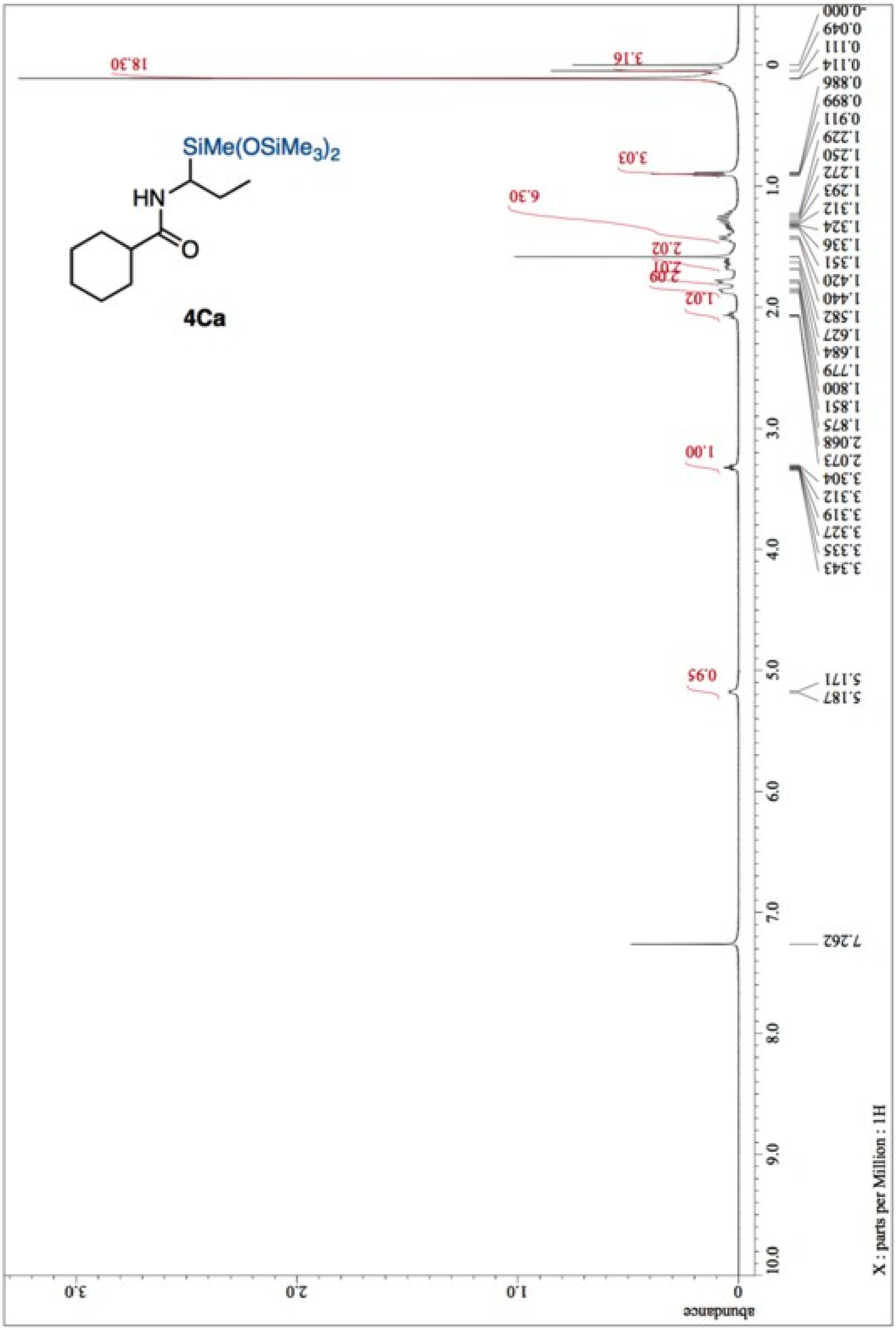

^{13}C NMR (151MHz, $CDCl_3$) of 4Ca

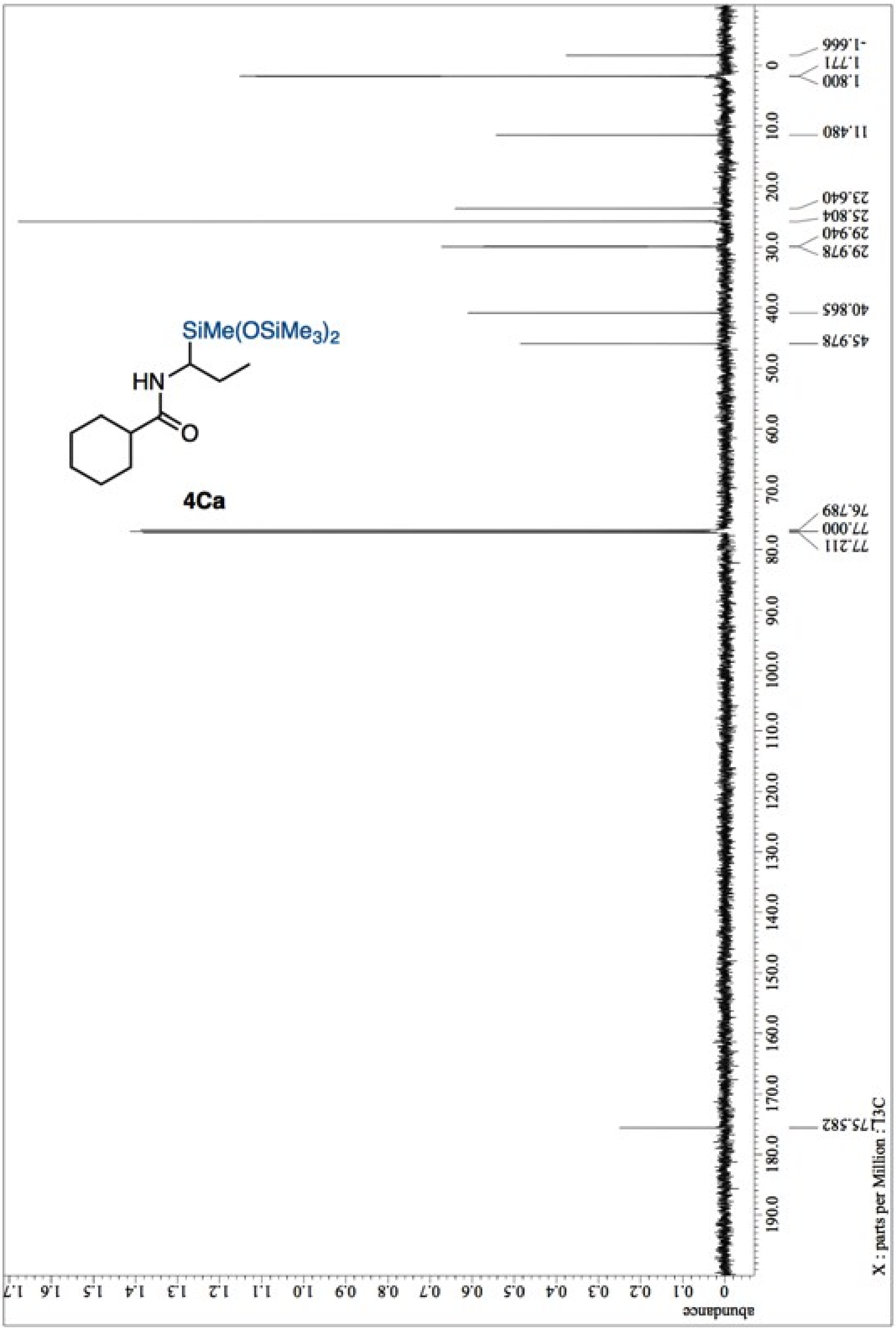

^{1}H NMR (600MHz, $CDCl_3$) of 4Da

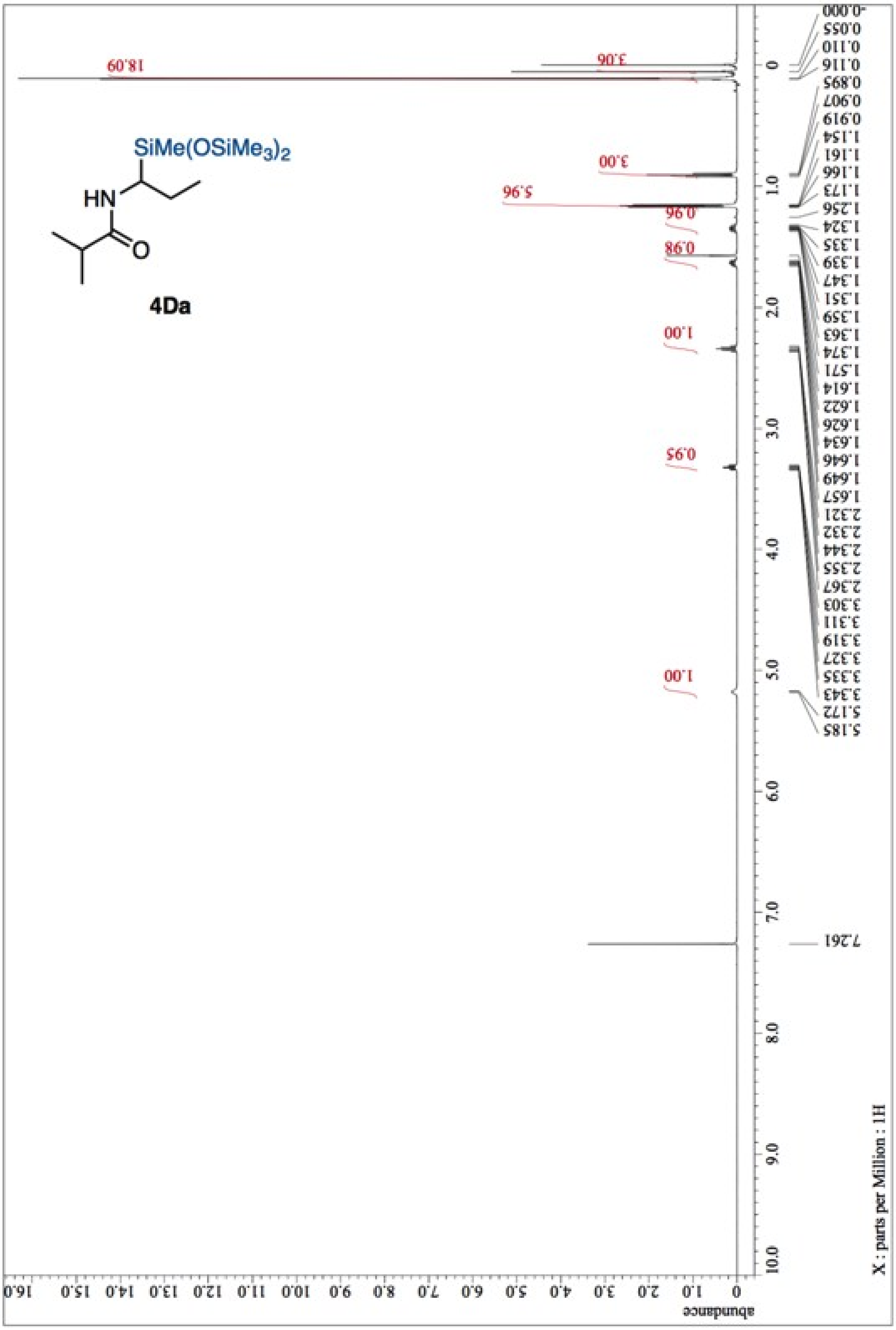

^{13}C NMR (151MHz, CDCl$_3$) of 4Da

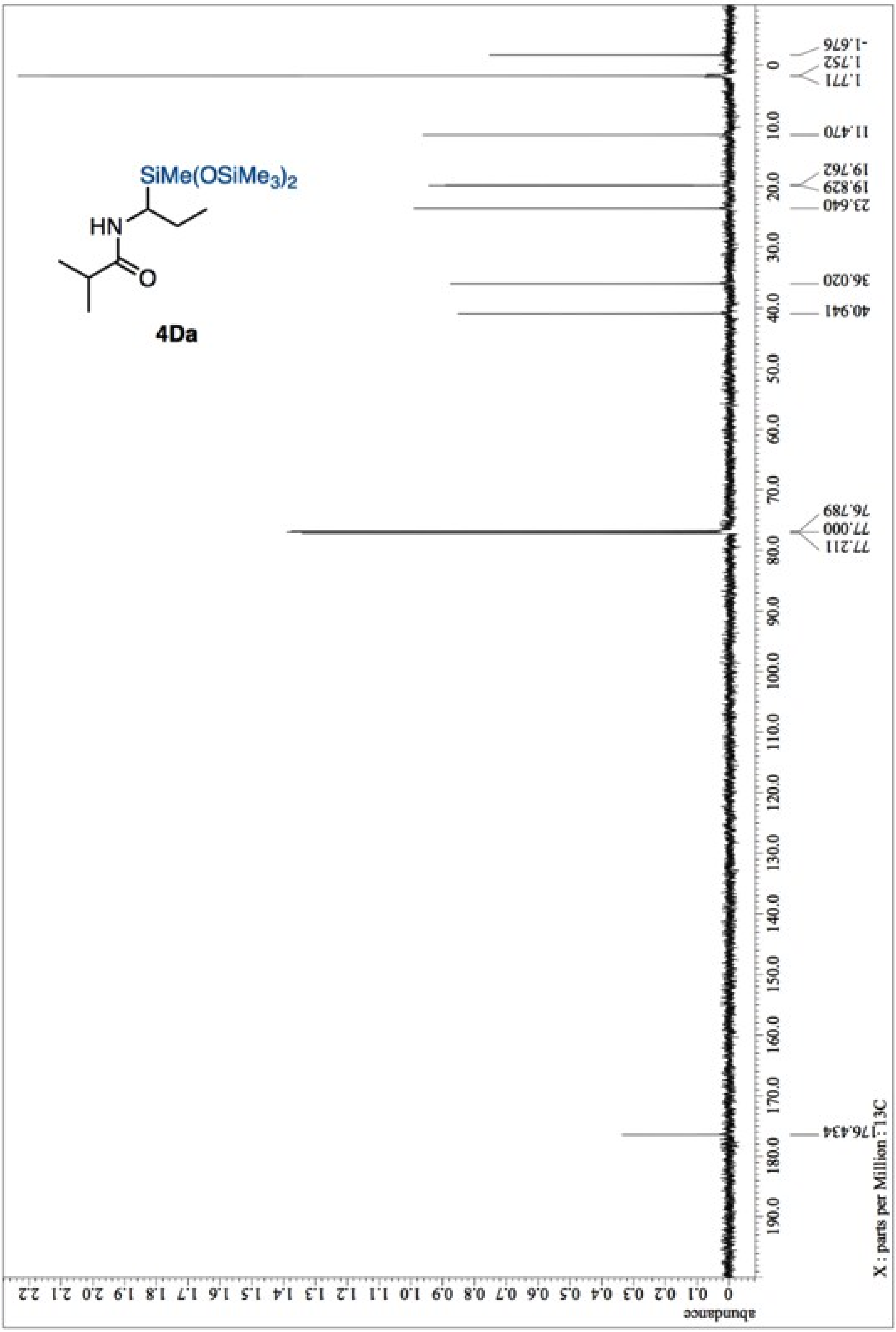

^{1}H NMR (400MHz, $CDCl_3$) of 4Ea

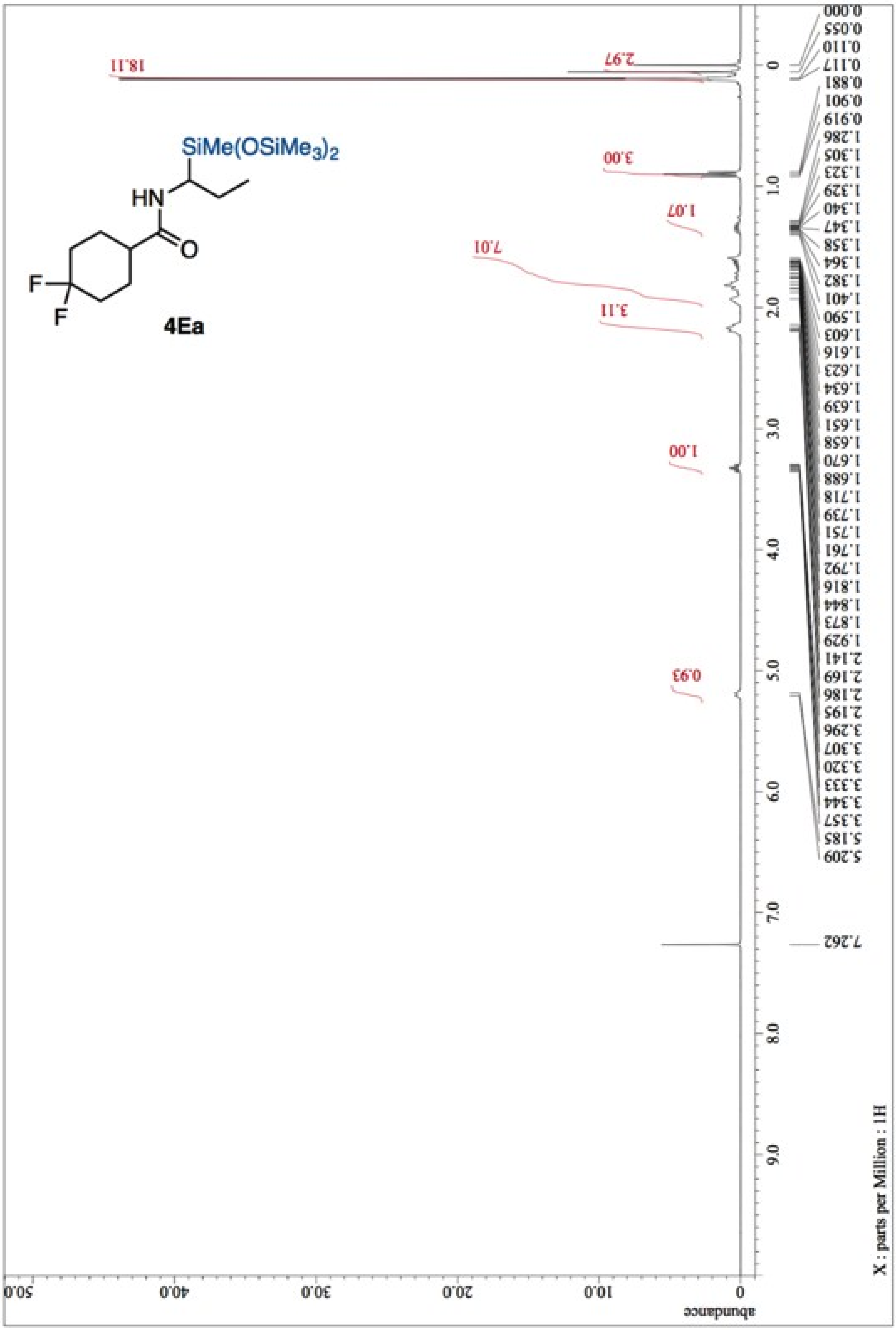

^{13}C NMR (101MHz, $CDCl_3$) of 4Ea

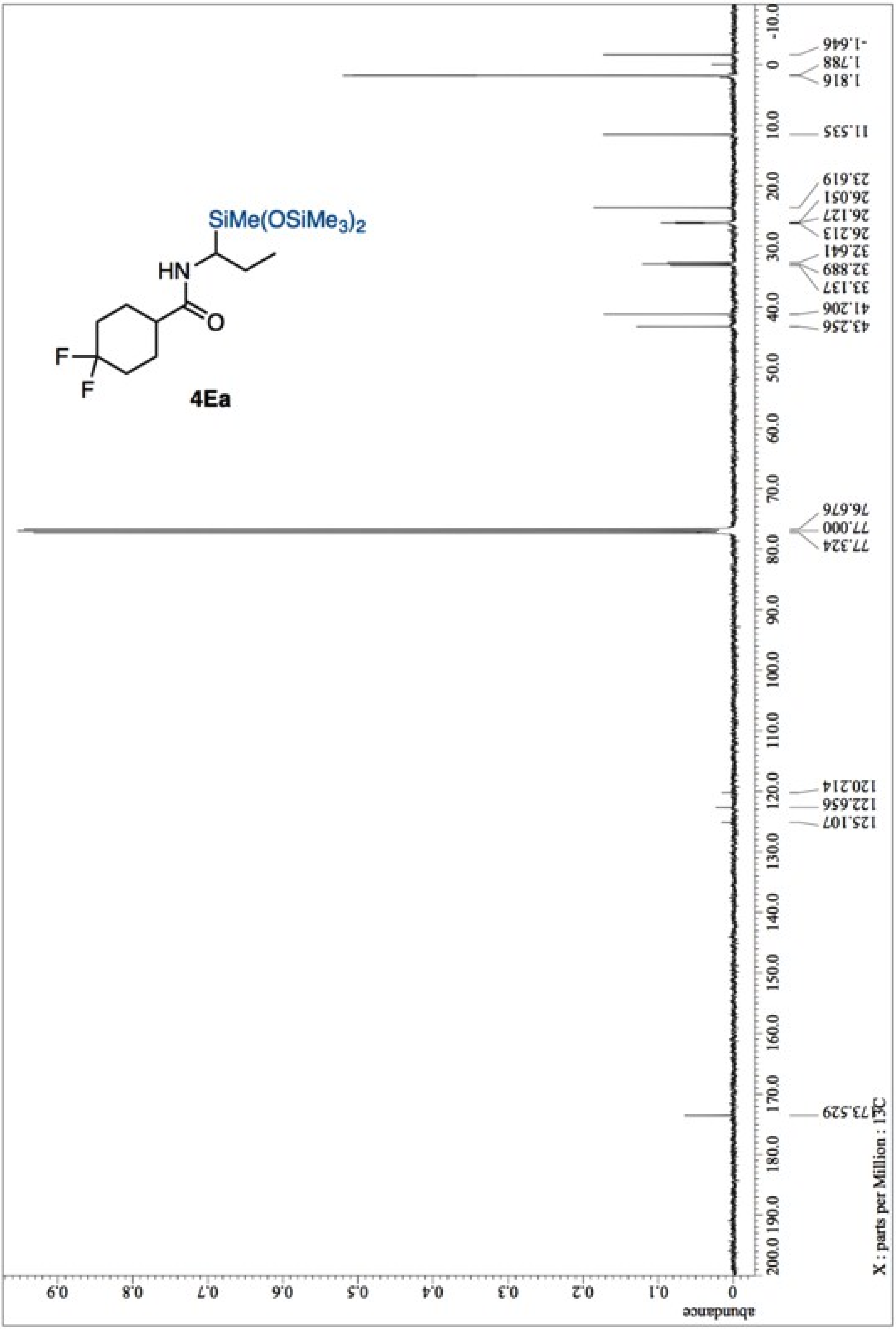

^{1}H NMR (600MHz, $CDCl_3$) of 4Fa

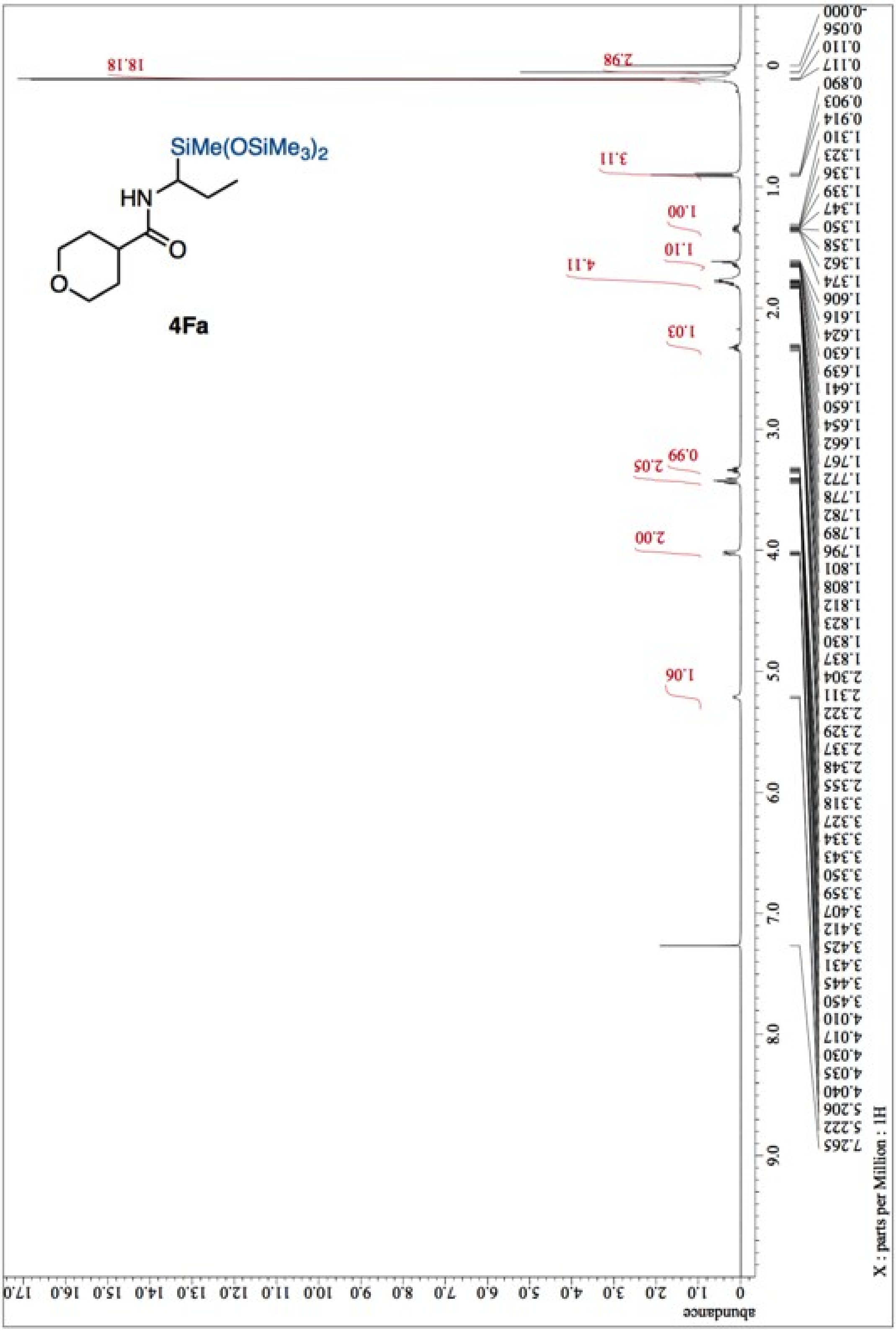

^{13}C NMR (151MHz, $CDCl_3$) of 4Fa

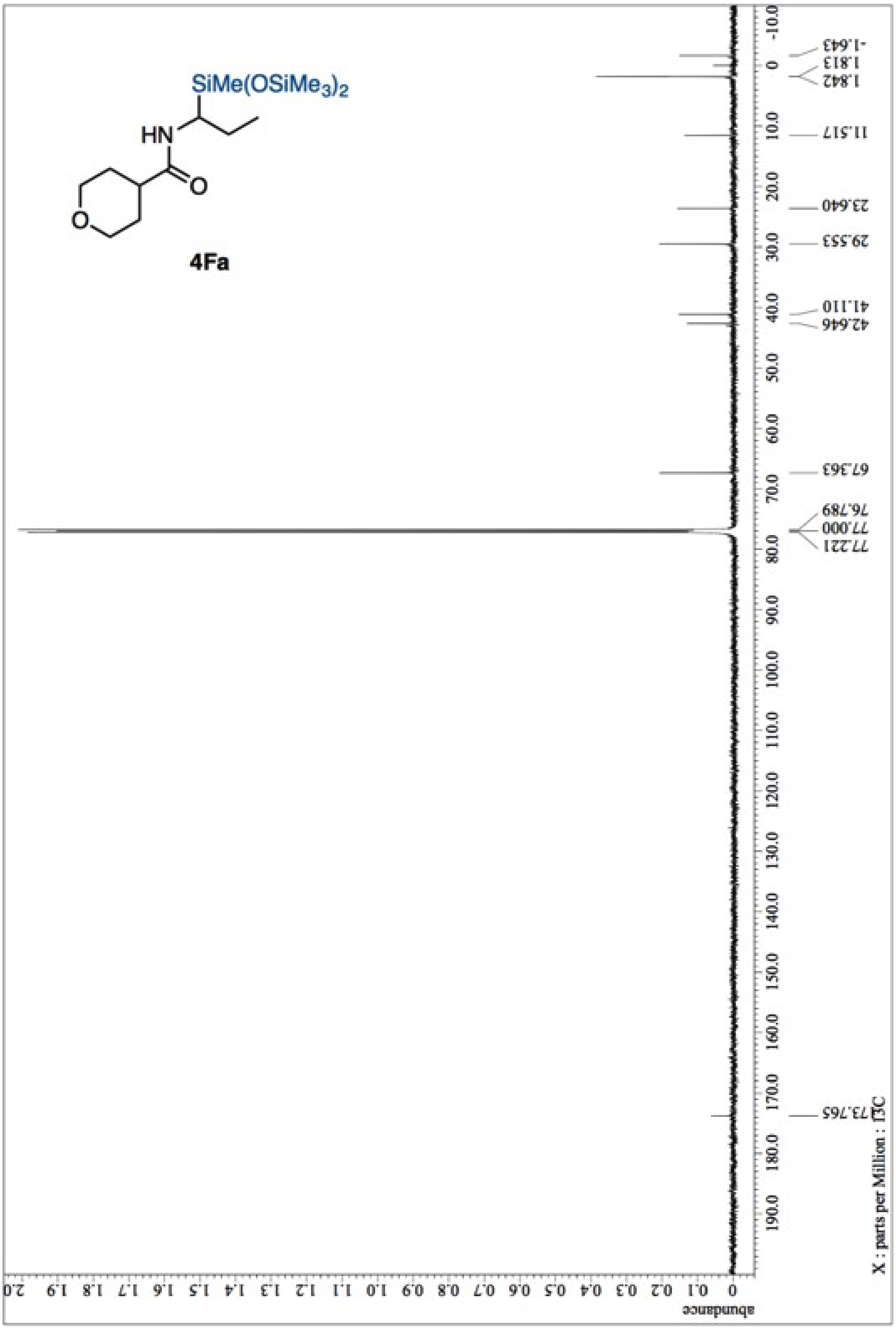

^{1}H NMR (600 MHz, 120 °C, DMSO-d_6) of 4Ga

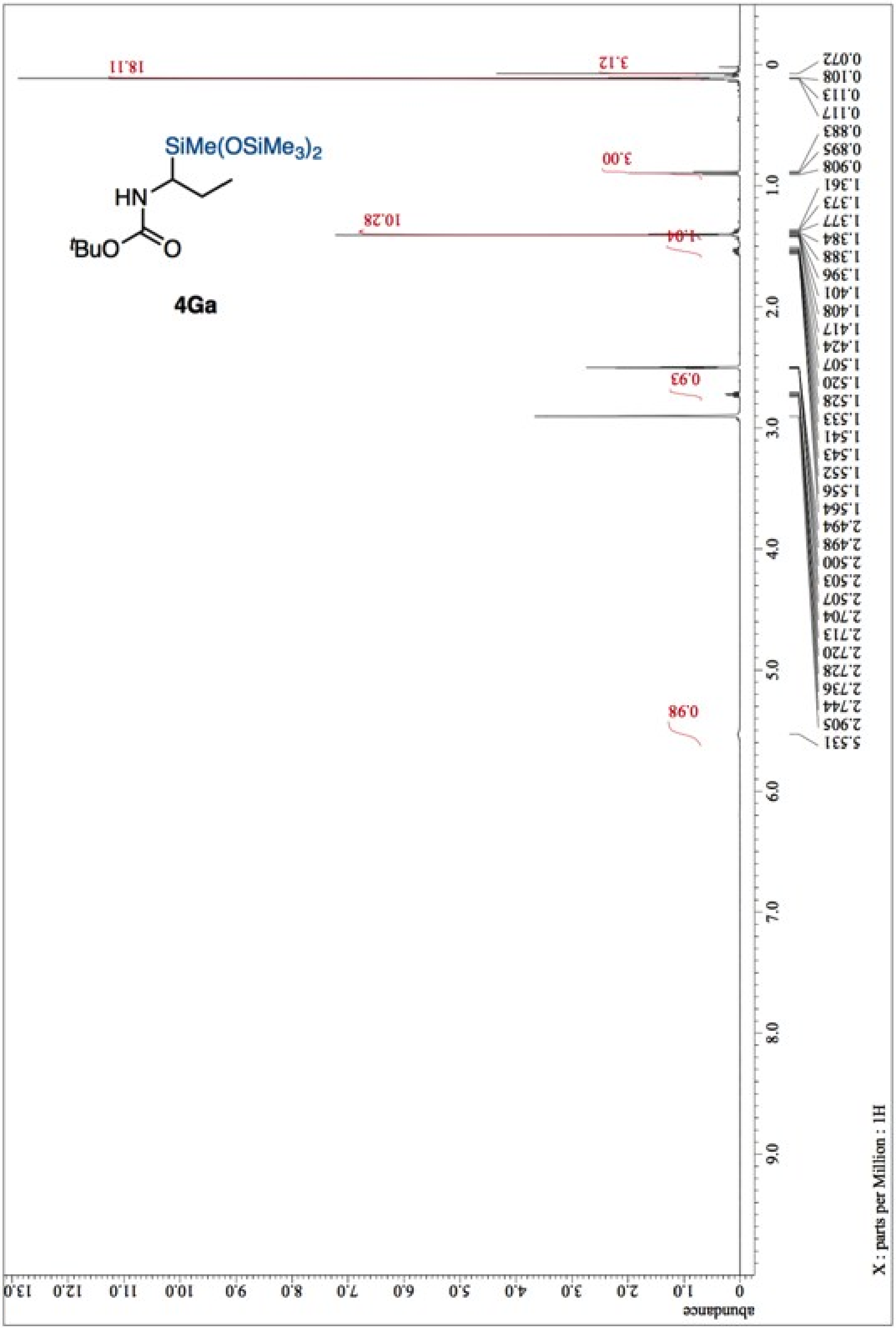

^{13}C NMR (151 MHz, 120 °C, DMSO-d_6) of 4Ga

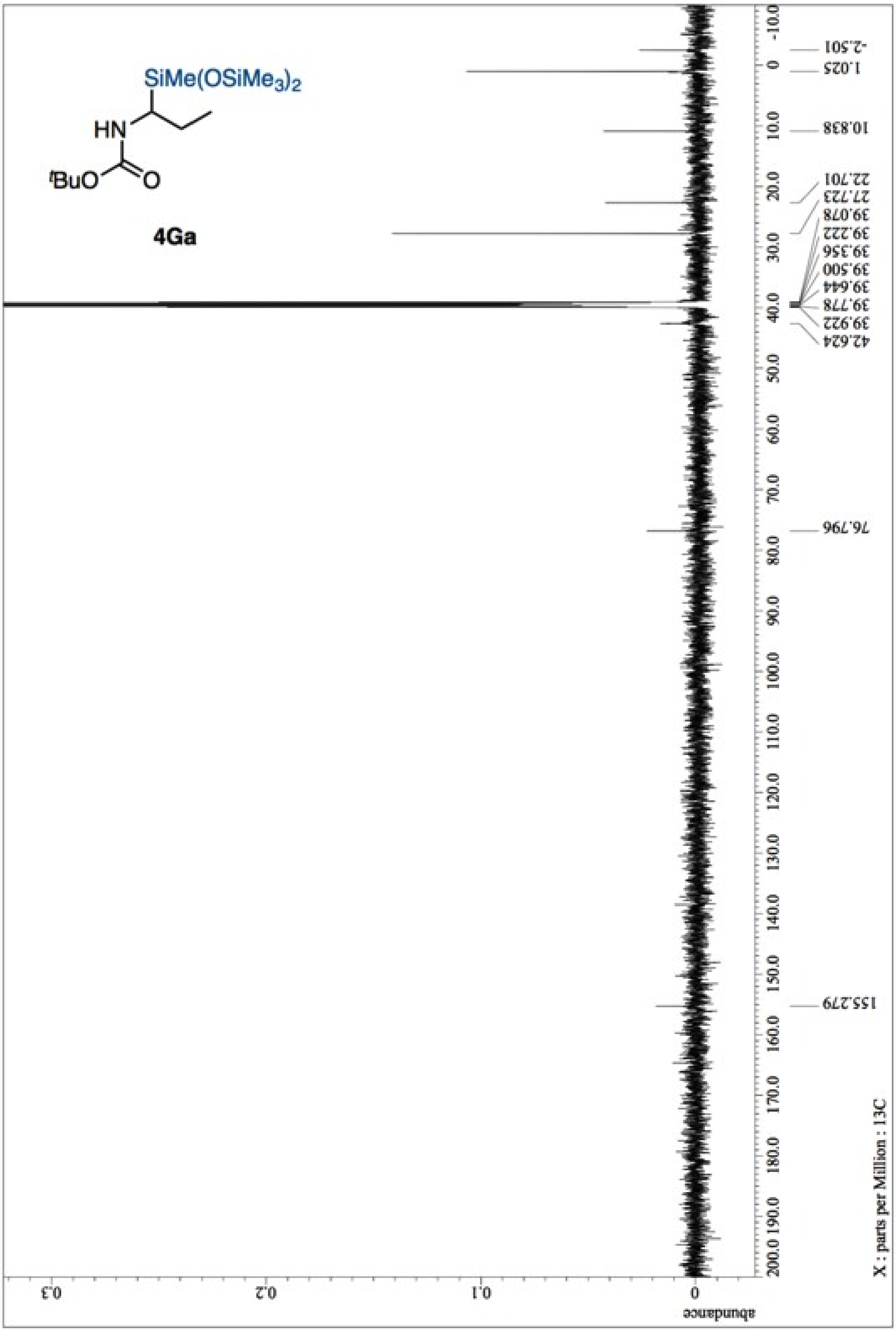

^{1}H NMR (600MHz, $CDCl_3$) of 4Ha

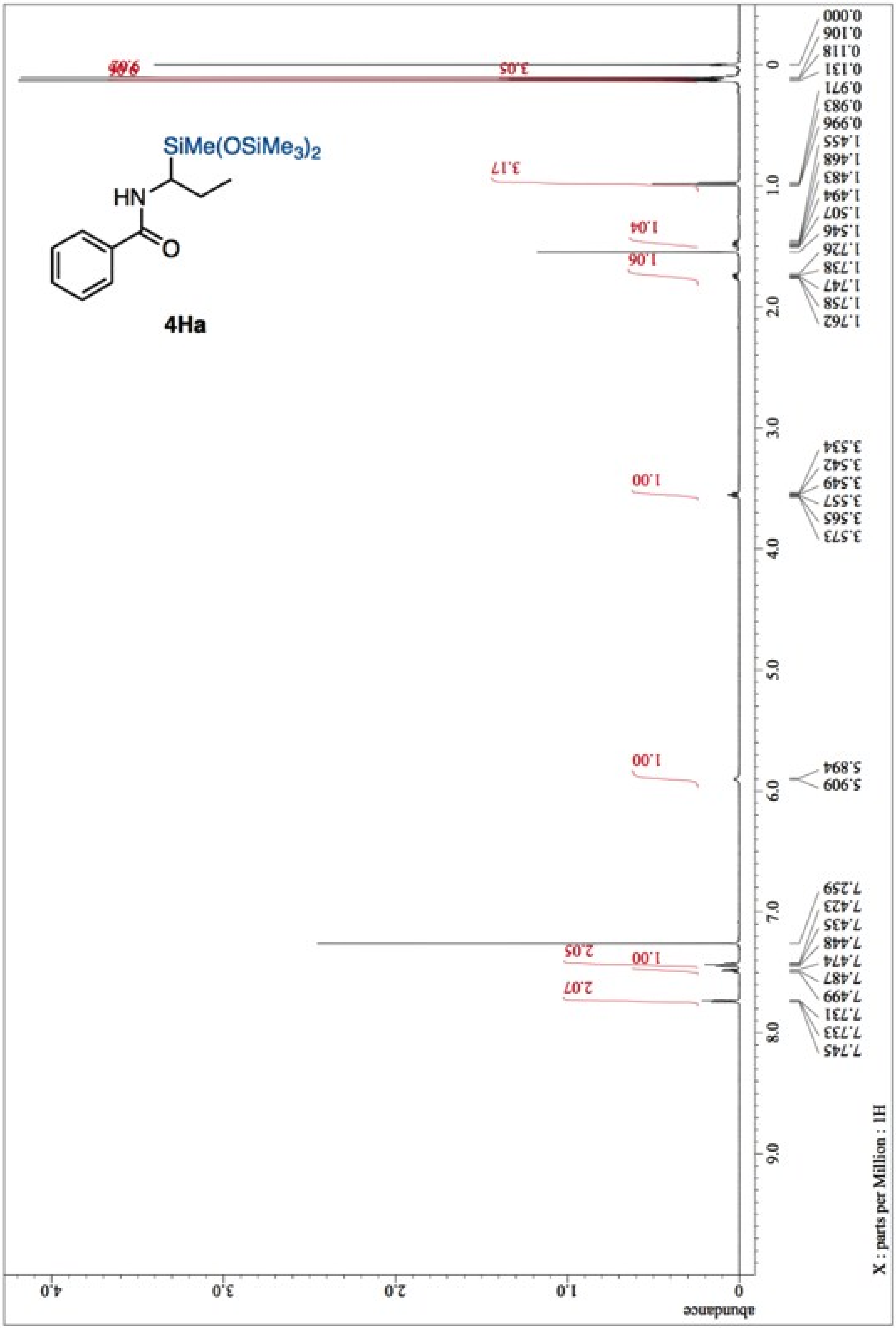

^{13}C NMR (151MHz, $CDCl_3$) of 4Ha

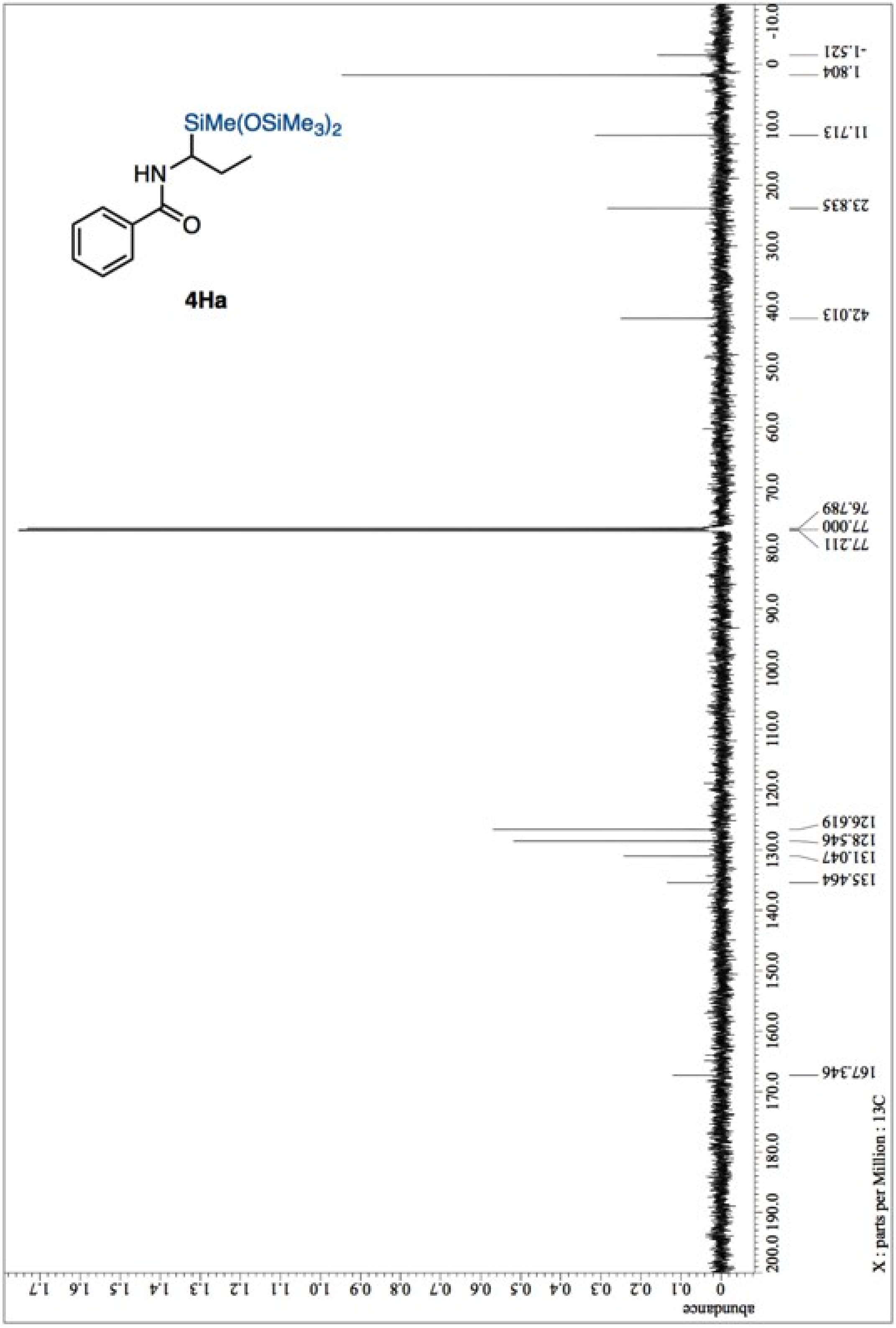

^{1}H NMR (600MHz, $CDCl_3$) of 4Ia

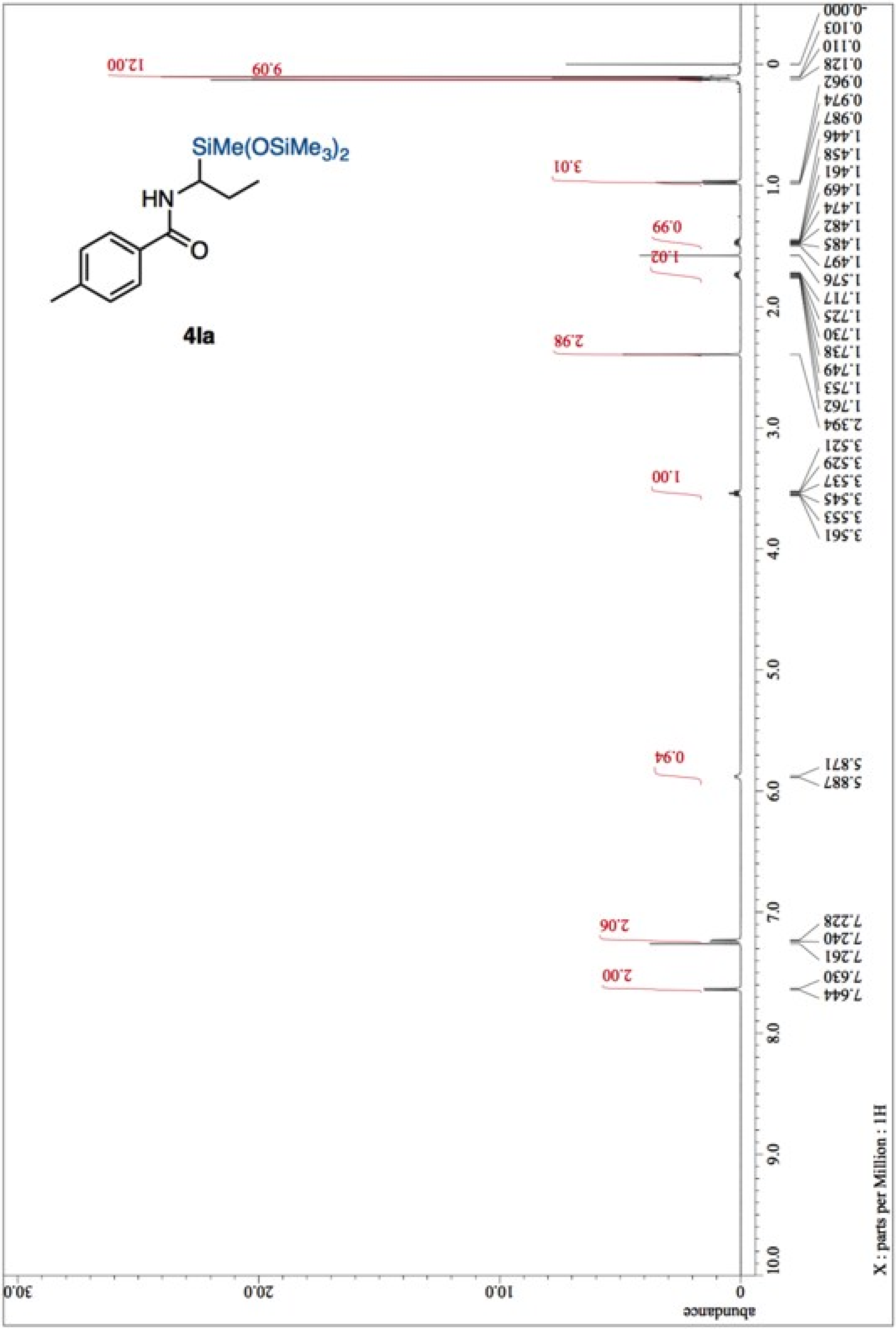

^{13}C NMR (151MHz, $CDCl_3$) of 4Ia

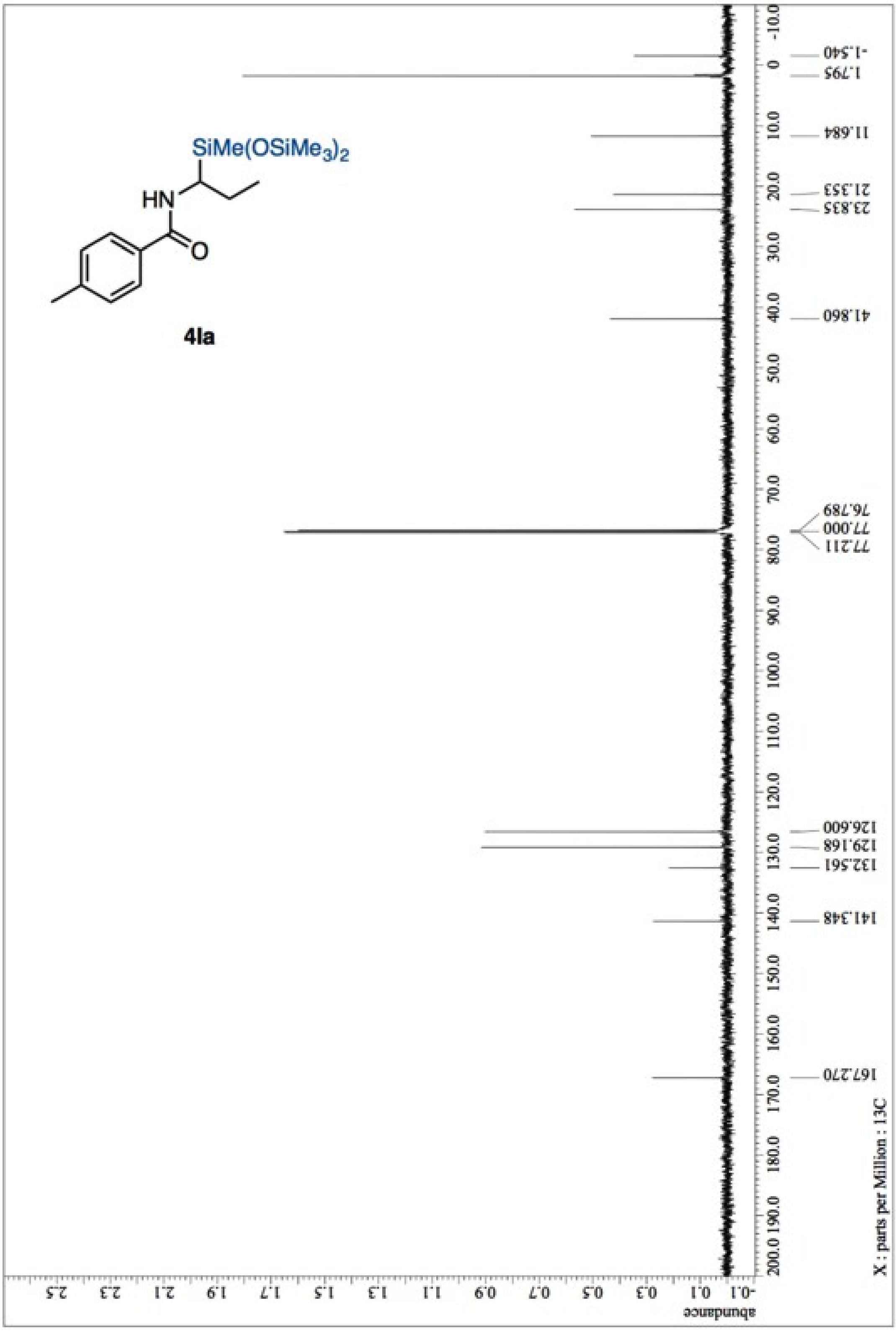

^{1}H NMR (600MHz, $CDCl_3$) of 4Ja

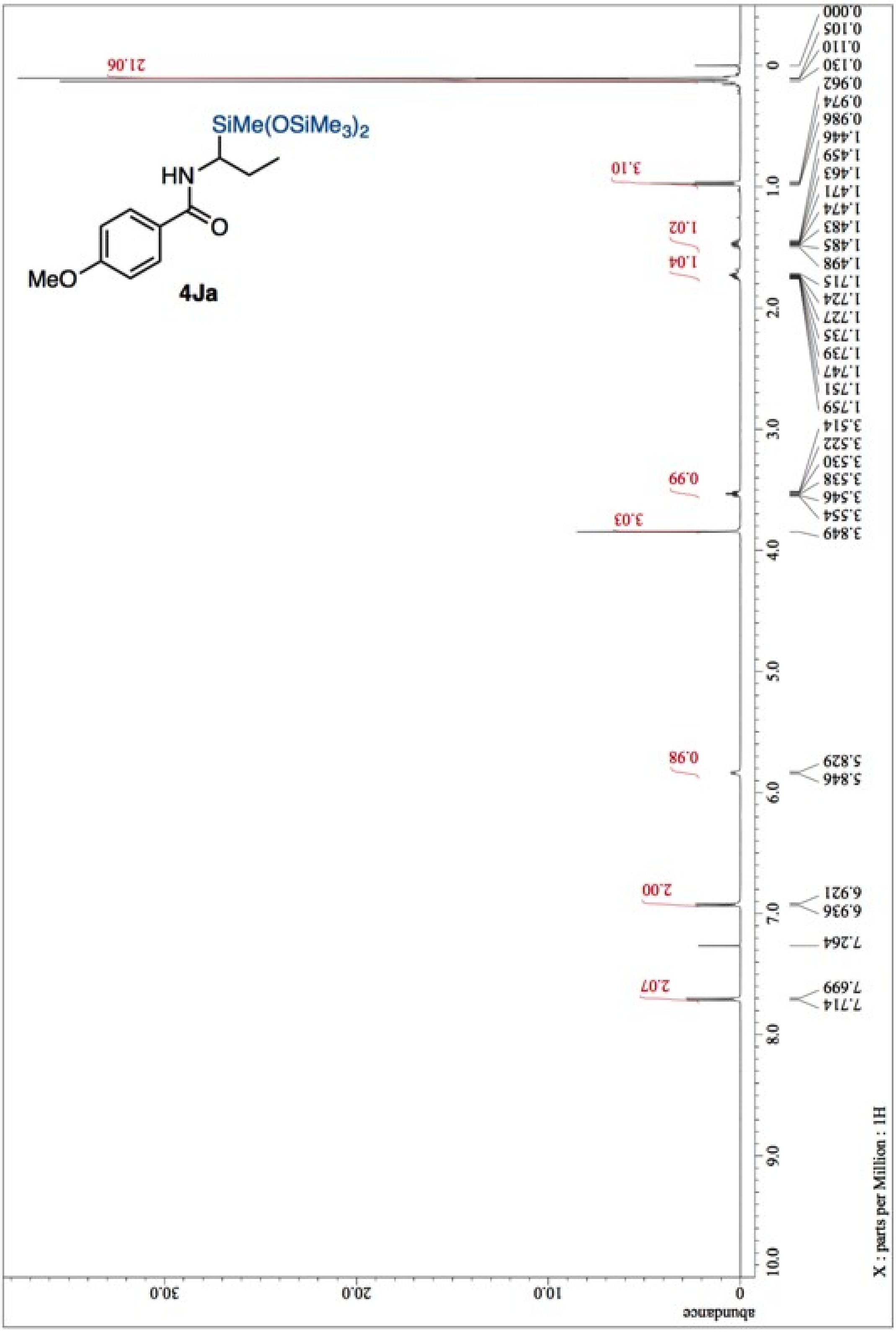

^{13}C NMR (151MHz, $CDCl_3$) of 4Ja

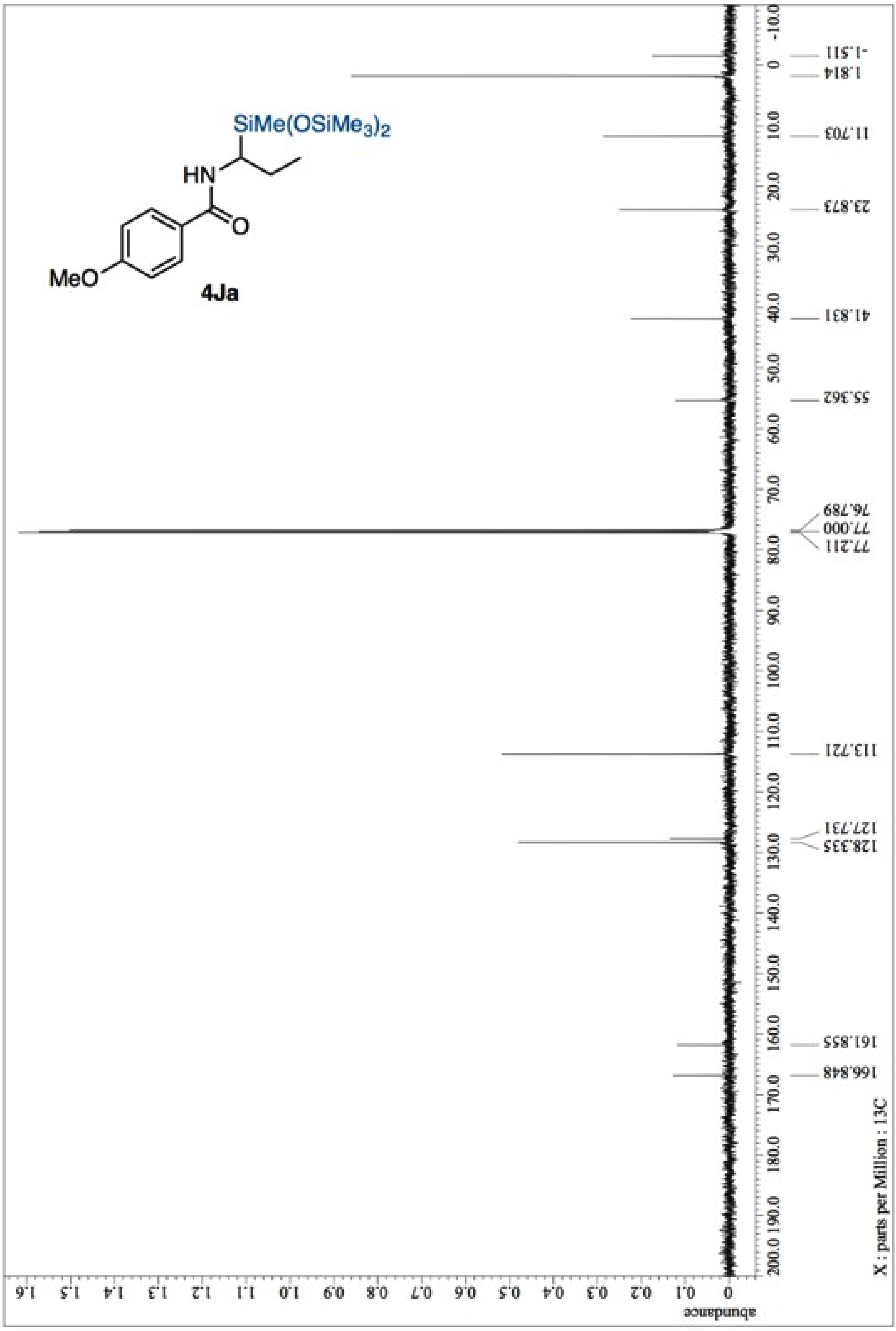

^{1}H NMR (600MHz, $CDCl_3$) of 4Ka

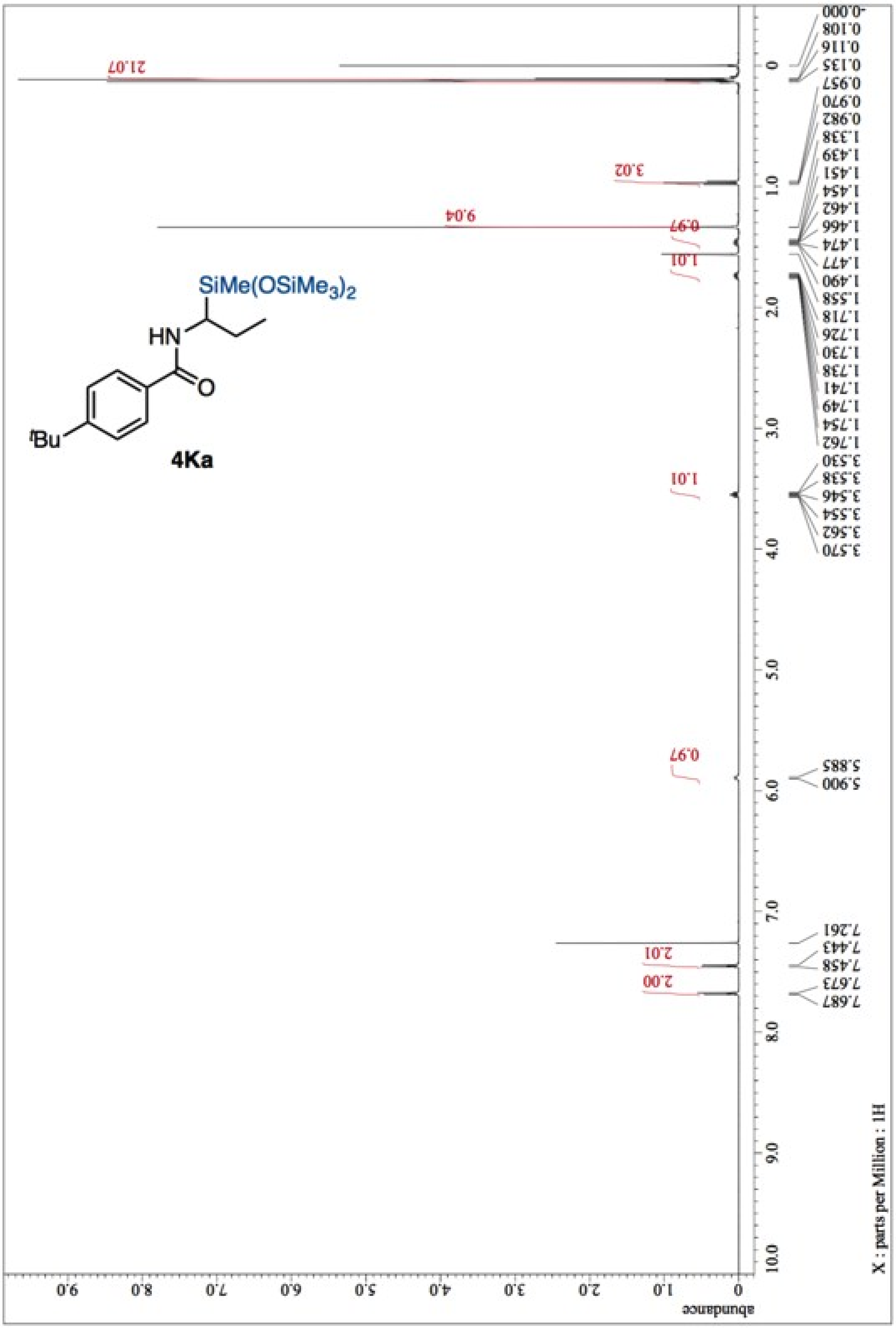

^{13}C NMR (151MHz, $CDCl_3$) of 4Ka

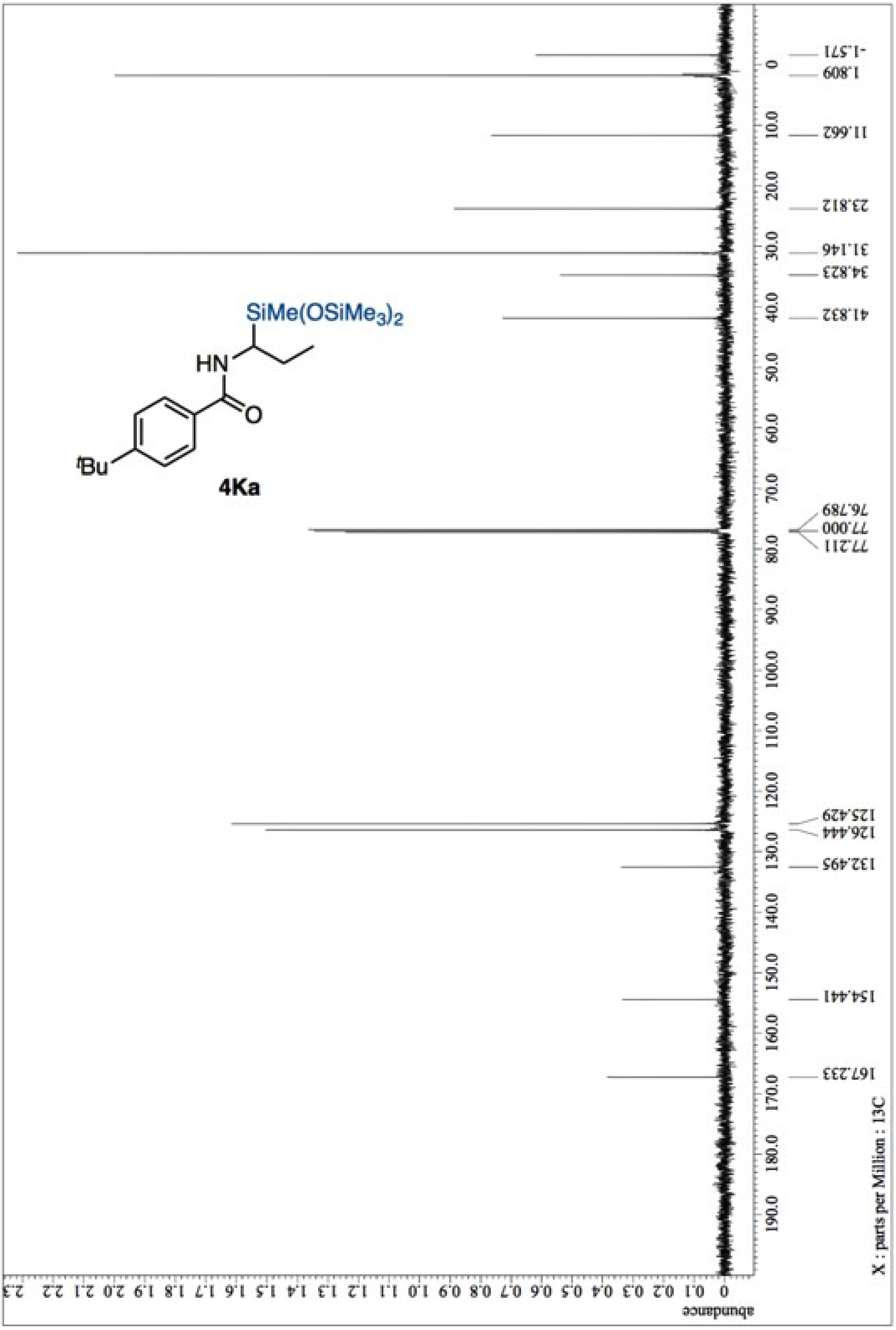

^{1}H NMR (600MHz, $CDCl_3$) of 4La

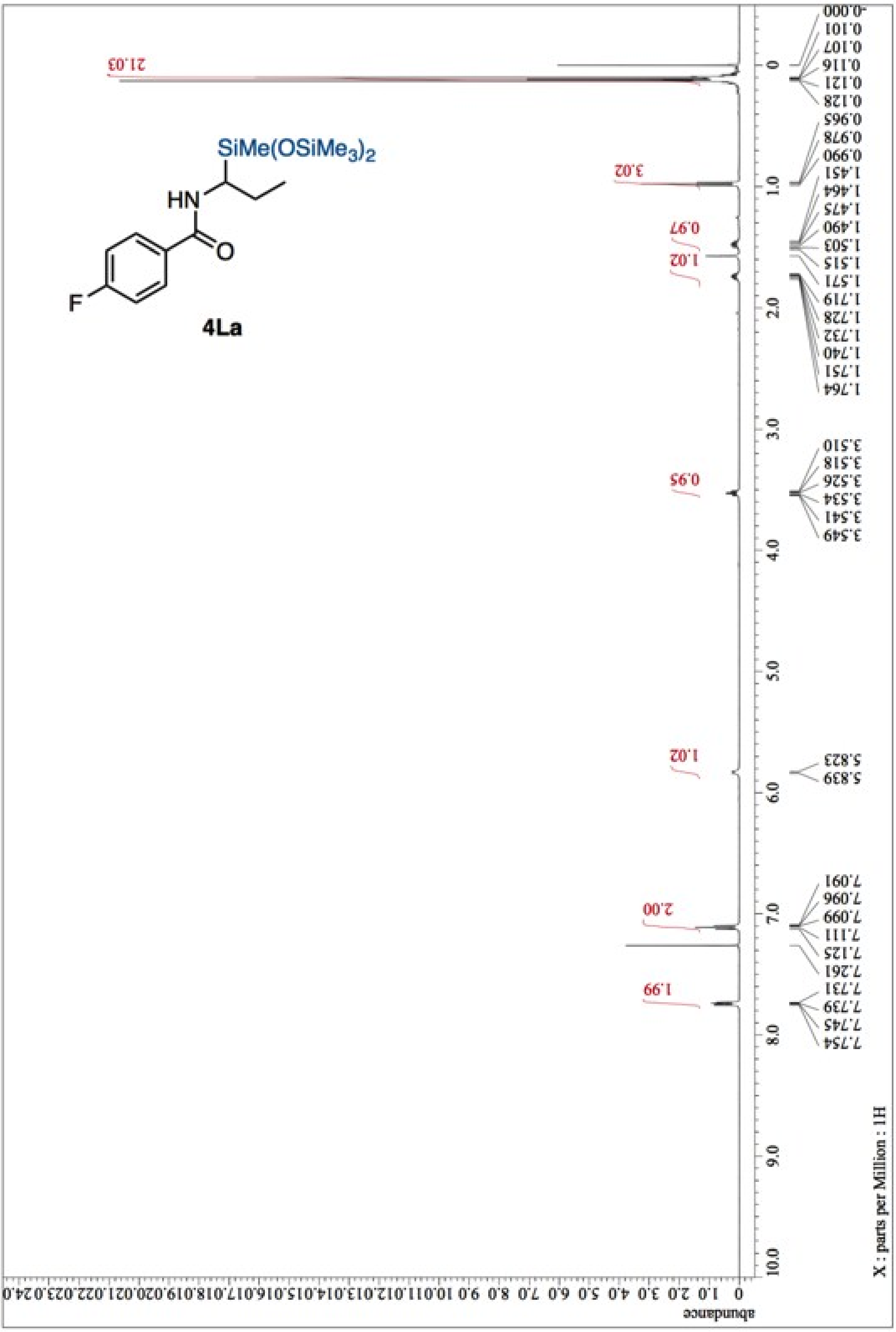

^{13}C NMR (151MHz, $CDCl_3$) of 4La

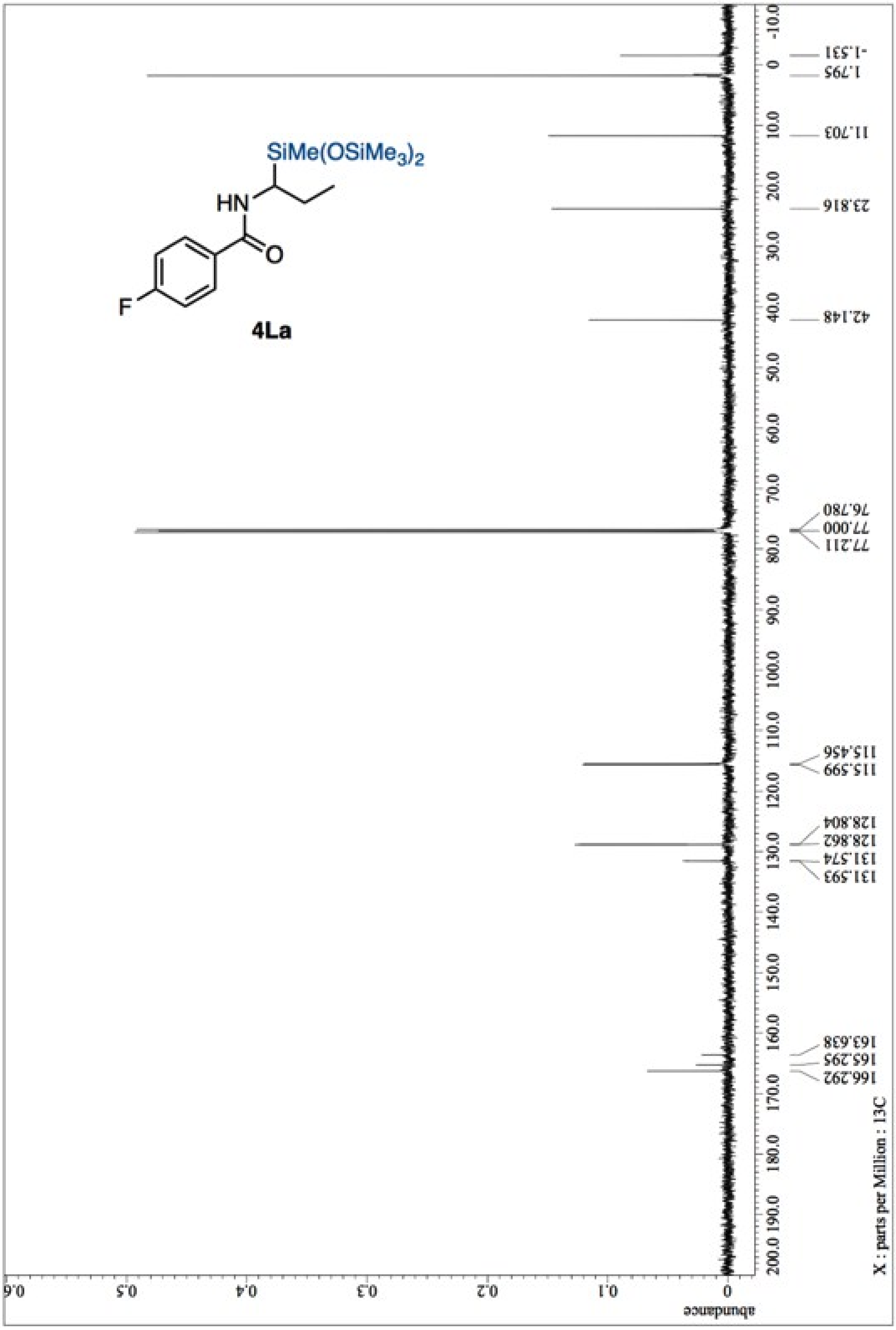

^{1}H NMR (600MHz, $CDCl_3$) of 4Ma

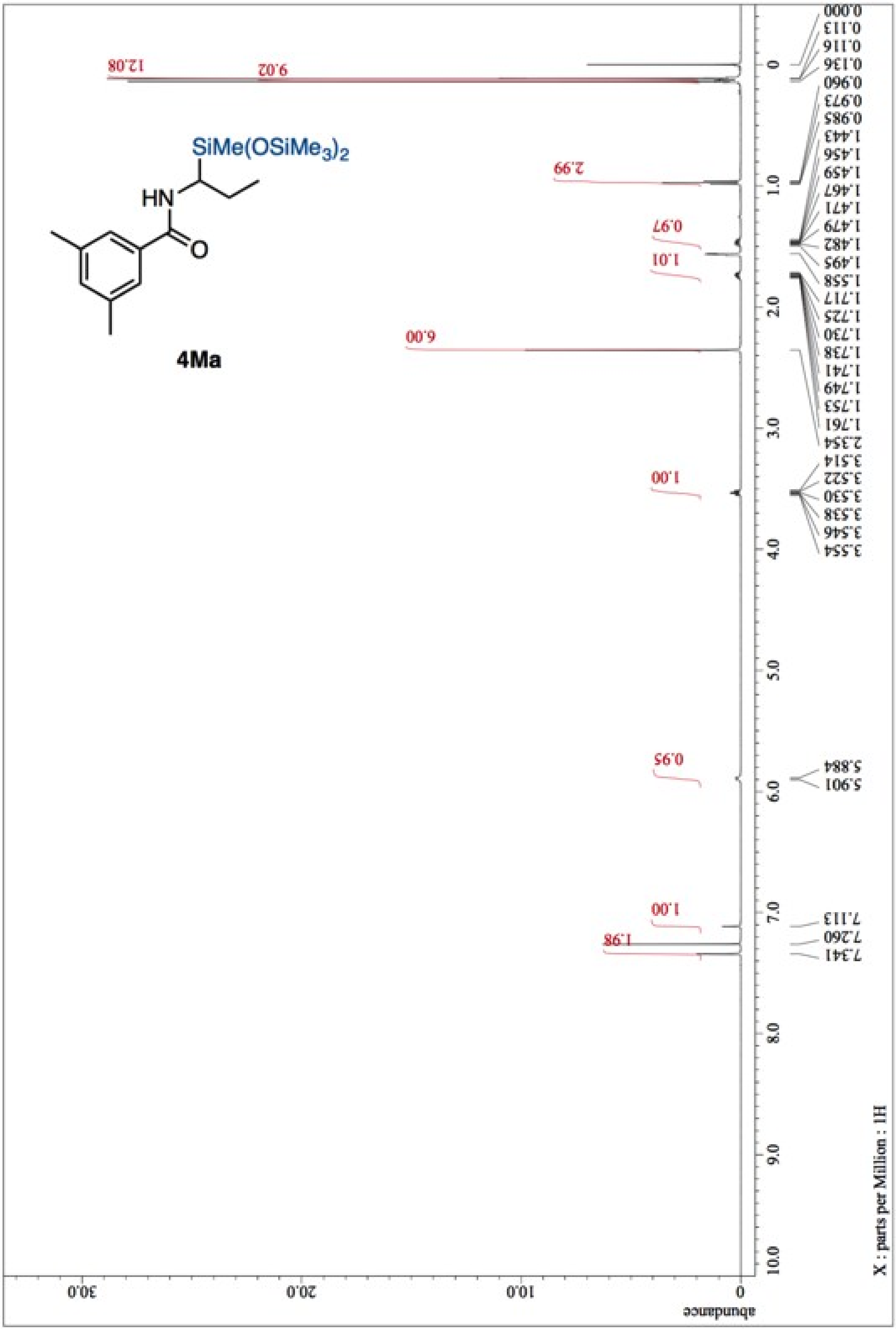

^{13}C NMR (151MHz, CDCl$_3$) of 4Ma

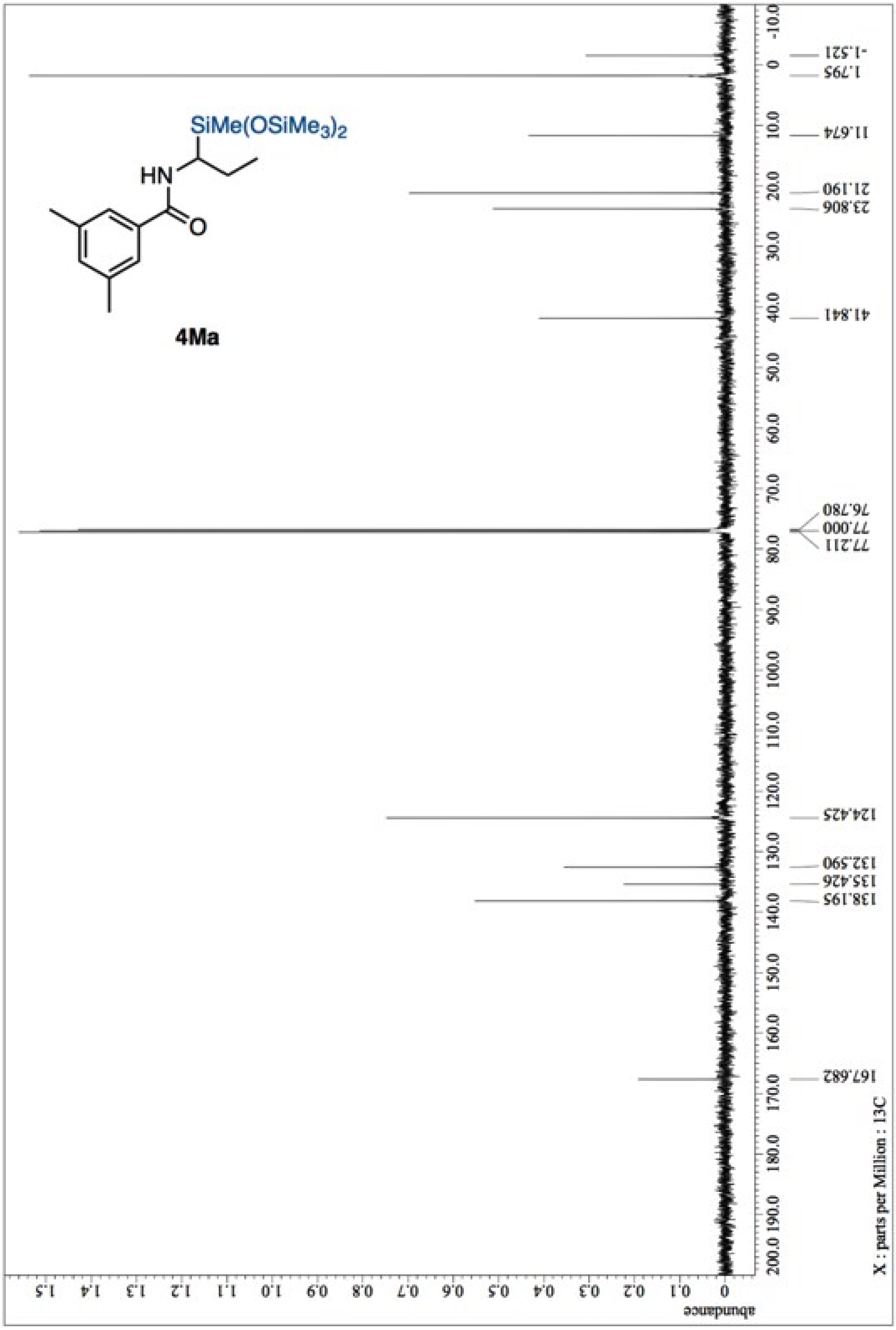

^{1}H NMR (600MHz, $CDCl_3$) of 4Na

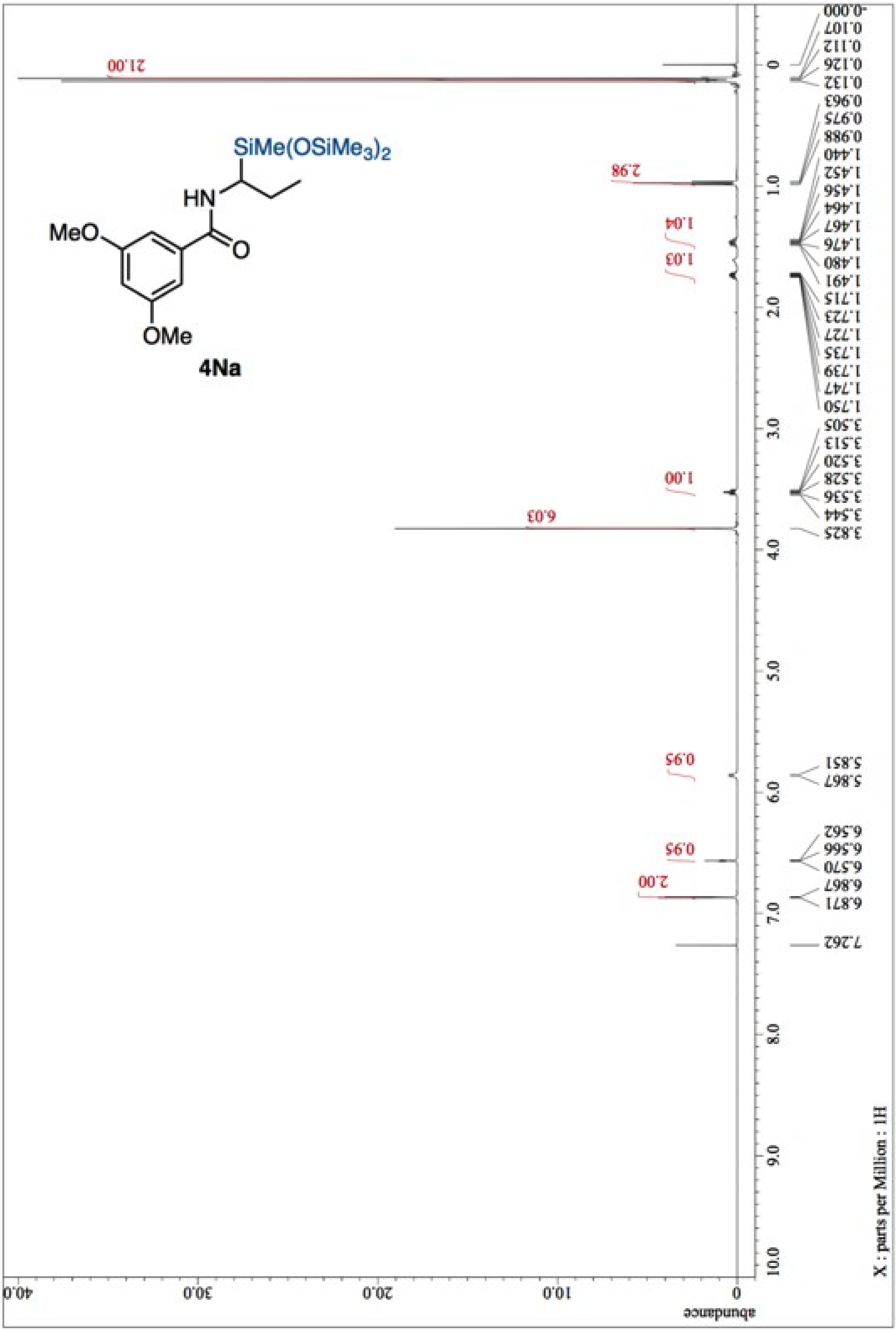

^{13}C NMR (151MHz, $CDCl_3$) of 4Na

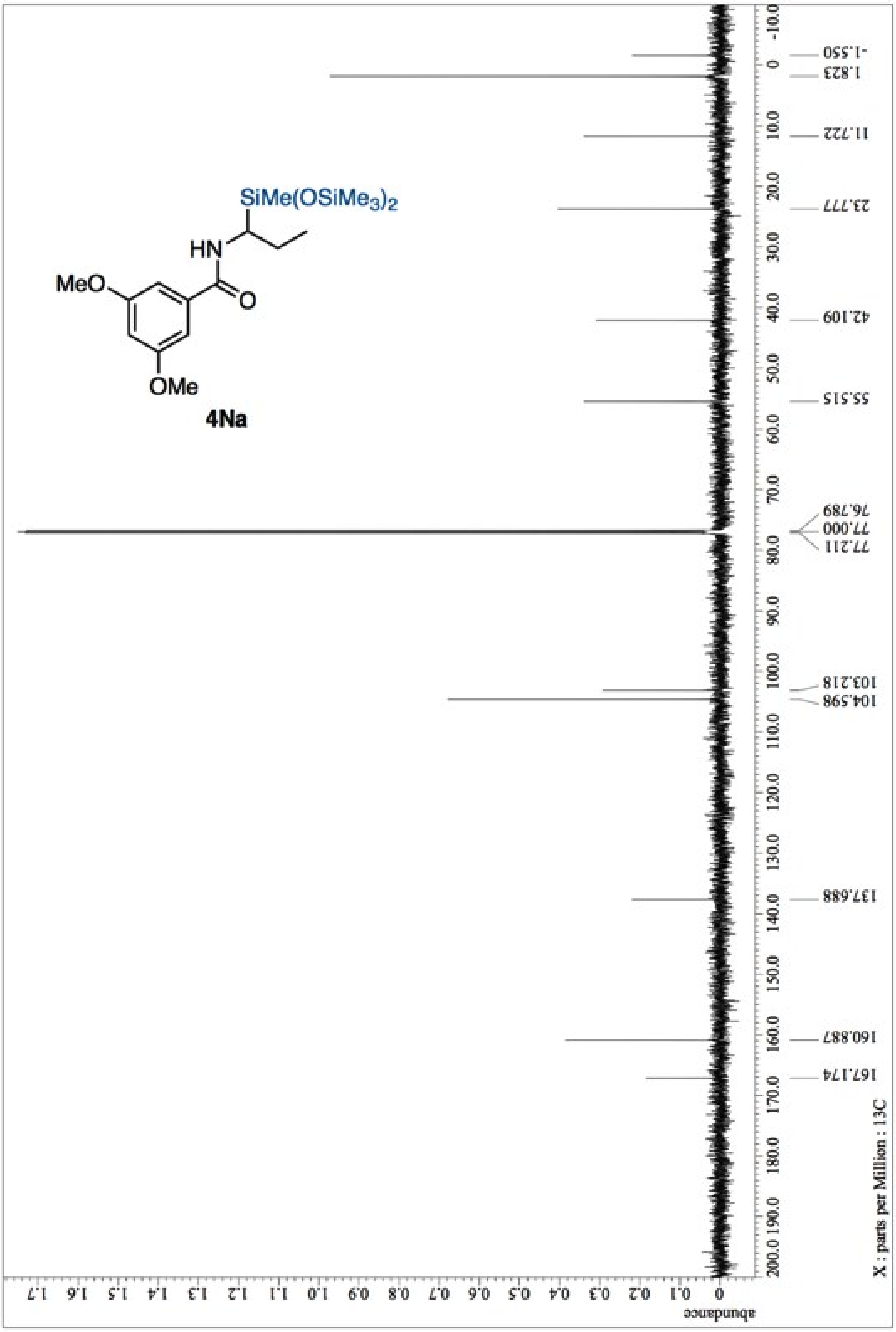

^{1}H NMR (600MHz, $CDCl_3$) of 4Oa

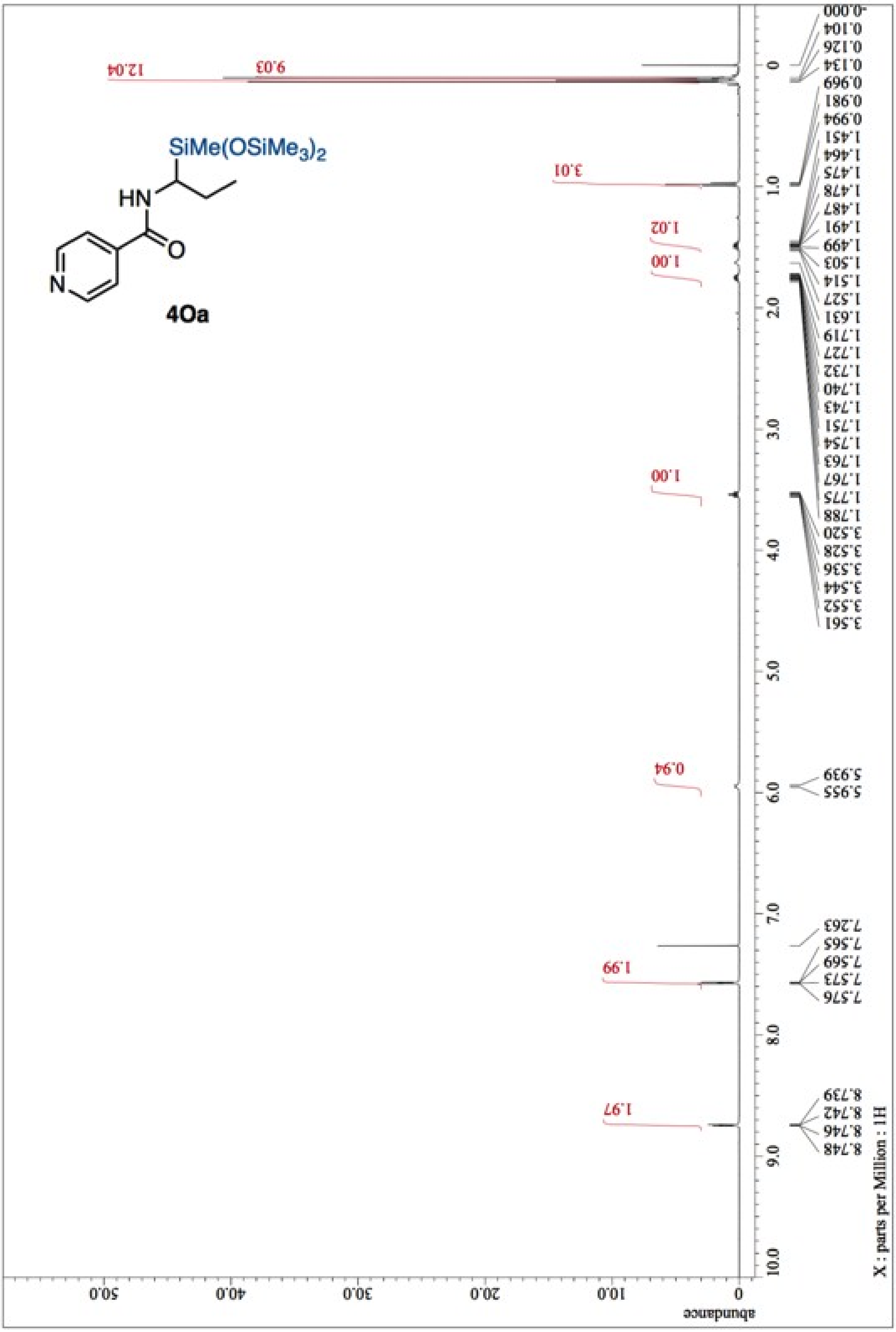

^{13}C NMR (151MHz, $CDCl_3$) of 4Oa

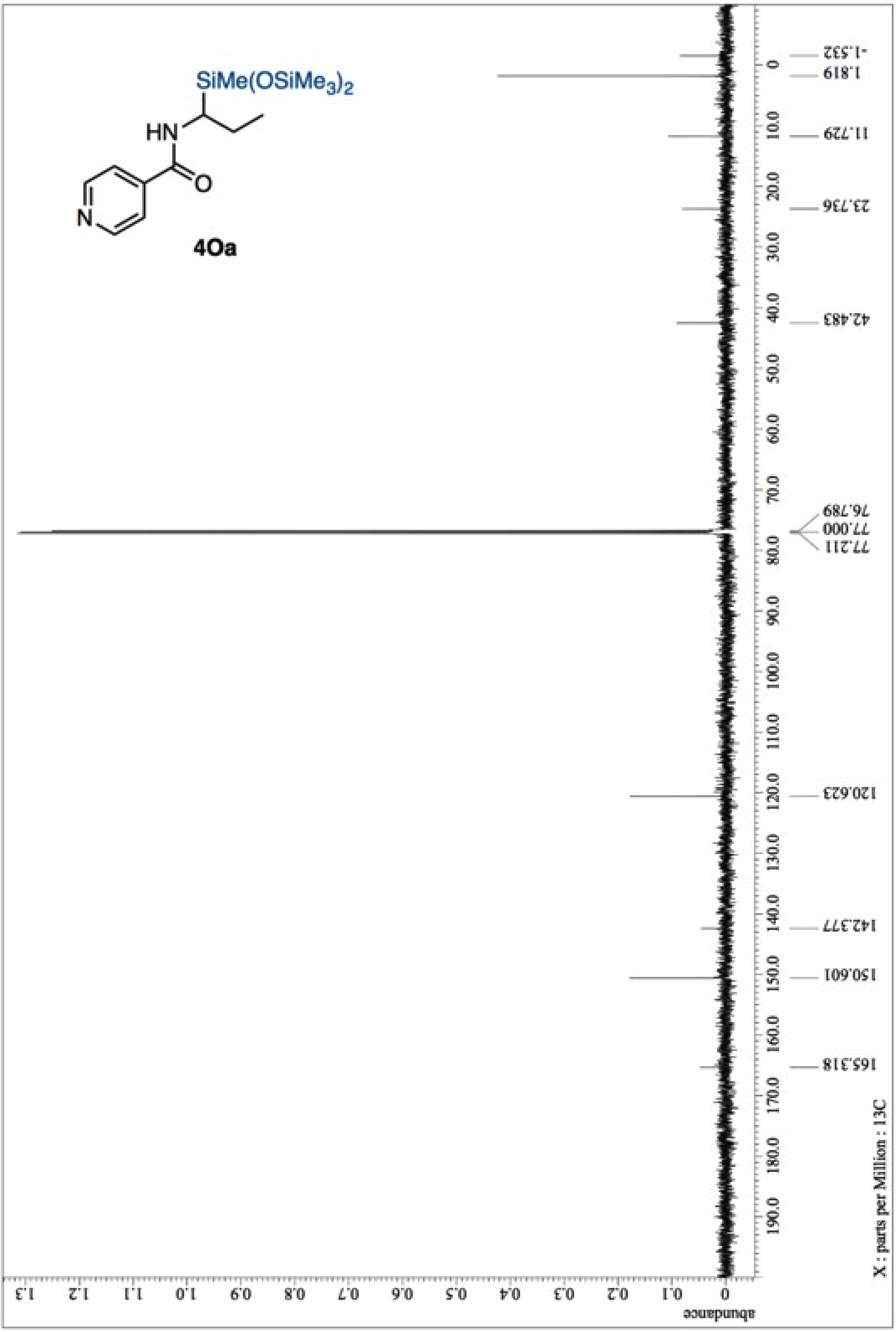

^{1}H NMR (600MHz, $CDCl_3$) of 4Pa

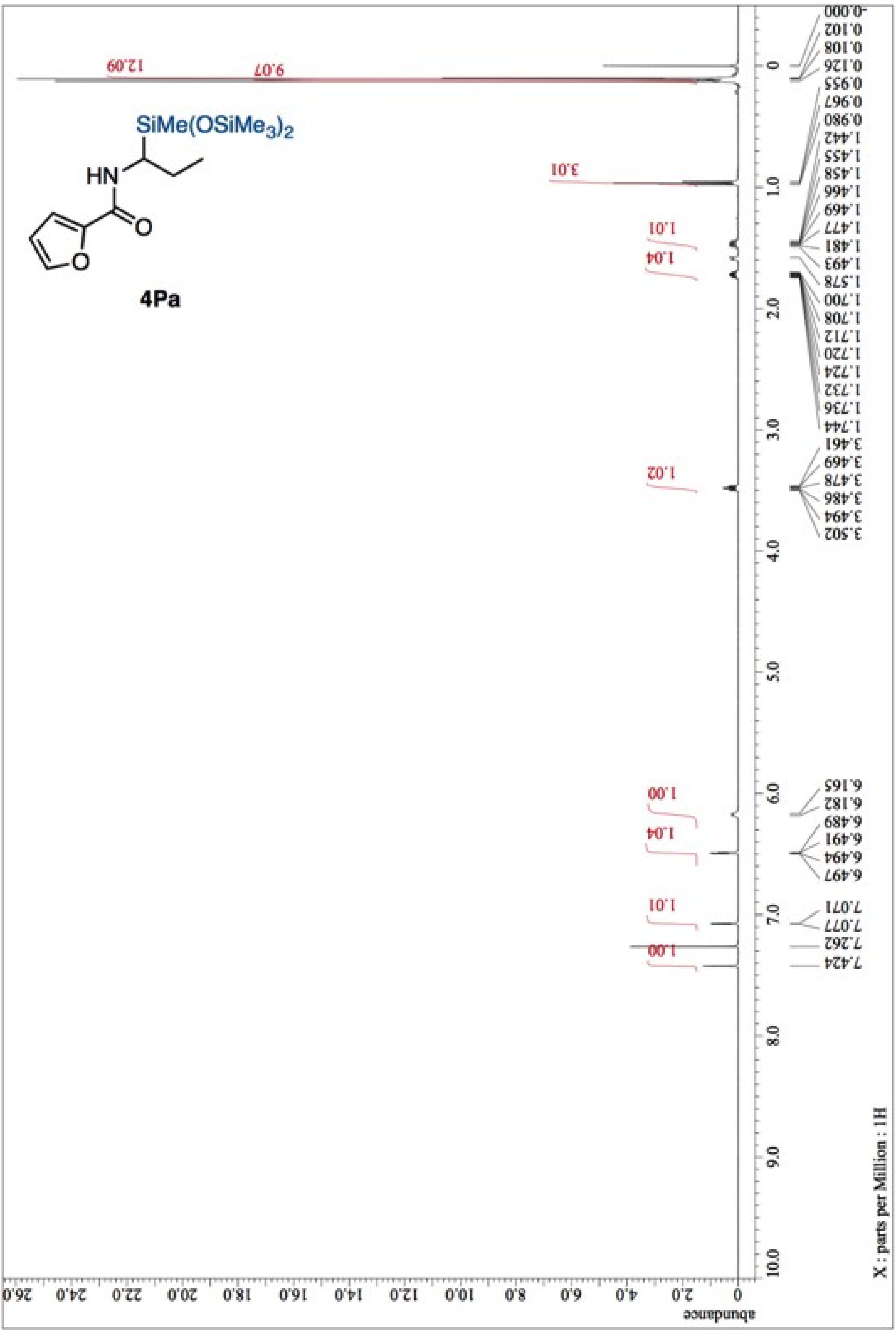

^{13}C NMR (151MHz, $CDCl_3$) of 4Pa

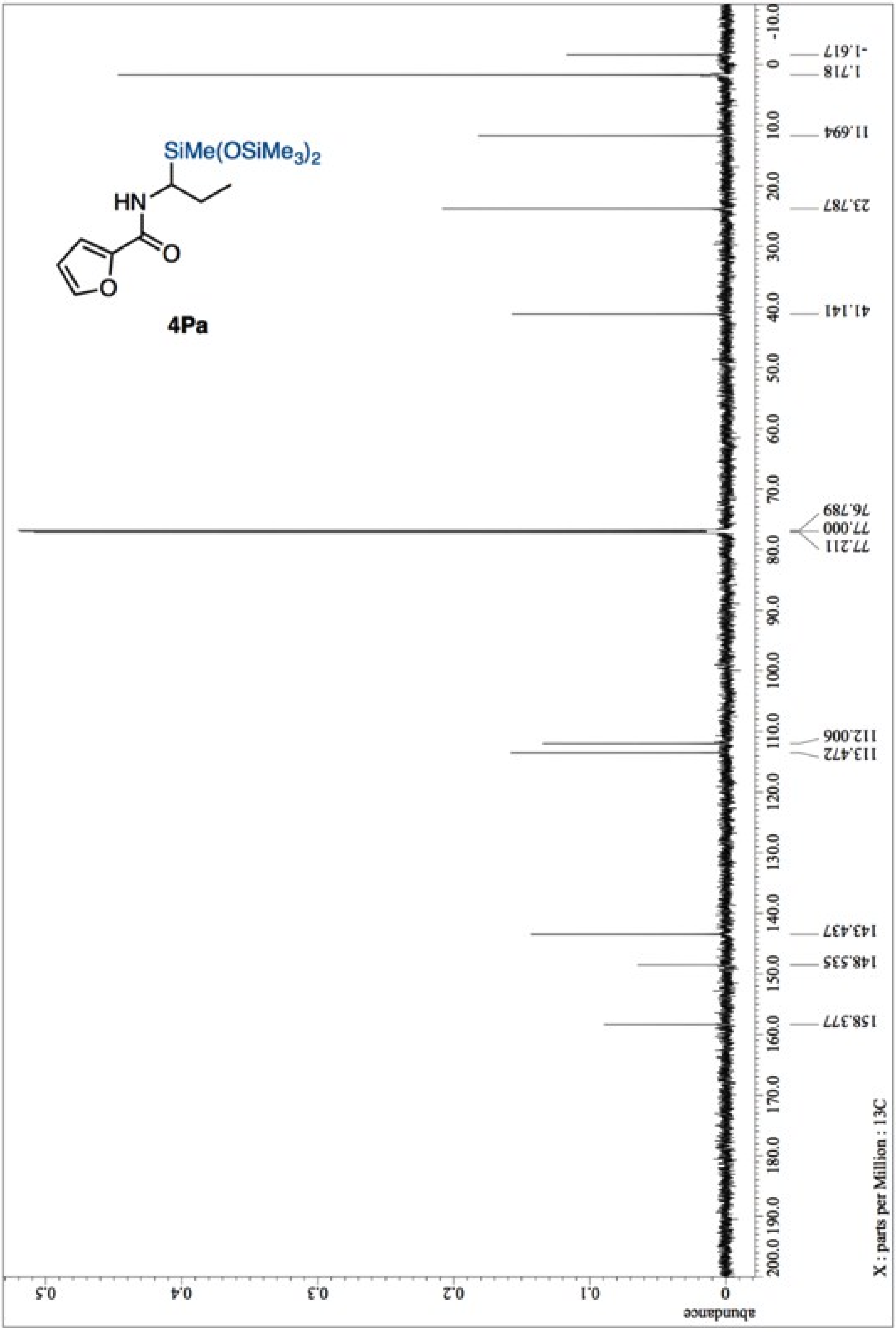

^{1}H NMR (500MHz, CDCl$_3$) of 4Qa

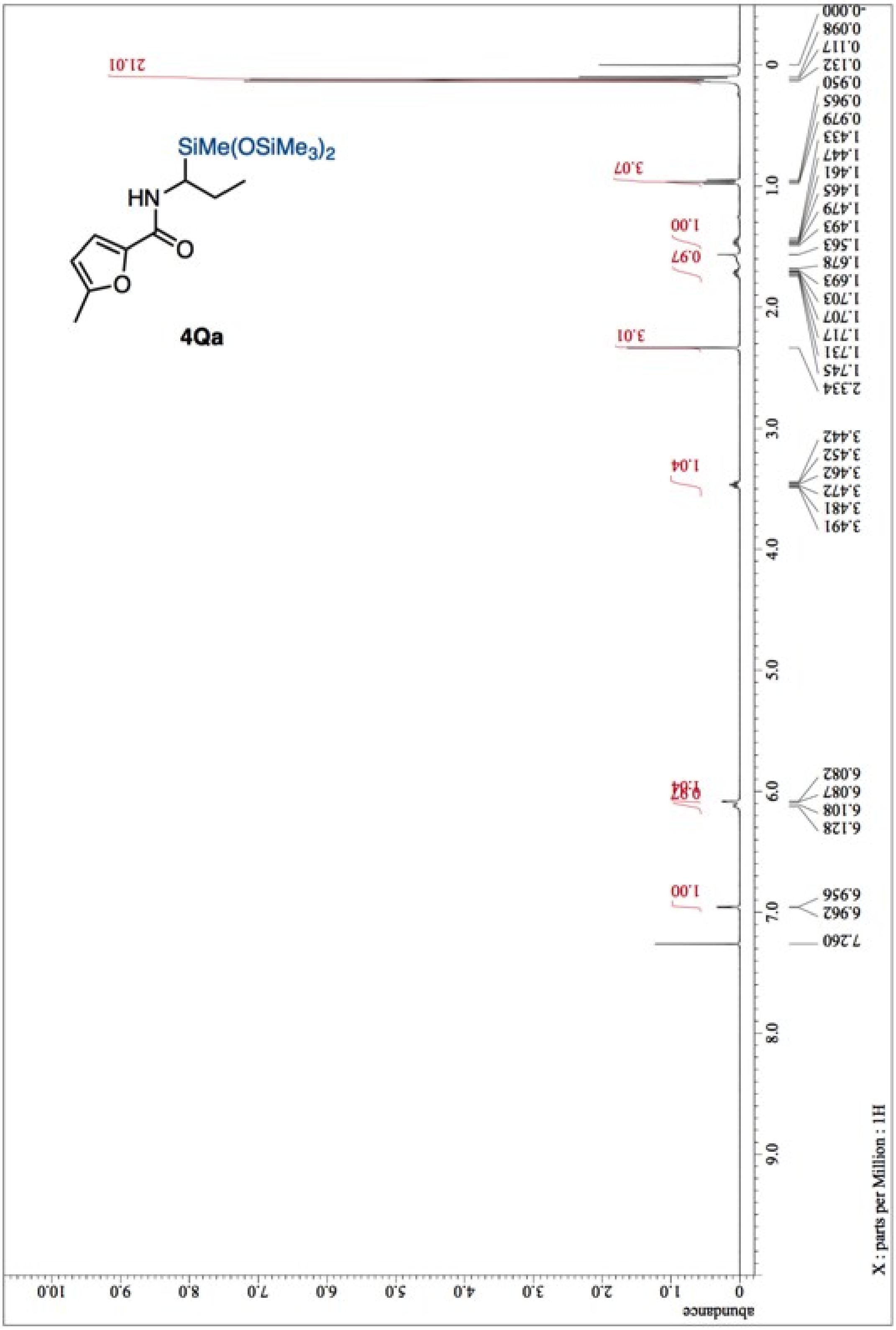

^{13}C NMR (151MHz, $CDCl_3$) of 4Qa

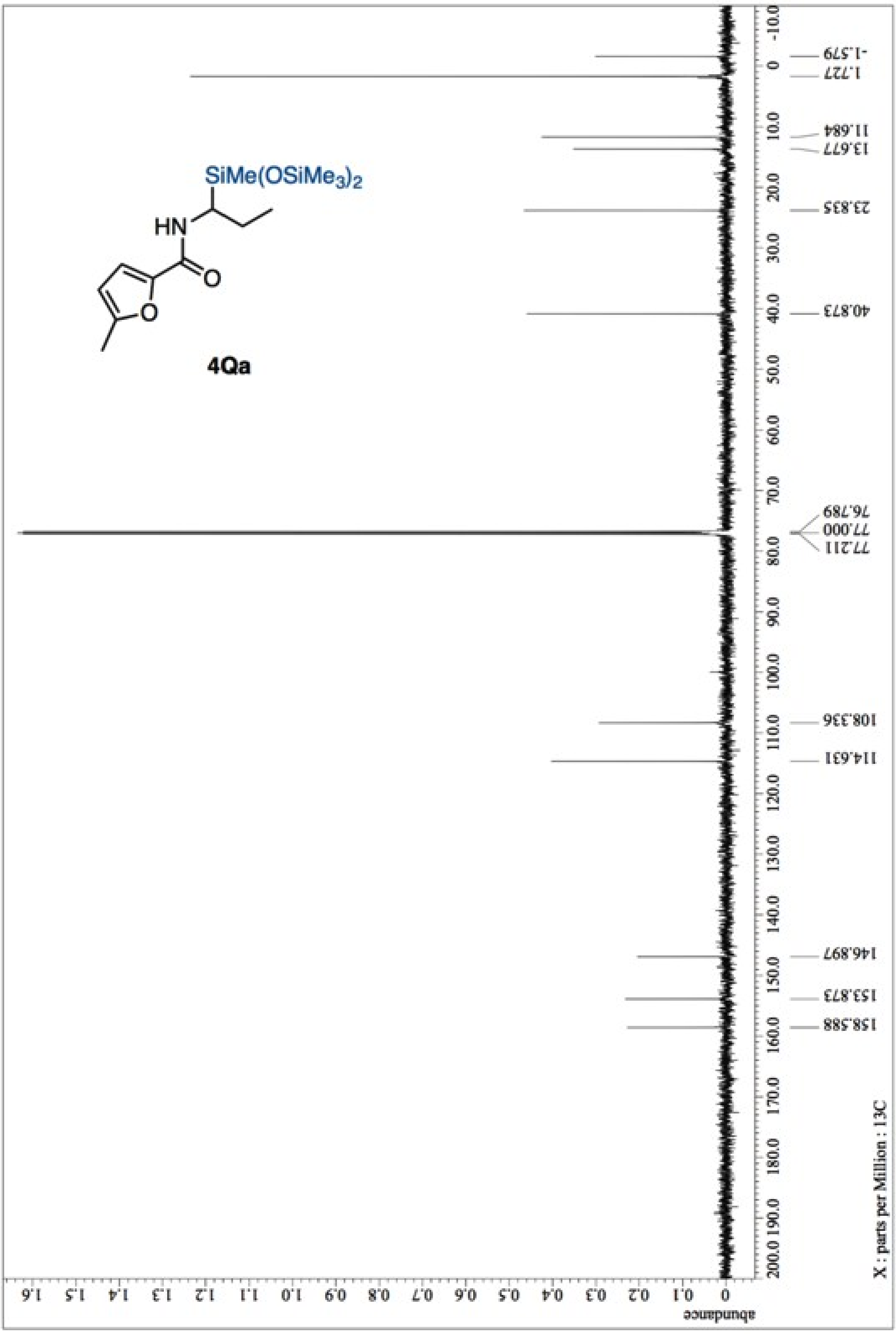

^{1}H NMR (500MHz, $CDCl_3$) of 4Ra

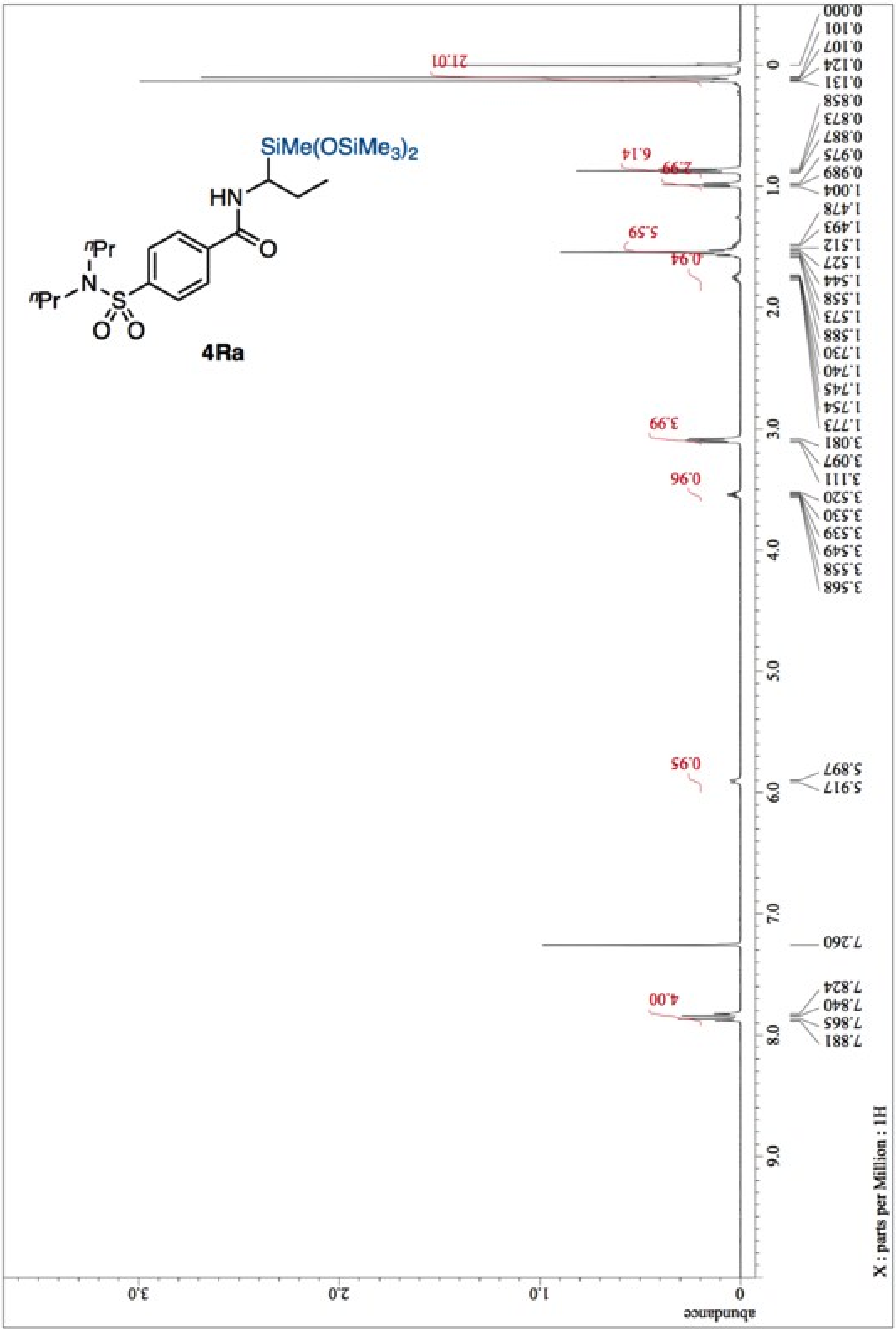

^{13}C NMR (151MHz, $CDCl_3$) of 4Ra

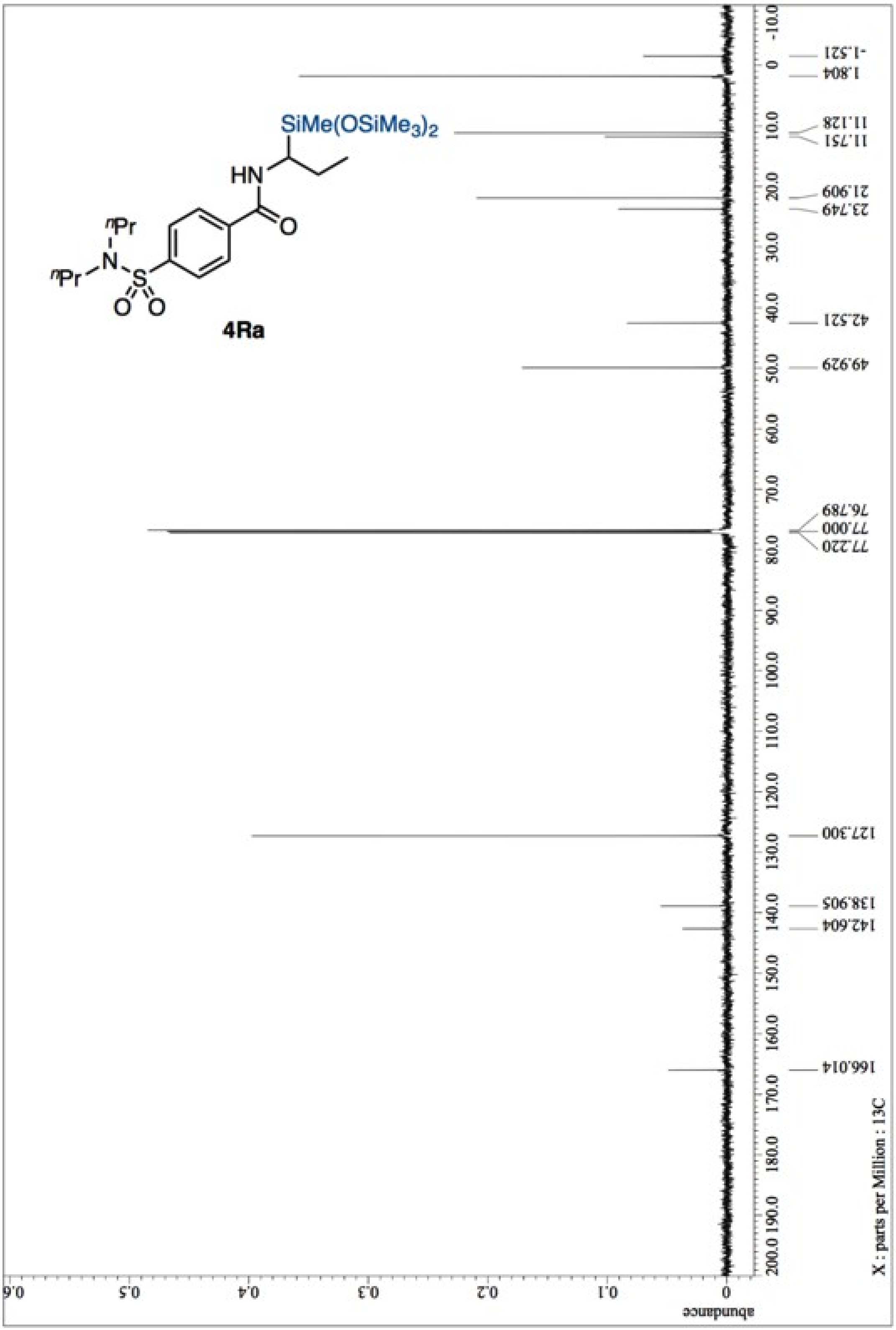

^{1}H NMR (600MHz, 60 ˚C, CDCl$_3$) of 4Sa

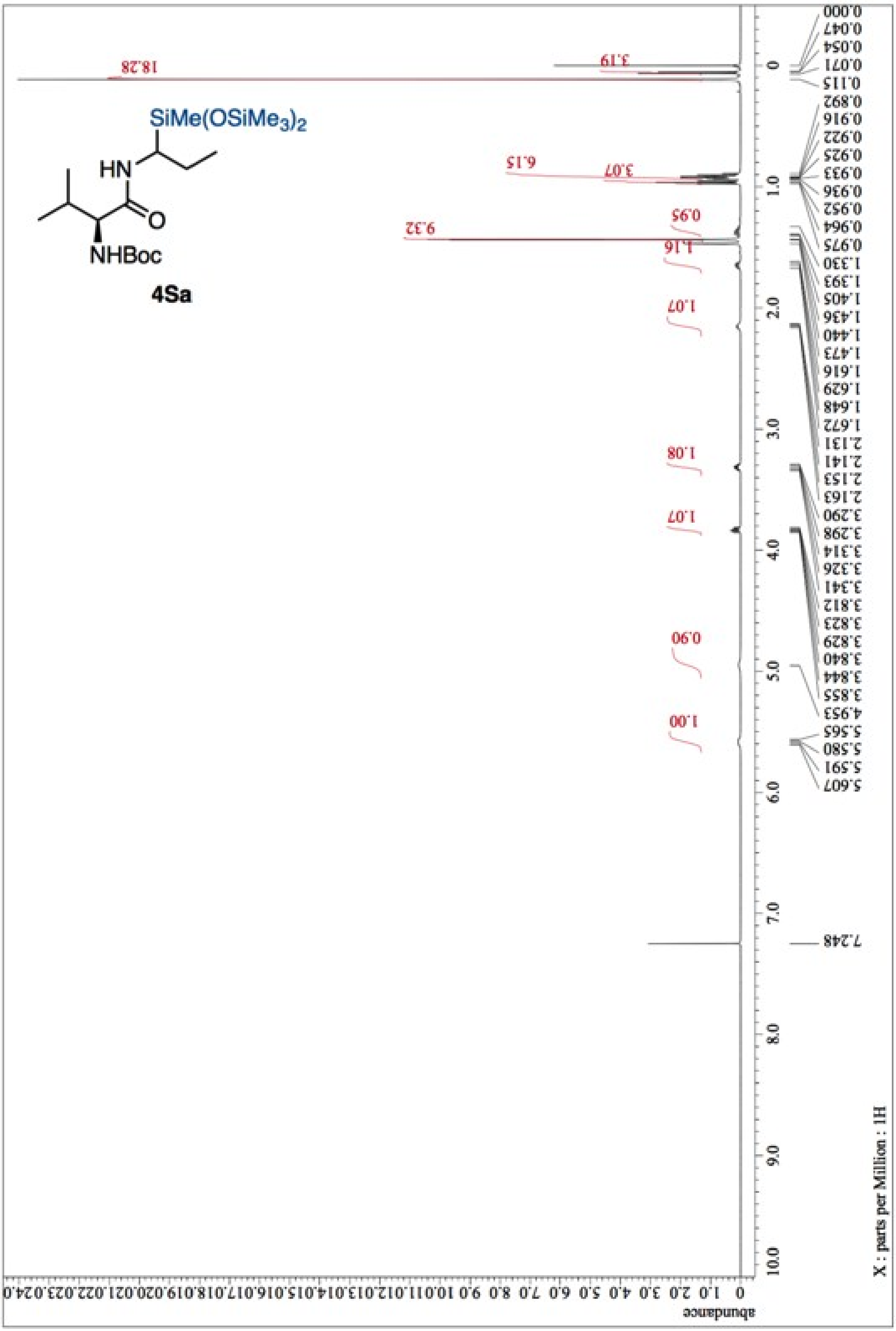

^{13}C NMR (101MHz, 60 ˚C, $CDCl_3$) of 4Sa

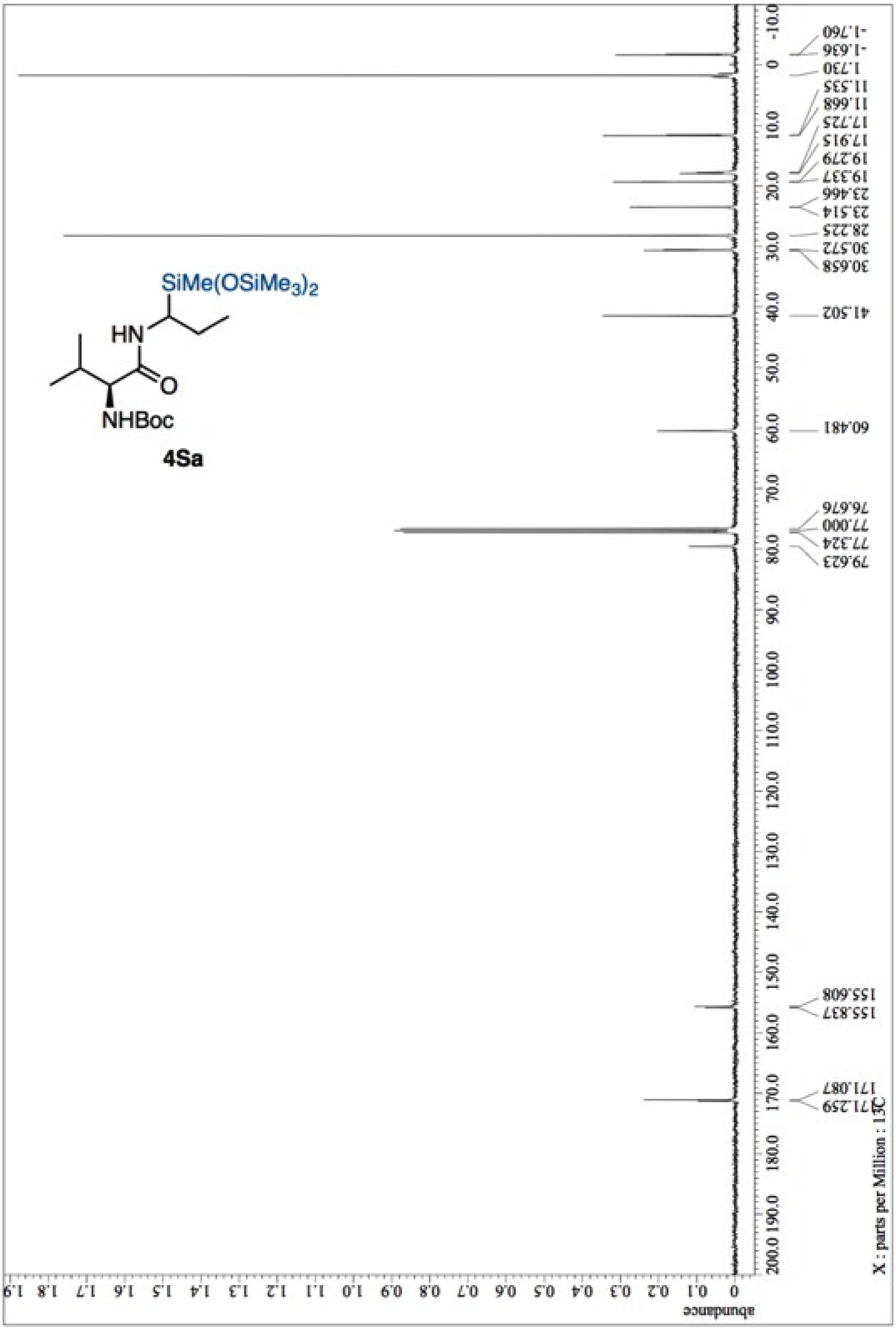

^{1}H NMR (600MHz, 120 ˚C, DMSO-d_6) of 4Ta

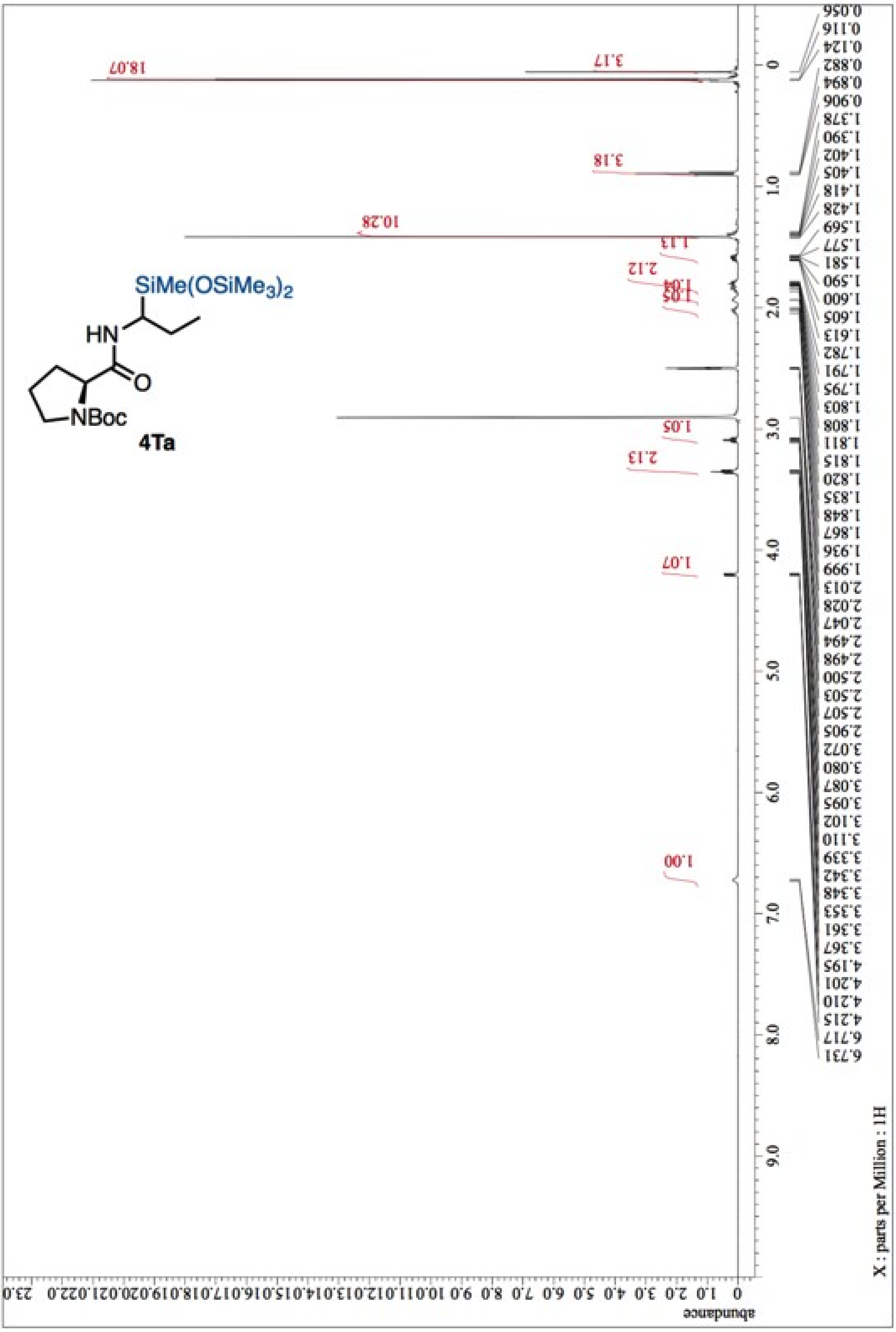

^{13}C NMR (151MHz, 120 ˚C, DMSO-d_6) of 4Ta

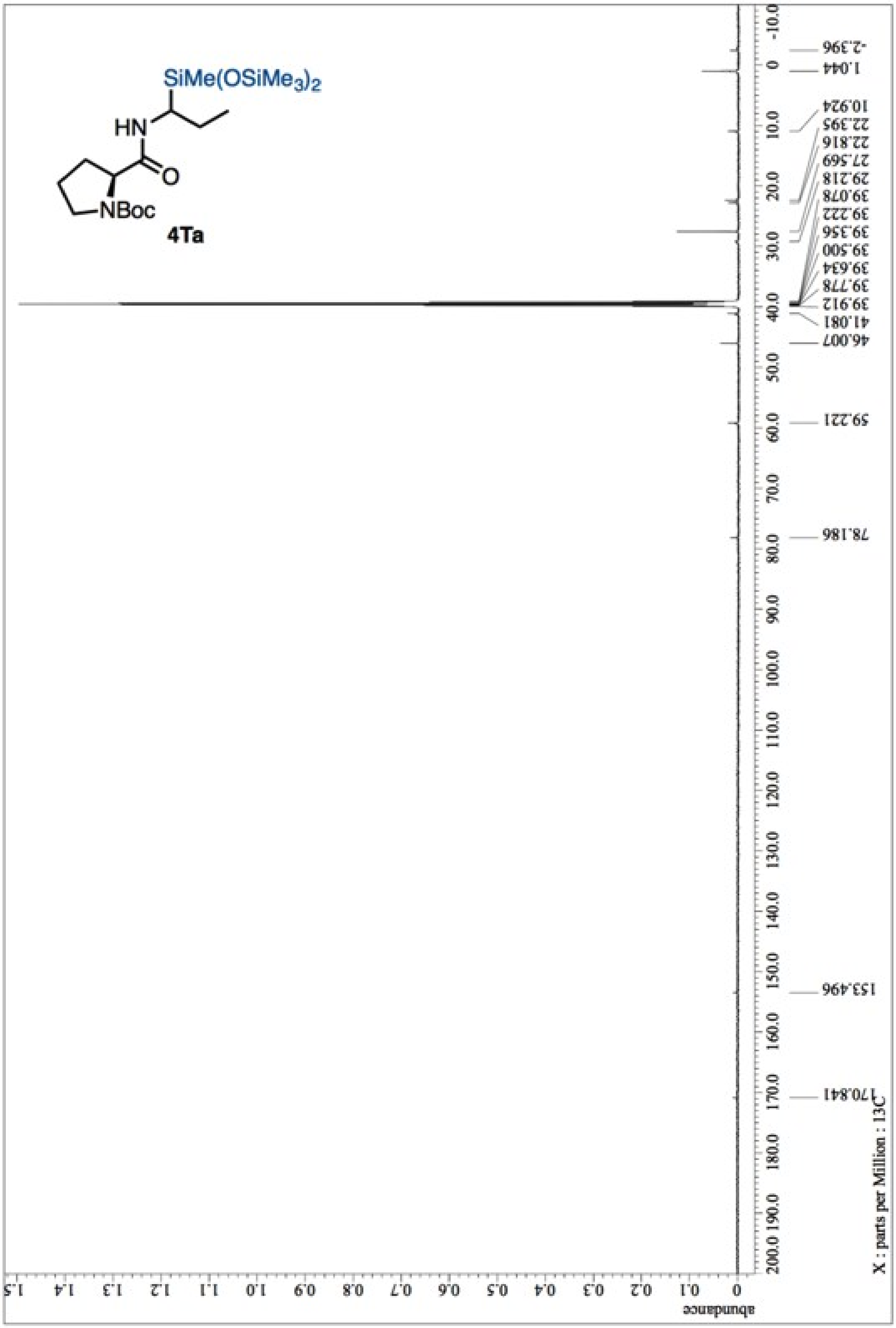

^{1}H NMR (600MHz, 120 ˚C, DMSO-d_6) of diastereomer-4Ta

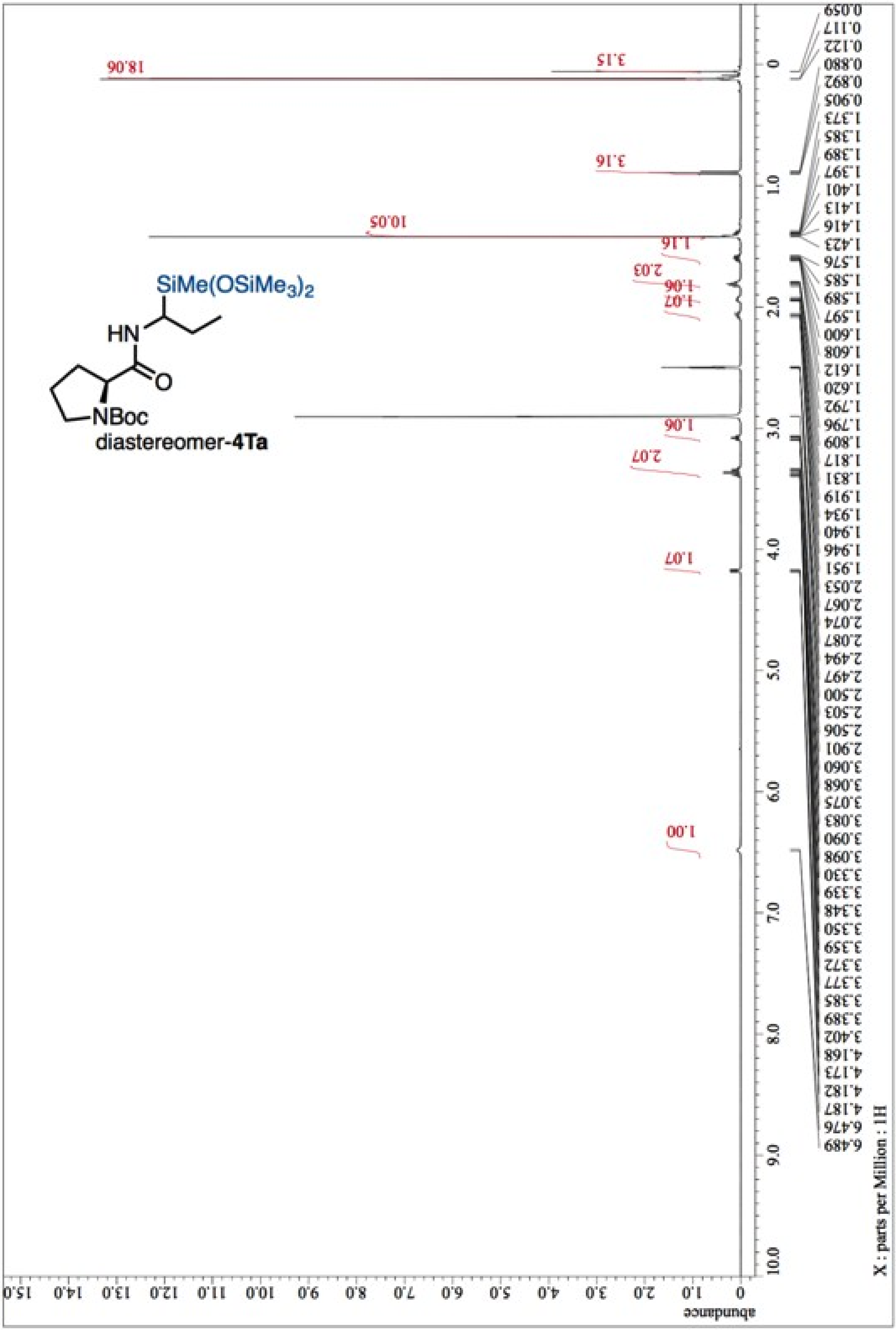

^{13}C NMR (151MHz, 120 ˚C, DMSO-d_6) of diastereomer-4Ta

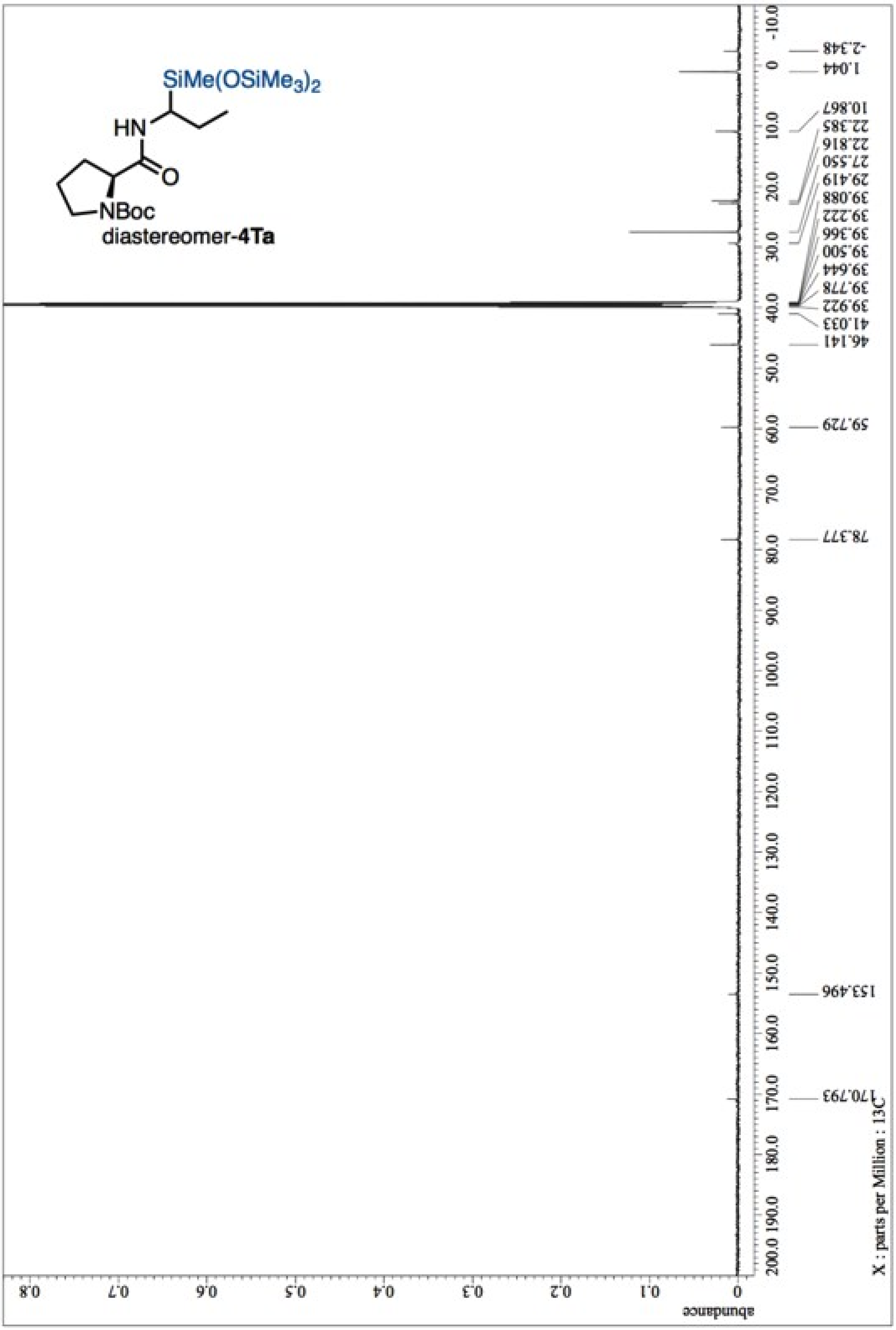

^{1}H NMR (600MHz, $CDCl_3$) of 4Ab

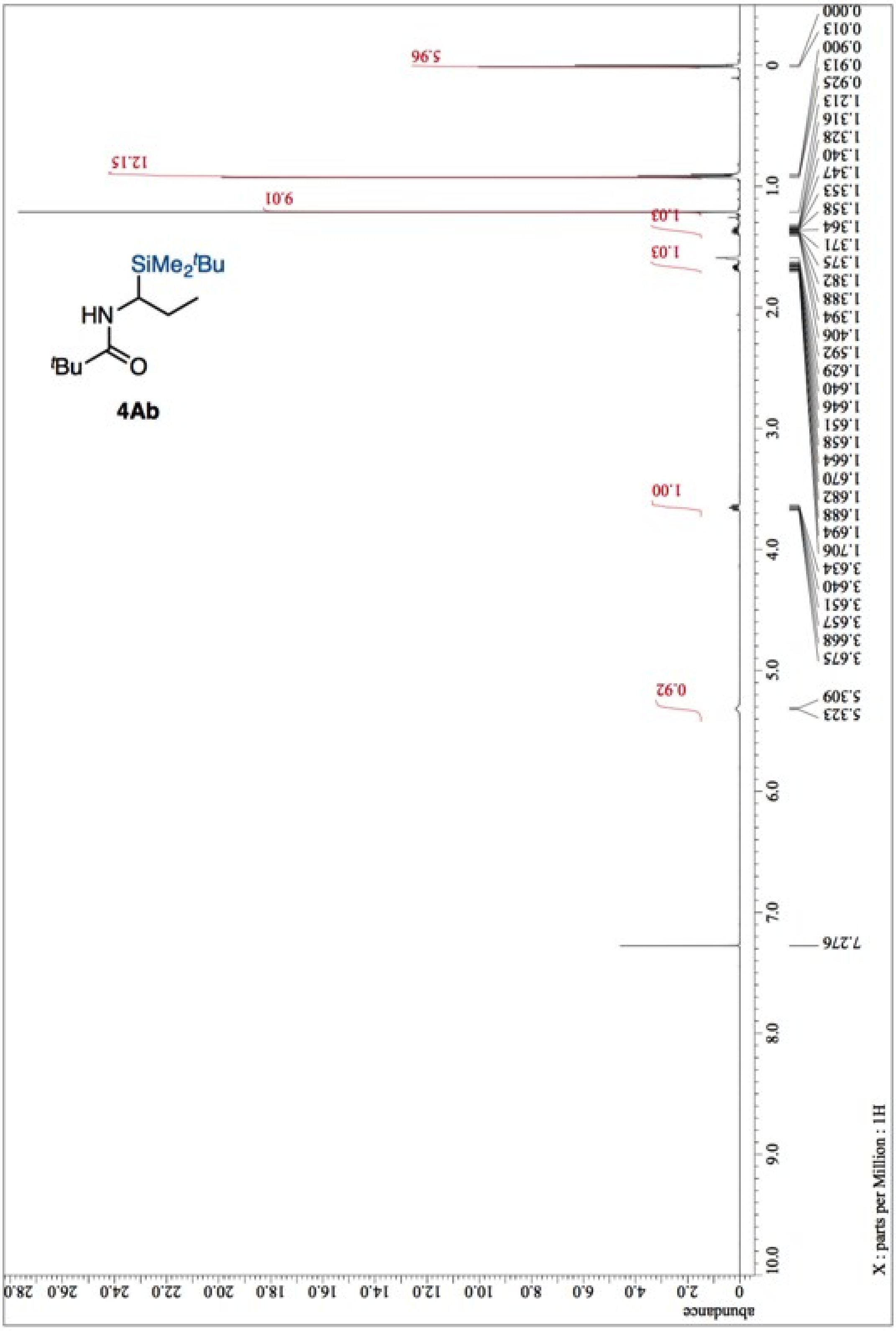

^{13}C NMR (151MHz, $CDCl_3$) of 4Ab

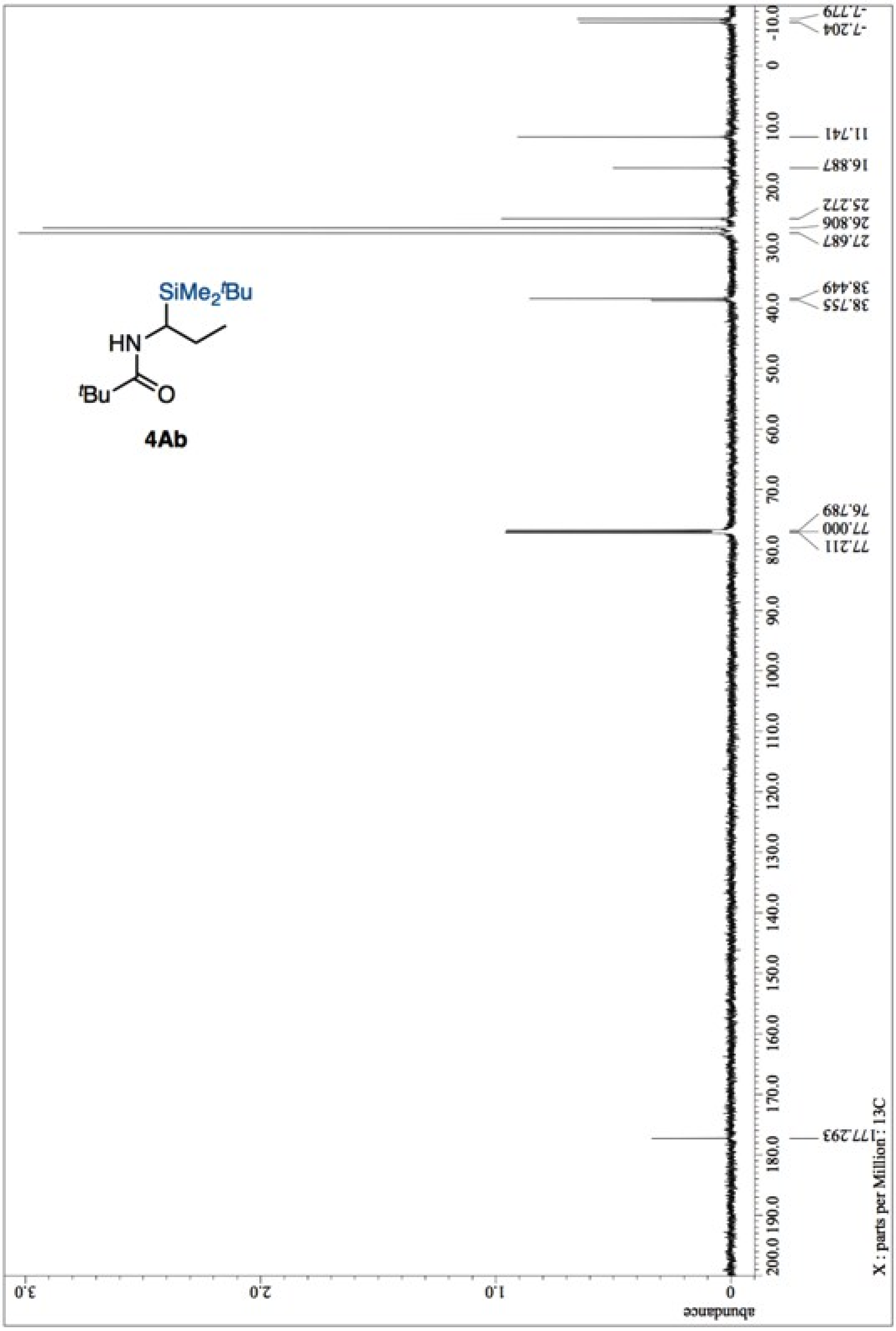

^{1}H NMR (600MHz, CDCl$_3$) of 4Ac

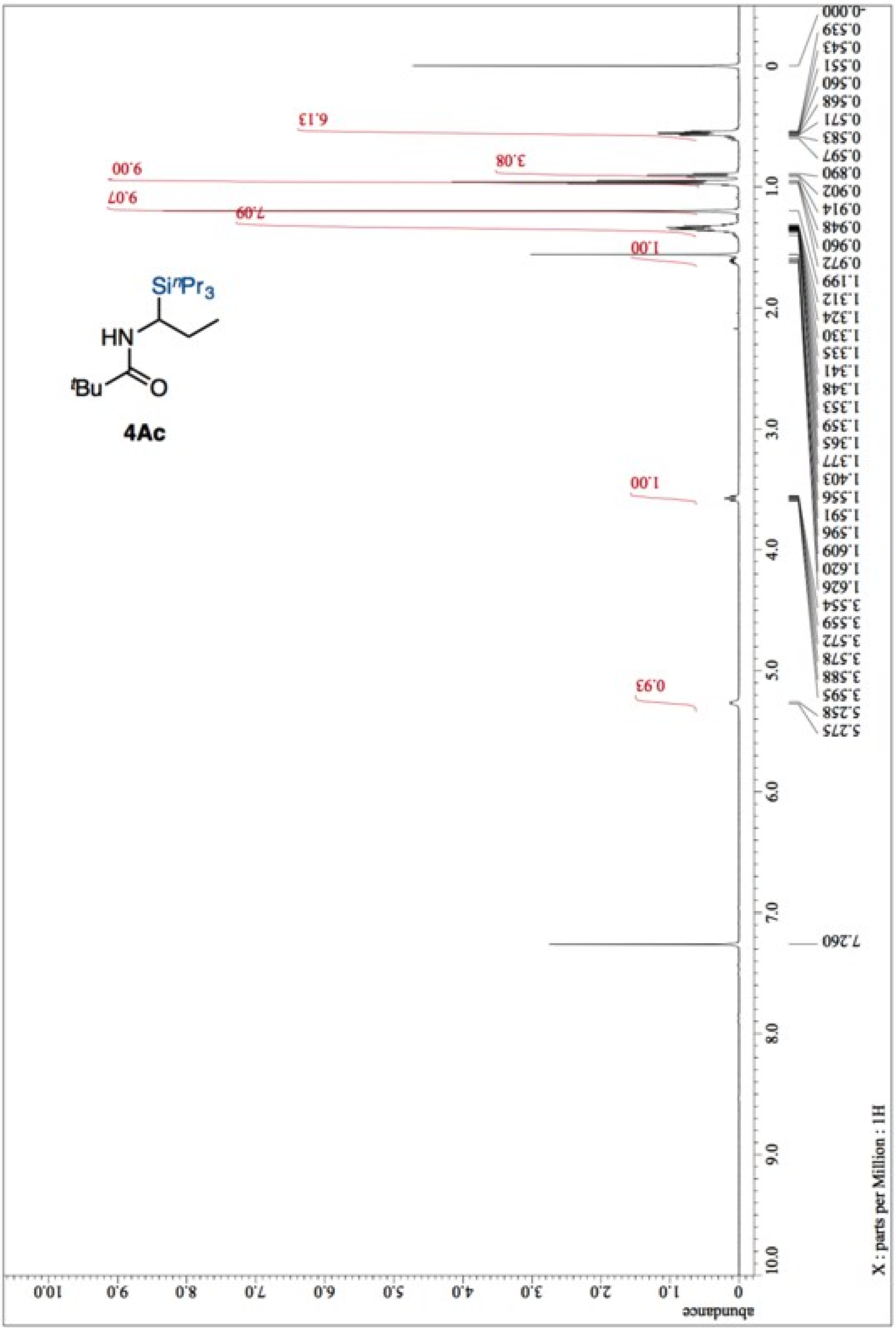

^{13}C NMR (151MHz, $CDCl_3$) of 4Ac

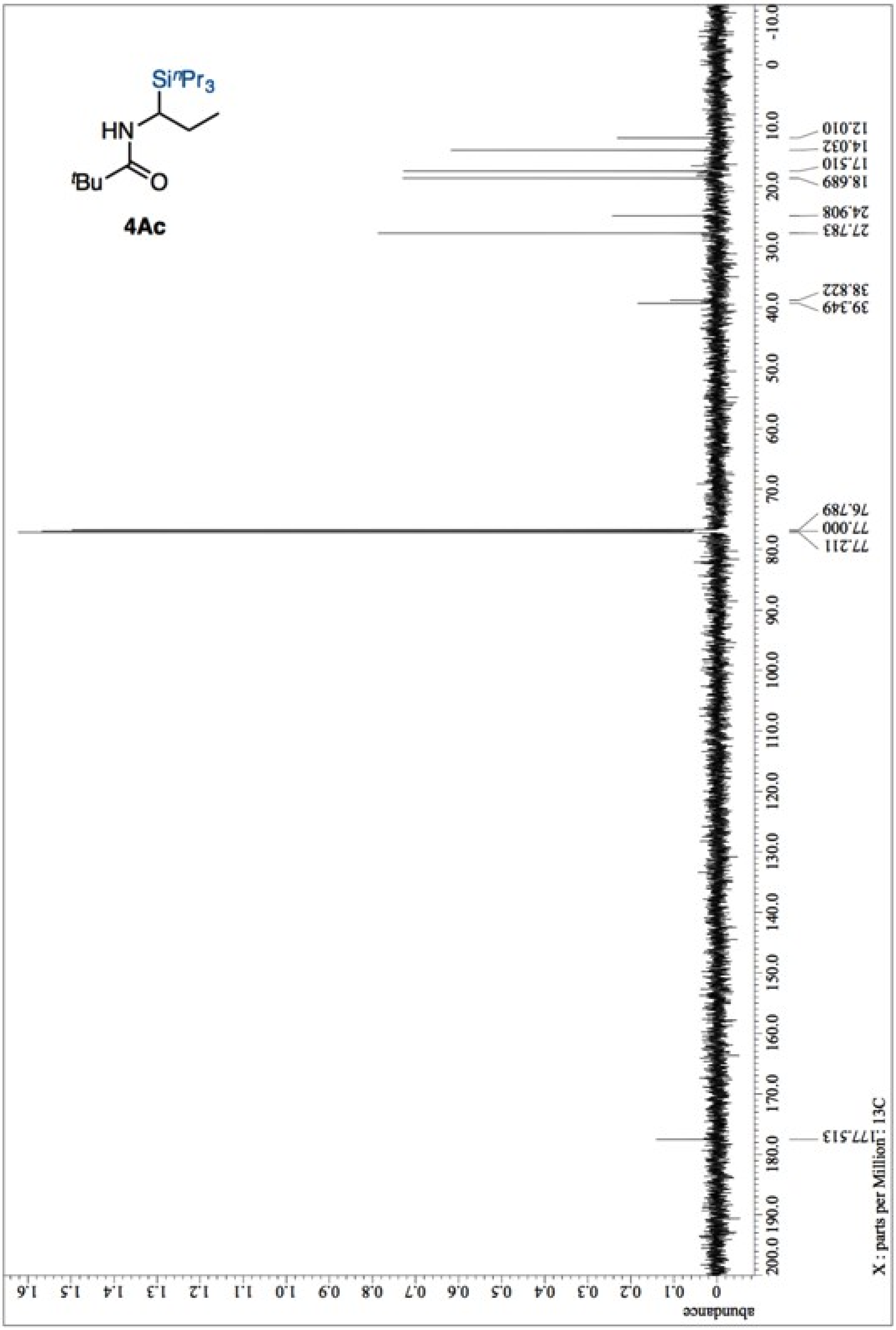

^{1}H NMR (600MHz, $CDCl_3$) of 4Ad

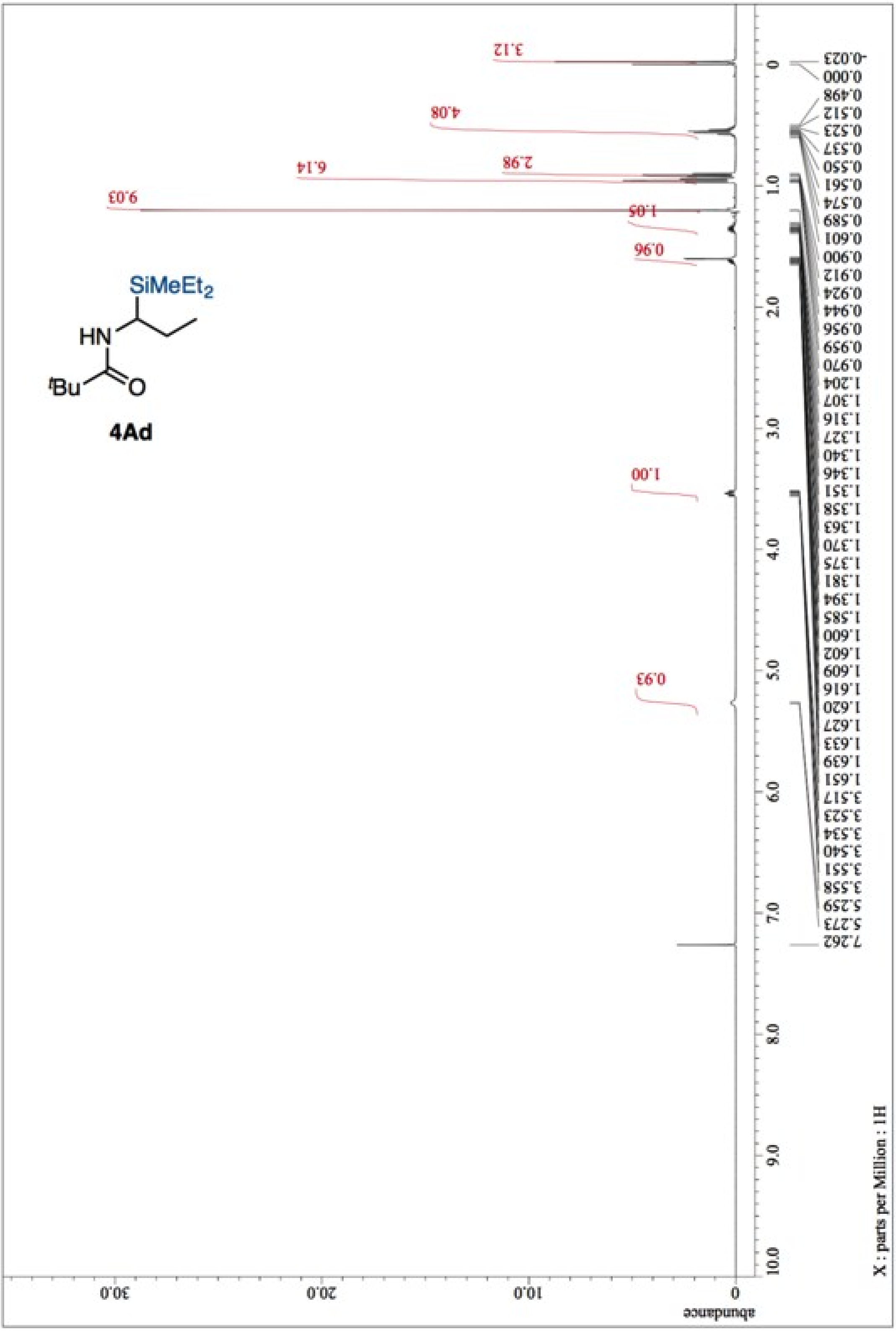

^{13}C NMR (151MHz, $CDCl_3$) of 4Ad

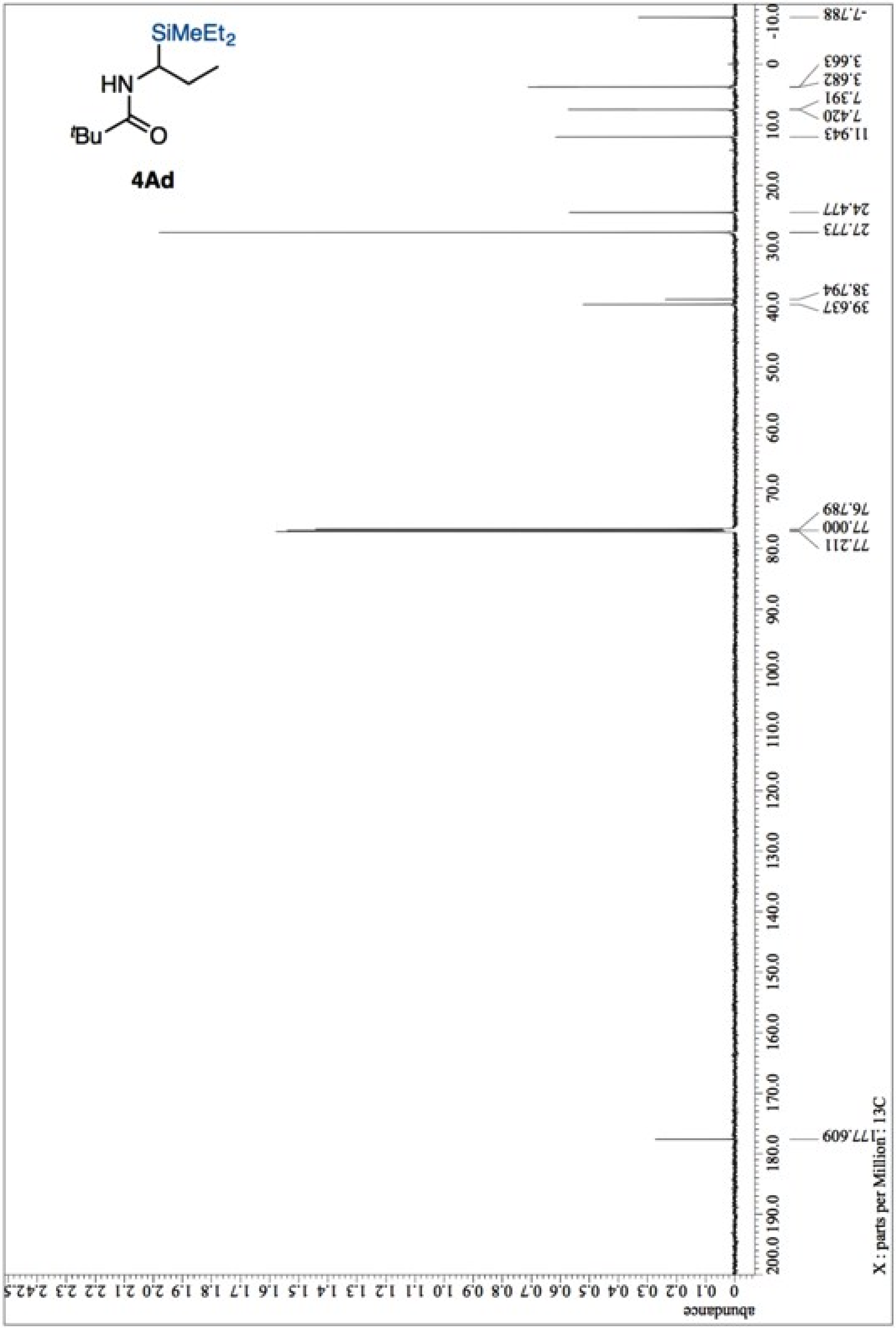

^{1}H NMR (600MHz, $CDCl_3$) of 4Ae

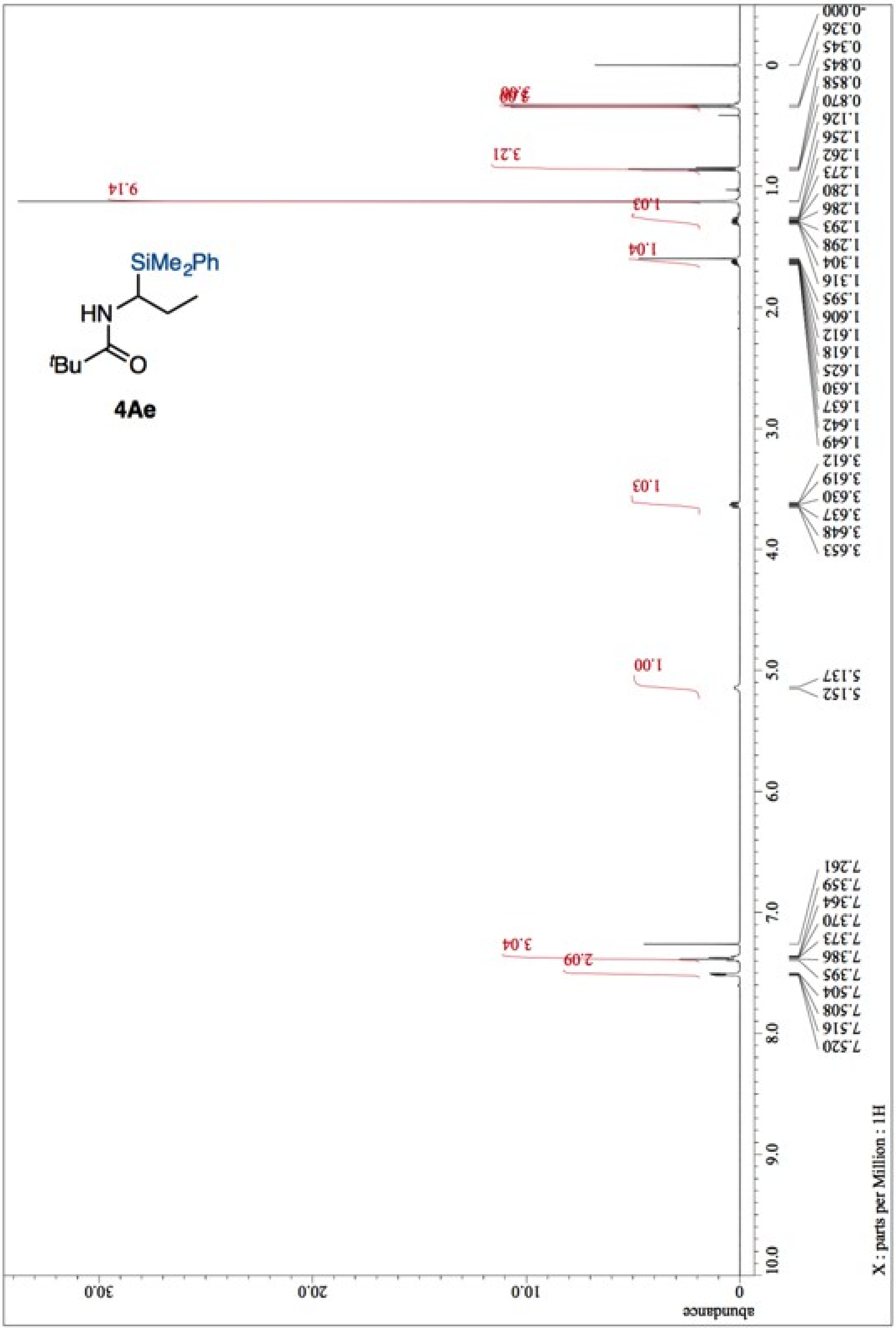

^{13}C NMR (151MHz, $CDCl_3$) of 4Ae

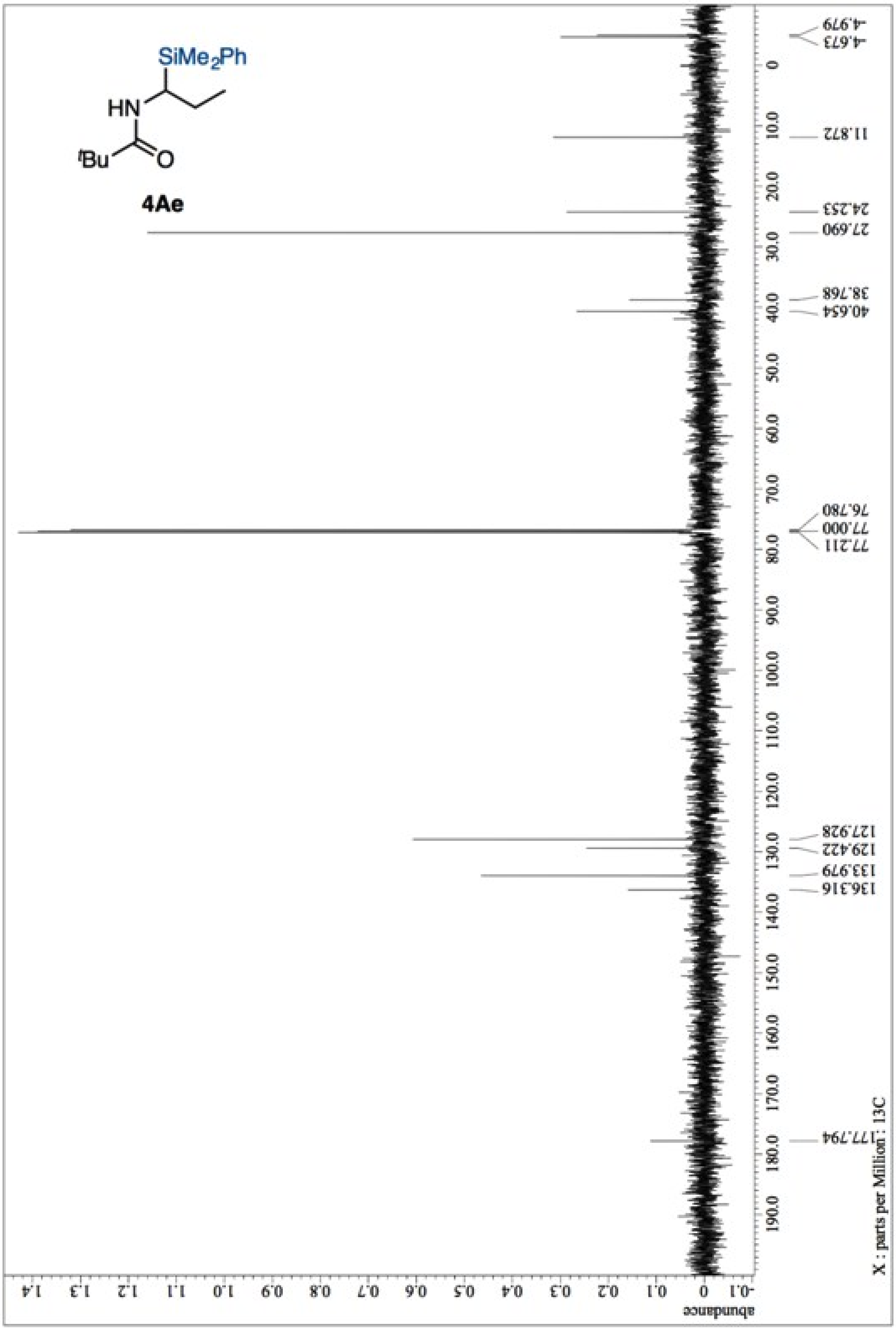

^{1}H NMR (600MHz, $CDCl_3$) of 3Aa

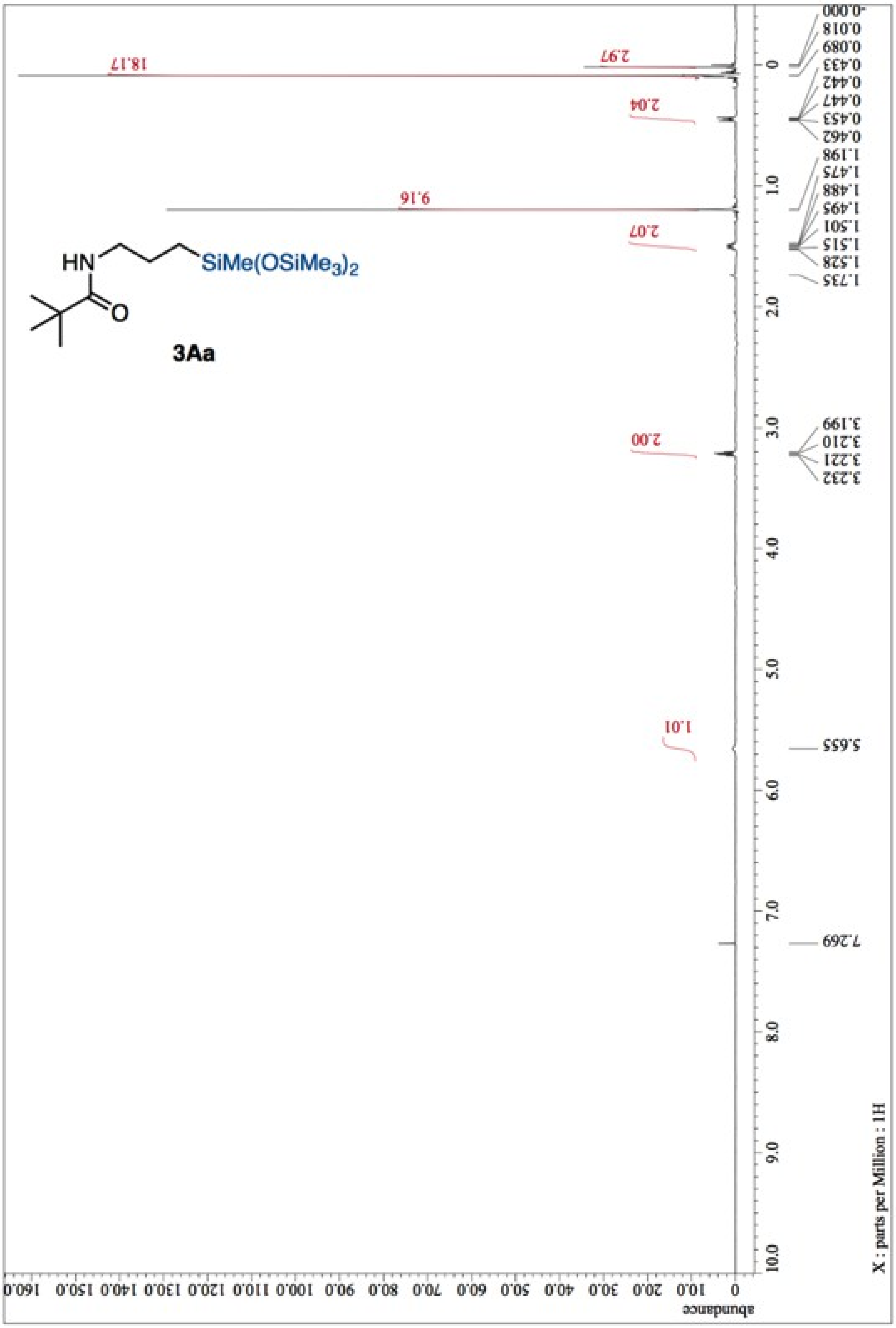

^{13}C NMR (151MHz, $CDCl_3$) of 3Aa

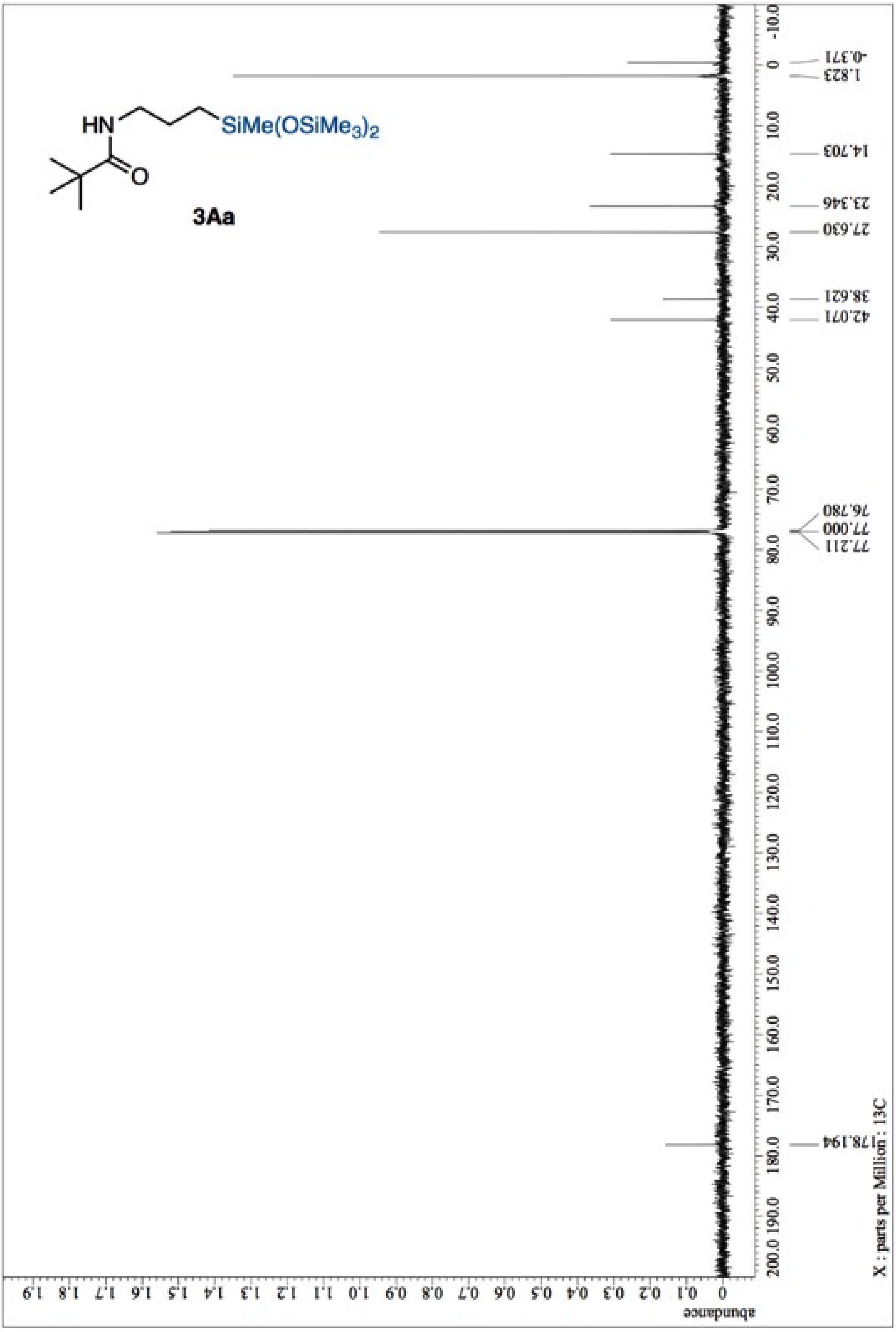

^{1}H NMR (600MHz, $CDCl_3$) of 3Ba

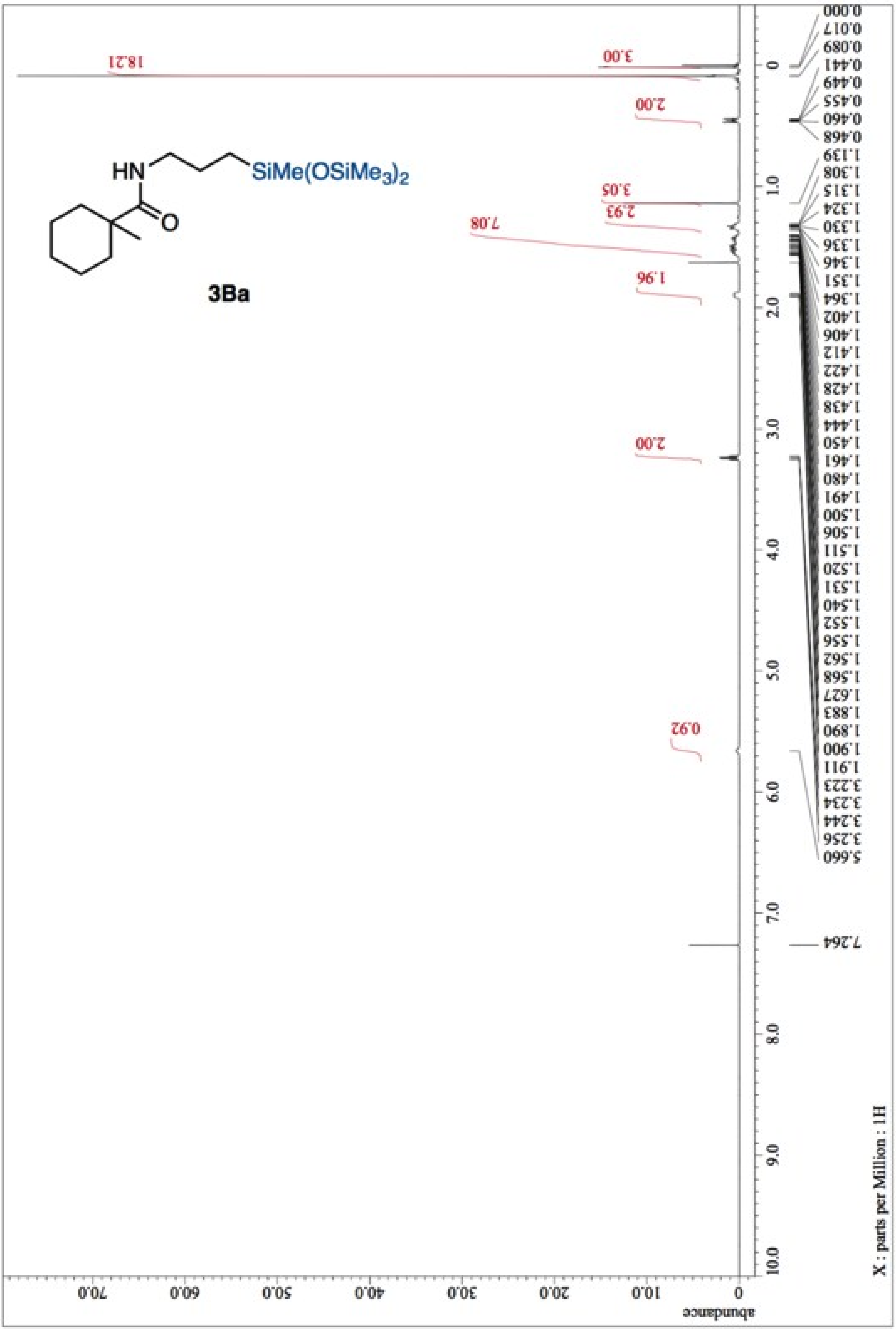

^{13}C NMR (151MHz, $CDCl_3$) of 3Ba

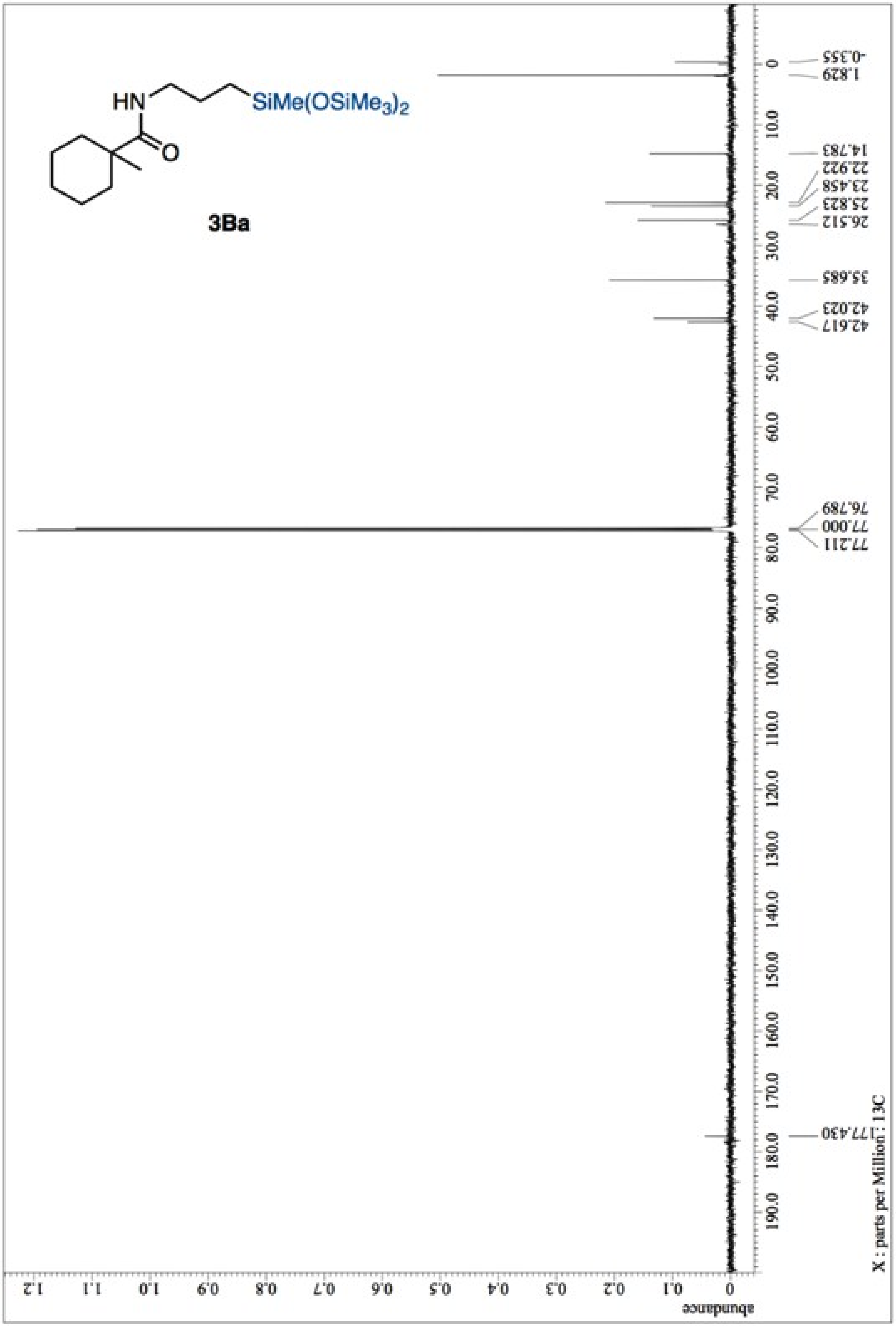

^{1}H NMR (600MHz, $CDCl_3$) of 3Ma

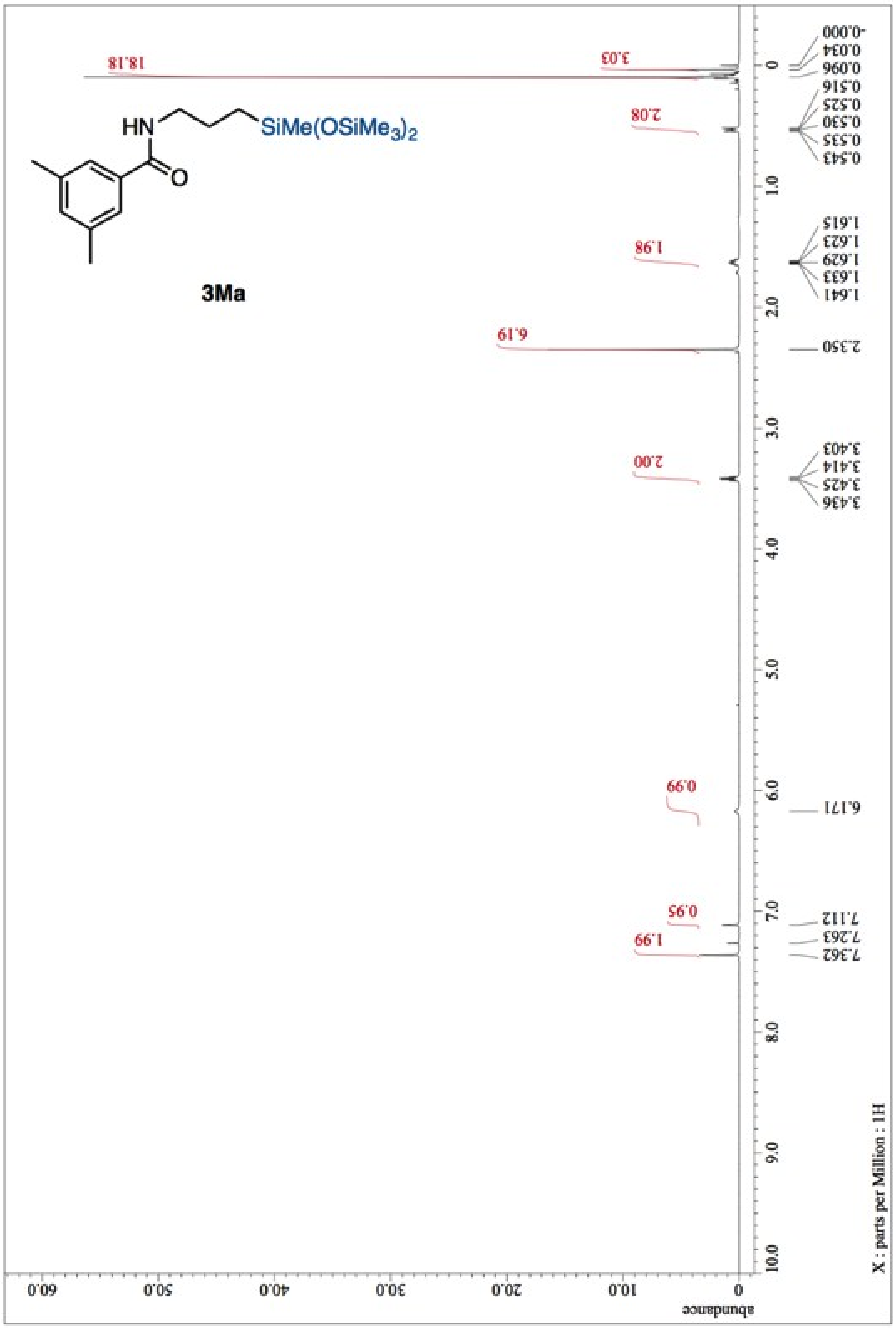

^{13}C NMR (151MHz, $CDCl_3$) of 3Ma

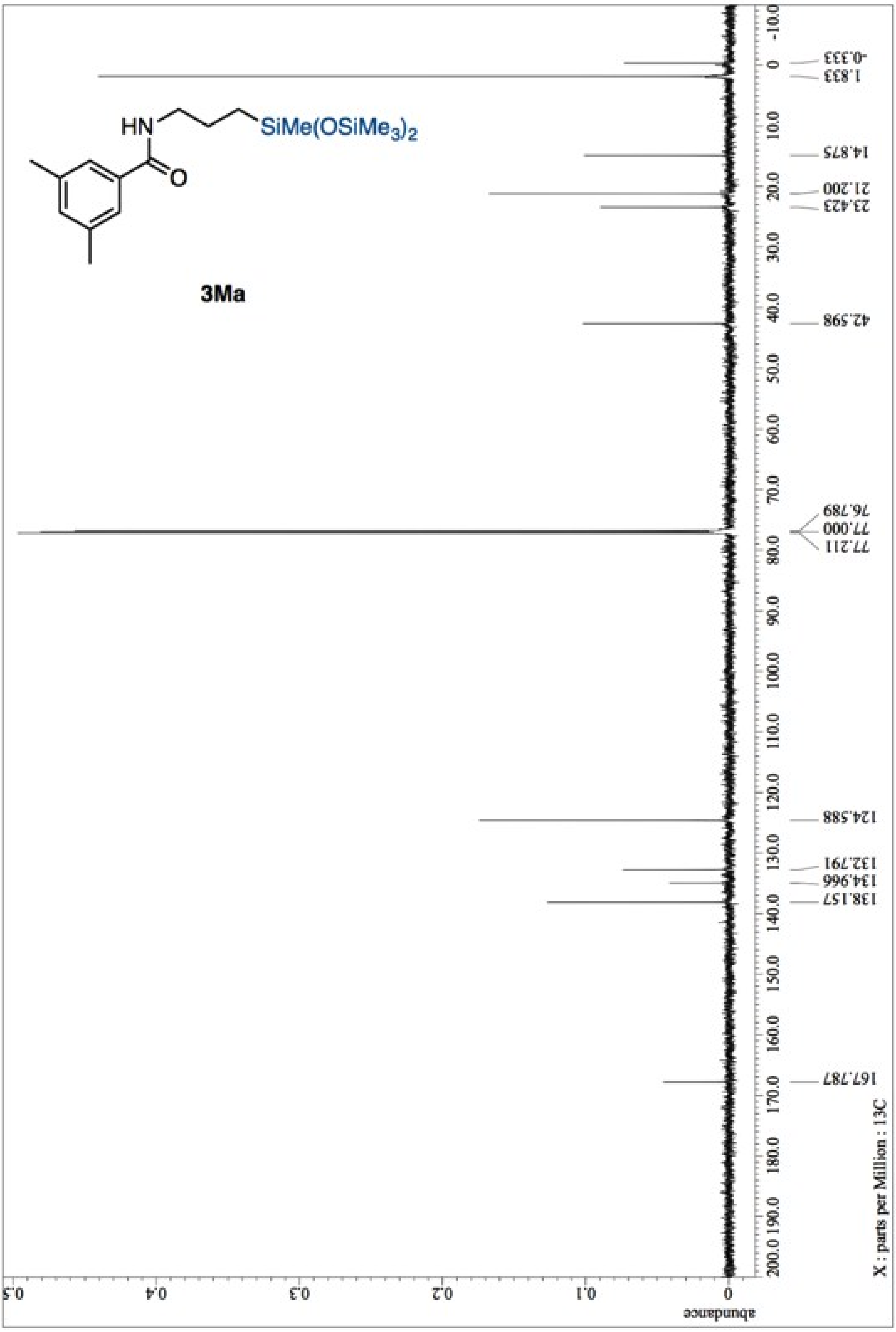

^{1}H NMR (600MHz, $CDCl_3$) of 3Na

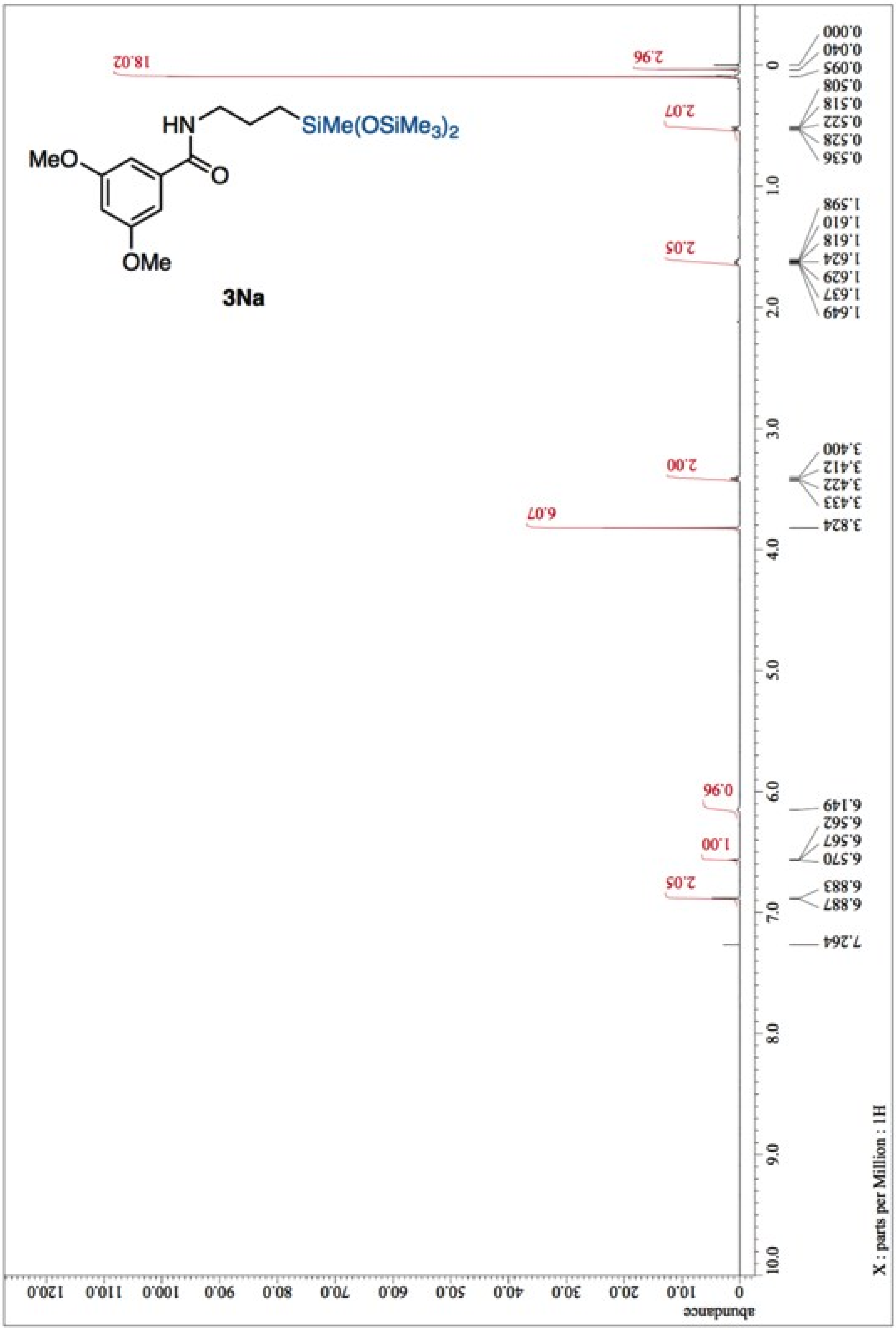

^{13}C NMR (151MHz, $CDCl_3$) of 3Na

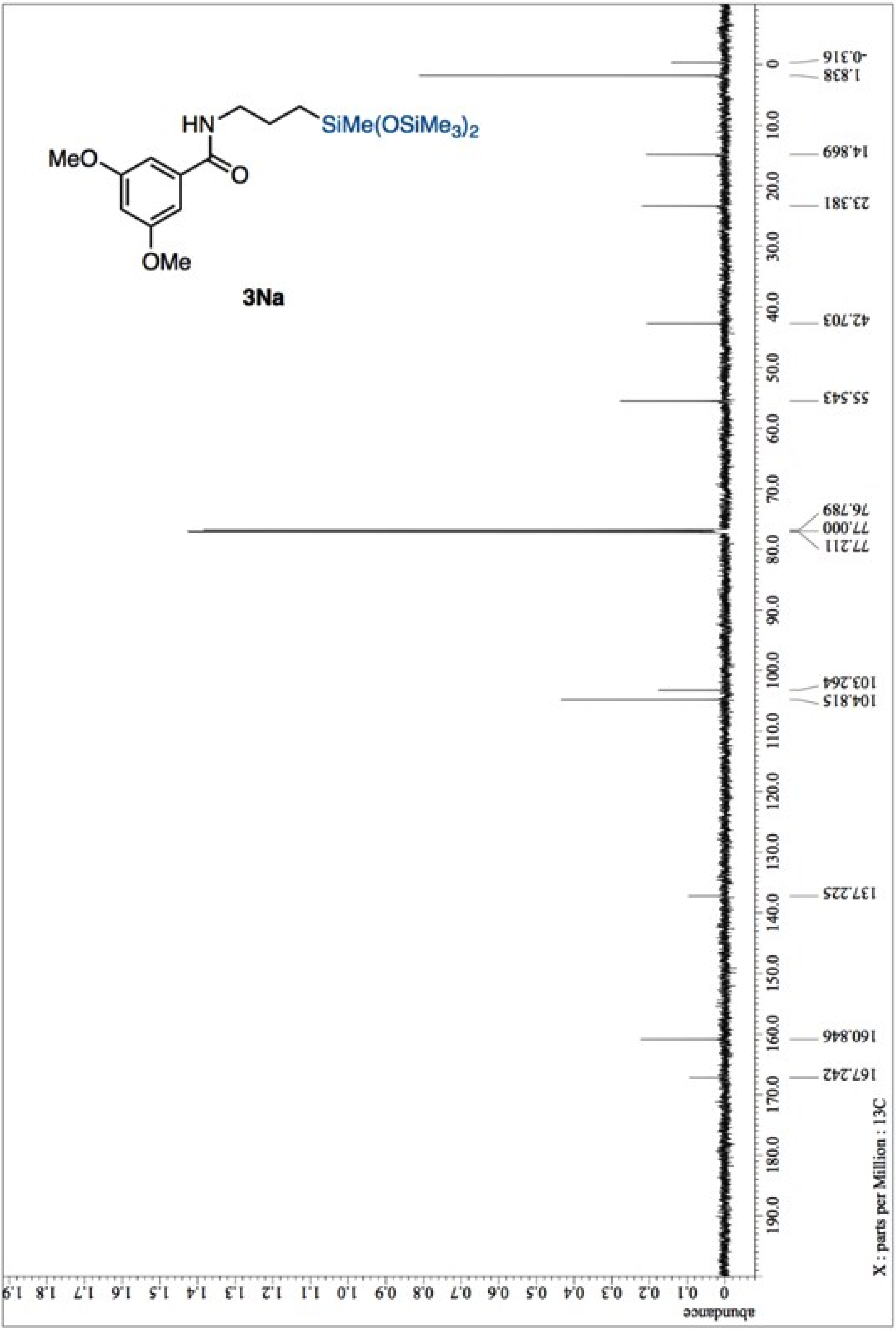

^{1}H NMR (600MHz, $CDCl_3$) of 3Ua

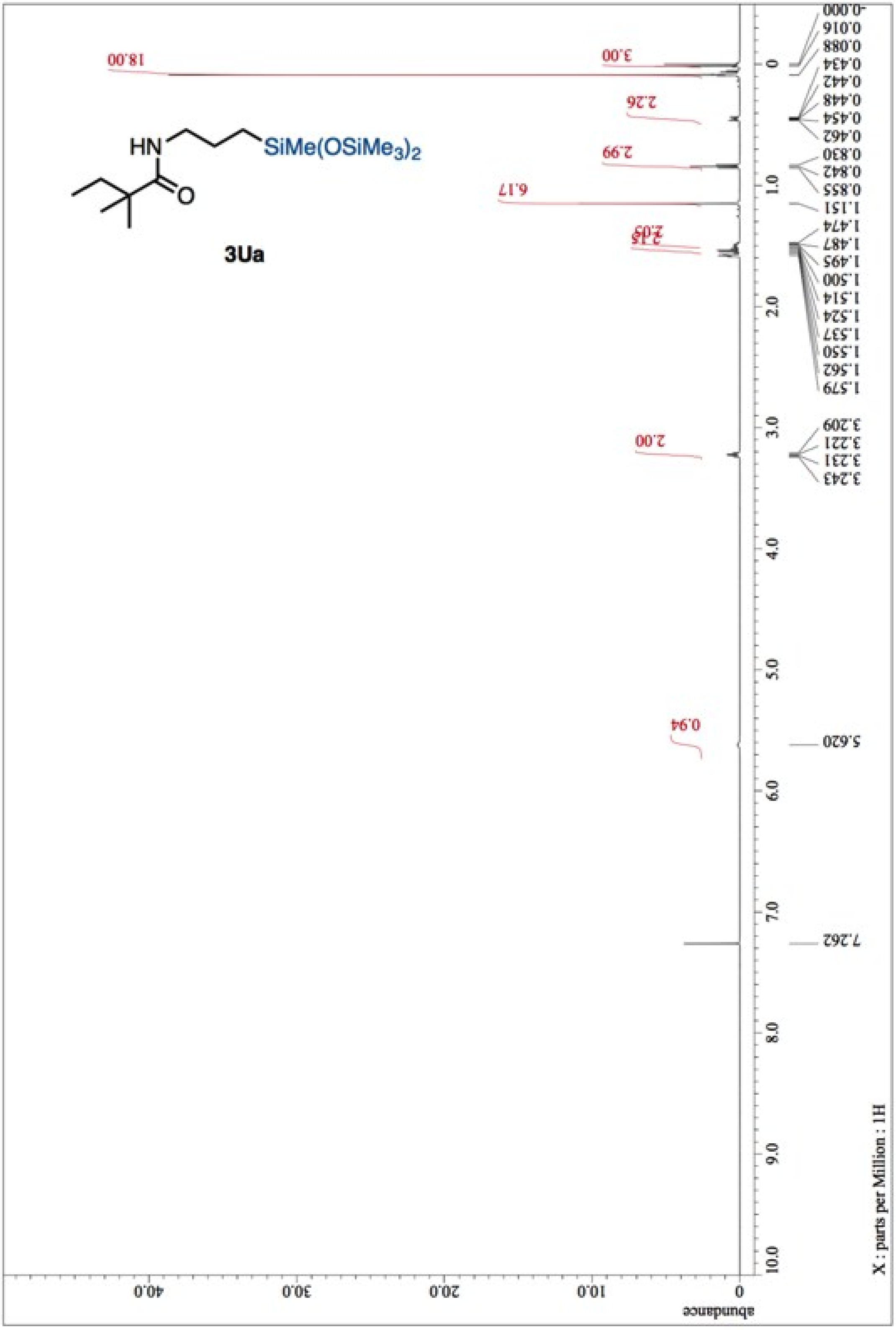

^{13}C NMR (151MHz, $CDCl_3$) of 3Ua

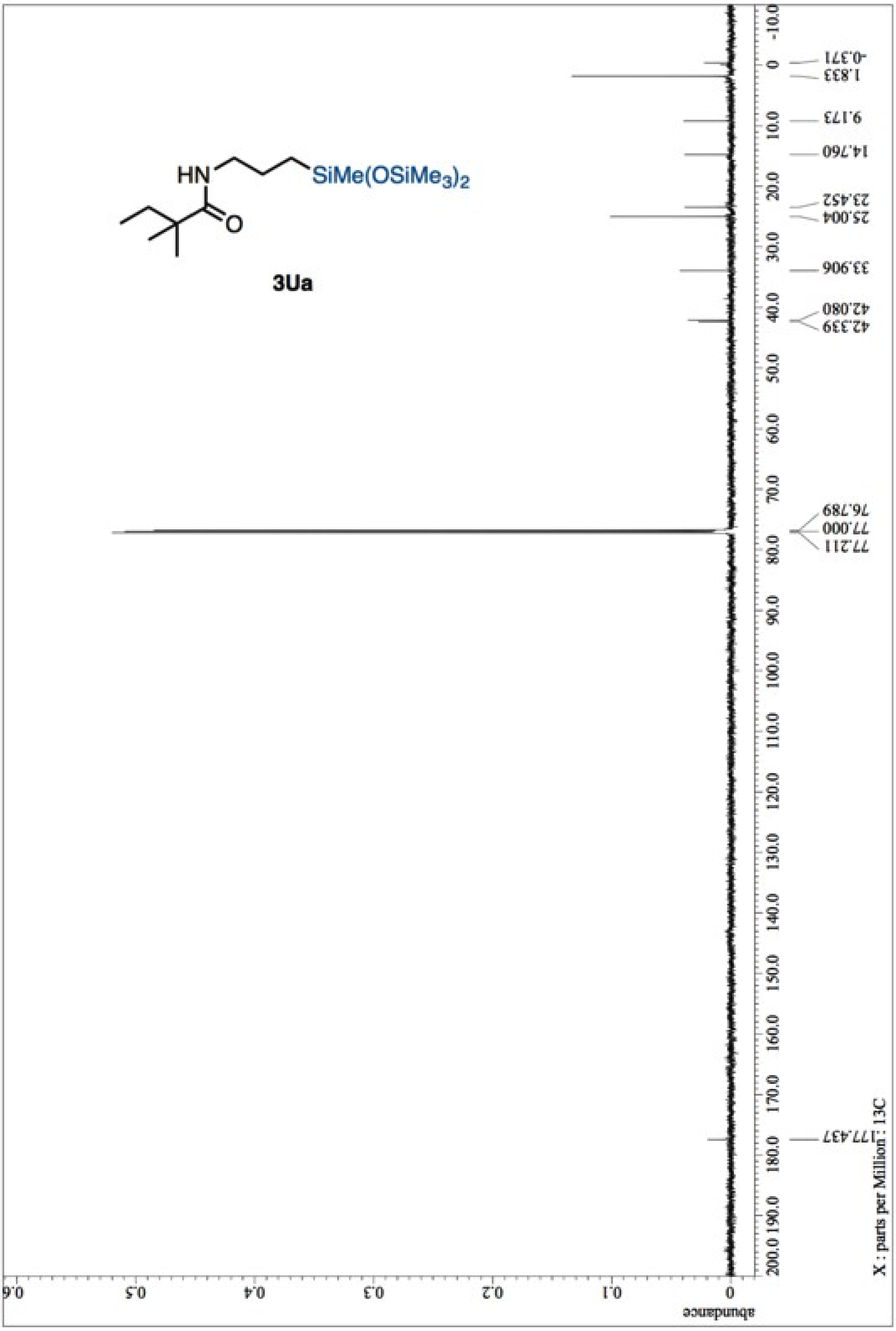

^{1}H NMR (600MHz, CDCl$_3$) of 3Va

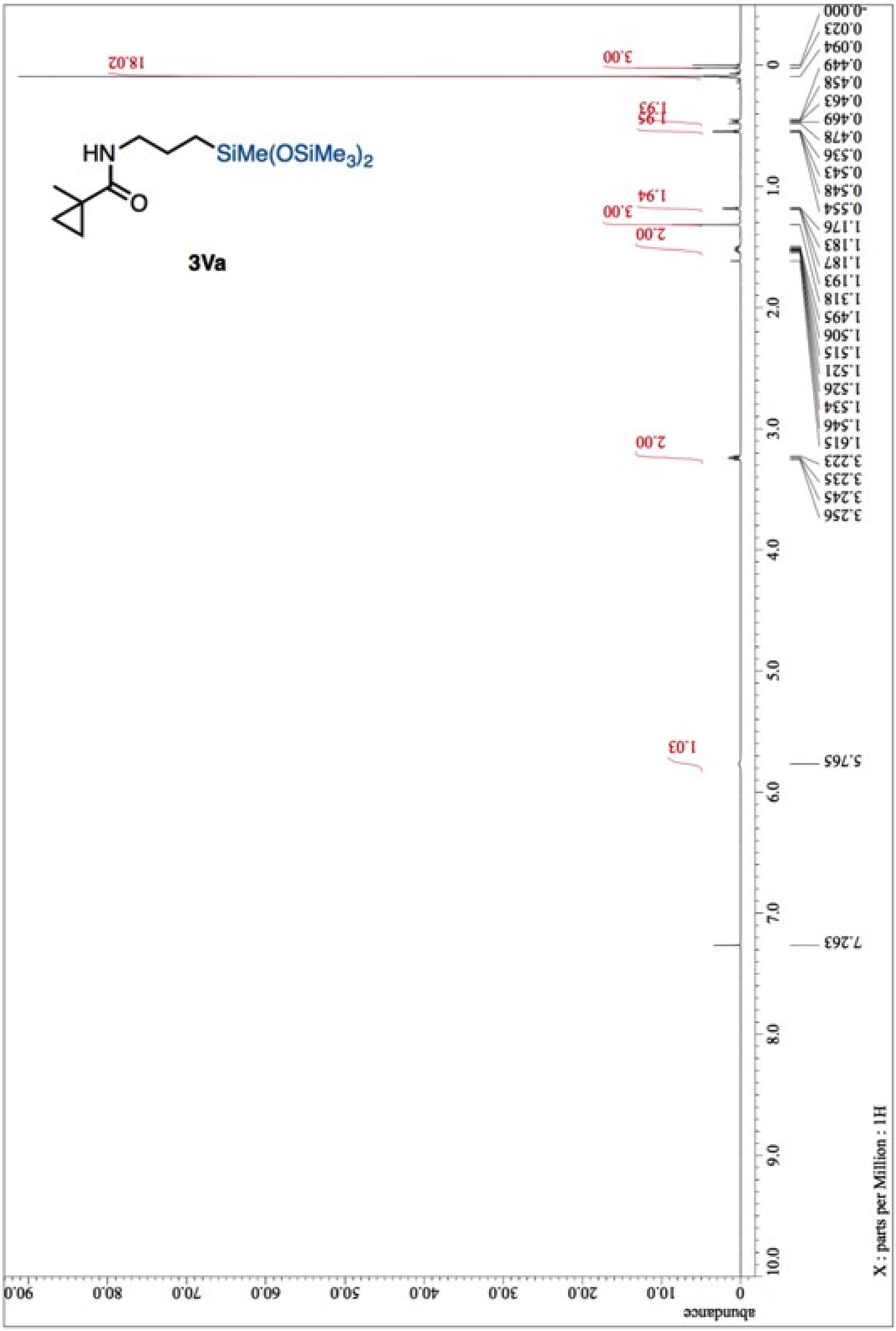

^{13}C NMR (151MHz, $CDCl_3$) of 3Va

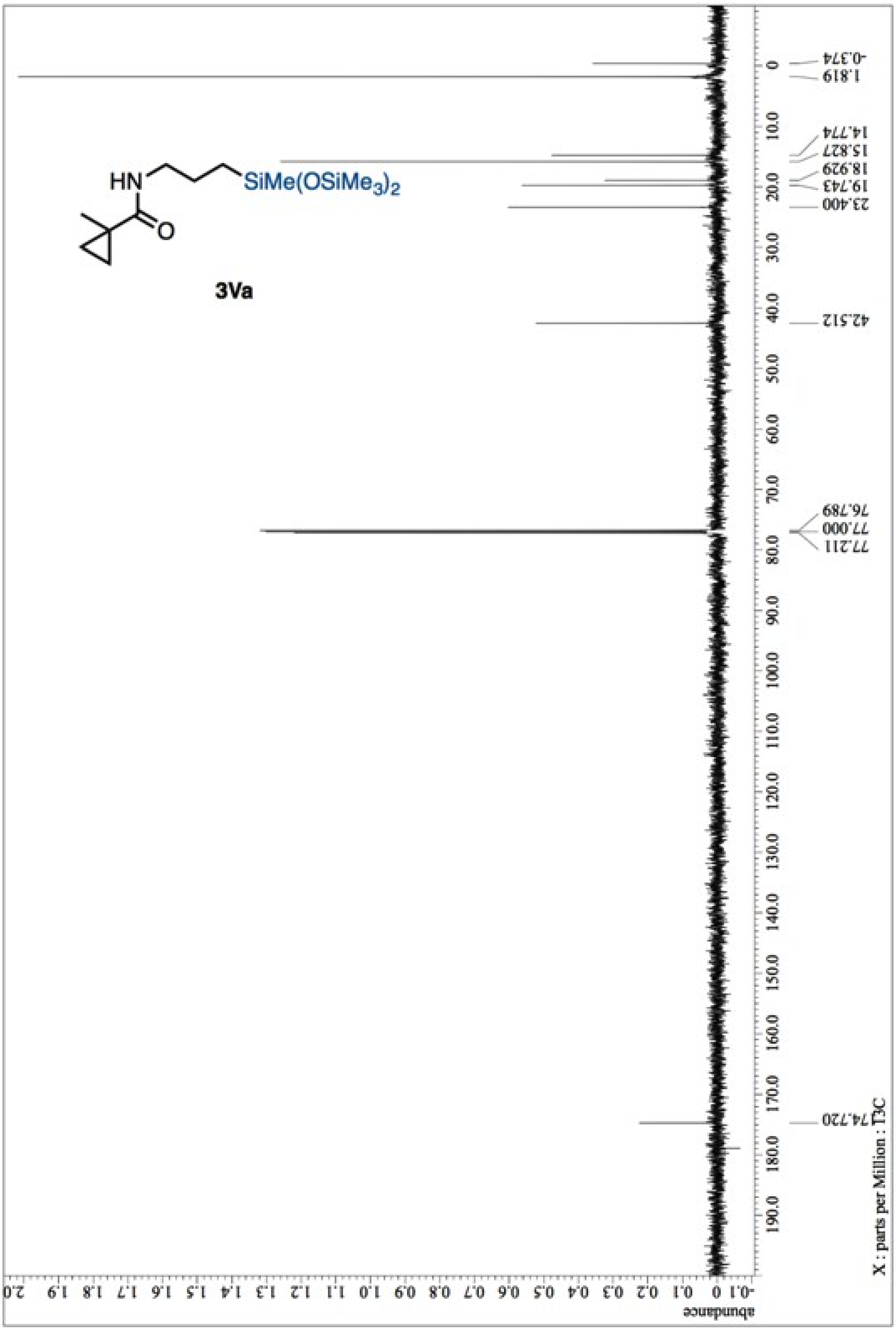

^{1}H NMR (500MHz, $CDCl_3$) of 5Ma

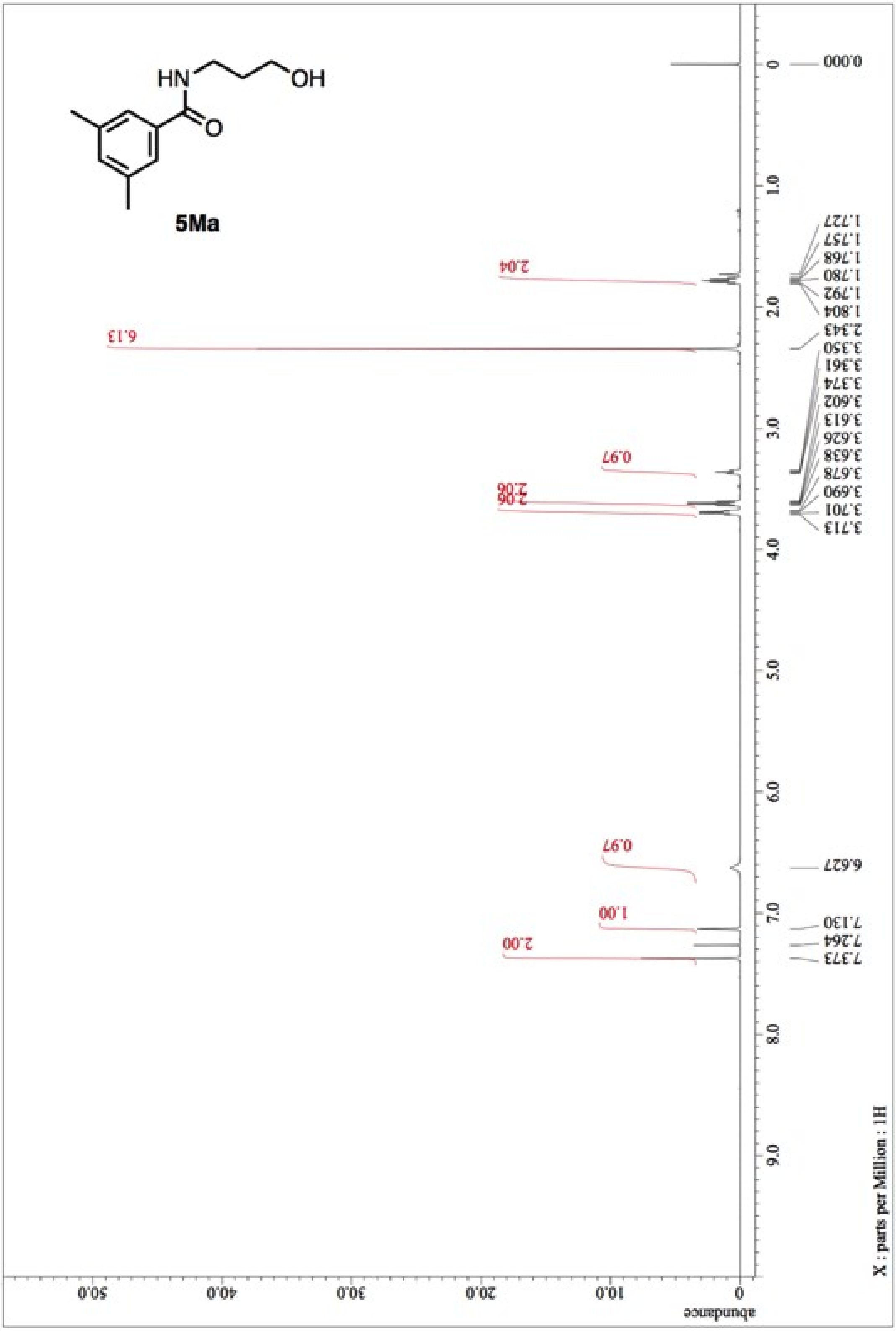

^{13}C NMR (126MHz, CDCl$_3$) of 5Ma

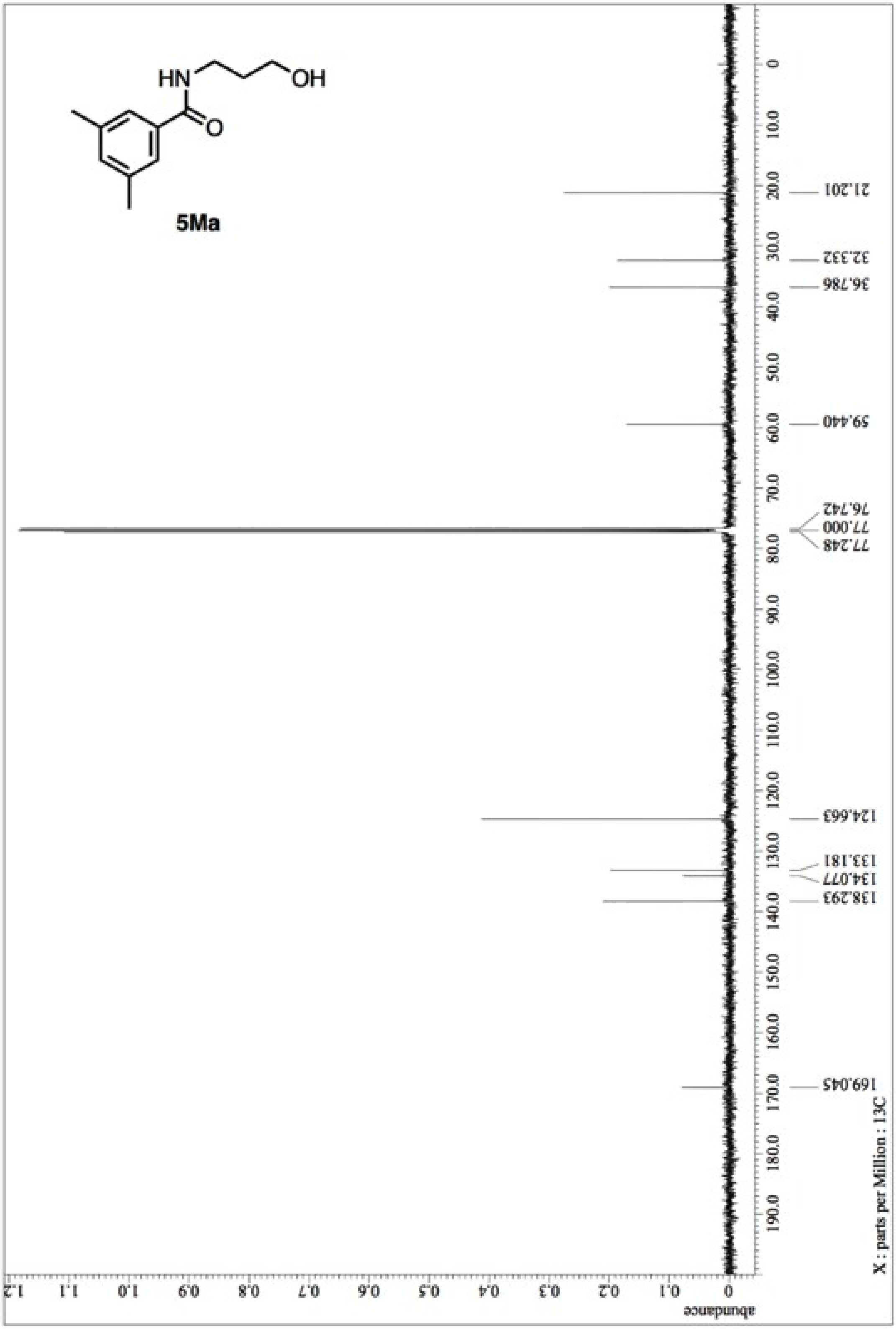

^{1}H NMR (600MHz, $CDCl_3$) of (*E*)-6A

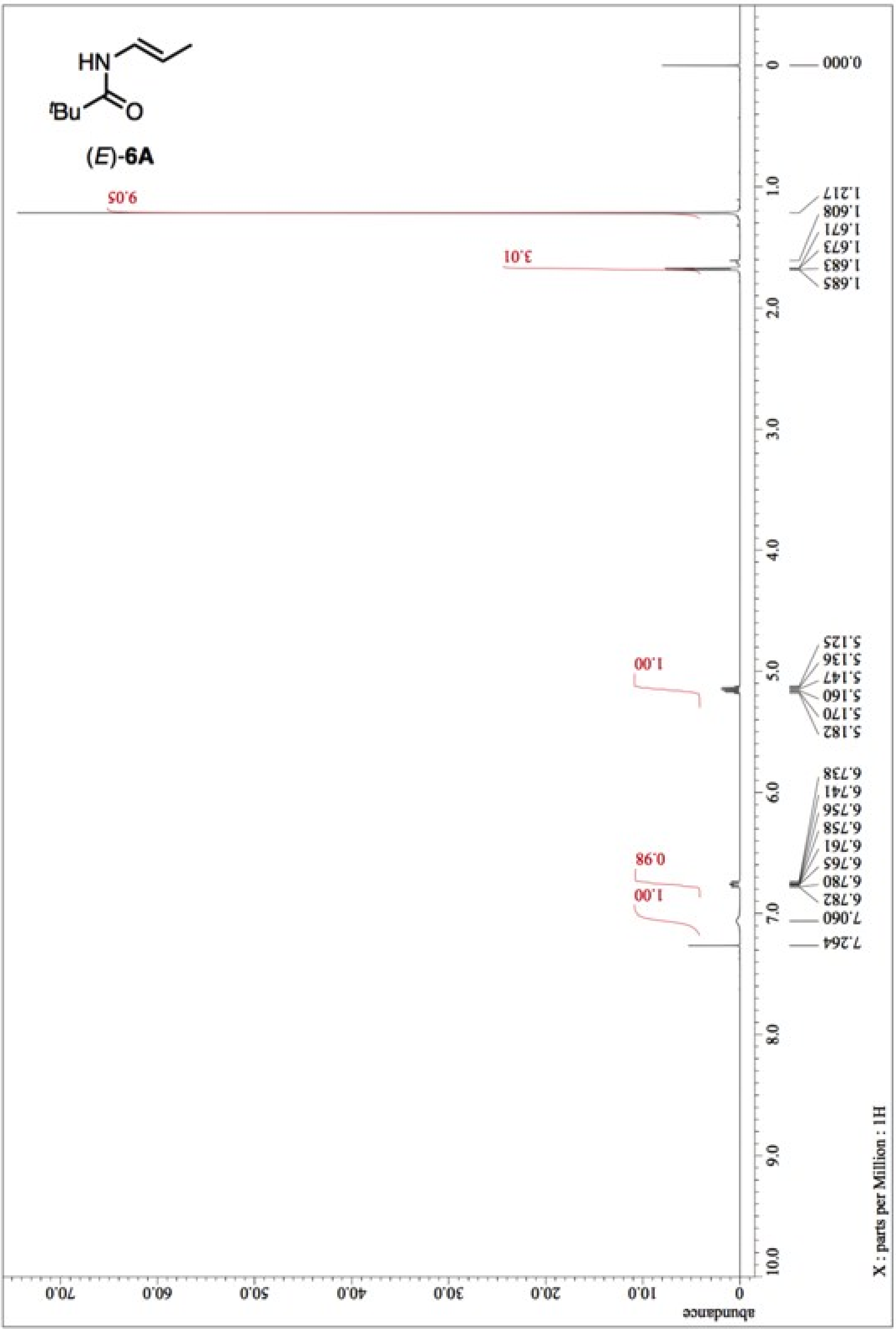

^{13}C NMR (151MHz, $CDCl_3$) of (*E*)-6A

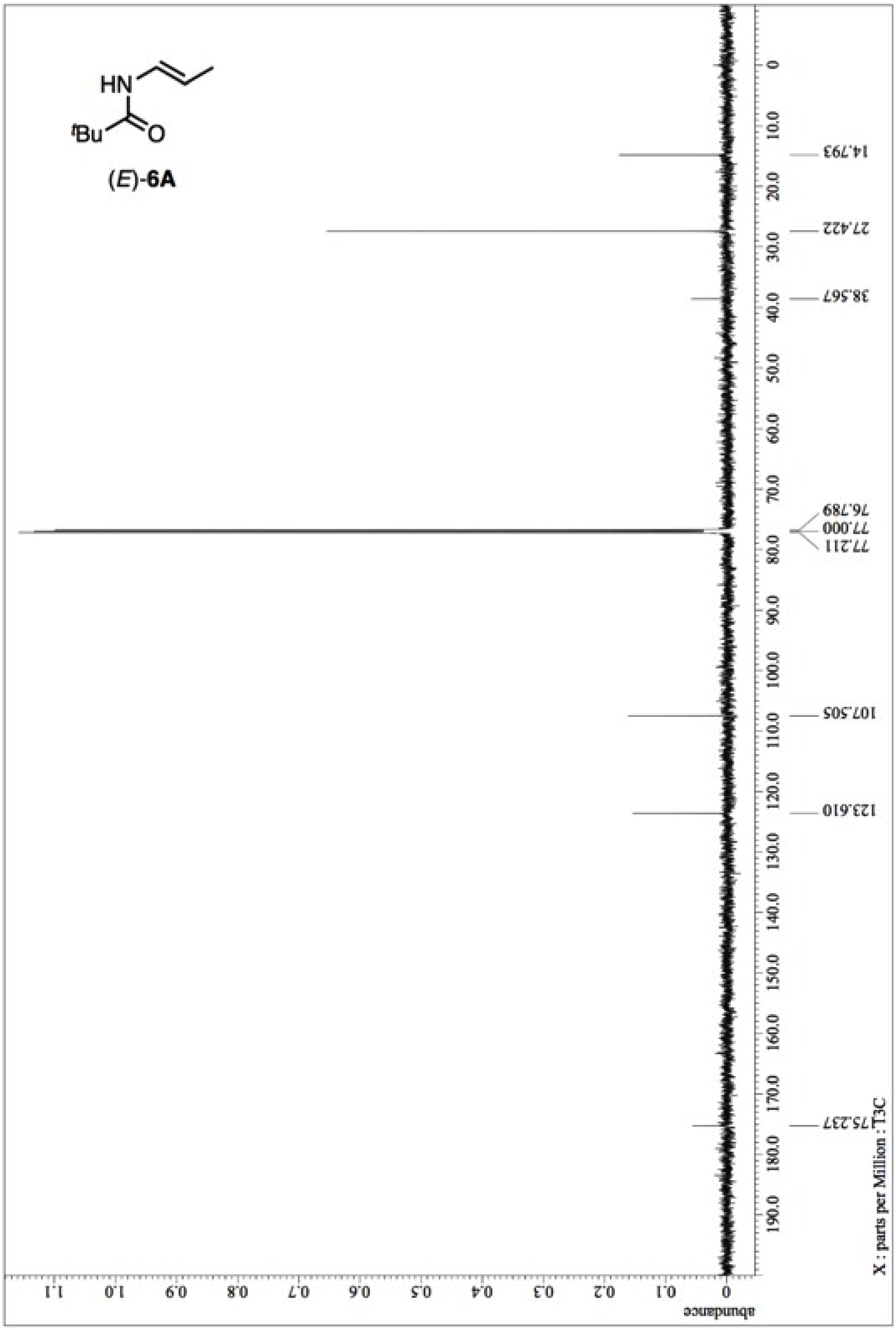

^{1}H NMR (600MHz, $CDCl_3$) of (*Z*)-6A

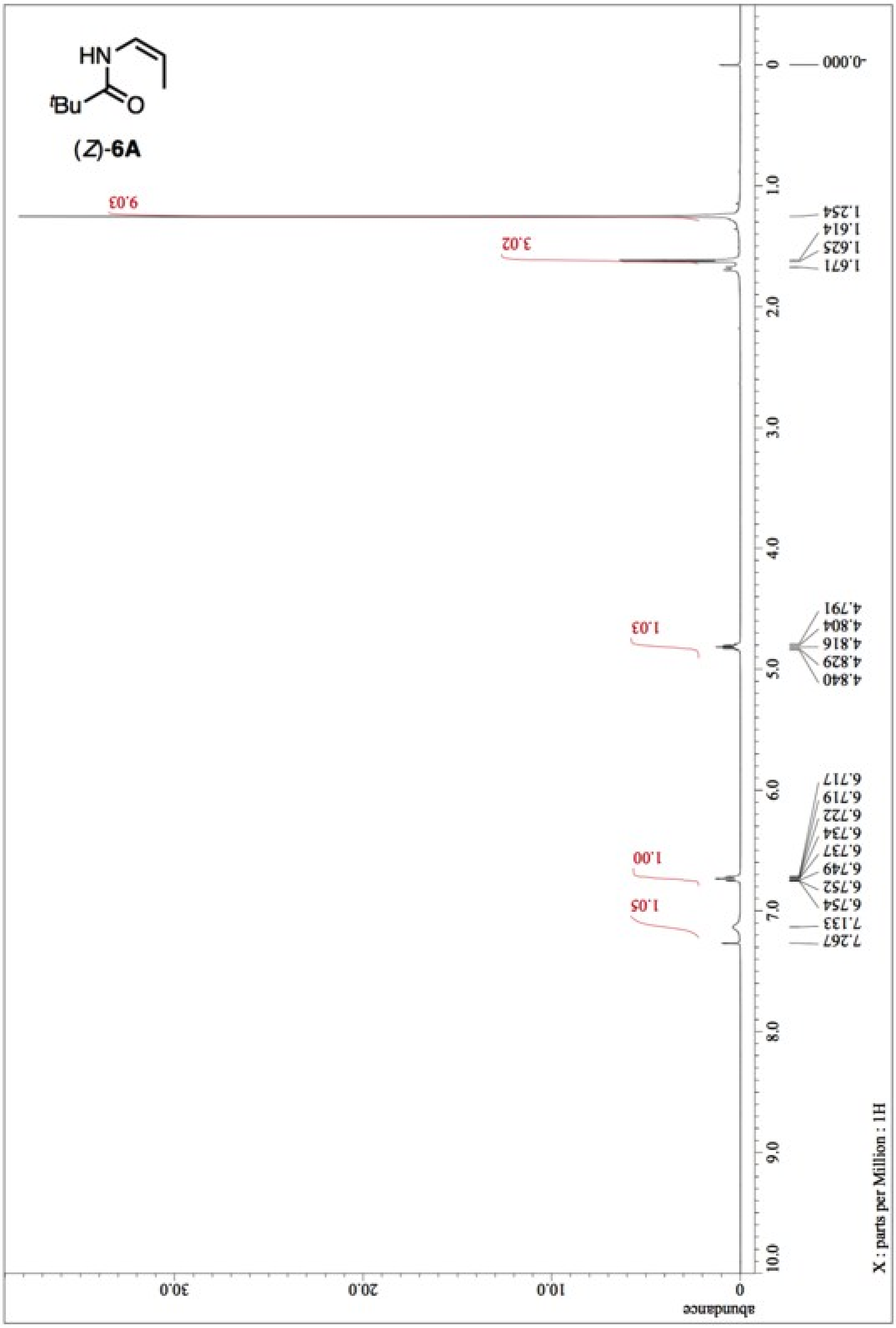

^{13}C NMR (151MHz, $CDCl_3$) of (*Z*)-6A

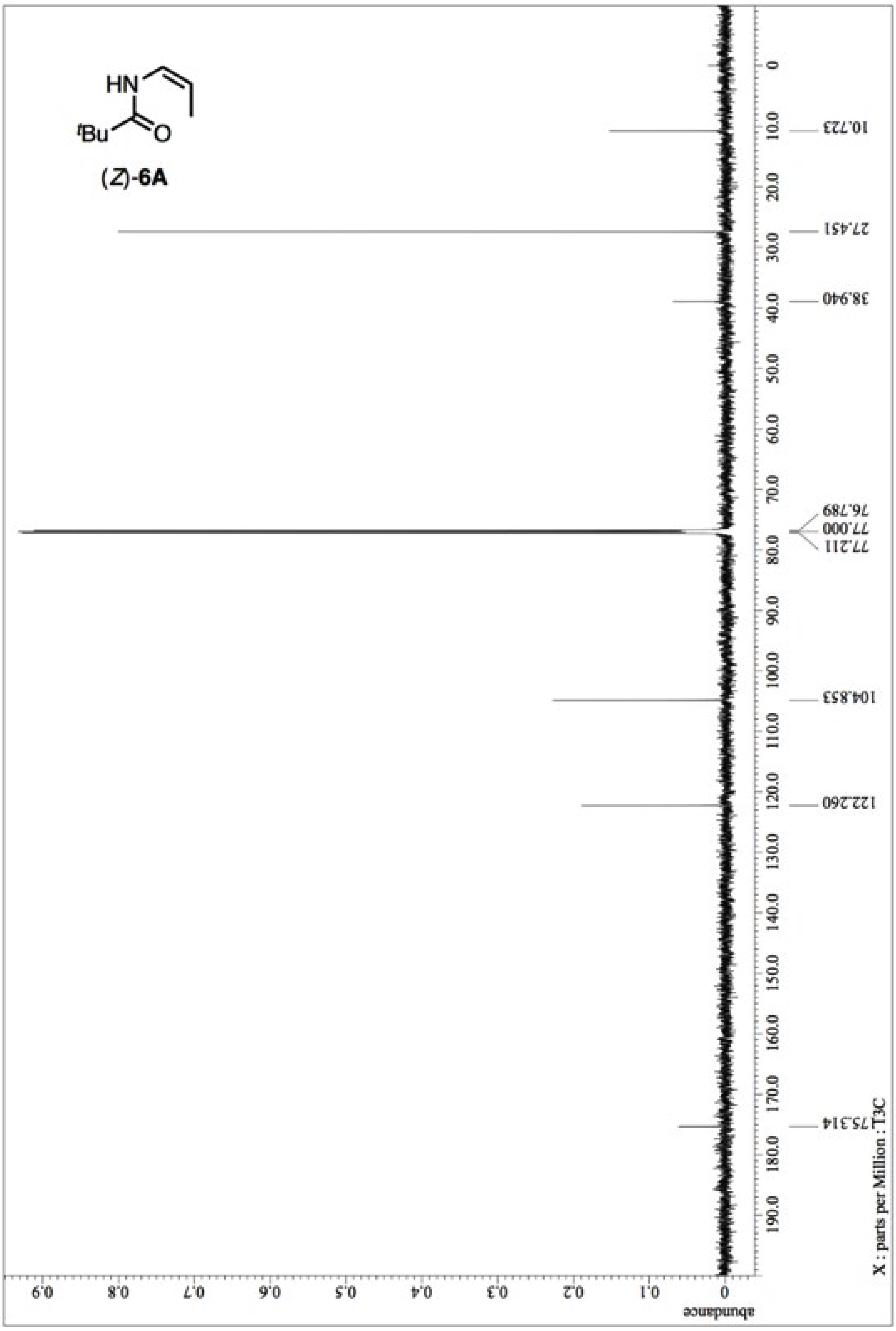

^{1}H NMR (600MHz, CD_2Cl_2) of 9Aa

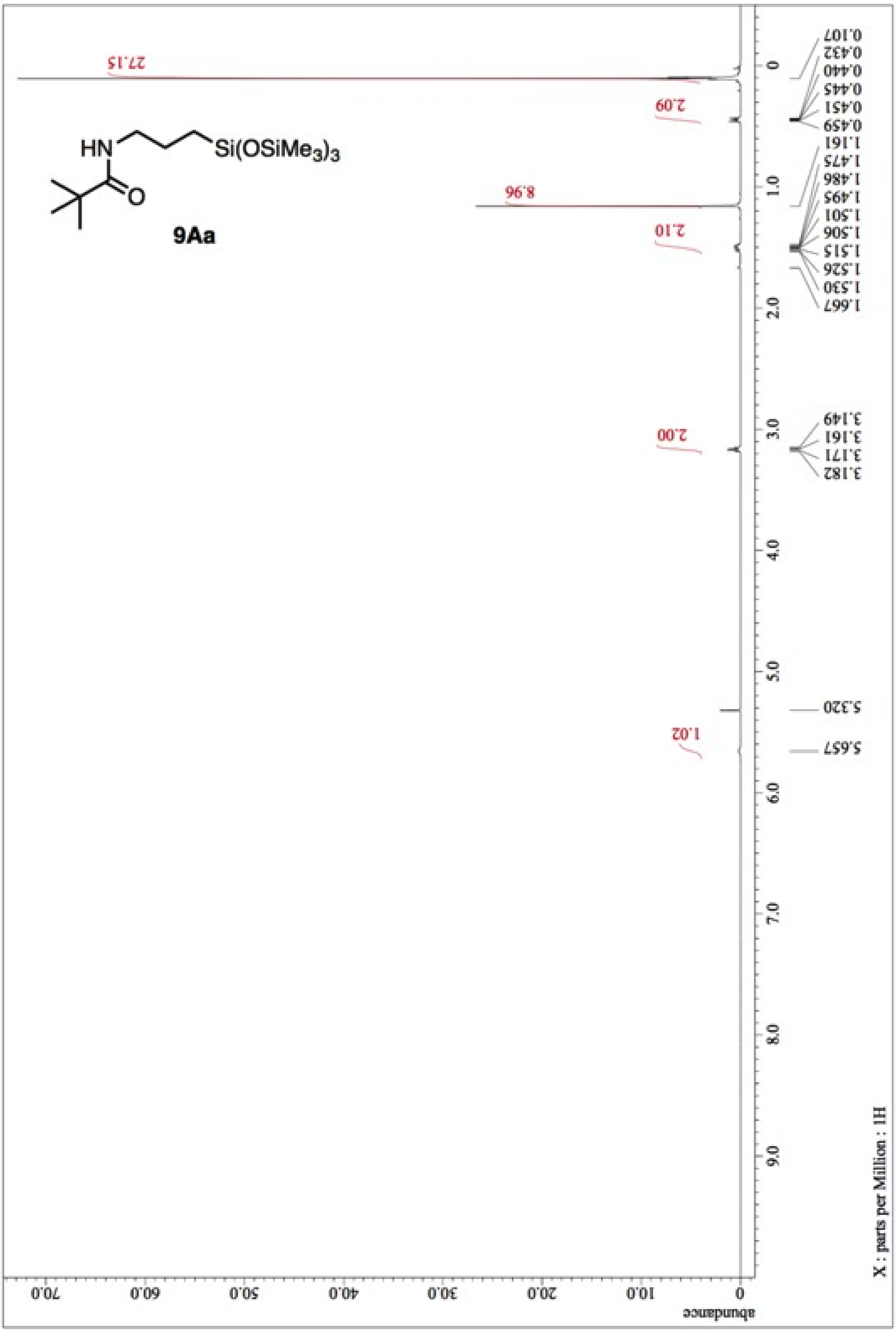

^{13}C NMR (151MHz, CD_2Cl_2) of 9Aa

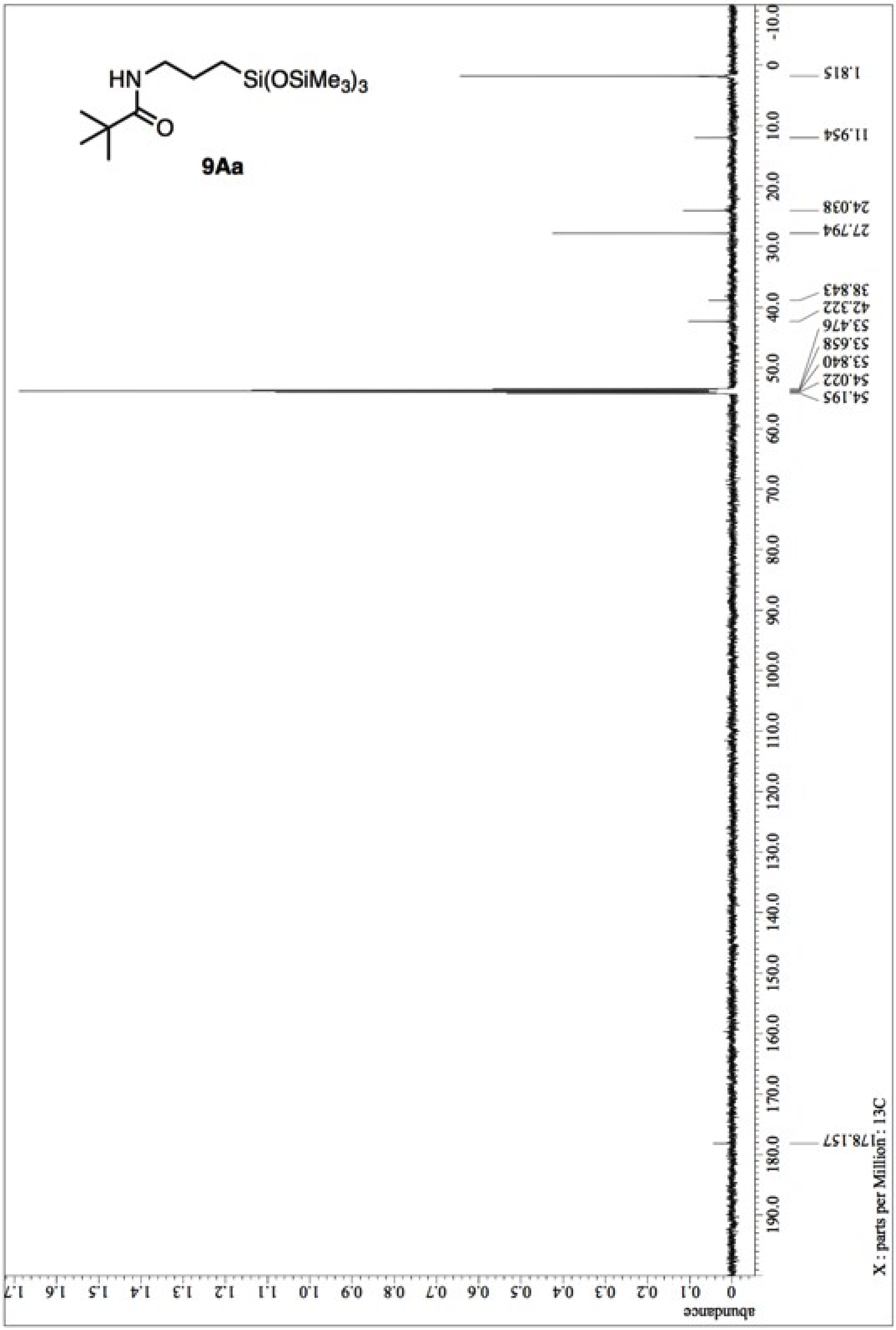

Supplement: Supplementary file 1 [file SC-008-C7SC00071E-s001.pdf]
